# Design, synthesis, *in silico* and *in vitro* evaluation of pyrrole–indole hybrids as dual tubulin and aromatase inhibitors with potent anticancer activities†

**DOI:** 10.1039/d4ra09000d

**Published:** 2025-06-27

**Authors:** Rungroj Saruengkhanphasit, Jaruwan Chatwichien, Lukana Ngiwsara, Kriengsak Lirdprapamongkol, Worawat Niwetmarin, Chatchakorn Eurtivong, Prasat Kittakoop, Jisnuson Svasti, Somsak Ruchirawat

**Affiliations:** a Chulabhorn Graduate Institute, Program in Chemical Sciences 54 Kamphaeng Phet 6, Talat Bang Khen, Lak Si Bangkok 10210 Thailand rungrojs@cgi.ac.th +66 25541900 ext. 2629; b Center of Excellence on Environmental Health and Toxicology (EHT), OPS, Ministry of Higher Education, Science, Research and Innovation Bangkok Thailand; c Chulabhorn Royal Academy Bangkok 10210 Thailand; d Laboratory of Biochemistry, Chulabhorn Research Institute Bangkok 10210 Thailand; e Department of Pharmaceutical Chemistry, Faculty of Pharmacy, Mahidol University 447 Si Ayutthaya Road, Ratchathewi Bangkok 10400 Thailand chatchakorn.eur@mahidol.edu +66 26448677-91 ext. 5402; f Laboratory of Natural Products, Chulabhorn Research Institute Bangkok 10210 Thailand; g Laboratory of Medicinal Chemistry, Chulabhorn Research Institute Bangkok 10210 Thailand

## Abstract

Twenty-four new pyrrolyl-3-phenyl-1*H*-indole-2-carbohydrazide derivatives were designed, synthesized and evaluated for their anticancer activities and dual inhibition properties against tubulin and aromatase. Their anticancer activities were highly potent against the NCI60 human cancer cell line panel. Amongst them, single chloro-substituted derivative 3h was the strongest tubulin inhibitor, disrupting the microtubule structure by inhibiting the colchicine site, while potently inhibiting aromatase (IC_50_ = 1.8 µM) with strong activity against the estrogen receptor-positive T47D breast cancer cell line (IC_50_ = 2.4 µM). Ester derivative 3k showed the best aromatase inhibitory activity (IC_50_ = 18 nM) with moderate anti-T47D activity (IC_50_ = 10.6 µM). Molecular docking predicted the derivatives inhibited the colchicine site of tubulin by forming mainly hydrophobic interactions with the surrounding amino acid residues. Moreover, heme chelation with the pyrrole ring was predicted as a key interaction, and the formation of intermolecular bonds with adjacent amino acid residues was predicted as important for inhibitory activity.

## Abstract 2

The study introduces a new series of pyrrolyl-3-phenyl-1*H*-indole-2-carbohydrazide derivatives which were rationalized and designed as potent dual inhibitors of two oncologic targets, tubulin and aromatase.

## Graphical abstract

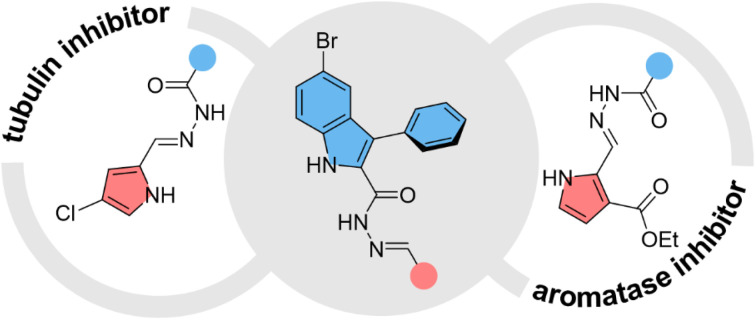

## 1. Introduction

Cancer has been reported by the World Health Organization (WHO) as a leading cause of death, with almost 10 million deaths in 2020.^1^ Furthermore, the WHO estimated that there would be around 20 million new cancer cases and 9.7 million deaths globally in 2022, and also projected that by 2050, there would be a 77% increase in new cases.^2^ Statistics revealed that lung (12.6%), breast (11.6%), and colon (9.6%) cancers were the first, second and third most common types of new cases in 2022, respectively.^2^ In the same report, lung cancer was the most common cause of deaths at 18.7% followed by colon cancer at 9.3%, and liver cancer and breast cancer at 7.8% and 6.9%, respectively. These statistics clearly indicate that cancer is a major global health burden.^2^

**Fig. 1:**
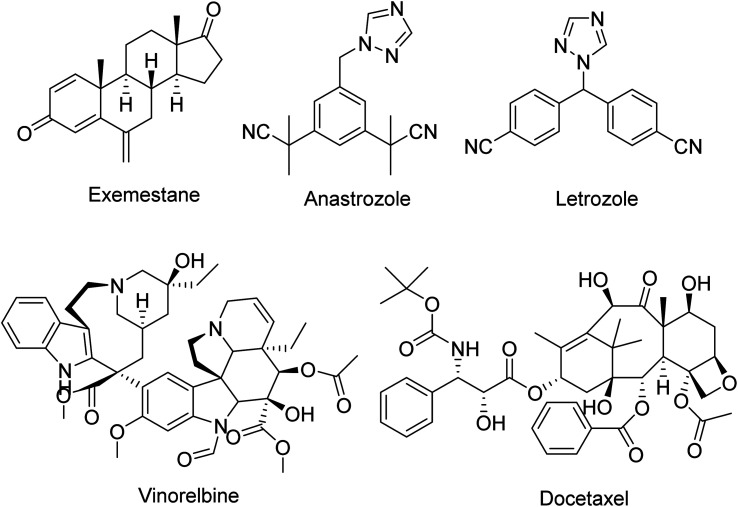
The chemical structures of clinically approved aromatase inhibitors (exemestane, anastrozole and letrozole), and microtubule-modulating agents (vinorelbine and docetaxel) for breast cancer treatment.

Estrogen receptor-positive (ER+) breast cancer is the most prevalent type of breast cancer, and is characterized by the predominant expression of estrogen and progesterone receptors, which drives cell proliferation in the breast.^3^ The chemotherapeutic approach to treat ER+ breast cancer is mainly by administration of hormonal drugs, *i.e.*, selective estrogen receptor modulators (SERMs), selective estrogen receptor degrader (SERD) and aromatase inhibitors to suppress estrogenic signals.^4^ Aromatase is a cytochrome P450 enzyme that contains a heme cofactor that is central to the catalytic conversion of androgens into estrogens.^3^ Aromatase activity is often upregulated in ER+ breast cancer tissues and has been designated as a key drug target for many clinically approved aromatase inhibitors: exemestane, letrozole and anastrozole (Fig. 1).^3^ Despite their clinical effectiveness, they are currently being challenged by drug resistance and undesirable side effects in patients, such as musculoskeletal pain, osteoporosis and cardiovascular problems.^5–8^ Other anticancer agents used to treat breast cancers include drugs that modulate microtubule activities such as vinorelbine and docetaxel (Fig. 1), which belong to the *Vinca* alkaloid and taxane classes of drugs, respectively.^9^ Microtubule-modulating agents exert their mechanism of action by binding to tubulin monomers that interfere with microtubule dynamics during mitosis, subsequently leads to mitotic arrest.^9^ Although clinically effective, they are limited by their large size, and are administered *via* injection and non-oral routes leading to inconvenience and compliancy issues for patients. Furthermore, cases of multidrug resistance were observed through various mechanisms, such as overexpression of drug efflux transporter MDR-1 (P-glycoprotein) resulting in reduced drug presence in cancer cells, and alteration of signal transduction pathways.^10^ Given these drawbacks, there is a need for the discovery and development of new anti-breast cancer agents with orally acceptable pharmacokinetic profiles.

It has been observed that co-administration of aromatase inhibitor and microtubule-modulating drugs can improve clinical effectiveness in breast cancer patients.^11,12^ Despite of these positive outcomes, such combinations can increase the risk of adverse effects, drug interactions, and detrimental physiological change. An alternative approach is to use dual-targeting strategies by employing dual-targeting agents, *i.e.*, agents that simultaneously interact with two different targets or pathways. This strategy has been applied broadly in medicinal chemistry with proven enhanced pharmacological potencies, clinical effectiveness and to overcome multidrug resistance mechanisms, *e.g.* antitubercular agents,^13,14^ anti-Alzheimer's agents,^15,16^ antimalarials,^17^ anti-rheumatics^18^ and anticancer agents.^19–23^ Regardless of the potential benefits, marginal development of agents that modulate aromatase and tubulin activities were reported such as the 1-(diarylmethyl)-1*H*-azoles series,^24^ whereas, dual modulators of tubulin and estrogen receptor activities have shown slightly more traction, *e.g.*, modulation of tubulin and antagonism of estrogen receptors.^25,26^

**Fig. 2:**
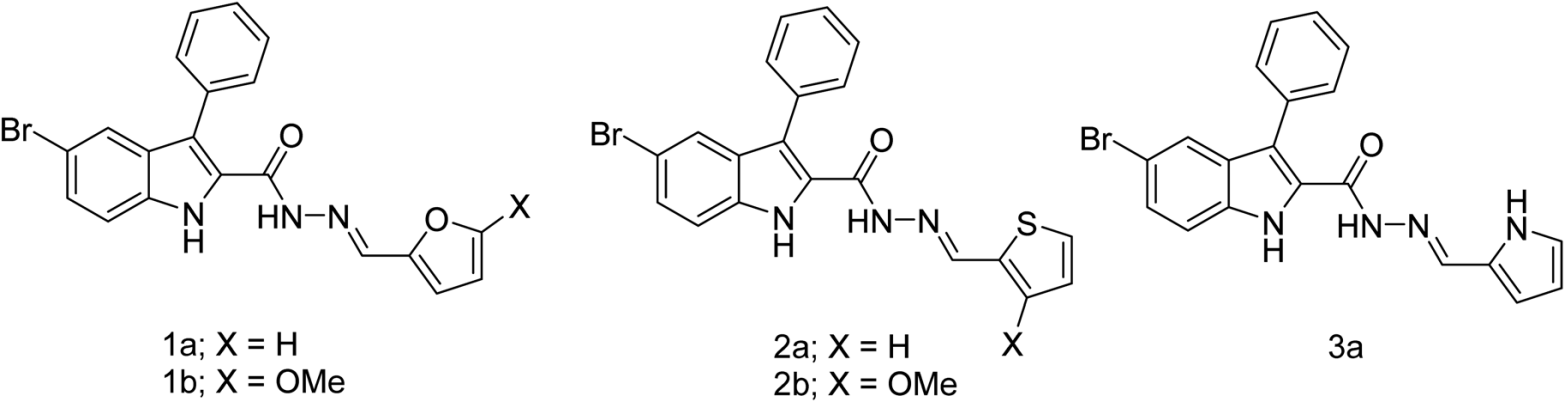
The chemical structures of compounds 1a–b, 2a–b, and 3a.

In our previous work, we have identified furanyl derivatives 1a, and thiophenyl derivatives 2a as promising anticancer agents, which subsequently led to the synthesis of 1b and 2b (Fig. 2) that exhibited potent anti-tubulin and anticancer activities.^27,28^ Typically, it was revealed that several derivatives from the thiophenyl subseries, including 2b, displayed potent antiproliferative activities and selectivity for certain cancer cell lines.^28^ The series is based on the 3-phenyl-1*H*-indole-2-carbohydrazide as the core scaffold. Consequently, this scaffold was used in this work as a platform for the design of new anticancer agents. Prior to this work, we were interested in attaching a pyrrole ring to our core scaffold, since pyrrole is a natural chelator of metals in many biological structures such as magnesium in chlorophyll, heme iron in hemoglobin and cytochrome P450 enzymes, including aromatase. Given this rationale, the pyrrole ring was attached to the side chain of the 3-phenyl-1*H*-indole-2-carbohydrazide core scaffold affording 3a (Fig. 2) with the expectation of a dual-targeting mechanism, *i.e.*, tubulin and aromatase inhibition. This was followed by establishing a preliminary structure–activity relationship (SAR) by the synthesis of twenty-four new derivatives with various substituents installed at the pyrrole ring followed by experimental validation of their anticancer, anti-tubulin, and anti-aromatase activities. Furthermore, molecular modeling was employed to gain an understanding of the interactions between the compounds and the protein targets, and their physicochemical properties were evaluated. It should be noted that previously, aryl-substituted derivatives were synthesised by Cai and co-workers,^29^ however these derivatives displayed only weak anticancer effects, Moreover, cycloalkyl derivatives were previously prepared by our groups, but they exhibited poor anticancer activities.^27^ The present work focuses on anticancer pyrrole–indole hybrids with dual tubulin and aromatase inhibitory activities.

## 2. Results and discussion

### 2.1. Chemistry

**Scheme 1:**
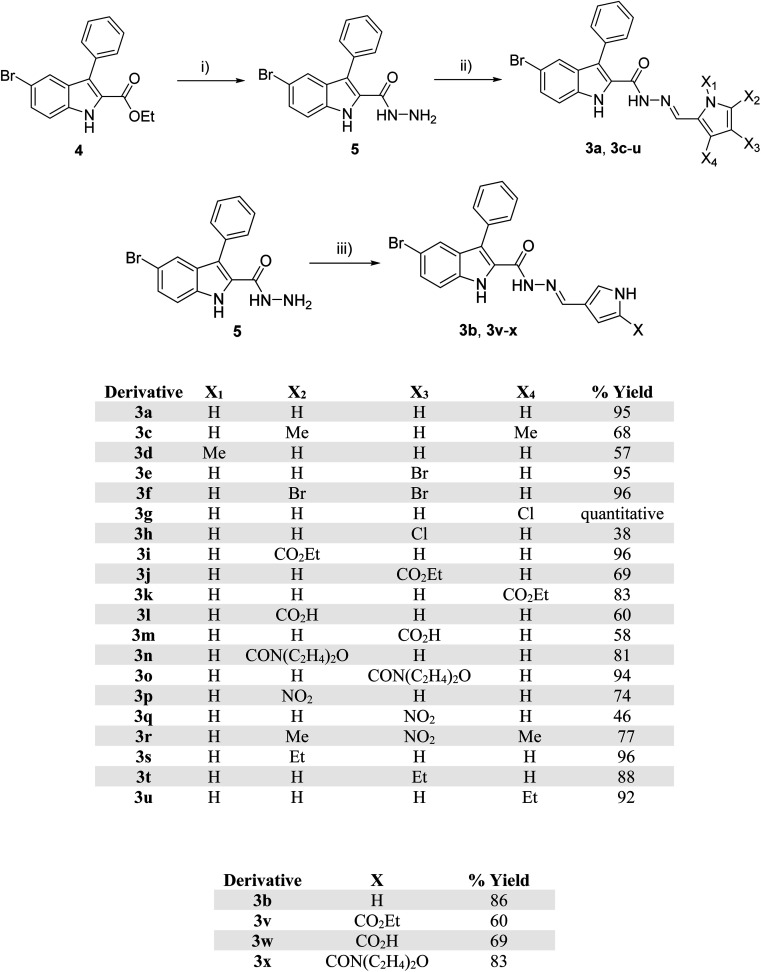
Synthesis of indole hydrazide derivatives; reagent and conditions: (i) N_2_H_4_, EtOH, 80 °C, 17 h, 70%; (ii) ArCHO, few drops AcOH, EtOH, 80 °C, 17 h, 46–100%; (iii) ArCHO, few drops AcOH, EtOH, 80 °C, 17 h, 67%.

The synthesis of pyrrolyl-3-phenyl-1*H*-indole-2-carbohydrazide 3a–x was achieved by the condensation of hydrazide 5 (ref. 28) with various pyrrole aldehyde (Scheme 1). The synthesis was initiated by replacing the ethoxy group of indole 4 (ref. 27) with hydrazine. Condensation of hydrazide 5 under acid condition with various substituted aldehydes afforded indole hydrazides. The indole hydrazide products from the condensation reaction could generate mixtures of *E*- and *Z*-isomers, but the *E*-isomer was isolated due to its high stability.^27,28^

The dimethyl substitutions at positions X_2_ and X_4_ on the pyrrole ring gave 3c, whereas *N*-methyl afforded 3d. The halogen-containing derivatives include bromo at X_3_ (3e), dibromo (3f) at X_2_ and X_3_, single chloro substitution at X_4_ (3g) and X_3_ (3h). The electron-withdrawing groups were introduced for ester substitution at X_2_ (3i), X_3_ (3j), and X_4_ (3k), carboxylic acid at X_2_ (3l) and X_3_ (3m), morpholine-4-carbonyl at positions X_2_ (3n), and X_3_ (3o), and nitro at positions X_2_ (3p) and position X_3_ (3q). Nitro substitution at X_3_ and dimethyl at X_2_ and X_4_ positions generated 3r. Weak electron-donating ethyl group was introduced at positions X_2_ (3s), X_3_ (3t), and X_4_ (3u). Altering the connectivity from 2-pyrrolyl to 3-pyrrolyl afforded 3b, and varying the functionality at position X produced ester (3v), carboxylic acid (3w), and amide (3x) derivatives. The purity of the compounds was assessed by ^1^H NMR spectroscopy, which showed the purity of *ca.* 93–97% of the final products. The ^1^H NMR spectra of some compounds have additional peaks due to the existence of rotamers arising from the amide bond rotation. To prove the existence of rotamers of these compounds, compounds 3d, 3i, and 3p were selected for the ^1^H NMR experiment at 90 °C (see ^1^H and ^13^C spectra in ESI†). Indeed, ^1^H NMR spectra of compounds 3d, 3i, and 3p at 90 °C showed only one set of a single isomer, confirming the presence of rotamers.

### 2.2. Biological evaluation

#### 2.2.1. Anticancer activities

In order to evaluate the anticancer effects of derivatives 3a–x, the derivatives were submitted to the NCI60 screening program.^30^ Firstly, the derivatives were screened at the 10 µM single dose against the NCI60 human cell line panel. The results of the single dose experiment are reported as the growth percentage against each cell line together with mean growth inhibition (NCI mean) are reported (Fig. S1†). Most of the derivatives displayed strong antiproliferative effects in the single dose experiment with NCI mean values ranging from −10.66 to 29.76. Candidates that displayed potent anticancer activities were selected for the five-dose experiment to derive the concentrations that inhibit cell growth by 50% (GI_50_) and kill cells by 50% (LC_50_) values; the complete data are shown in Table S2 and Fig. S3.†

The pyrrole derivatives are mostly strong anticancer agents (GI_50_ < 1 µM), with some exhibiting strong lethal effects (LC_50_ < 10 µM). The parent compound 3a displayed strong growth inhibition, and was most lethal against five cancer cell lines: HT29 colon adenocarcinoma (LC_50_ = 9.31 µM), UAAC-62 melanoma (LC_50_ = 8.03 µM), OVCAR-8 ovarian carcinoma (LC_50_ = 6.55 µM), SN12C renal cell carcinoma (LC_50_ = 3.97 µM), and BT-549 breast carcinoma (LC_50_ = 6.39). Derivative 3c exhibited moderate growth inhibition activities with GI_50_ values between 1.77 and 8.25 µM, and was most lethal against SK-MEL-5 melanoma cell line (LC_50_ = 19.3 µM). Derivative 3d displayed potent growth inhibition activities and was best at suppressing growth of MCF-7 breast cancer cells at double-digit nanomolar potency (GI_50_ = 72 nM), whilst exhibited selectivity for NCI/ADR-RES doxorubicin-resistant ovarian cancer cell line (LC_50_ = 40.9 µM) that overexpresses MDR1 drug efflux transporter.^31^ Single bromo-substituted derivative 3e displayed potent growth inhibition and exhibited most potent lethal effects against COLO 205 (LC_50_ = 0.89 µM), HT29 (LC_50_ = 8.24 µM), LOX IMVI (LC_50_ = 6.98 µM), SK-MEL-5 (LC_50_ = 1.22 µM), NCI/ADR-RES (LC_50_ = 0.97 µM), and SN12C (LC_50_ = 5.49 µM) cancer cell lines, whereas dibromo-substituted derivative 3f displayed moderate growth inhibition effects with GI_50_ values ranging between 0.53 to 6.8 µM, and was potent at killing SK-MEL-5 (LC_50_ = 8.65 µM) and NCI/ADR-RES (LC_50_ = 9.73 µM). Single chloro-substituted derivatives 3g and 3h were effective at suppressing growth inhibition, albeit 3g was less lethal. Derivative 3h displayed potent lethal effects against HL-60 (TB), K-562 and SR leukemia, NCI-H460 non-small cell lung carcinoma, COLO 205, HCC-2998 and HT29 colon carcinoma cell lines, SK-MEL-5 melanoma, and OVCAR-8 ovarian carcinoma with LC_50_ values in the range of 0.80 to 7.20 µM. Introduction of electron-withdrawing ester groups to derivatives 3i and 3j resulted in moderate growth inhibition activities. Nevertheless, 3i showed strong lethal effects against SK-MEL-5 (LC_50_ = 7.68 µM) and NCI/ADR-RES cell lines (LC_50_ = 9.2 µM), and 3j was potent against HL-60 (TB) (LC_50_ = 9.88 µM). Ester derivative 3k, and the carboxylic acid derivatives 3l and 3m were opted out from the five-dose experiment, *i.e.*, 3k displayed moderate growth inhibition (NCI mean = 49%), whereas 3l and 3m exhibited mild effects. Morpholine-4-carbonyl derivative 3n was potent at growth suppression and exhibited potent lethality against HL-60 (TB) with LC_50_ = 8.56 µM, whereas 3o showed moderate growth inhibition (NCI mean = 45%), and was subsequently excluded from the five-dose experiment. Single electron-withdrawing nitro-substituted derivatives 3p and 3q showed potent growth inhibition effects; 3p was most lethal against COLO 205 (LC_50_ = 8.08 µM) and SK-MEL-5 (LC_50_ = 8.69 µM), and 3q was most selective for U251 glioblastoma (LC_50_ = 66.8 µM), as well as MDA-MB-435 melanoma (LC_50_ = 8.78 µM) and SK-MEL-5 melanoma (LC_50_ = 69.9 µM). Derivative 3r exhibited moderate growth inhibition activities, but displayed lethal effects against NCI-H460, COLO 205, HT29, LOX IMVI, SK-MEL-5, NCI/ADR-RES and SN12C cell lines. Ethyl-substituted derivatives 3s, 3t and 3u exhibited strong growth inhibition activities with moderate lethal effects for 3s and 3u, whilst 3t displayed potent lethal effects for LOX IMVI and SK-MEL-5 melanoma cell lines, and MDA-MB-468 breast carcinoma. Altering the connectivity of the pyrrole ring resulted in apparent reduction in growth inhibition activities (3a*vs.*3b) with moderate lethal activities for most cell lines. The ester derivative 3v exhibited strong lethal effects for MDA-MB-435 melanoma with LC_50_ = 9.35 µM, and the substituted carboxylic acid (3w) and morpholine-4-carbonyl (3x) derivatives displayed moderate growth inhibition in the single dose experiment with NCI mean values ranging between 66–68%.

Six derivatives were the most potent: compound 3a, single electron-donating chloro- (3e) and bromo- (3h) substituted derivatives at X_2_ position, a single nitro-substituted derivative 3p at X_1_ position, and a dimethylated and nitro-substituted derivative 3r, and an ethylated derivative 3t at X_2_ position (Table 1). Small electron-donating and withdrawing substituents are favored for the anticancer effects, whereas derivatives with larger electron-withdrawing groups such as carbonyl-substituted derivatives are unfavorable. This can be attributed to an increase in molecular size and change in molecular shape which affects target binding. Additionally, an increase in molecular size and polarity perturbs drug-like behavior, *e.g.*, decrease passive diffusion of drugs into cancer cells. Comparing the NCI60 screening results from this work with our previous studies on the furanyl and thiophenyl derivatives, all three subseries possess strong efficacies for COLO 205 colon carcinoma, SK-MEL-5 and MDA-MB-435 melanoma, and OVCAR-8 ovarian carcinoma suggesting part of the mechanism of action is influenced by the presence of the same core scaffold.^28^ Interestingly, 3d displayed selectivity for the NCI/ADR-RES cell line, suggesting it as a highly promising candidate to treat multidrug-resistant ovarian cancers. In addition, 3q was revealed to be the most selective at killing MDA-MB-435 and SK-MEL-5 melanoma cell lines implying it as an appealing candidate for invasive forms of melanoma treatment. Moreover, 3q was identified to be relatively potent against U251 glioblastoma, which is highly associated with acquired drug resistance CNS cancer.^32^

**Table 1:** Dose–response for the most active derivatives shown for eight sensitive cancer cell lines in micro-molar (µM) concentrations

	3a		3e		3h		3p		3r		3t	
	GI_50_	LC_50_	GI_50_	LC_50_	GI_50_	LC_50_	GI_50_	LC_50_	GI_50_	LC_50_	GI_50_	LC_50_
HL-60 (TB)	0.242	>100	0.327	>100	0.185	0.886	0.374	>100	2.31	>100	0.237	91.9
COLO 205	0.261	35.1	0.192	0.884	0.251	1.88	1.52	8.08	1.9	7.51	0.207	—
LOX IMVI	0.244	19	0.36	6.98	0.37	16.7	0.519	35.8	1.63	5.73	0.427	8.27
MDA-MB-435	0.105	—	0.207	24.3	0.185	—	0.253	29.8	1.77	17.9	0.23	38.7
SK-MEL-5	0.302	15.7	0.195	1.22	0.228	3.08	1.02	8.69	1.5	5.87	0.279	6.96
OVCAR-8	0.323	6.55	0.304	18.2	0.214	0.988	1.02	39	3.08	36.5	0.316	26
NCI/(ADR-RES)	0.44	59.2	0.197	0.971	0.2	—	0.285	16.2	1.89	9.15	0.247	22
SN12C	0.371	3.97	0.310	5.49	0.391	15.3	1.24	36.7	1.74	6.73	0.372	30

#### 2.2.2. Tubulin polymerization inhibition

**Fig. 3:**
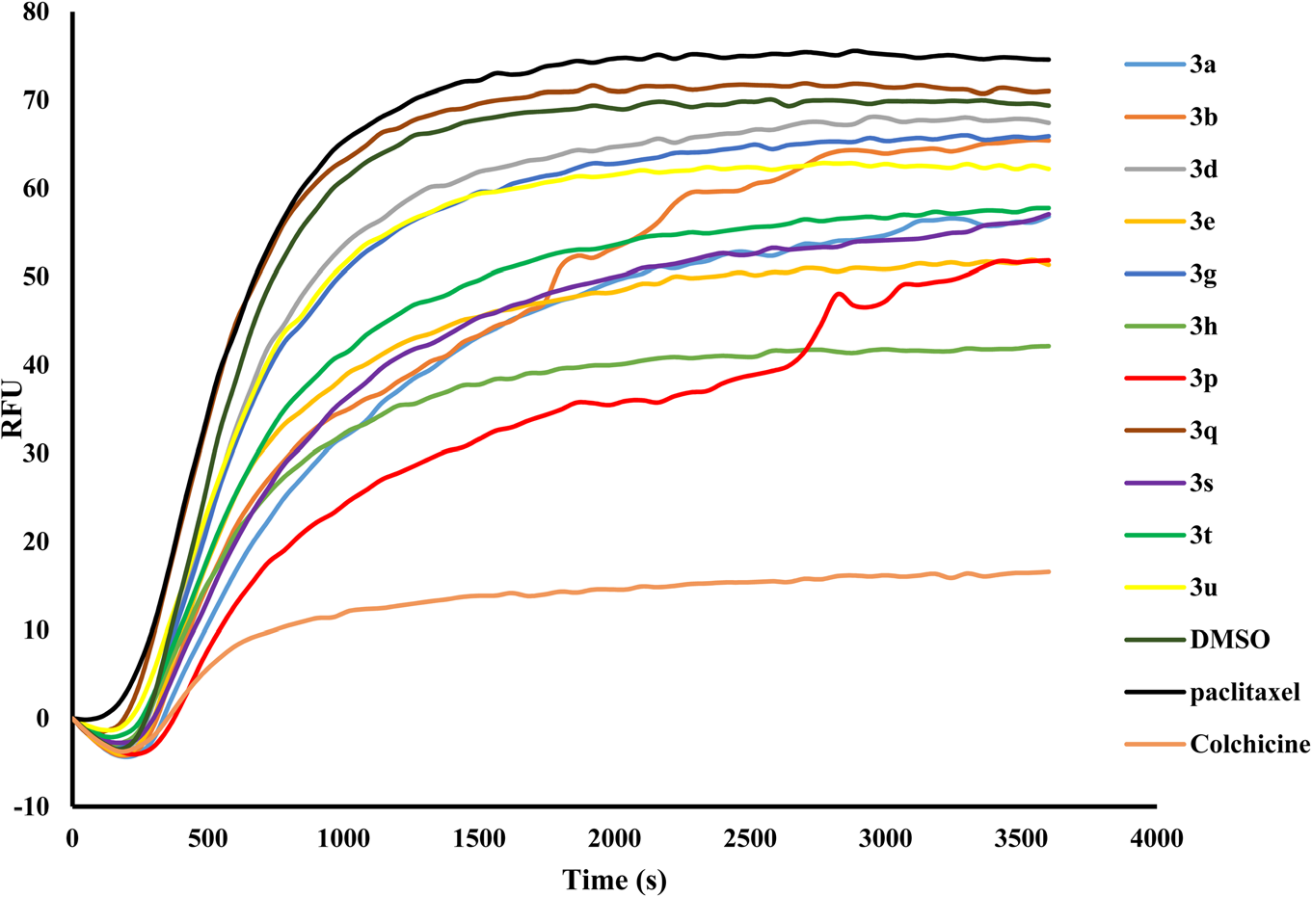
Effects of derivatives 3a–b, 3d–e, 3g–h, 3p–q, and 3s–u at a concentration of 10 µM on tubulin polymerization assay at 37 °C with colchicine (10 µM) as appositive control and paclitaxel (3 µM) as negative control. The curve is the average from two experiment runs.

The 3-phenyl-1*H*-indole-2-carbohydrazide core scaffold has been previously identified to have tubulin inhibition properties.^27–29^ To investigate the mechanism of action of the new derivatives, eleven derivatives were selected to be tested in a tubulin polymerization inhibition assay (3a–b, 3d–e, 3g–h, 3p–q, and 3s–u). Colchicine (10 µM) and paclitaxel (3 µM) was used as positive controls (Fig. 3). From the results, 3h was the most potent tubulin inhibitor with RFU ∼ 40 arbitrary units. Derivatives 3e and 3p were equally the second most potent inhibitors exhibiting tubulin inhibition at RFU ∼ 50 arbitrary units. This is followed by 3a, 3s and 3t with moderate inhibition activities at RFU ∼ 55 arbitrary units. Derivatives 3b, 3d, 3g and 3u were weak tubulin inhibitors, and 3q had no tubulin inhibition properties. The results showed poor correlations with the NCI60 data, suggesting that tubulin inhibition is not the primary mechanism of action.

#### 2.2.3. Cell cycle analysis

**Fig. 4:**
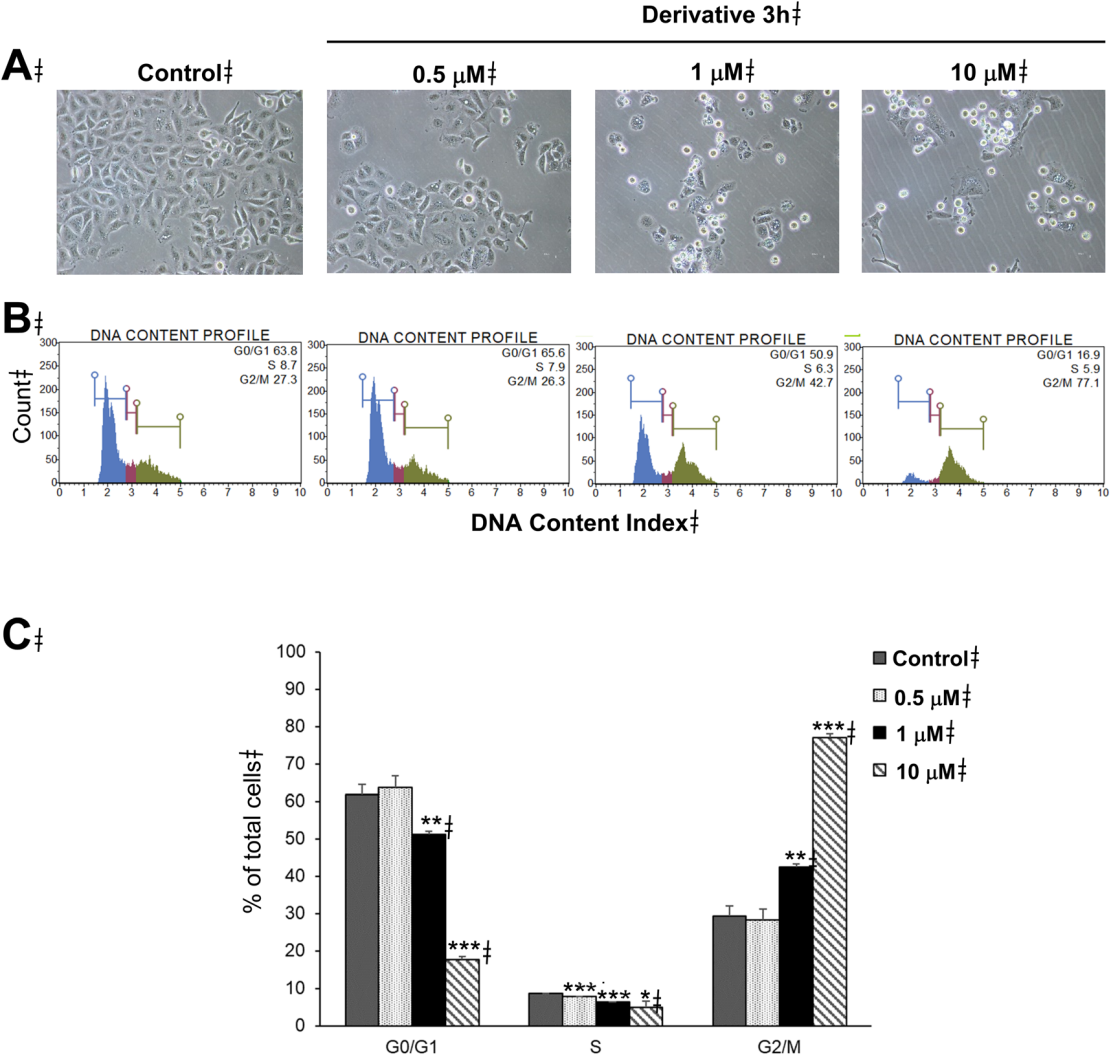
Effects of derivative 3h on cell morphology and cell cycle progression of A549 human non-small cell lung cancer cells. The cells were treated with 3h at indicated concentrations for 24 h. The cells were subjected to cell cycle analysis by flow cytometric method. (A) Cell morphology after treatment, images of a representative experiment are shown with original magnification of ×200. (B) DNA content histograms of a representative experiment. (C) Percentages of cell populations in G0/G1, S, and G2/M phases. Data are shown as average with SD from three independent experiments, *p < 0.05, **p < 0.01, and ***p < 0.001 *versus* control group.

Disruption of tubulin polymerization has been shown to cause the arrest of cell division at the G2/M phase of the cell cycle.^27^ This drew our curiosity to study the effects of the series on cell cycle progression, so we used the strongest tubulin inhibitor, 3h, to study the effects of cell cycle progression against the A549 non-small cell lung cancer cell line as a model. By treating with varying concentrations of 3h (0.5–10.0 µM) for 24 h, morphological changes were observed. There was a decrease in cell density, while the number of spherical cells increased in a dose-dependent fashion (Fig. 4A) indicating an increase of cell population in the mitotic phase. This suggested that 3h was able to induce cell cycle arrest.^33^ Results showed that 3h dose-dependently increased G2/M cell population to 42.4–77.1% of total cells, compared to 29.3% in the control group (Fig. 4B and C) indicating arrest at G2/M phase.

#### 2.2.4. Aromatase inhibitory activity

All derivatives were tested for aromatase inhibition (Table 2); active derivatives are defined as derivatives with IC_50_ < 12.5 µM. In Table 2, the pyrrolyl derivatives displayed potent aromatase inhibitory activities with IC_50_ values ranging between 18 nM to double digit micromolar values. However, aromatase inhibitory activities of the derivatives lacked correlation with anti-breast cancer activities suggesting that the series can act on multiple pathways. The strongest aromatase inhibitor was the ester derivative, 3k (IC_50_ = 18 nM), which has a strong electron-withdrawing ester group at position X_4_. Unfortunately, 3k has relatively moderate growth inhibition activities, and hence, the GI_50_ values of 3k were not determined by NCI. In this regard, the IC_50_ values were determined for the derivatives against the T47D breast cancer cell line based on our protocol. Initially, the derivatives were screened at two different concentrations, 10 µM and 50 µM respectively (Fig. S4†). Most of the derivatives displayed promising results from the two-dose experimental. However, alternate pyrrole (3b), derivatives with carboxylic acid substituents (3l–m and 3w), nitro group (3p–q), amide group (3x) showed weak activity. Promising candidates were evaluated for cytotoxic activity with T47D. The determined GI_50_ and IC_50_ values have relatively satisfactory correlations with *R*^2^ = 0.788 suggesting the results are relatively reliable (Fig. S5†). From this, it was revealed that 3k showed relatively moderate antiproliferative effects against T47D with IC_50_ = 10.6 µM. From Table 2, aromatase inhibition tends to be stronger with electron-withdrawing groups at positions X_3_ and X_4_ on the pyrrole ring for the 2-pyrrolyl derivatives, so only 3v (IC_50_ = 2.5 µM) and 3x (IC_50_ = 3.7 µM) in the 3-pyrrolyl derivatives were active. The trisubstituted derivative, 3r (IC_50_ = 0.2 µM) was the second best aromatase inhibitor, followed by 3f, 3j, 3h and 3o, which contain electron-withdrawing groups at X_3_ with IC_50_ values ranging between 1.3 to 1.8 µM, whereas 3a, 3g, 3i, 3n and 3v were slightly less potent with IC_50_ values ranging between 2.5 to 3.4 µM. Single bromo-substituted derivative 3e, and ethyl-substituted derivatives 3s and 3t exhibited weaker IC_50_ values between 4.0 to 4.7 µM, whilst 3c and 3u which contain electron-donating alkyl groups at X_4_ are weaker inhibitors with IC_50_ values at 11.1 and 9.7 µM, respectively. Despite the fact that *N*-methylation of pyrrole (3d) showed inactivity against aromatase, it exhibited the most potent antiproliferative effects for T47D (IC_50_ = 1.0 µM), comparable to 3a (IC_50_ = 1.6 µM). The carboxylated derivatives 3l, 3m, and 3w, and single nitro-substituted derivative 3q were inactive against aromatase. The unsubstituted furanyl- (1a) and thiophenyl- (2a) derivatives were also tested for aromatase inhibition. Interestingly, furanyl 1a was able to inhibit aromatase, albeit with weak potency (IC_50_ = 11.3 µM). However, thiophenyl 2a lacked aromatase inhibition.

**Table 2:** The aromatase inhibition, antiproliferative activities and *in vitro* toxicity data of derivatives 1a, 2a, and 3a–x, expressed as IC_50_ values (µM)

Derivative	Aromatase (µM)	T47D^a^ (µM)	L929^a^ (µM)	Selectivity index^d^ (SI)
1a	11.3 ± 1.7	—b	—b	—
2a	>12.5	—b	—b	—
3a	3.4 ± 0.6	1.6 ± 1.0	26.3 ± 14.5	16.4
3b	11.0 ± 1.3	—b	—b	—
3c	11.1 ± 2.0	11.6 ± 0.3	29.5 ± 12.6	2.54
3d	>12.5	1.0 ± 0.4	—c	—
3e	4.0 ± 1.0	2.4 ± 1.4	22.3 ± 11.9	9.29
3f	1.5 ± 0.3	19.1 ± 5.4	47.9 ± 0.5	2.51
3g	2.6 ± 0.5	8.2 ± 1.8	20.1 ± 10.3	2.45
3h	1.8 ± 0.4	2.4 ± 1.8	53.7 ± 30.4	22.4
3i	2.6 ± 0.8	11.8 ± 1.4	—c	—
3j	1.3 ± 0.4	10.4 ± 2.9	12.2 ± 1.0	1.17
3k	0.018 ± 0.002	10.6 ± 1.1	15.0 ± 8.9	1.42
3l	>12.5	—b	—b	—
3m	>12.5	—b	—b	—
3n	3.3 ± 0.6	5.4 ± 4.8	21.8 ± 11.5	4.04
3o	1.6 ± 0.4	14.9 ± 1.0	43.2 ± 9.9	3.57
3p	1.4 ± 0.3	—b	—b	—
3q	>12.5	—b	—b	—
3r	0.2 ± 0.1	12.3 ± 0.3	—c	—
3s	4.6 ± 0.5	3.8 ± 1.0	19.5 ± 11.8	5.13
3t	4.7 ± 0.5	4.6 ± 2.9	7.1 ± 3.6	1.54
3u	9.6 ± 1.0	6.2 ± 3.6	12.2 ± 0.7	1.97
3v	2.5 ± 0.6	24.9 ± 8.4	90.1 ± 40.3	3.62
3w	>12.5	—b	—b	—
3x	3.7 ± 0.6	—b	—b	—
Letrozole	0.0014 ± 0.0003	—b	—b	—
Cisplatin	—b	>100	23.7 ± 8.9	—

a IC_50_ values determined by protocol in Methodology section 4.6.

b Not determined.

c No toxicity observed at 50 µM single concentration (>80% cell survival).

d = IC_50_ (L929)/IC_50_ (T47D).

#### 2.2.5. *In vitro* toxicity in non-tumoral cell line

To determine the toxic effects of the derivatives in a non-tumour cell line, the cytotoxic effects of the derivatives were tested against the L929 normal mouse fibroblast cell line and compared to the T47D cell line (Table 2). Derivatives 3d, 3i and 3r were determined to have no toxicity towards L929 cells at 50 µM single concentration, *i.e.*, >80% cell survival. The IC_50_ values were determined for other derivatives with <80% L929 cell survival tested at 50 µM single concentration. Interestingly, the highly potent single halogen-substituted derivatives 3e and 3h were some of the least toxic derivatives with SI ∼ 22 for 3h and SI ∼ 9 for 3e, followed by the non-substituted parent compound, 3a (SI ∼ 16). Derivatives 3s, 3n, 3v and 3o, had SI values in the range of 3.5 and 5.1. Derivatives 3c, 3f, 3g and 3u displayed approximately two-folds less toxicity against normal cell line L929 than in T47D cancer cells, whilst 3j, 3k and 3t exhibited relatively similar toxicity profiles in both cell lines.

### 2.3. Molecular modelling

#### 2.3.1. Molecular docking analysis of derivatives to tubulin

**Fig. 5:**
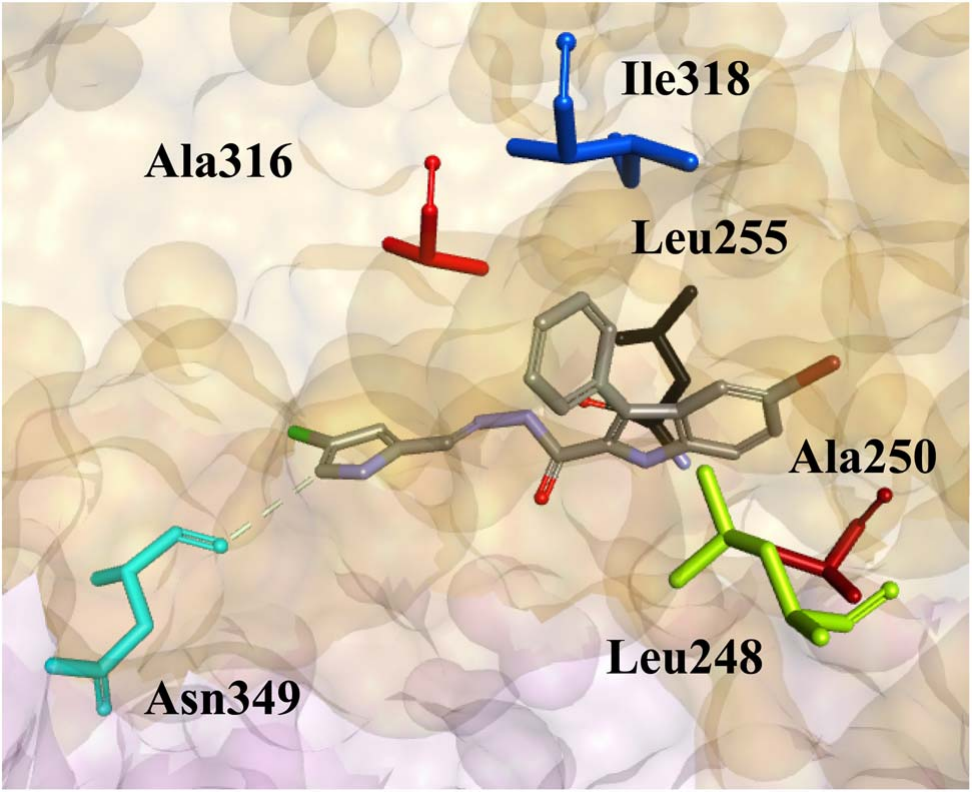
The predicted binding orientation of derivative 3h at the colchicine binding site. The dipole–dipole interactions between Asn349 and the ligands are shown. The surface of tubulin was rendered orange for the β-subunit and light pink for the α-subunit. Amino acid residues Ala316, Ile318, Leu255, Ala250, Leu248 and Asn349 are depicted as sticks and colored as red, blue, black, brown, lime green and turquoise, respectively.

From the literature, it is known that compounds with the 3-phenyl-1*H*-indole-2-carbohydrazide core scaffold interact most favorably with the colchicine binding site located at the α- and β-subunit interface leading to destabilization of the microtubule structure.^27–29^ Thus, we planned to dock the pyrrolyl derivatives into the colchicine site of tubulin to study their mechanism of binding. However, before this was performed, the docking protocol was first validated by docking the co-crystallized structure of colchicine to the colchicine binding site of tubulin in accordance with the protocol (see Experimental section 4.7). The docked conformation of colchicine was overlaid on to the co-crystallized structure (Fig. S6†) and the root-mean square deviation (RMSD) was calculated to be 1.33 Å using the DockRMSD^34^ web server as previous described^28^ suggesting the reproducibility of the results and reliability of the docking procedures, *i.e.*, RMSD < 2 Å.^35^ Following this, the derivatives were docked to the colchicine binding site of tubulin. The binding mode of the pyrrolyl series can be exemplified by the most active derivative 3h as shown in Fig. 5. The pyrrolyl derivatives were predicted to have a different binding orientation to the furanyl and thiophenyl derivatives, which can explain their weaker tubulin inhibitory activities. Interactions between the pyrrolyl derivatives and the colchicine site lacked hydrogen bonding and tended to rely mainly on hydrophobic and dipole–dipole interactions. Orientation of the pyrrolyl side chain tend to reside with the β-subunit of tubulin with dipole–dipole interactions seen with Asn249. The 3-phenyl-1*H*-indole-2-carbohydrazide core scaffold is predicted to form hydrophobic interactions with vicinal hydrophobic residues Leu248, Ala250, Leu255, Ala316 and Ile318 in the hydrophobic region of the colchicine site.

#### 2.3.2. Molecular docking analysis of derivatives to aromatase

**Fig. 6:**
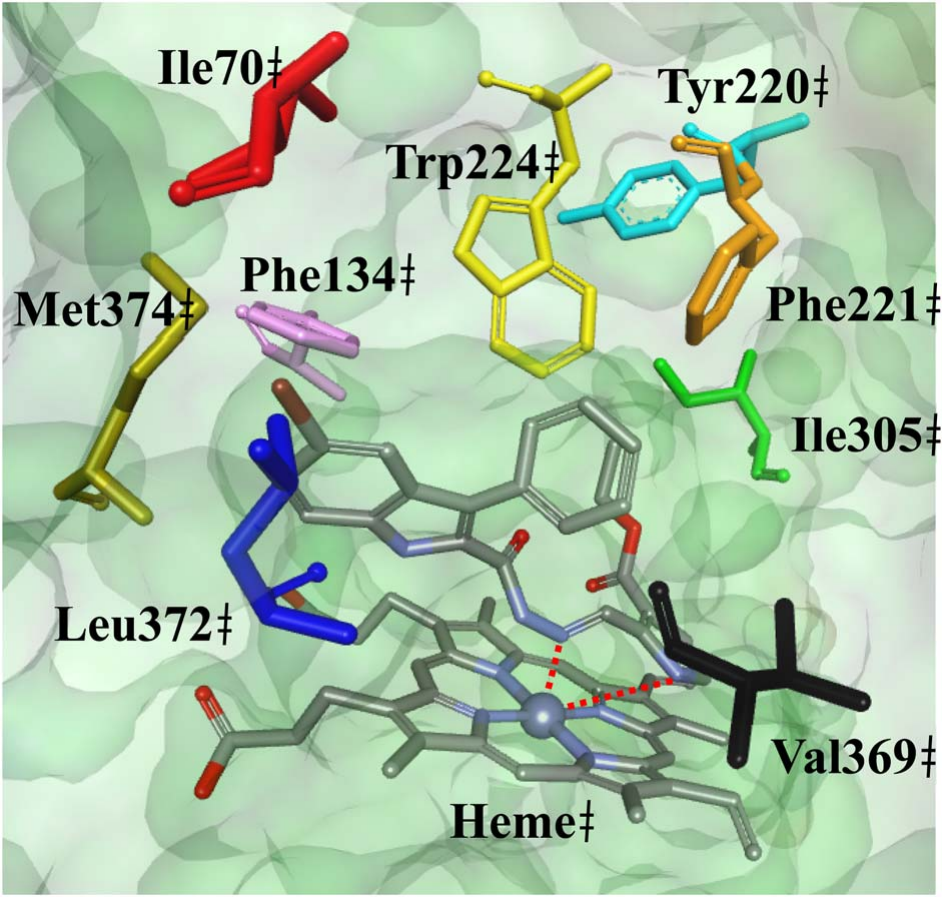
The predicted binding orientation of derivative 3k in the aromatase binding site. Potential chelation between 3k and heme (blue spherical shape) is depicted as red dotted lines. The surface of the aromatase was rendered in green. Amino acid residues Ile70, Phe134, Tyr220, Phe221, Trp224, Val369, Ile305, Leu372, and Met374 are depicted in sticks and colored as red, turquoise, orange, yellow, black, green, blue, and olive green, respectively.

To elucidate the mechanism of the binding of pyrrolyl derivatives to aromatase, the derivatives were docked to the binding site of aromatase. The docking protocol was validated by docking the co-crystallized structure of exemestane to the aromatase binding site in accordance to the protocol in the Experimental section 4.7. The docked conformation of exemestane was overlaid to the co-crystallized structure (Fig. S7†) and the root-mean squared deviation (RMSD) was calculated to be 0.46 Å using the DockRMSD^34^ web server, which suggest the reliability of the docking protocol and reproducibility of the docking results.^35^ The binding modes of the pyrrolyl derivatives are represented by the most active derivative 3k (Fig. 6). It can be seen that the hydrazone functionality and the pyrrole ring is oriented towards the heme group, and is predicted to chelate with heme iron, which can explain the lack of inhibition by non-chelating entities, *e.g.*, thiophenyl 2a and *N*-methylated pyrrole 3d. However, furanyl 1a exhibited inhibition of aromatase suggesting the hydrazone potentially holds a fair share of influence over heme chelation for some derivatives. Inhibition of the series to aromatase is predicted to be accommodated mainly by hydrophobic interactions with surrounding hydrophobic residues, with the phenyl group is oriented to nearby Trp224, Tyr220, Phen221 and Ile305, the indole scaffold is oriented to form interactions with Phe134, Ile70, Met374 and Leu372, whereas the pyrrole ring is predicted to interact with Val369.

#### 2.3.3. Chemical space

The physicochemical properties of the new derivatives were predicted using the Dragon 7.0 software. The software calculates for the molecular descriptors that are generally used to assess the pharmacokinetic profiles of drugs: molecular weight (MW), hydrogen bond donors (HD) and acceptors (HA), rotatable bonds (RB), Moriguchi log P (*M*logP)^36^ and polar surface area (PSA). The results are shown in Table S8.† Results revealed that most synthesized derivatives are within the Lipinski's^37^ and Veber's^38^ limits, *i.e.*, most derivatives are predicted to be adequate for oral drug administration. Nonetheless, derivatives with dibromo (3f) and morpholine-4-carbonyl (3n, 3o and 3x) substitutions are large in size resulting in the molecular weights exceeding the 500 g mol^−1^ Lipinski's limit.

The toxicity profiles of the most active compounds were predicted using ADMETlab 3.0.^39^ The results of the mainstream toxicity types for the compounds are shown in Table S9.† The compounds were predicted to have low cardiotoxic and immunotoxic effects with moderate chances of being mutagens, albeit they are generally well-tolerated. Nonetheless, strong heptotoxic effects were predicted, which indicate further need for hepatotoxicity studies.

## 3. Conclusion

In this study, twenty-four new pyrrolyl-3-phenyl-1*H*-indole-2-carbohydrazides were successfully designed, synthesized and evaluated for their dual inhibitory effects against two targets: tubulin and aromatase. Derivative 3h which has a single chloro substitution at X_3_ was the strongest tubulin inhibitor with strong aromatase inhibitory activity. Furthermore, strong lethal effects and growth inhibition activities were observed for 3h, which was confirmed to cause mitotic arrest at the G2/M phase. Ester derivative 3k was the best aromatase inhibitor but exhibited moderate antiproliferative effects. In general, electron-withdrawing substituents tended to favor aromatase inhibition. However, the series lacked correlation between aromatase inhibition and anti-T47D breast cancer activities. All derivatives displayed lower cytotoxic effects against the non-tumoral L929 cell line compared to the T47D cancer cell line. Molecular docking predicted the subseries interact with and inhibit tubulin at the colchicine site and revealed that in addition to the pyrrole as a chelating group, the hydrazone functionality is predicted to contribute to heme chelation for some derivatives resulting in the subsequent inhibition of aromatase. Eight cancer cell lines were particularly sensitive to the subseries: HL-60 (TB), COLO 205, LOX IMVI, MDA-MB-435, SK-MEL-5, OVCAR-8, NCI/(ADR-RES) and SN12C. Strong antiproliferative effects against COLO 205, MDA-MB-435, SK-MEL-5 and OVCAR-8 cancer cell lines are shared with precedent furanyl and thiophenyl subseries. The derivatives were mostly predicted to be drug-like with acceptable oral pharmacokinetics. The most active compounds were generally predicted to have adequate toxicity profiles, aside from speculation of strong hepatotoxic effects.

## 4. Experimental section

### 4.1. Synthesis

All chemicals for synthesis were purchased from commercial suppliers and used directly unless specifically stated. Thin layer chromatography was performed on Merck silica gel 60 F254 plates and visualized by UV irradiation at 254 nm. Flash column chromatography was performed using SilicaFlash® F60 (40–63 µm) or (63–200 µm) to obtain purified products. Automated flash chromatography was performed on Ultra Performance Flash Purification Chromatography (Model: Interchim puriflash 5.050) to obtain purified products. Bruker AVANCE-400 and 600 MHz were used to collect ^1^H and ^13^C NMR spectra. Chemical shifts (*δ*) are reported in parts-per-million (ppm) with respect to the solvent peaks and coupling constants (*J*) are in Hertz. Multiplicity of NMR peaks are given the abbreviations: s = singlet, d = doublet, t = triplet, q = quartet, m = multiplet, br = broad. Thermo Scientific Nicolet iS 5 Fourier Transform IR spectrometer was used to record Infrared (IR) spectra. Only selected peaks are reported and absorption maxima are given in cm^−1^. Thermo Scientific Orbitrap Q Exactive Focus mass spectrometer was used to record High Resolution mass spectra (HR-MS). Melting points were measured by using a Stuart SMP30 capillary melting point apparatus.

#### 4.1.1. 5-Bromo-3-phenyl-1*H*-indole-2-carbohydrazide (5)

To a solution of indole 5 (0.864 g, 2.59 mmol) in EtOH (30 mL), was added hydrazine (50% in H_2_O, 9.60 mL, 154.11 mmol). The resulting mixture was cooled to room temperature, and the solvent was evaporated. Solid product was recrystallized from EtOH giving 5-bromo-3-phenyl-1*H*-indole-2-carbohydrazide 5 (0.610 g, 1.85 mmol, 71%) as a pale yellow solid; mp 240–242 °C, lit.^25^ mp for 5 242–244 °C; ^1^H NMR (400 MHz, (CD_3_)_2_SO) *δ* 11.94 (s, 1H), 8.89 (s, 1H), 7.62 (d, *J* = 1.8 Hz, 1H), 7.49–7.35 (m, 7H), 4.49 (s, 1H). The data were consistent with that in the literature.^25^

#### 4.1.2. (*E*)-*N*′-((1*H*-Pyrrol-2-yl)methylene)-5-bromo-3-phenyl-1*H*-indole-2-carbohydrazide (3a)

To a solution of hydrazide 2 (0.071 g, 0.22 mmol) in EtOH (10 mL) was added pyrrole-2-carboxaldehyde (0.021 g, 0.22 mmol) and a few drop of acetic acid. The resulting mixture was heated to 80 °C, and stirred for 17 h. The reaction mixture was allowed to cool to room temperature, and solvent was evaporated. Purification by flash column chromatography, eluted with EtOAc–hexane (1 : 9 to 1 : 1), gave indole hydrazide 3a (0.085 g, 0.21 mmol, 95%) as a yellow solid; *R*_f_ 0.5 [EtOAc–hexane (1 : 1)]; mp 206 °C decomposed; ^1^H NMR (600 MHz (CD_3_)_2_SO) *δ* 12.14 (s, 1H), 11.51 (s, 1H), 11.07 (s, 1H), 7.87 (s, 1H), 7.71 (s, 1H), 7.50–7.44 (m, 5H), 7.38 (dd, *J* = 8.6, 1.9 Hz, 1H), 7.34 (t, *J* = 7.5 Hz, 1H), 6.89 (s, 1H), 6.42 (s, 1H), 6.10 (s, 1H); ^13^C NMR (150 MHz (CD_3_)_2_SO) *δ* 157.66, 140.54, 134.29, 133.10, 129.53, 129.28, 128.58, 127.88, 126.85, 126.75, 126.34, 122.78, 121.83, 116.76, 114.47, 113.62, 112.91, 109.40; *ν*_max_/cm^−1^ 3254, 1629, 1599, 1290; HRMS (ESI) *m*/*z* [M + H]^+^ calcd for C_20_H_16_ON_4_^79^Br 407.0502; found 407.0504. Compounds 3e, 3g, 3f and 3h were synthesized following the procedure described here.

#### 4.1.3. (*E*)-5-Bromo-*N*′-((4-bromo-1*H*-pyrrol-2-yl)methylene)-3-phenyl-1*H*-indole-2-carbohydrazide (3e)

Yellow solid (0.103 g, 0.21 mmol, 95%); *R*_f_ 0.6 [EtOAc–hexane (1 : 1)]; mp 231–233 °C decomposed; ^1^H NMR (600 MHz (CD_3_)_2_SO) *δ* 12.05 (s, 1H), 11.76 (s, 1H), 11.15 (s, 1H), 7.76 (s, 1H), 7.62 (s, 1H), 7.41–7.35 (m, 5H), 7.30–7.23 (m, 2H), 6.91 (s, 1H), 6.44 (s, 1H); ^13^C NMR (150 MHz (CD_3_)_2_SO) *δ* 157.98, 139.39, 134.40, 133.07, 129.62, 129.01, 128.66, 127.94, 127.87, 126.98, 126.55, 122.31, 121.95, 117.19, 114.58, 114.35, 113.04, 96.05; *ν*_max_/cm^−1^ 3305, 1645, 1615, 1538, 1288; HRMS (ESI) *m*/*z* [M + H]^+^ calcd for C_20_H_15_ON_4_^79^Br_2_ 484.9607; found 484.9604.

#### 4.1.4. (*E*)-5-Bromo-*N*′-((4,5-dibromo-1*H*-pyrrol-2-yl)methylene)-3-phenyl-1*H*-indole-2-carbohydrazide (3f)

Yellow solid (0.145 g, 0.26 mmol, 96%); *R*_f_ 0.5 [EtOAc–hexane (2 : 3)]; mp 164 °C decomposed; ^1^H NMR (600 MHz (CD_3_)_2_SO) *δ* 12.64 (s, 1H), 12.08 (s, 1H), 11.23 (s, 1H), 7.73 (s, 1H), 7.64 (s, 1H), 7.42–7.26 (m, 7H), 6.56 (s, 1H); ^13^C NMR (150 MHz (CD_3_)_2_SO) *δ* 158.03, 138.69, 134.39, 133.03, 129.60, 129.13, 128.89, 128.63, 127.91, 126.97, 126.57, 121.95, 117.28, 115.11, 114.57, 113.04, 105.49, 99.00; *ν*_max_/cm^−1^ 3259, 3066, 1646, 1536, 1244; HRMS (ESI) *m*/*z* [M + H]^+^ calcd for C_20_H_14_ON_4_^79^Br_3_ 562.8712; found 562.8714.

#### 4.1.5. (*E*)-5-Bromo-*N*′-((3-chloro-1*H*-pyrrol-2-yl)methylene)-3-phenyl-1*H*-indole-2-carbohydrazide (3g)

Yellow solid (0.105 g, 0.24 mmol, quantitative); *R*_f_ 0.7 [EtOAc–hexane (1 : 1)]; mp 170 °C decomposed; ^1^H NMR (400 MHz (CD_3_)_2_SO) *δ* 12.34 (s, 1H), 12.17 (s, 1H), 11.18 (s, 1H), 7.84 (s, 1H), 7.74 (s, 1H), 7.54–7.36 (m, 7H), 6.47 (s, 1H), 6.11 (s, 1H); ^13^C NMR (100 MHz (CD_3_)_2_SO) *δ* 157.83, 139.78, 134.34, 133.07, 129.56, 129.11, 128.60, 127.91, 126.91, 126.77, 126.44, 121.88, 118.52, 116.99, 114.53, 114.18, 112.96, 107.96; *ν*_max_/cm^−1^ 3241, 3080, 1645, 1538, 1246; HRMS (ESI) *m*/*z* [M + H]^+^ calcd for C_20_H_15_ON_4_^79^Br^35^Cl 441.0112; found 441.0112.

#### 4.1.6. (*E*)-5-Bromo-*N*′-((4-chloro-1*H*-pyrrol-2-yl)methylene)-3-phenyl-1*H*-indole-2-carbohydrazide (3h)

Yellow solid (0.039 g, 0.09 mmol, 38%); *R*_f_ 0.6 [EtOAc–hexane (1 : 1)]; mp 190 °C decomposed; ^1^H NMR (400 MHz (CD_3_)_2_SO) *δ* 12.14 (s, 1H), 11.78 (s, 1H), 11.22 (s, 1H), 7.84 (s, 1H), 7.71 (s, 1H), 7.51–7.33 (m, 7H), 6.97 (s, 1H), 6.48 (s, 1H); ^13^C NMR (100 MHz (CD_3_)_2_SO) *δ* 157.81, 139.51, 134.30, 133.02, 129.52, 129.02, 128.55, 127.84, 126.86, 126.41, 121.85, 119.76, 117.01, 114.49, 112.92, 111.73, 111.57; *ν*_max_/cm^−1^ 3254, 1613, 1537, 1237; HRMS (ESI) *m*/*z* [M + H]^+^ calcd for C_20_H_15_ON_4_^79^Br^35^Cl 441.0112; found 441.0113.

#### 4.1.7. (*E*)-*N*′-((1*H*-Pyrrol-3-yl)methylene)-5-bromo-3-phenyl-1*H*-indole-2-carbohydrazide (3b)

To a solution of hydrazide 2 (0.091 g, 0.28 mmol) in EtOH (10 mL) was added pyrrole-3-carboxaldehyde (0.027 g, 0.28 mmol) and a few drops of acetic acid. The resulting mixture was heated to 80 °C, and stirred for 17 h. The reaction mixture was allowed to cool to room temperature, and solvent was evaporated. Purification by automated flash chromatography, eluted with EtOAc–hexane (0 : 100 to 100 : 0), gave indole hydrazide 3b (0.096 g, 0.24 mmol, 86%) as a brown solid; *R*_f_ 0.5 [EtOAc–hexane (100 : 0)]; mp 177–180 °C decomposed; ^1^H NMR (600 MHz (CD_3_)_2_SO) *δ* 12.09 (s, 1H), 11.15 (s, 1H), 10.88 (s, 1H), 7.90 (s, 1H), 7.69 (s, 1H), 7.50–7.44 (m, 5H), 7.37–7.33 (m, 2H), 7.15 (s, 1H), 6.80 (s, 1H), 6.36 (s, 1H); ^13^C NMR (150 MHz (CD_3_)_2_SO) *δ* 157.48, 144.53, 134.22, 133.15, 129.57, 129.42, 128.57, 127.96, 126.83, 126.25, 122.01, 121.78, 120.10, 119.01, 116.61, 114.44, 112.85, 105.30; *ν*_max_/cm^−1^ 3239, 3049, 1629, 1557, 1288; HRMS (ESI) *m*/*z* [M + H]^+^ calcd for C_20_H_16_ON_4_^79^Br 407.0502; found 407.0495. Compounds 3k, 3o (eluted with MeOH–CH_2_Cl_2_ (0 : 100 to 15 : 85)), and 3s–u were synthesized following the procedure described here.

#### 4.1.8. Ethyl (*E*)-2-((2-(5-bromo-3-phenyl-1*H*-indole-2-carbonyl)hydrazono)methyl)-1*H*-pyrrole-3-carboxylate (3k)

Yellow solid (0.097 g, 0.20 mmol, 83%); *R*_f_ 0.4 [EtOAc–hexane (1 : 1)]; mp 259–261 °C decomposed; ^1^H NMR (600 MHz (CD_3_)_2_SO) *δ* 12.16 (s, 1H), 12.04 (s, 1H), 11.54 (s, 1H), 8.59 (s, 1H), 7.72 (s, 1H), 7.51–7.33 (m, 7H), 6.86 (s, 1H), 6.47 (s, 1H), 4.18 (q, *J* = 7.1 Hz, 2H), 1.24 (t, *J* = 7.1 Hz, 3H); ^13^C NMR (150 MHz (CD_3_)_2_SO) *δ* 163.79, 158.06, 139.50, 134.35, 133.07, 129.94, 129.56, 128.86, 128.56, 127.87, 126.88, 126.51, 122.15, 121.91, 117.33, 116.43, 114.53, 112.97, 111.09, 59.51, 14.35; *ν*_max_/cm^−1^ 3262, 3061, 2853, 1698, 1640, 1538, 1268; HRMS (ESI) *m*/*z* [M + H]^+^ calcd for C_23_H_20_O_3_N_4_^79^Br 479.0713; found 479.0712.

#### 4.1.9. (*E*)-5-Bromo-*N*′-((4-(morpholine-4-carbonyl)-1*H*-pyrrol-2-yl)methylene)-3-phenyl-1*H*-indole-2-carbohydrazide (3o)

Yellow solid (0.09 g, 0.17 mmol, 94%); *R*_f_ 0.5 [MeOH–CH_2_Cl_2_ (7 : 93)]; mp 198–200 °C; ^1^H NMR (600 MHz (CD_3_)_2_SO) *δ* 12.15 (s, 1H), 11.94 (s, 1H), 11.23 (s, 1H), 7.90 (s, 1H), 7.71 (s, 1H), 7.51–7.44 (m, 5H), 7.38 (dd, *J* = 8.6, 1.9 Hz, 1H), 7.34 (t, *J* = 7.4 Hz, 1H), 7.20 (s, 1H), 6.64 (s, 1H), 3.57 (s, 8H); ^13^C NMR (150 MHz (CD_3_)_2_SO) *δ* 164.66, 157.82, 139.89, 134.33, 133.04, 129.53, 129.07, 128.58, 127.86, 127.00, 126.88, 126.43, 125.09, 121.87, 119.01, 116.98, 114.50, 113.61, 112.95, 66.27; *ν*_max_/cm^−1^ 3213, 2856, 1601, 1544, 1225; HRMS (ESI) *m*/*z* [M + H]^+^ calcd for C_25_H_23_O_3_N_5_^79^Br 520.0979; found 520.0977.

#### 4.1.10. (*E*)-5-Bromo-*N*′-((5-ethyl-1*H*-pyrrol-2-yl)methylene)-3-phenyl-1*H*-indole-2-carbohydrazide (3s)

Yellow solid (0.110 g, 0.25 mmol, 96%); *R*_f_ 0.5 [EtOAc–hexane (1 : 1)]; mp 136 °C decomposed; ^1^H NMR (600 MHz (CD_3_)_2_SO) *δ* 12.08 (s, 1H), 11.23 (s, 1H), 10.93 (s, 1H), 7.72 (s, 1H), 7.66 (s, 1H), 7.45–7.39 (m, 5H), 7.33 (dd, *J* = 8.7, 1.9 Hz, 1H), 7.29 (t, *J* = 7.4 Hz, 1H), 6.25 (s, 1H), 5.79 (s, 1H), 2.52 (q, *J* = 7.6 Hz, 2H), 1.10 (t, *J* = 7.6 Hz, 3H); ^13^C NMR (150 MHz (CD_3_)_2_SO) *δ* 157.59, 140.63, 139.96, 134.32, 133.16, 129.55, 129.42, 128.62, 127.94, 126.89, 126.33, 125.49, 121.84, 116.67, 114.66, 114.49, 112.94, 106.43, 20.41, 13.96; *ν*_max_/cm^−1^ 3313, 2925, 1612, 1544, 1247; HRMS (ESI) *m*/*z* [M + H]^+^ calcd for C_22_H_20_ON_4_^79^Br 435.0815; found 435.0812.

#### 4.1.11. (*E*)-5-Bromo-*N*′-((4-ethyl-1*H*-pyrrol-2-yl)methylene)-3-phenyl-1*H*-indole-2-carbohydrazide (3t)

Yellow solid (0.095 g, 0.22 mmol, 88%); *R*_f_ 0.5 [EtOAc–hexane (1 : 1)]; mp 141 °C decomposed; ^1^H NMR (600 MHz (CD_3_)_2_SO) *δ* 12.08 (s, 1H), 11.13 (s, 1H), 10.98 (s, 1H), 7.75 (s, 1H), 7.66 (s, 1H), 7.45–7.38 (m, 5H), 7.32 (dd, *J* = 8.6, 1.9 Hz, 1H), 7.29 (t, *J* = 7.4 Hz, 1H), 6.63 (s, 1H), 6.24 (s, 1H), 2.34 (q, *J* = 7.6 Hz, 2H), 1.05 (t, *J* = 7.5 Hz, 3H); ^13^C NMR (150 MHz (CD_3_)_2_SO) *δ* 157.65, 140.57, 134.33, 133.14, 129.58, 129.32, 128.62, 127.94, 126.89, 126.57, 126.51, 126.37, 121.87, 119.93, 116.81, 114.50, 113.15, 112.95, 19.49, 15.36; *ν*_max_/cm^−1^ 3313, 2928, 1613, 1544, 1247; HRMS (ESI) *m*/*z* [M + H]^+^ calcd for C_22_H_20_ON_4_^79^Br 435.0815; found 435.0812.

#### 4.1.12. (*E*)-5-Bromo-*N*′-((3-ethyl-1*H*-pyrrol-2-yl)methylene)-3-phenyl-1*H*-indole-2-carbohydrazide (3u)

Yellow solid (0.106 g, 0.24 mmol, 92%); *R*_f_ 0.5 [EtOAc–hexane (1 : 1)]; mp 154 °C decomposed; ^1^H NMR (600 MHz (CD_3_)_2_SO) *δ* 12.09 (s, 1H), 11.17 (s, 1H), 10.96 (s, 1H), 7.95 (s, 1H), 7.68 (s, 1H), 7.48–7.41 (m, 5H), 7.34 (dd, *J* = 8.6, 1.9 Hz, 1H), 7.31 (t, *J* = 7.4 Hz, 1H), 6.76 (s, 1H), 5.95 (s, 1H), 2.43 (q, *J* = 7.5 Hz, 2H), 1.06 (t, *J* = 7.6 Hz, 3H); ^13^C NMR (150 MHz (CD_3_)_2_SO) *δ* 157.47, 139.53, 134.30, 133.17, 130.74, 129.59, 129.39, 128.59, 127.93, 126.82, 126.34, 122.29, 122.25, 121.85, 116.76, 114.47, 112.93, 109.21, 18.54, 16.03; *ν*_max_/cm^−1^ 3312, 3058, 2925, 1606, 1538, 1248; HRMS (ESI) *m*/*z* [M + H]^+^ calcd for C_22_H_20_ON_4_^79^Br 435.0815; found 435.0811.

#### 4.1.13. (*E*)-5-Bromo-*N*′-((3,5-dimethyl-1*H*-pyrrol-2-yl)methylene)-3-phenyl-1*H*-2-carbohydrazide (3c)

To a solution of hydrazide 2 (0.081 g, 0.25 mmol) in EtOH (10 mL) was added 3,5-dimethyl-2-pyrrolecarboxaldehyde (0.031 g, 0.25 mmol) and a few drops of acetic acid. The resulting mixture was heated to 80 °C, and stirred for 17 h. The reaction mixture was allowed to cool to room temperature. Precipitated solid was filtered, and was washed with EtOH (10 mL) to give indole hydrazide 3c (0.073 g, 0.17 mmol, 68%) as a yellow solid; mp 153–155 °C decomposed; ^1^H NMR (600 MHz (CD_3_)_2_SO) *δ* 11.96 (s, 1H), 10.82 (s, 1H), 10.78 (s, 1H), 7.72 (s, 1H), 7.57 (s, 1H), 7.36–7.30 (m, 5H), 7.24 (dd, *J* = 8.7, 1.9 Hz, 1H), 7.21 (t, *J* = 7.2 Hz, 1H), 5.52 (s, 1H), 2.00 (s, 3H), 1.84 (s, 3H); ^13^C NMR (150 MHz (CD_3_)_2_SO) *δ* 157.52, 139.35, 134.39, 133.28, 133.00, 129.66, 129.53, 128.72, 128.04, 126.96, 126.40, 124.78, 121.96, 121.60, 116.73, 114.57, 113.01, 109.92, 12.84, 10.83; *ν*_max_/cm^−1^ 3312, 3058, 2863, 1610, 1545, 1241; HRMS (ESI) *m*/*z* [M + H]^+^ calcd for C_22_H_20_ON_4_^79^Br 435.0815; found 435.0809. Compounds 3d, 3i–j, 3l–n, 3p–r and 3v–x were synthesized following the procedure described here.

#### 4.1.14. (*E*)-5-Bromo-*N*′-((1-methyl-1*H*-pyrrol-2-yl)methylene)-3-phenyl-1*H*-indole-2-carbohydrazide (3d)

Brown solid (0.052 g, 0.12 mmol, 57%); mp 261 °C decomposed; ^1^H NMR (600 MHz (CD_3_)_2_SO) *δ* 8.01 (s, 1H), 7.87 (s, 0.3H)*, 7.75 (s, 0.3H)*, 7.71 (s, 1H), 7.54–7.34 (m, 7H), 7.25 (s, 0.3H), 6.97 (s, 1H), 6.79 (s, 0.3H)*, 6.46 (s, 1H), 6.30 (s, 0.3H)*, 6.09 (s, 1H), 5.99 (s, 0.3H)*, 3.84 (s, 3H); ^13^C NMR (100 MHz (CD_3_)_2_SO) *δ* 157.57, 140.67, 134.25, 133.08, 129.62, 129.14, 128.52, 128.33, 127.98, 126.83, 126.76, 126.38, 121.84, 117.05, 114.96, 114.47, 112.90, 108.46, 35.94; *ν*_max_/cm^−1^ 3262, 3051, 1649, 1535, 1236; HRMS (ESI) *m*/*z* [M + H]^+^ calcd for C_21_H_18_ON_4_^79^Br 421.0659; found 421.0655.

* Rotamer.

^1^H NMR (600 MHz (CD_3_)_2_SO, 90 °C) *δ* 7.99 (s, 1H), 7.69 (d, *J* = 1.9 Hz, 1H), 7.54–7.53 (m, 2H), 7.48–7.46 (m, 3H), 7.37–7.34 (m, 2H), 6.89 (s, 1H), 6.41 (s, 1H), 6.07 (s, 1H), 3.74 (s, 3H).

#### 4.1.15. Ethyl (*E*)-5-((2-(5-bromo-3-phenyl-1*H*-indole-2-carbonyl)hydrazono)methyl)-1*H*-pyrrole-2-carboxylate (3i)

Yellow solid (0.114 g, 0.24 mmol, 96%); mp 247–249 °C; ^1^H NMR (600 MHz (CD_3_)_2_SO) *δ* 12.36 (s, 1H), 12.16 (s, 1H), 11.95 (s, 0.3H)*, 11.89 (s, 0.3H)*, 11.76 (s, 0.3H)*, 11.46 (s, 1H), 8.05 (s, 1H), 7.86 (s, 0.3H)*, 7.72 (s, 1H), 7.53–7.36 (m, 7H), 7.22 (s, 0.3H)*, 6.83 (s, 1H), 6.72 (s, 0.3H)*, 6.63 (s, 1H), 6.27 (s, 0.3H)*, 4.25 (q, *J* = 7.1 Hz, 2H), 1.29 (t, *J* = 7.1 Hz, 3H); ^13^C NMR (100 MHz (CD_3_)_2_SO) *δ* 160.49, 158.43, 139.85, 134.79, 133.47, 132.87, 130.06, 129.19, 128.98, 128.35, 127.35, 126.98, 124.76, 122.35, 117.90, 116.71, 114.98, 113.40, 110.31, 60.39, 14.83; *ν*_max_/cm^−1^ 3252, 1683, 2852, 1642, 1544, 1228; HRMS (ESI) *m*/*z* [M + H]^+^ calcd for C_23_H_20_O_3_N_4_^79^Br 479.0713; found 479.0714.

* Rotamer.

^1^H NMR (600 MHz (CD_3_)_2_SO, 90 °C) *δ* 11.84 (s, 2H), 11.08 (s, 1H), 8.03 (s, 1H), 7.69 (d, *J* = 1.9 Hz, 1H), 7.53–7.50 (m, 3H), 7.6 (t, *J* = 7.6 Hz, 2H), 7.40 (dd, *J* = 8.7, 1.9 Hz, 1H), 7.35 (t, *J* = 6.8 Hz, 1H), 6.81 (d, *J* = 3.9 Hz, 1H), 6.54 (s, 1H), 4.29 (q, *J* = 7.1 Hz, 2H), 1.32 (t, *J* = 7.1 Hz, 3H).

#### 4.1.16. Ethyl (*E*)-5-((2-(5-bromo-3-phenyl-1*H*-indole-2-carbonyl)hydrazono)methyl)-1*H*-pyrrole-3-carboxylate (3j)

Yellow solid (0.084 g, 0.18 mmol, 69%); mp 230 °C decomposed; ^1^H NMR (600 MHz (CD_3_)_2_SO) *δ* 12.11 (s, 2H), 11.27 (s, 1H), 7.89 (s, 1H), 7.68 (s, 1H), 7.47–7.30 (m, 8H), 6.76 (s, 1H), 4.13 (q, *J* = 7.1 Hz, 2H), 1.20 (t, *J* = 7.1 Hz, 3H); ^13^C NMR (100 MHz (CD_3_)_2_SO) *δ* 163.55, 157.96, 139.68, 134.36, 133.05, 129.57, 128.96, 128.62, 128.26, 127.89, 127.28, 126.92, 126.51, 121.92, 117.16, 116.54, 114.54, 113.60, 112.99, 59.29, 14.40; *ν*_max_/cm^−1^ 3320, 2976, 1686, 1538, 1210; HRMS (ESI) *m*/*z* [M + H]^+^ calcd for C_23_H_20_O_3_N_4_^79^Br 479.0713; found 479.0711.

#### 4.1.17. (*E*)-5-((2-(5-Bromo-3-phenyl-1*H*-indole-2-carbonyl)hydrazono)methyl)-1*H*-pyrrole-2-carboxylic acid (3l)

Yellow solid (0.068 g, 0.15 mmol, 60%); mp 227 °C decomposed; ^1^H NMR (400 MHz (CD_3_)_2_SO) *δ* 12.15 (s, 1H), 12.05 (s, 1H), 11.35 (s, 1H), 7.93 (s, 1H), 7.62 (s, 1H), 7.43–7.26 (m, 8H), 6.69 (s, 1H), 6.53 (s, 1H); ^13^C NMR (100 MHz (CD_3_)_2_SO) *δ* 161.55, 158.08, 139.56, 134.39, 133.06, 131.95, 129.67, 128.76, 128.62, 127.98, 126.98, 126.60, 125.42, 121.96, 117.52, 116.09, 114.60, 113.02, 110.06; *ν*_max_/cm^−1^ 3312, 3151, 1692, 1637, 1491, 1209; HRMS (ESI) *m*/*z* [M + H]^+^ calcd for C_21_H_16_O_3_N_4_^79^Br 451.0400; found 451.0400.

#### 4.1.18. (*E*)-5-((2-(5-Bromo-3-phenyl-1*H*-indole-2-carbonyl)hydrazono)methyl)-1*H*-pyrrole-3-carboxylic acid (3m)

Yellow solid (0.062 g, 0.14 mmol, 58%); mp 260 °C decomposed; ^1^H NMR (600 MHz (CD_3_)_2_SO) *δ* 12.14 (s, 1H), 12.05 (s, 1H), 11.93 (s, 1H), 11.26 (s, 1H), 7.91 (s, 1H), 7.70 (s, 1H), 7.49–7.32 (m, 8H), 6.75 (s, 1H); ^13^C NMR (100 MHz (CD_3_)_2_SO) *δ* 165.04, 157.89, 139.84, 134.33, 133.04, 129.54, 129.02, 128.59, 128.02, 127.87, 127.23, 126.89, 126.46, 121.89, 117.41, 117.07, 114.51, 113.96, 112.96; *ν*_max_/cm^−1^ 3217, 1670, 1636, 1549, 1200; HRMS (ESI) *m*/*z* [M + H]^+^ calcd for C_21_H_16_O_3_N_4_^79^Br 451.0400; found 451.0395.

#### 4.1.19. (*E*)-5-Bromo-*N*′-((5-(morpholine-4-carbonyl)-1*H*-pyrrol-2-yl)methylene)-3-phenyl-1*H*-indole-2-carbohydrazide (3n)

Yellow solid (0.11 g, 0.21 mmol, 81%); mp 178–180 °C; ^1^H NMR (600 MHz (CD_3_)_2_SO) *δ* 12.10 (s, 1H), 11.92 (s, 1H), 11.31 (s, 1H), 7.98 (s, 1H), 7.68 (s, 1H), 7.48–7.32 (m, 7H), 6.55 (s, 1H), 6.52 (s, 1H), 3.62 (s, 4H), 3.58 (t, *J* = 4.2 Hz, 4H); ^13^C NMR (100 MHz (CD_3_)_2_SO) *δ* 161.05, 157.90, 139.71, 134.33, 133.06, 129.77, 129.63, 128.88, 128.58, 127.93, 126.99, 126.91, 126.50, 121.91, 117.30, 114.53, 113.35, 112.96, 109.87, 66.22; *ν*_max_/cm^−1^ 3213, 3054, 2856, 1613, 1489, 1233; HRMS (ESI) *m*/*z* [M + H]^+^ calcd for C_25_H_23_O_3_N_5_^79^Br 520.0979; found 520.0981.

#### 4.1.20. (*E*)-5-Bromo-*N*′-((5-nitro-1*H*-pyrrol-2-yl)methylene)-3-phenyl-1*H*-indole-2-carbohydrazide (3p)

Yellow solid (0.092 g, 0.20 mmol, 74%); mp 140 °C decomposed; ^1^H NMR (600 MHz (CD_3_)_2_SO) *δ* 13.64 (s, 1H), 13.31 (s, 0.4H)*, 12.19 (s, 1H), 11.98 (s, 0.4H)*, 11.72 (s, 1H), 8.05 (s, 1H), 7.74 (s, 1H), 7.50–7.37 (m, 7H), 7.20 (s, 1H), 7.08 (s, 0.4H)*, 6.74 (s, 1H), 6.29 (s, 0.4H)*; ^13^C NMR (150 MHz (CD_3_)_2_SO) *δ* 158.17, 138.41, 137.96, 134.43, 133.36, 132.90, 129.66, 128.55, 128.37, 127.86, 126.97, 126.74, 122.00, 117.93, 114.59, 113.05, 112.79, 110.30; *ν*_max_/cm^−1^ 3307, 1645, 1538, 1277; HRMS (ESI) *m*/*z* [M + H]^+^ calcd for C_20_H_15_O_3_N_5_^79^Br 452.0353; found 452.0352.

* Rotamer.

^1^H NMR (600 MHz (CD_3_)_2_SO, 90 °C) *δ* 13.23 (s, 1H), 11.88 (s, 1H), 11.36 (s, 1H), 8.03 (s, 1H), 7.70 (d, *J* = 1.9 Hz, 1H), 7.52–7.50 (m, 3H), 7.45 (t, *J* = 7.6 Hz, 2H), 7.40 (dd, *J* = 8.7, 1.9 Hz, 1H), 7.35 (t, *J* = 7.4 Hz, 1H), 7.14 (d, *J* = 4.3 Hz), 6.62 (s, 1H).

#### 4.1.21. (*E*)-5-Bromo-*N*′-((4-nitro-1*H*-pyrrol-2-yl)methylene)-3-phenyl-1*H*-indole-2-carbohydrazide (3q)

Yellow solid (0.056 g, 0.12 mmol, 46%); mp 302 °C decomposed; ^1^H NMR (400 MHz (CD_3_)_2_SO) *δ* 12.72 (s, 1H), 12.17 (s, 1H), 11.47 (s, 1H), 7.95 (s, 1H), 7.90 (s, 1H), 7.72 (s, 1H), 7.49–7.33 (m, 7H), 7.07 (s, 1H); ^13^C NMR (100 MHz (CD_3_)_2_SO) *δ* 158.08, 138.74, 137.00, 134.38, 132.96, 129.53, 128.68, 128.56, 128.05, 127.83, 126.90, 126.56, 123.80, 121.92, 117.40, 114.54, 112.98, 107.54; *ν*_max_/cm^−1^ 3312, 3138, 1640, 1559, 1285; HRMS (ESI) *m*/*z* [M + H]^+^ calcd for C_20_H_15_O_3_N_5_^79^Br 452.0353; found 452.0352.

#### 4.1.22. (*E*)-5-Bromo-*N*′-((3,5-dimethyl-4-nitro-1*H*-pyrrol-2-yl)methylene)-3-phenyl-1*H*-indole-2-carbohydrazide (3r)

Brown solid (0.096 g, 0.2 mmol, 77%); mp 181 °C decomposed; ^1^H NMR (600 MHz (CD_3_)_2_SO) *δ* 12.33 (s, 1H), 12.15 (s, 1H), 11.28 (s, 1H), 8.02 (s, 1H), 7.70 (s, 1H), 7.49–7.30 (m, 7H), 2.51 (s, 3H), 2.23 (s, 3H); ^13^C NMR (100 MHz (CD_3_)_2_SO) *δ* 157.81, 137.85, 137.48, 134.39, 133.00, 132.71, 129.55, 128.92, 128.61, 127.85, 126.91, 126.54, 122.00, 121.93, 119.36, 117.20, 114.53, 113.03, 13.73, 10.30; *ν*_max_/cm^−1^ 3254, 1620, 1545, 1359; HRMS (ESI) *m*/*z* [M + H]^+^ calcd for C_22_H_19_O_3_N_5_^79^Br 480.0666; found 480.0665.

#### 4.1.23. Ethyl (*E*)-4-((2-(5-bromo-3-phenyl-1*H*-indole-2-carbonyl)hydrazono)methyl)-1*H*-pyrrole-2-carboxylate (3v)

White solid (0.07 g, 0.15 mmol, 60%); mp 248–250 °C; ^1^H NMR (400 MHz (CD_3_)_2_SO) *δ* 12.18 (s, 1H), 12.07 (s, 1H), 11.07 (s, 1H), 7.92 (s, 1H), 7.67 (s, 1H), 7.48_7.30 (m, 8H), 7.00 (s, 1H), 4.23 (q, *J* = 7.1 Hz, 2H), 1.26 (t, *J* = 7.1 Hz, 3H); ^13^C NMR (100 MHz (CD_3_)_2_SO) *δ* 160.19, 157.78, 143.19, 134.28, 133.10, 129.60, 129.14, 128.58, 127.97, 126.90, 126.40, 125.86, 123.75, 121.85, 120.51, 116.98, 114.49, 112.92, 112.30, 59.97, 14.33; *ν*_max_/cm^−1^ 3268, 3064, 2982, 1690, 1659, 1543, 1237; HRMS (ESI) *m*/*z* [M + H]^+^ calcd for C_23_H_20_O_3_N_4_^79^Br 479.0713; found 479.0715.

#### 4.1.24. (*E*)-4-((2-(5-Bromo-3-phenyl-1*H*-indole-2-carbonyl)hydrazono)methyl)-1*H*-pyrrole-2-carboxylic acid (3w)

Pale yellow solid (0.079 g, 0.18 mmol, 69%); mp 277 °C decomposed; ^1^H NMR (400 MHz (CD_3_)_2_SO) *δ* 12.03 (s, 1H), 12.00 (s, 1H), 11.00 (s, 1H), 7.88 (s, 1H), 7.63 (s, 1H), 7.44–7.26 (m, 8H), 6.91 (s, 1H); ^13^C NMR (100 MHz (CD_3_)_2_SO) *δ* 161.69, 157.79, 143.50, 134.30, 133.13, 129.63, 129.18, 128.62, 128.01, 126.94, 126.43, 125.58, 124.75, 121.88, 120.37, 117.00, 114.52, 112.95, 111.97; *ν*_max_/cm^−1^ 3311, 3133, 1697, 1636, 1555, 1240; HRMS (ESI) *m*/*z* [M + H]^+^ calcd for C_21_H_16_O_3_N_4_^79^Br 451.0400; found 451.0397.

#### 4.1.25. (*E*)-5-Bromo-*N*′-((5-(morpholine-4-carbonyl)-1*H*-pyrrol-3-yl)methylene)-3-phenyl-1*H*-indole-2-carbohydrazide (3x)

Pale yellow solid (0.107 g, 0.20 mmol, 83%); mp 282–284 °C; ^1^H NMR (600 MHz (CD_3_)_2_SO) *δ* 12.07 (s, 1H), 11.80 (s, 1H), 11.02 (s, 1H), 7.91 (s, 1H), 7.67 (s, 1H), 7.47–7.26 (m, 8H), 6.73 (s, 1H), 3.66 (s, 4H), 3.59 (t, *J* = 4.8 Hz, 4H); ^13^C NMR (100 MHz (CD_3_)_2_SO) *δ* 160.96, 157.69, 143.68, 134.28, 133.11, 129.58, 129.23, 128.59, 127.95, 126.89, 126.37, 125.80, 124.23, 121.84, 119.59, 116.86, 114.49, 112.92, 108.76, 66.23; *ν*_max_/cm^−1^ 3238, 2853, 1631, 1596, 1556, 1252; HRMS (ESI) *m*/*z* [M + H]^+^ calcd for C_25_H_23_O_3_N_5_^79^Br 520.0979; found 520.0980.

### 4.2. NCI60 screening

The synthesized compounds were evaluated their anticancer properties by submitted to National Cancer Institute 60 human tumour cell lines (NCI60) screening program in the USA.

#### 4.2.1. One-dose screen

The one-dose experimental protocol followed the method described in this literature.^28^ First, all compounds were tested at a single concentration (10 µM) across the entire NCI60 panel. The results of this assay indicate the growth relative to no-drug control and the number of cells at time zero. This method allows for the detection of growth inhibition (values between 0 to 100) and lethality (value below 0).

#### 4.2.2. Five-dose screen

The five-dose experimental protocol followed the method described in this literature.^28^ Compounds that displayed significant results in the single-dose screen were selected for further experiment across the 60 cell panel at five concentration levels (10 nM, 100 nM, 1 µM, 10 µM and 100 µM). Briefly, the cancer screening panels were cultured in RPMI 1640 medium supplemented with 5% fetal bovine serum and 2 mM l-glutamine. First, cells were plated into 96-well plates at densities in range 5000 to 40 000 cells/well and incubated at 37 °C for 24 hours. After incubation, some plates were treated with trichloroacetic acid (TCA) to determine the time of drug addition (*T*_z_) for each cell line. The plates containing the test compounds at five different concentrations were incubated for 48 hours, fixed, stained with sulforhodamine B (SRB), and incubated at room temperature for 10 minutes. An automate plate was used to measure the absorbance at a wavelength of 515 nm. The percentage of growth inhibition was calculated by use the absorbance from time zero (*T*_z_), control growth (*C*), and test growth in the presence of drug at the five concentration levels (*T*_i_) using this equation.[(*T*_i_ − *T*_z_)/(*C* − *T*_z_)] × 100 for concentrations which *T*_i_ ≥ *T*_z_[(*T*_i_ − *T*_z_)/*T*_z_] × 100 for concentration which *T*_i_ < *T*_z_

The results are reported as Growth Inhibition at 50% (GI_50_), Total Growth Inhibition (TGI), and Median Lethal Concentration (LC_50_).

### 4.3. Tubulin polymerisation assay

The tubulin polymerisation experimental protocol followed the method described in this literature.^28^ The experimental was using fluorescence-based assay kit (Cytoskeleton, catalog no. BK011P). In Brief, 5 µL of test compounds was added to a 96-well plate, then mix with 50 µL of a solution containing porcine brain tubulin (2 mg mL^−1^) in buffer with 20% glycerol and 1 mM GTP. A microplate reader (Varioskan™ LUX multimode microplate reader, Thermo Scientific™) was used to measure fluorescence intensity every 60 seconds for 60 minutes, with excitation at 360 nm and emission at 450 nm, maintained at 37 °C throughout the experiment.

### 4.4. Cell cycle

A549 human non-small cell lung cancer cell line (ATCC CCL-185) was purchased from American Type Culture Collection (Rockville, MD, USA). Analysis of cell cycle phase distribution was performed as previously described.^40^ Briefly, A549 cells in 6-well plate (1 × 10^5^ cells per well) were treated with test compound (or 0.2% DMSO for control). After 24 h treatment, the treated cells were harvested and subjected to cell cycle analysis using Muse Cell Cycle Assay kit (Luminex, Austin, TX, USA) and analyzed with Muse Cell Analyzer™ with (Luminex). The number of cells in each cell cycle phase was expressed as percentage of total cells.

### 4.5. Aromatase inhibition assay

Aromatase enzyme was purchased from GENTEST, Woburn, MA01801, USA (Supersom human CYP19+oxidoreductase, baculovirus/insect cell-expressed; Product No. 456260). The aromatase inhibitory activity was performed using Human CYP19 enzymes and DBF as a fluorometric substrate as described by Stresser, *et al.*^41^ with minor modifications. The mixture, containing 100 µL of cofactor/serial dilution buffer was pre-incubated in 37 °C for 10 min. The reaction was initiated by addition of 100 µL of enzyme/substrate and 10 µL of test compounds (in 10% DMSO) or 10% DMSO as solvent control or 5 µM letrozole (6.3 nM final concentration) as positive control. After incubation at 37 °C for 30 min, the reaction was stopped by addition of 50 µL of 2.2 M NaOH. Fluorescence signal was monitored using an excitation wavelength of 490 nm and emission wavelength of 530 nm. The test compounds showing inhibition >50% were expressed as the half-maximal inhibitory concentration (IC_50_).

### 4.6. Cytotoxicity assay

T47D (ATCC HTB-133) was purchased from Biomedia (Thailand) Co., LTD. L929 (ATCC CCL-1) was received as a gift from Prof. Tanapat Palaga, Department of Microbiology, Faculty of Science, Chulalongkorn University, Bangkok, Thailand. The cells were cultured in Dulbecco's Modified Eagle's Medium (DMEM; Gibco) supplemented with 10% fetal bovine serum (FBS; HyClone), 100 U mL^−1^ penicillin and 100 µg mL^−1^ streptomycin (Gibco) at 37 °C with 5% CO_2_.

The antiproliferative activity was determined by MTT assay. Briefly, the cells were plated into a 96-well microplate at the density of 10 000 cells per well. The cells were allowed to grow overnight before treated with the solutions of compounds in culture medium at the designated concentrations for 48 hours. The medium was then replaced with MTT solution (0.5 mg mL^−1^ MTT in culture medium) and incubated for 3 hours. A 100 µL of DMSO was used to dissolve the produced formazan. Absorbance at 570 nm was then measured using a microplate reader (Varioskan™ LUX multimode microplate reader, Thermo Scientific™). Percent viability was calculated relative to the vehicle control (DMSO).

### 4.7. Molecular docking calculations

The ligands were constructed using Chem3D Pro 12.0 and energy minimized using the MM2 (ref. 42) force field. The tubulin structure was downloaded from the Protein Data Bank (PDB):^43^ tubulin (PDB ID: 4O2B, resolution 2.3 Å),^44^ as aromatase (PDB ID: 3S7S, resolution 3.2 Å).^45^ The protein structure were prepared for the docking simulations*, i.e.*, co-crystallised colchicine and spectator ligands were removed. The centre of the colchicine binding pocket of tubulin, and aromatase binding site were defined at coordinates (*x* = 13.222, *y* = 8.371, *z* = −23.331) and (*x* = 85.219, *y* = 49.732, *z* = 42.202), respectively, with 10 Å radius. The docking runs were set at fifty docking runs per ligand with default search efficiency (100%) to permit thorough searches for the best bioactive conformations. Basic amino acids including lysine and arginine were defined as protonated, whilst acidic amino acids including aspartic and glutamic acids were deprotonated. The GoldScore (GS) scoring function was implemented to calculate the binding score and predict the binding modes, which was included in the GOLD version 2023.2.0 software suite.^46^ Discovery Studio 4.5 visualizer was used as a visualizer to examine and analyse the predicted binding modes and intermolecular interactions.

### 4.8. Calculations of physicochemical properties

The Dragon 7.0 software package was automated to calculate the molecular descriptors of the derivatives: MWs, number of HAs and HDs, number of RBs, PSA and *M*logP. The ADMETlab 3.0 web server was automated to predict for the toxicities of the compounds.

## Author contributions

Rungroj Saruengkhanphasit: conceptualization, project administration, methodology, data curation, writing – original draft, writing – review & editing, funding acquisition. Jaruwan Chatwichien: methodology. Lukana Ngiwsara: methodology. Kriengsak Lirdprapamongkol: methodology, writing – review & editing, funding acquisition. Worawat Niwetmarin: methodology, writing – review & editing. Chatchakorn Eurtivong: conceptualization, methodology, writing – review & editing. Prasat Kittakoop: conceptualization, data curation, supervision, writing – review & editing. Jisnuson Svasti: data curation, writing – review & editing. Somsak Ruchirawat: data curation, writing – review & editing.

## Conflicts of interest

The authors declare no competing financial interest.

## Supplementary Material

RA-015-D4RA09000D-s001

## Data Availability

The data supporting this article have been included as part of the ESI.†
