# Supplementary material for: Design, synthesis, in silico and in vitro evaluation of pyrrole–indole hybrids as dual tubulin and aromatase inhibitors with potent anticancer activities
Source: RSC Adv. 2025 Jun 27;15(27):21962–76. doi: 10.1039/d4ra09000d (PMC12203311; doi:10.1039/d4ra09000d)
Supplement: RA-015-D4RA09000D-s001 [file RA-015-D4RA09000D-s001.pdf]

Supplementary Information (SI) for RSC Advances.
This journal is © The Royal Society of Chemistry 2025

Supplementary Information

Design, synthesis, *in silico* and *in vitro* evaluation of pyrrole-indole hybrids as dual tubulin and aromatase inhibitors with potent anticancer activities

Rungroj Saruengkhanphasit,[1, 2*] *Jaruwan Chatwichien,*[1, 3] *Lukana Ngiwsara,*[4] *Kriengsak Lirdprapamongkol,*[2, 4] *Worawat Niwetmarin,*[1] *Chatchakorn Eurtivong,*[5] *Prasat Kittakoop,*[1, 2, 6] *Jisnuson, Svasti,*[4] *Somsak Ruchirawat*[1, 2, 7]

[1]Chulabhorn Graduate Institute, Program in Chemical Sciences, 54 Kamphaeng Phet 6, Talat Bang Khen, Lak Si, Bangkok 10210, Thailand.

[2]Center of Excellence On Environmental Health and Toxicology (EHT), OPS, Ministry of Higher Education, Science, Research and Innovation, Bangkok, Thailand.

[3]Chulabhorn Royal Academy, Bangkok 10210, Thailand.

[4]Laboratory of Biochemistry, Chulabhorn Research Institute, Bangkok 10210, Thailand.

[5]Department of Pharmaceutical Chemistry, Faculty of Pharmacy, Mahidol University, 447 Si Ayutthaya Road, Ratchathewi, Bangkok 10400, Thailand.

[6]Laboratory of Natural Products, Chulabhorn Research Institute, Bangkok 10210, Thailand.

[7]Laboratory of Medicinal Chemistry, Chulabhorn Research Institute, Bangkok 10210, Thailand.

Table of content

Synthesis of various aldehydes page 2–8

^{1}H and ^{13}C Spectra page 9–55

Figure S1. NCI60 one dose screen for compounds **3a**–**x** page 56–79

Table S2. GI_{50} and LC_{50} screen for compounds **3a-b**, **3n**, and **3p-3x** page 80–83

Figure S3. NCI60 five dose screen for compounds **3a-b**, **3n**, and **3p-3x** page 84–119

Figure S4. Screening derivatives **3a**–**x** against T–47D page 120

Figure S5. Correlations between IC_{50} values and GI_{50} values page 121

Figure S6. Overlay between the co-crystallized colchicine page 122

Figure S7. Overlay between the co-crystallized exemestane page 122

Figure S8. Physicochemical properties of compounds **3a**–**x** page 123

Table S9. Toxicity evaluation of the most active compounds. page 124

Synthesis of various aldehyde

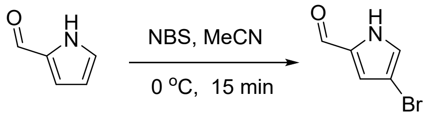

4-bromo-1*H*-pyrrole-2-carbaldehyde

To a solution of pyrrole-2-carboxaldehyde (0.302 g, 3.18 mmol) in MeCN (5 mL) was added *N*-bromosuccinimide (0.566 g, 3.18 mmol) at 0 °C. The reaction mixture was stirred at 0 °C for 15 min. The mixture was diluted by H_2O (5 mL) and was extracted with Et_2O (3 x 10 mL). The combined organic layers were dried (Na_2SO_4), filtered, and the solvent was evaporated. Purification by flash column chromatography, eluted with EtOAc-Hexane (3:7), gave 4-bromo-1*H*-pyrrole-2-carbaldehyde (0.305 g, 1.76 mmol, 55%) as a white solid; R_f 0.6 [EtOAc-Hexane (3:7)]; m.p. 123–125 °C, lit.[1] m.p. 122–124 °C; ^{1}H NMR (600 MHz, $CDCl_3$) δ 9.73 (br. s, 1H), 9.50 (s, 1H), 7.14–7.13 (m, 3H), 6.99 (m, 1H) Data were in agreement to those reported in the literature.[1]

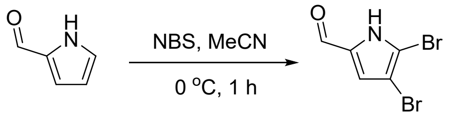

4,5-dibromo-1*H*-pyrrole-2-carbaldehyde

To a solution of pyrrole-2-carboxaldehyde (0.316 g, 3.32 mmol) in MeCN (5 mL) was added *N*-bromosuccinimide (1.21 g, 6.80 mmol) at 0 °C. The reaction mixture was stirred at 0 °C for 1 h. The mixture was diluted by H_2O (5 mL) and was extracted with Et_2O (3 x 10 mL). The combined organic layers were dried (Na_2SO_4), filtered, and the solvent was evaporated. Purification by flash column chromatography, eluted with EtOAc-Hexane (3:7), gave 4,5-dibromo-1*H*-pyrrole-2-carbaldehyde (0.765 g 3.02 mmol, 91%) as a white solid; R_f 0.6 [EtOAc-Hexane (3:7)]; m.p. 148–150 °C, lit.[1] m.p. 144–146 °C; ^{1}H NMR (300 MHz, $CDCl_3$) δ 10.09 (br. s, 1H), 9.35 (s, 1H), 6.96 (s, 1H) Data were in agreement to those reported in the literature.[2]

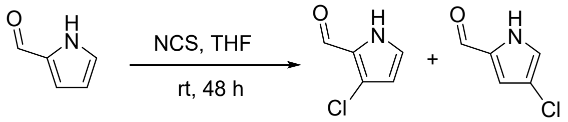

3-chloro-1*H*-pyrrole-2-carbaldehyde and 4-chloro-1*H*-pyrrole-2-carbaldehyde

To a solution of pyrrole-2-carboxaldehyde (0.249 g, 2.62 mmol) in THF (5 mL) was added *N*-chlorosuccinimde (0.390 g, 2.92 mmol) at room temperature. The reaction mixture was stirred at room temperature for 48 h. The mixture was dilute by MeOH (3 mL) and was evaporated. Purification by flash column chromatography, eluted with EtOAc-Hexane (0:100 to 1.5:8.5), gave 3-chloro-1*H*-pyrrole-2-carbaldehyde (0.156 g) as a white solid and 4-chloro-1*H*-pyrrole-2-carbaldehyde (0.051 g) as a yellow solid. The compounds were used in the next step without characterisation.

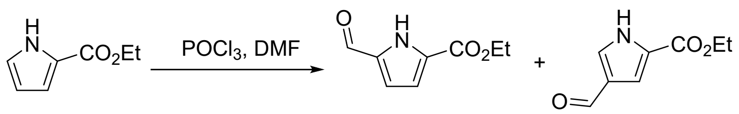

Ethyl 5-formyl-1*H*-pyrrole-2-carboxylate and **ethyl 4-formyl-1*H*-pyrrole-2-carboxylate**

To a solution of ethyl 1*H*-pyrrole-2-carboxylate (1.06 g, 7.62 mmol) in anhydrous DMF (14 mL) was added $POCl_3$ (2.1 mL, 15.7 mmol) at 0 °C. The reaction mixture was allowed to warm to room temperature 15 min and stirred at room temperature for 16 h. The mixture was diluted by H_2O (8 mL) and was added 25% aqueous NH_3 until the pH of solution adjust to 7. The aqueous layer was extracted with CH_2Cl_2 (3 x 20 mL). The combind organic layers were washed with saturated aqueous $NaHCO_3$ (25 mL), brine (25 mL), was dried (Na_2SO_4), and was evaporated. Purification by flash column chromatography, eluted with EtOAc-Hexane (3:7 to 1:1), gave ethyl 5-formyl-1*H*-pyrrole-2-carboxylate (0.514 g, 3.07 mmol, 40%) as a yellow solid and ethyl 4-formyl-1*H*-pyrrole-2-carboxylate (0.544 g, 3.25 mmol, 43%) as a yellow solid.

Ethyl 5-formyl-1*H*-pyrrole-2-carboxylate; R_f 0.6 [EtOAc-Hexane (3:7)]; m.p. 71–74 °C, lit.[3] m.p. 122–124 °C; ^1H NMR (300 MHz, $CDCl_3$) δ 9.83 (br. s, 1H), 9.68 (s, 1H), 6.97 (d, *J* = 2.5 Hz, 2H), 4.44 (q, *J* = 7.1 Hz, 2H), 1.43 (t, *J* = 7.1 Hz, 3H) Data were in agreement to those reported in the literature.[3]

Ethyl 4-formyl-1*H*-pyrrole-2-carboxylate; R_f 0.3 [EtOAc-Hexane (3:7)]; m.p. 100–102 °C, lit.[3] m.p. 101–102 °C; ^1H NMR (300 MHz, $CDCl_3$) δ 9.89 (br. s, 1H), 9.85 (s, 1H), 7.58 (dd, *J* = 3.4, 1.6 Hz, 2H), 7.33–7.31 (m, 1H), 4.44 (q, *J* = 7.1 Hz, 2H), 1.43 (t, *J* = 7.1 Hz, 3H) Data were in agreement to those reported in the literature.[3]

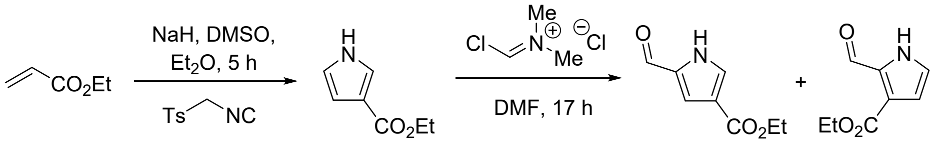

Ethyl 1*H*-pyrrole-3-carboxylate

Under Argon atmosphere, anhydrous Et_2O (20 mL) was added to NaH (60% in oil, 1.11 g, 27.72 mmol) at room temperature. With a magnetic stirring, TosMIC (3.0 g, 15.4 mmol) and ethyl acrylate (1.8 mL, 16.5 mmol) in anhydrous Et_2O-DMSO (20 mL: 10 mL) was added dropwise. The reaction mixture was stirred at room temperature for 5h. The mixture was diluted by H_2O (20 mL) and was extract with Et_2O (3 x 30 mL). The combined organic layers were dried (Na_2SO_4), filtered, and the solvent was evaporated. Purification by flash column chromatography, eluted with EtOAc-Hexane (1:9 to 3:7), gave ethyl 1*H*-pyrrole-3-carboxylate (0.883 g, 6.35 mmol, 41%) as a yellow oil; R_f 0.4 [EtOAc-Hexane (3:7)]; ^1H NMR (400 MHz, $CDCl_3$) δ 8.58 (br. s, 1H), 7.44–7.42 (m, 1H), 6.76–6.75 (m, 1H), 6.67–6.65 (m, 1H), 4.31 (q, *J* = 7.1 Hz, 2H), 1.36 (t, *J* = 7.1 Hz, 3H) Data were in agreement to those reported in the literature.[4]

Ethyl 5-formyl-1*H*-pyrrole-3-carboxylate and **ethyl 2-formyl-1*H*-pyrrole-3-carboxylate**

To a solution of ethyl 1*H*-pyrrole-3-carboxylate (0.883 g, 6.35 mmol) in anhydrous DMF (20 mL) was added (Chloromethylene)dimethyliminium chloride (1.3 g, 10.16 mmol) at 0 °C. The reaction mixture was allowed to warm to room temperature 15 min and stirred at room temperature for 16 h. The mixture was diluted by H_2O (10 mL) and was added saturated aqueous Na_2CO_3 until the pH of solution adjust to 7. The aqueous layer was extracted with CH_2Cl_2 (3 x 20 mL). The combind organic layers were washed with saturated aqueous $NaHCO_3$

(25 mL), brine (25 mL), was dried (Na_2SO_4), and was evaporated. Purification by flash column chromatography, eluted with EtOAc-Hexane (1:9 to 3:7), gave ethyl 5-formyl-1*H*-pyrrole-3-carboxylate (0.847 g, 5.07 mmol, 80%) as a yellow solid and ethyl 2-formyl-1*H*-pyrrole-3-carboxylate (0.137 g, 0.82 mmol, 13%) as a yellow solid.

Ethyl 5-formyl-1*H*-pyrrole-3-carboxylate; R_f 0.4 [EtOAc-Hexane (3:7)]; m.p. 90–93 °C, lit.[5] m.p. 84.1–85.1 °C; ^{1}H NMR (400 MHz, $CDCl_3$) δ 10.18 (br. s, 1H), 9.56 (d, *J* = 1.1 Hz, 1H), 7.71 (dt, *J* = 3.3, 1.3 Hz, 1H), 7.40 (dd, *J* = 2.5, 1.4 Hz, 1H), 4.35 (q, *J* = 7.1 Hz, 2H), 1.38 (t, *J* = 7.1 Hz, 3H); ^{13}C NMR (100 MHz, $CDCl_3$) δ 180.10, 163.74, 133.20, 129.92, 121.57, 119.36, 60.54, 14.52; ν_{max}/cm^{-1} 3254, 3051, 2838, 1699, 1651, 1189; HRMS (ESI) *m/z* $[M+H]^+$ calcd for $C_8H_{10}O_3N$ 168.0655; found 168.0654.

Ethyl 2-formyl-1*H*-pyrrole-3-carboxylate; R_f 0.6 [EtOAc-Hexane (3:7)]; m.p. 116_119 °C, lit.[6] m.p. 84.1–85.1 °C; ^{1}H NMR (400 MHz, $CDCl_3$) δ 10.23 (d, *J* = 1.0 Hz, 1H), 10.13 (br. s, 1H), 7.05 (td, *J* = 2.8, 1.0 Hz, 1H), 6.78 (t, *J* = 2.7 Hz, 1H), 4.40 (q, *J* = 7.1 Hz, 2H), 1.41 (t, *J* = 7.1 Hz, 3H); ^{13}C NMR (100 MHz, $CDCl_3$) δ 182.06, 163.73, 132.94, 124.13, 123.06, 113.85, 60.93, 14.47; ν_{max}/cm^{-1} 3205, 3123, 2901, 1697, 1633, 1118; HRMS (ESI) *m/z* $[M+H]^+$ calcd for $C_8H_{10}O_3N$ 168.0655; found 168.0654.

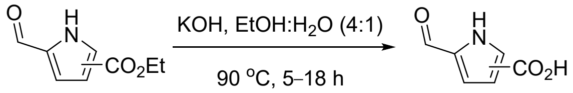

5-formyl-1*H*-pyrrole-2-carboxylic acid

To a solution of ethyl 5-formyl-1*H*-pyrrole-2-carboxylate (0.45 g, 2.69 mmol) and KOH (166 mg, 2.96 mmol) in EtOH/H_2O (4:1, 5 mL) was heated to 90 °C. The reaction mixture was stirred for 3–6 h. The reaction mixture was cooled to room temperature, and the solvent was evaporated. The residue was dissolved in H_2O (3 mL), acidified with conc. HCl until the pH of solution adjusted to 1–2. The percipitate product was filtered, washed with H_2O (3 x 10 mL) and dried over night to gave 5-formyl-1*H*-pyrrole-2-carboxylic acid (0.347 g, 2.49 mmol, 93%) as brown solid; m.p. decomposed; ^{1}H NMR (300 MHz, $(CD_3)_2SO$) δ 13.13 (br. s, 1H), 12.87 (br. s, 1H), 9.68 (s, 1H), 6.95–6.93 (m, 1H), 6.84–6.82 (m, 1H) Data were in agreement to those reported in the literature.[7] Compounds below were synthesized following the procedure described here.

4-formyl-1*H*-pyrrole-2-carboxylic acid

Brown solid (0.256 g, 1.84 mmol, 95%); m.p. decomposed; ^{1}H NMR (300 MHz, $(CD_3)_2SO$) δ 12.84 (br. s, 1H), 12.53 (br. s, 1H), 9.74 (s, 1H), 7.77 (dd, *J* = 3.4, 1.6 Hz, 1H), 7.08–7.06 (m, 1H); ^{13}C NMR (75 MHz, $(CD_3)_2SO$) δ 185.90, 161.62, 130.90, 126.67, 125.67, 112.70; ν_{max}/cm^{-1} 3287, 3119, 2877, 2766, 1677, 1436, 1119; HRMS (ESI) *m/z* $[M–H]^-$ calcd for $C_6H_4O_3N$ 138.0186; found 138.0182.

5-formyl-1*H*-pyrrole-3-carboxylic acid

To a solution of ethyl 5-formyl-1*H*-pyrrole-3-carboxylate (0.238 g, 1.42 mmol) and KOH (239 mg, 4.26 mmol) in EtOH/H_2O (1:1, 5 mL) was heated to 90 °C. The reaction mixture was stirred for 17 h. The reaction mixture was cool to room temperature, and the solvent was evaporated. The residue was dissolved in H_2O (3 mL), acidified with conc. HCl until the pH of solution adjusted to 1–2. The percipitate product was filtered, washed with H_2O (3 x 10 mL) and dried over night to gave 5-formyl-1*H*-pyrrole-2-carboxylic acid (0.139 g, 1.0 mmol, 70%)

as an orange solid; m.p. decomposed; ^{1}H NMR (400 MHz, $(CD_3)_2SO$) δ 12.59 (br. s, 1H), 12.25 (br. s, 1H), 9.55 (d, J = 1.0 Hz, 1H), 7.68–7.66 (m, 1H), 7.33–7.32 (m, 1H); ^{13}C NMR (100 MHz, $(CD_3)_2SO$) δ 180.36, 164.61, 133.31, 130.18, 120.92, 118.31; ν_{max}/cm^{-1} 3236, 3122, 2893, 2720, 1649, 1564, 1232; HRMS (ESI) *m/z* $[M–H]^-$ calcd for $C_6H_4O_3N$ 138.0186; found 138.0182.

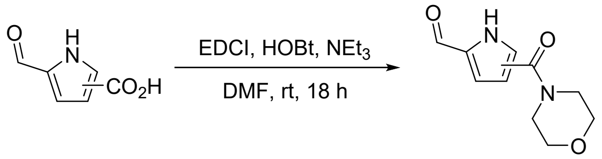

5-(morpholine-4-carbonyl)-1*H*-pyrrole-2-carbaldehyde

To a solution of 5-formyl-1*H*-pyrrole-2-carboxylic acid (0.119 g, 0.86 mmol), EDCI (0.247 g, 1.29 mmol), and HOBt (0.174 g, 1.29 mmol) in DMF (10 mL) was added morpholine (0.08 mL, 0.92 mmol) and NEt_3 (0.3 mL, 2.16 mmol) at roomtemperature. The reaction mixture was stirred at room temperature for 18 h. The mixture was diluted by H_2O (10 mL) and was extract with CH_2Cl_2 (3 x 30 mL). The combined organic layers were dried (Na_2SO_4), filtered, and the solvent was evaporated. Purification by flash column chromatography, eluted with MeOH-CH_2Cl_2 (1:99 to 1:19), gave 5-(morpholine-4-carbonyl)-1H-pyrrole-2-carbaldehyde (0.127 g, 0.61 mmol, 71%) as a white solid; R_f 0.6 [MeOH-CH_2Cl_2 (1:19)]; m.p. 160–162 °C; ^{1}H NMR (400 MHz, $(CD_3)_2SO$) δ 12.58 (br. s, 1H), 9.61 (s, 1H), 6.97 (d, J = 3.9 Hz, 1H), 6.57 (d, J = 4.0 Hz, 1H), 3.61 (s, 8H); ^{13}C NMR (100 MHz, $(CD_3)_2SO$) δ 180.55, 160.97, 133.54, 131.23, 117.52, 112.55, 66.10; ν_{max}/cm^{-1} 3183, 3086, 2856, 2819, 1673, 1589, 1233; HRMS (ESI) *m/z* $[M+H]^+$ calcd for $C_{10}H_{13}O_3N_2$ 209.0921; found 209.0917. Compounds below were synthesized following the procedure described here.

5-(morpholine-4-carbonyl)-1*H*-pyrrole-3-carbaldehyde

White solid (0.091 g, 0.44 mmol, 51%); R_f 0.5 [MeOH-CH_2Cl_2 (1:19)]; m.p. 172–175 °C; ^{1}H NMR (400 MHz, $CDCl_3$) δ 11.29 (br. s, 1H), 9.81 (s, 1H), 7.51 (dd, J = 3.4, 1.4 Hz, 1H), 6.94–6.93 (m, 1H), 3.87 (s, 4H), 3.77–3.74 (m, 4H); ^{13}C NMR (100 MHz, $CDCl_3$) δ 185.73, 161.50, 128.95, 127.14, 126.37, 110.63, 66.83; ν_{max}/cm^{-1} 3138, 3126, 2843, 1665, 1599, 1115; HRMS (ESI) *m/z* $[M–H]^-$ calcd for $C_{10}H_{11}O_3N_2$ 207.0764; found 207.0766.

4-(morpholine-4-carbonyl)-1*H*-pyrrole-2-carbaldehyde

Yellow solid (0.058 g, 0.29 mmol, 48%); R_f 0.4 [MeOH-CH_2Cl_2 (1:19)]; m.p. 180–182 °C decomposed; ^{1}H NMR (400 MHz, $(CD_3)_2SO$) δ 12.49 (br. s, 1H), 9.52 (d, J = 1.0 Hz, 1H), 7.51–7.50 (m, 1H), 7.20 (t, J = 7.9 Hz, 1H), 3.60 (s, 8H); ^{13}C NMR (100 MHz, $(CD_3)_2SO$) δ 180.02, 164.12, 132.42, 128.34, 120.34, 119.90, 66.19; ν_{max}/cm^{-1} 3172, 3001, 2923, 2858, 1658, 1591, 1114; HRMS (ESI) *m/z* $[M–H]^-$ calcd for $C_{10}H_{11}O_3N_2$ 207.0764; found 138.0765.

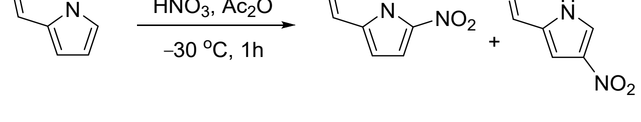

5-nitro-1*H*-pyrrole-2-carbaldehyde and **4-nitro-1*H*-pyrrole-2-carbaldehyde**

To acetic anhydride (4 mL) was added conc. HNO_3 (0.6 mL) at room temperature. After complete the addition, the nitration reagent was added dropwise to the solution of pyrrole-2-carboxaldehyde (0.511 g, 5.37 mmol) in acetic anhydride (5 mL) at –30 °C. The reaction

mixture was stirred at -30 °C for 1 h. The mixture was poured in H_2O (20 mL), basicdified with saturated aqueous Na_2CO3 until the pH of solution adjusted to 8, and was extracted with EtOAc (3 x 30 mL). The combined organic layers were dried (Na_2SO_4), filtered, and the solvent was evaporated. Purification by flash column chromatography, eluted with EtOAc-Hexane (1:9 to 3:7), gave 5-nitro-1*H*-pyrrole-2-carbaldehyde (0.097 g, 0.69 mmol, 13%) as a yellow solid and 4-nitro-1*H*-pyrrole-2-carbaldehyde (0.276 g, 1.97 mmol, 37%) as a yellow solid.

5-nitro-1*H*-pyrrole-2-carbaldehyde; R_f 0.4 [EtOAc-Hexane (3:7)]; m.p. 176-178 °C decomposed; ^1H NMR (400 MHz, $(CD_3)_2CO$) δ 9.83 (s, 1H), 7.20 (d, *J* = 4.2 Hz, 1H), 7.11 (d, *J* = 4.3 Hz, 1H); ^{13}C NMR (100 MHz, $(CD_3)_2SO$) δ 182.12, 134.70, 118.12, 111.23; ν_{max}/cm^{-1} 3418, 3127, 2890, 2853, 1680, 1279; HRMS (ESI) *m/z* $[M-H]^-$ calcd for $C_5H_3O_3N_2$ 139.0138; found 139.0138.

4-nitro-1*H*-pyrrole-2-carbaldehyde; R_f 0.3 [EtOAc-Hexane (3:7)]; m.p. 135-137 °C decomposed; ^1H NMR (400 MHz, $(CD_3)_2CO$) δ 12.05 (br. s, 1H), 9.69 (d, *J* = 1.1 Hz, 1H), 8.13_8.12 (m, 1H), 7.58 (d, *J* = 1.7 Hz, 1H); ^{13}C NMR (100 MHz, $(CD_3)_2SO$) δ 181.02, 138.98, 133.14, 126.01, 144.35; ν_{max}/cm^{-1} 3216, 3134, 2813, 2702, 1660, 1306; HRMS (ESI) *m/z* $[M-H]^-$ calcd for $C_5H_3O_3N_2$ 139.0138; found 139.0137.

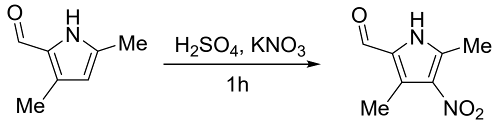

3,5-dimethyl-4-nitro-1*H*-pyrrole-2-carbaldehyde

3,5-Dimethyl-1H-pyrrole-2-carbaldehyde (0.22 g, 1.79 mmol) was added into conc. H_2SO_4 (10 mL) slowly at -20 °C, and the mixture was stirred until it become homogeneous. Subsequently, KNO_3 (0.198 g, 1.96 mmol) was added in portion below -10 °C. The mixture was stirred at -10 °C for 20 min and then stirred at room temperature for another 20 min. After completion, cold H_2O (20 mL) was added in portions. The parcipitate was filtered washed with H2O (20 mL), and dried over night to gave 3,5-dimethyl-4-nitro-1*H*-pyrrole-2-carbaldehyde (0.178 g, 1.06 mmol, 59%) as a brown solid; m.p. decomposed; ^1H NMR (400 MHz, $(CD_3)_2SO$) δ 12.79 (br. s, 1H), 9.73 (s, 1H), 2.55 (s, 3H), 2.54 (s, 3H) Data were in agreement to those reported in the literature.[8]

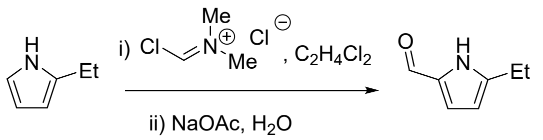

5-ethyl-1*H*-pyrrole-2-carbaldehyde

To a solution of 2-ethylpyrrole (0.3 mL, 2.93 mmol) in $C_2H_4Cl_2$ (10 mL) was added (Chloromethylene)dimethyliminium chloride (0.451 g, 3.52 mmol) at 0 °C. The reaction mixture was allowed to warm to room temperature 15 min. The reaction mixture was heated to 85 °C and stirred for 30 min. The mixture was cooled to room temperature, a solution of NaOAc (1.32 g, 16.11 mmol) in H_2O (10 mL) was added. The mixture was heated to 85 °C and stirred for 30 min. The mixture was wash with H_2O (10 mL), saturated Na_2CO_3 (2 x 10 mL), and brine (10 mL). The organic layer was dried (Na_2SO_4), filtered, and the solvent was evaporated. Purification by flash column chromatography, eluted with EtOAc-Hexane (1:9 to 3:7), gave 5-

ethyl-1*H*-pyrrole-2-carbaldehyde (0.331 g, 2.69 mmol, 92%) as a yellow solid; R_f 0.6 [EtOAc-Hexane (3:7)]; m.p. 38–41 °C; ^{1}H NMR (400 MHz, $CDCl_3$) δ 10.18 (br. s, 1H), 9.36 (s, 1H), 6.92 (dd, *J* = 3.8, 2.4 Hz, 1H), 6.10–6.09 (m, 1H), 2.76 (q, *J* = 7.6 Hz, 2H), 1.30 (t, *J* = 7.6 Hz, 3H); ^{13}C NMR (100 MHz, $CDCl_3$) δ 178.66, 145.42, 132.31, 123.51, 109.27, 21.54, 13.54; ν_{max}/cm^{-1} 3252, 3128, 2880, 2818, 1630, 1495; HRMS (ESI) *m/z* $[M+H]^+$ calcd for $C_7H_{10}ON$ 124.0757; found 124.0759.

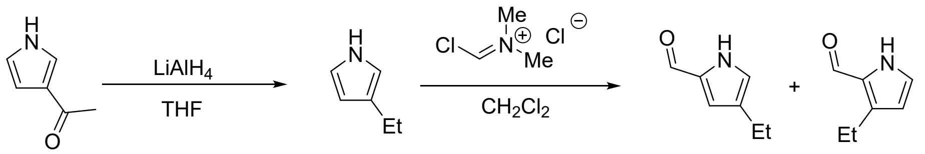

3-ethyl-1*H*-pyrrole

To a solution of $LiAlH_4$ (0.572 g, 15.08 mmol) in anhydrous THF (10 mL) was added 3-acetylpyrrole (0.822 g, 7.53 mmol) at 0 °C. The reaction mixture was allowed to warm to room temperature 15 min. The reaction mixture was heated to 72 °C and stirred for 17 h. The reaction was cooled to room temperature and quenched with a few drops of EtOAc followed by MeOH and H_2O until no bubbles were released. The mixture was extracted by CH_2Cl_2 (3 x 20 mL). The combined organic layers were dried (Na_2SO_4), filtered, and the solvent was evaporated. Purification by flash column chromatography, eluted with Et_2O-Pentane (3:7), gave 3-ethyl-1*H*-pyrrole (0.544 g) as colourless oil. The compounds were used in the next step without characterisation.

4-ethyl-1*H*-pyrrole-2-carbaldehyde and **3-ethyl-1*H*-pyrrole-2-carbaldehyde**

To a solution of 3-ethyl-1*H*-pyrrole (0.544 g, 5.72 mmol) in CH_2Cl_2 (10 mL) was added (Chloromethylene)dimethyliminium chloride (1.46 g, 11.44 mmol) at 0 °C. The reaction mixture was allowed to warm to room temperature 15 min. The mixture was stirred at room temperature for 16 h. The solvent was evaporated, the residue was added H_2O (10 mL) followed by NaOH (1.83 g, 45.76 mmol) and the mixture was stirred at room temperature for 1 h. EtOAc (20 mL) was added, the layers were separated and the aqueous layer was extracted by EtOAc (2 x 20 mL). The combined organic layers were dried (Na_2SO_4), filtered, and the solvent was evaporated. Purification by flash column chromatography, eluted with EtOAc-Hexane (1:9 to 3:7), gave 4-ethyl-1*H*-pyrrole-2-carbaldehyde (0.130 g, 1.06 mmol, 19%) as a yellow oil and 3-ethyl-1*H*-pyrrole-2-carbaldehyde (0.297 g, 2.41 mmol, 42%) as a yellow oil.

4-Ethyl-1*H*-pyrrole-2-carbaldehyde; R_f 0.5 [EtOAc-Hexane (3:7)]; ^{1}H NMR (300 MHz, $CDCl_3$) δ 9.45 (s, 1H), 6.93 (~t, 1H), 6.82 (t, *J* = 2.2 Hz, 1H), 2.54 (q, *J* = 7.6 Hz, 2H), 1.21 (t, *J* = 7.6 Hz, 3H) Data were in agreement to those reported in the literature.[9]

3-Ethyl-1*H*-pyrrole-2-carbaldehyde; R_f 0.5 [EtOAc-Hexane (3:7)]; ^{1}H NMR (300 MHz, $CDCl_3$) δ 9.64 (s, 1H), 9.34 (br. S, 1H), 7.02 (~t, 1H), 6.19j (t, *J* = 2.6 Hz, 1H), 2.82 (q, *J* = 7.6 Hz, 2H), 1.28 (t, *J* = 7.6 Hz, 3H) Data were in agreement to those reported in the literature.[9]

References

[1] M. A. Buil, M. Calbet, M. Castillo, J. Castro, C. Esteve, M. Ferrer, P. Forns, J. González, S. López, R. S. Roberts, S. Sevilla, B. Vidal, L. Vidal, P. Vilaseca, *Eur. J. Med. Chem.* **2016**, *113*, 102–103.

[2] J. K. Laha, S. Sharma, S. Kira, U. C. Banerjee, *J. Org. Chem.* **2017**, *82*, 9350–9359.

[3] T. Warashina, D. Matsuura, T. Sengoku, M. Takahashi, H. Yoda, Y. Kimura, *Org. Process Res. Dev.* **2019**, *23*, 614–618.

[4] K. C. Nguyen, P. Wang, R. D. Sommer, J. S. Lindsey, *J. Org. Chem.* **2020**, *85*, 6605–6619.

[5] Y.-Z. Jin, D.-X. Fu, N. Ma, Z.-C. Li, Q.-H. Liu, L. Xiao, R.-H. Zhang, *Molecules.* **2011**, *16*, 9368–9385.

[6] H. Jing, P. Wang, B. Chen, J. Jiang, P. Vairaprakash, S. Liu, J. Rong, C.-Y, Chen, P. Nalaoh, J. S. Lindsey, *New J. Chem.* **2022**, *46*, 5534–5555.

[7] C. Schmuck, V. Bickert, M. Merschky, L. Geiger, D. Rupprecht, J. Dudaczek, P. Wich, T. Rehm, U. Machon, *Eur. J. Org. Chem.* **2008**, *2*, 324–329.

[8] Q. Li, X. Pan, D. Wang, Q. Rong, B. Ma, X. Xie, Y. Zhang, J. Wang, L. Hu, *J. Med. Chem.* **2021**, *64*, 17184–17208.

[9] A. Sailer, J. C. M. Meiring, C. Heise, L. N. Pettersson, A. Akhmanova, J. Thorn-Seshold, O. Thorn-Seshold, *Angew. Chem. Int. Ed.* **2021**, *60*, 23695–23704.

1H and 13C spectra

4-bromo-1*H*-pyrrole-2-carbaldehyde spectra in $CDCl_3$

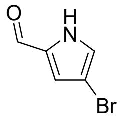

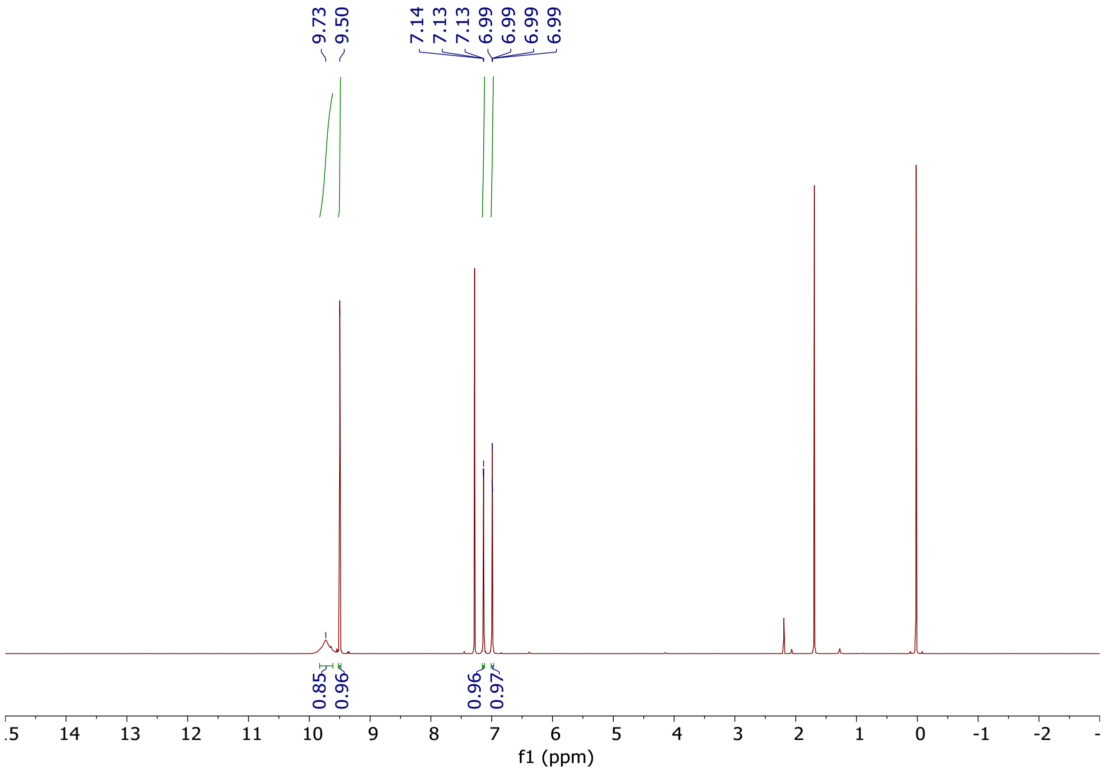

4,5-dibromo-1*H*-pyrrole-2-carbaldehyde spectra in $CDCl_3$

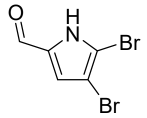

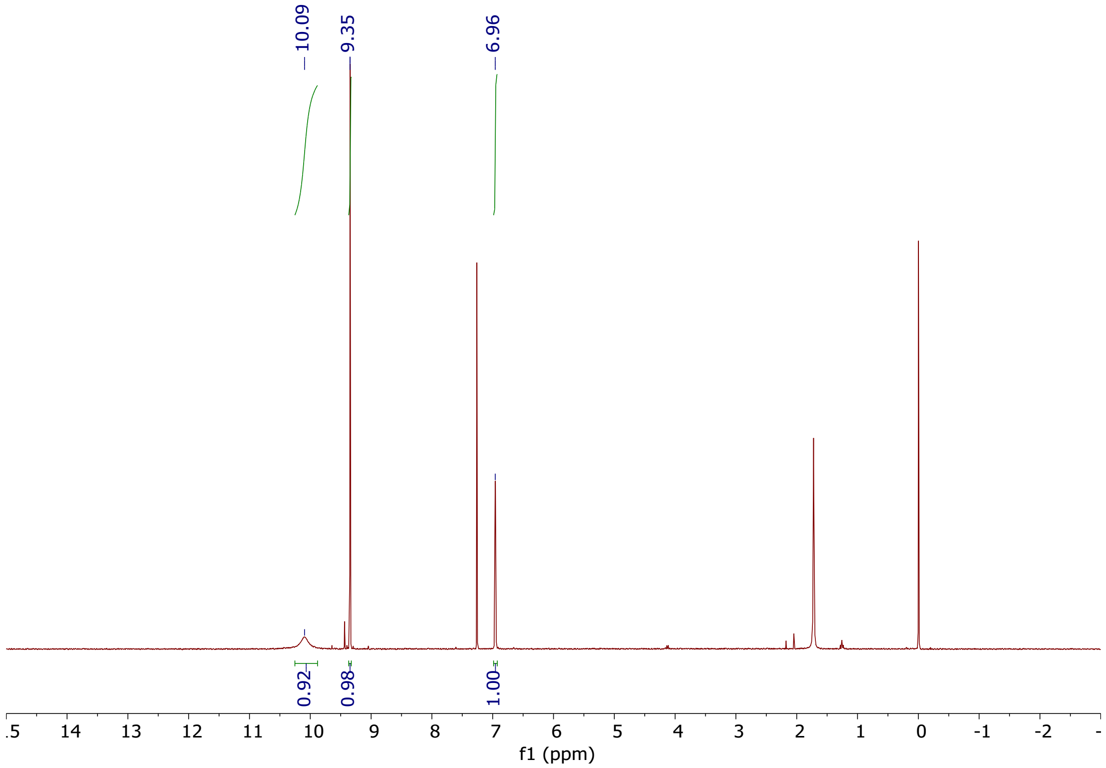

Ethyl 5-formyl-1*H*-pyrrole-2-carboxylate spectra in $CDCl_3$

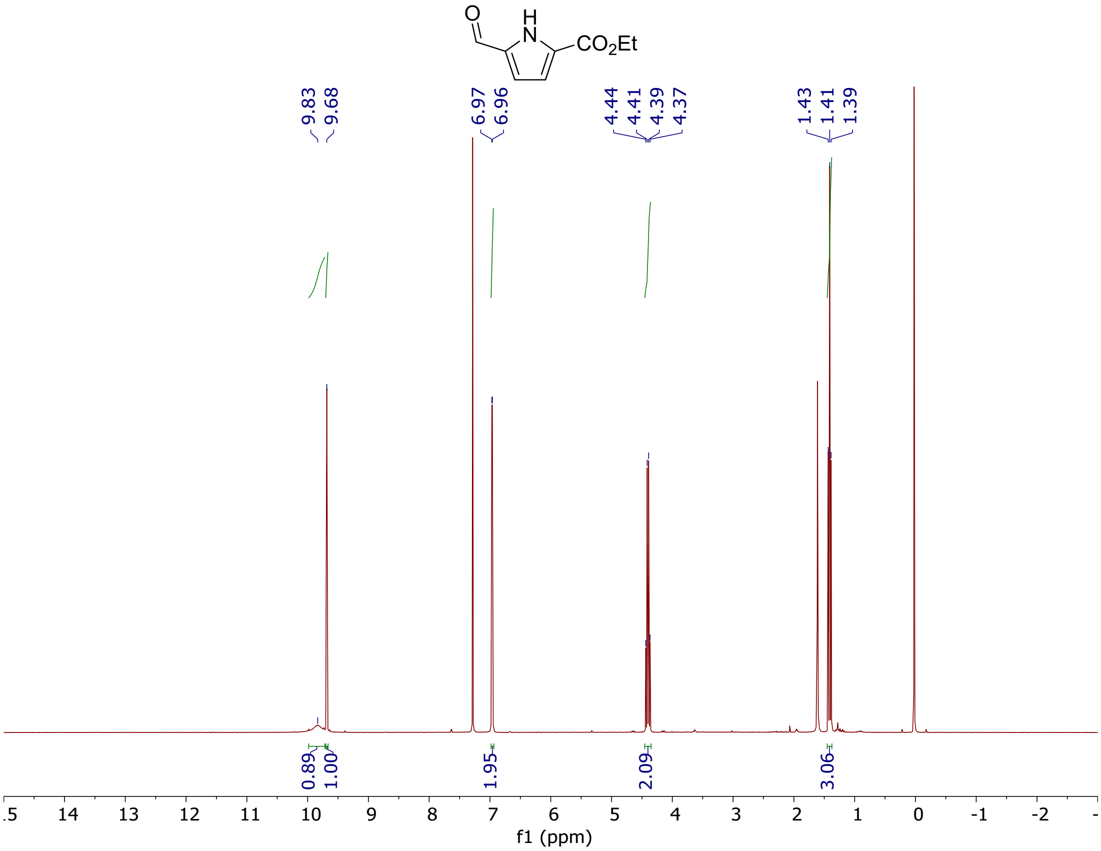

Ethyl 4-formyl-1*H*-pyrrole-2-carboxylate spectra in $CDCl_3$

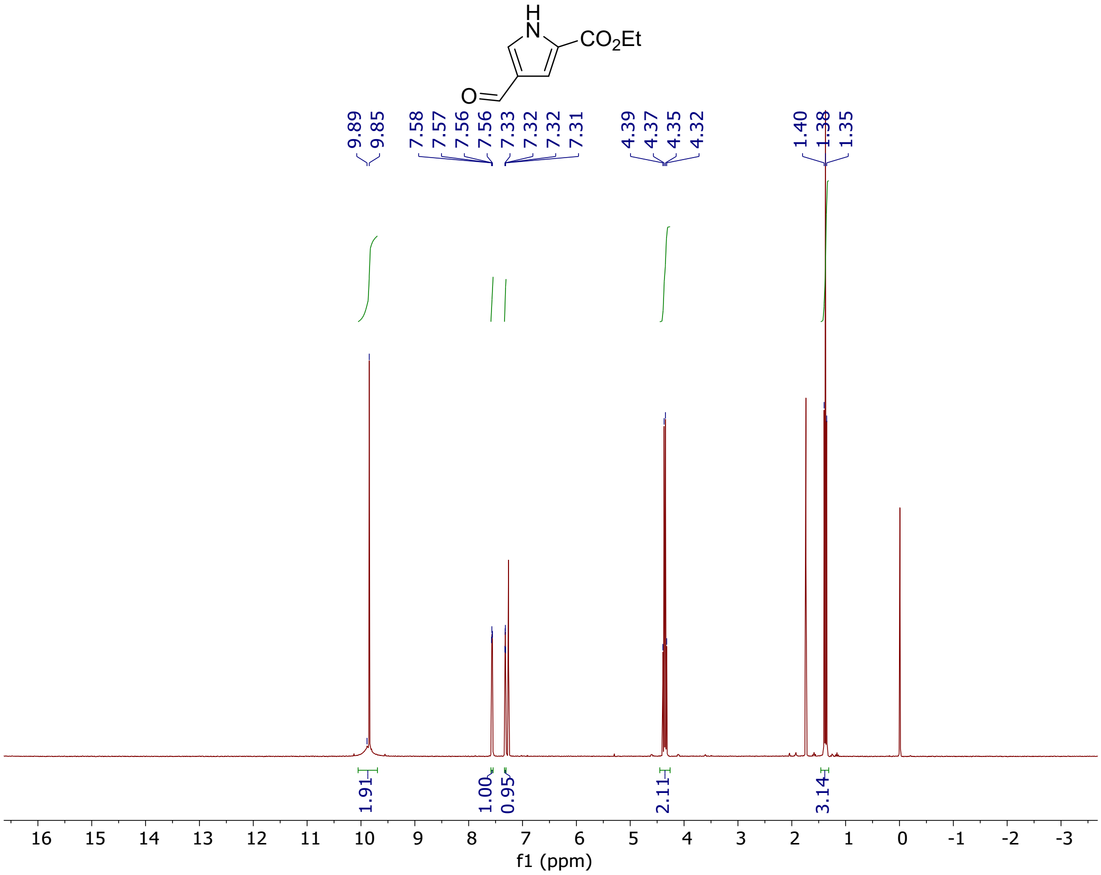

Ethyl 1*H*-pyrrole-3-carboxylate spectra in $CDCl_3$

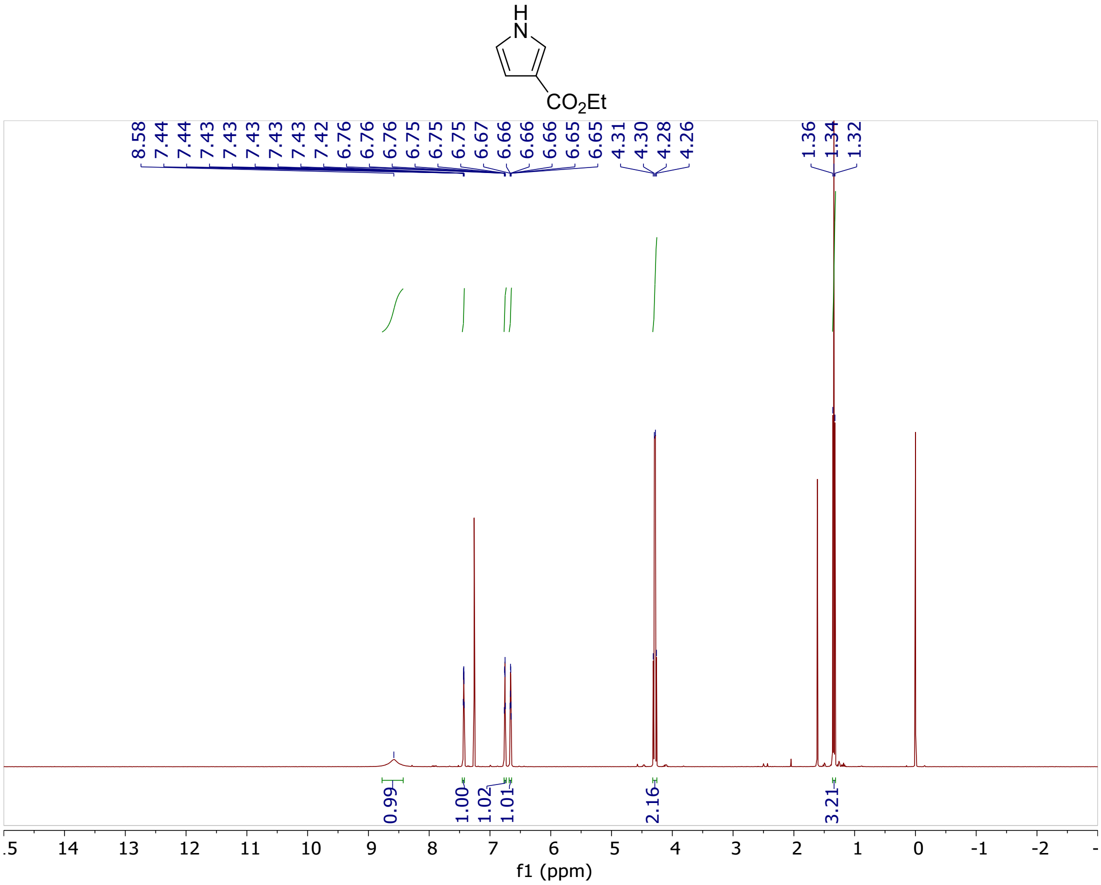

Ethyl 5-formyl-1*H*-pyrrole-3-carboxylate spectra in $CDCl_3$

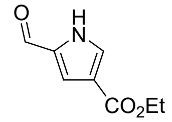

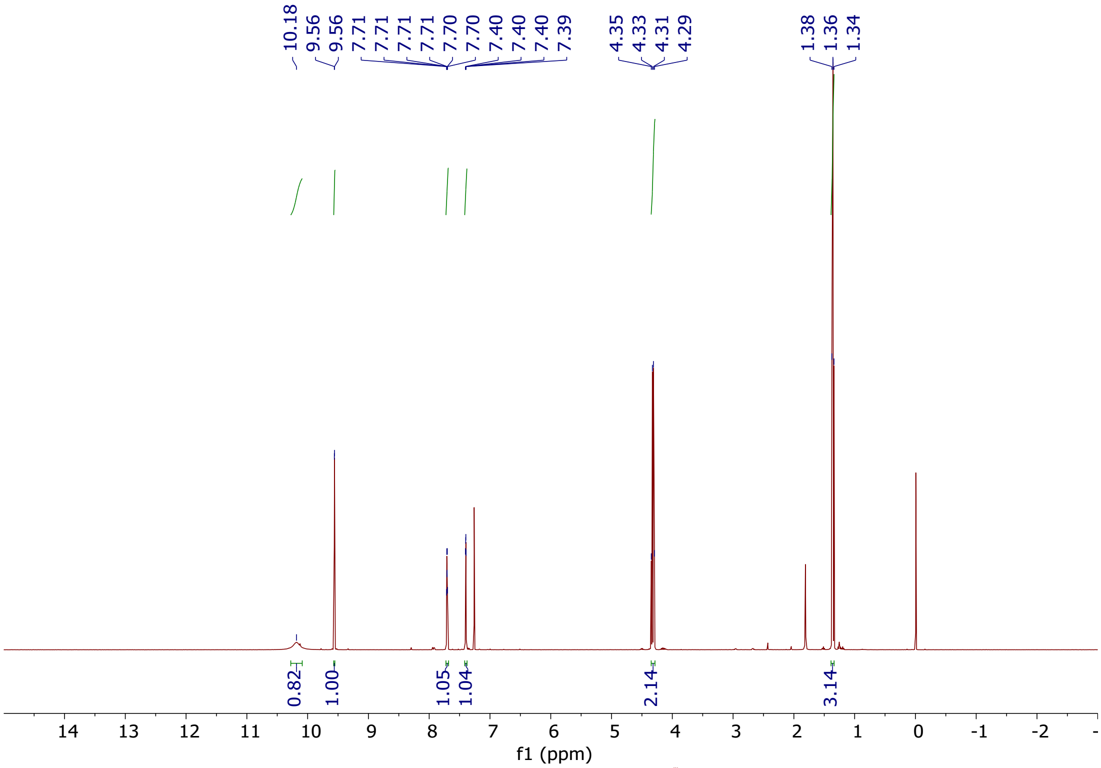

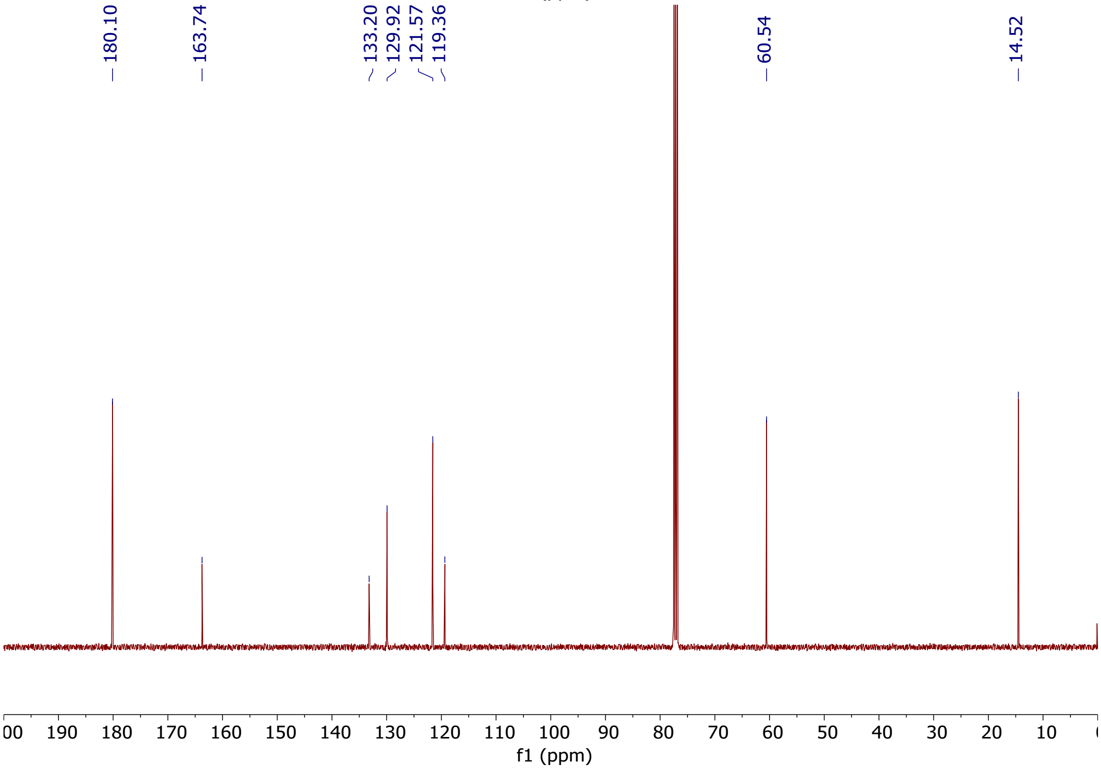

Ethyl 2-formyl-1*H*-pyrrole-3-carboxylate spectra in $CDCl_3$

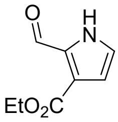

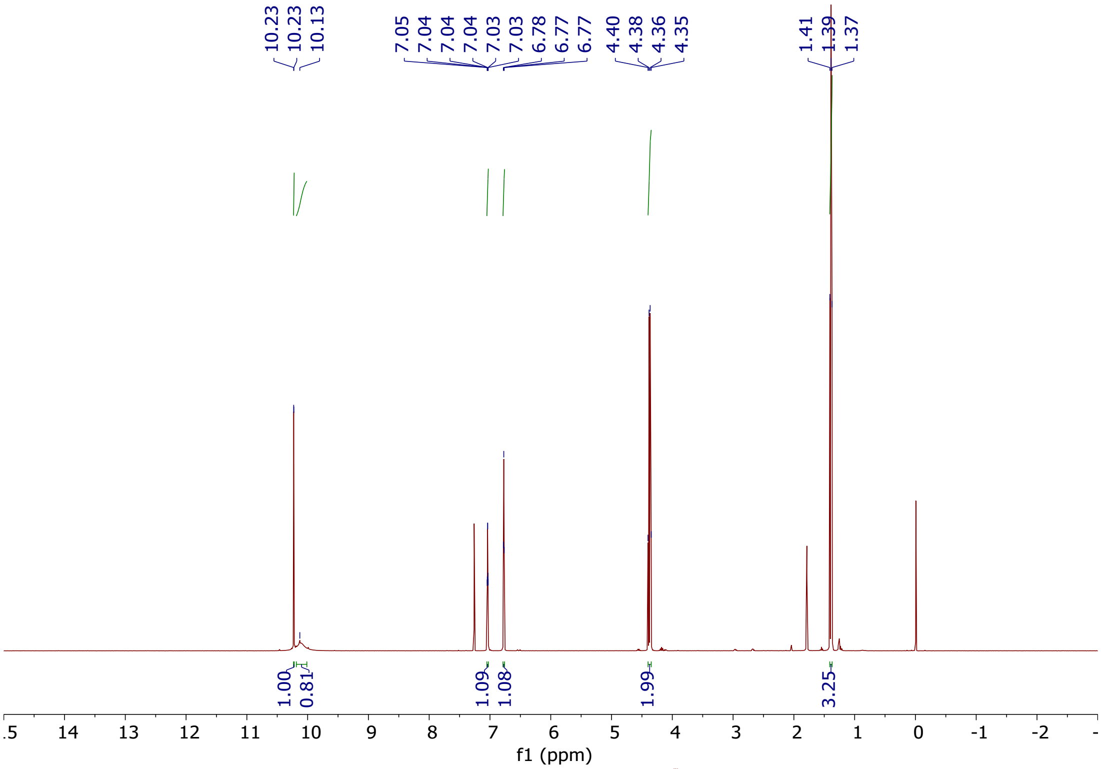

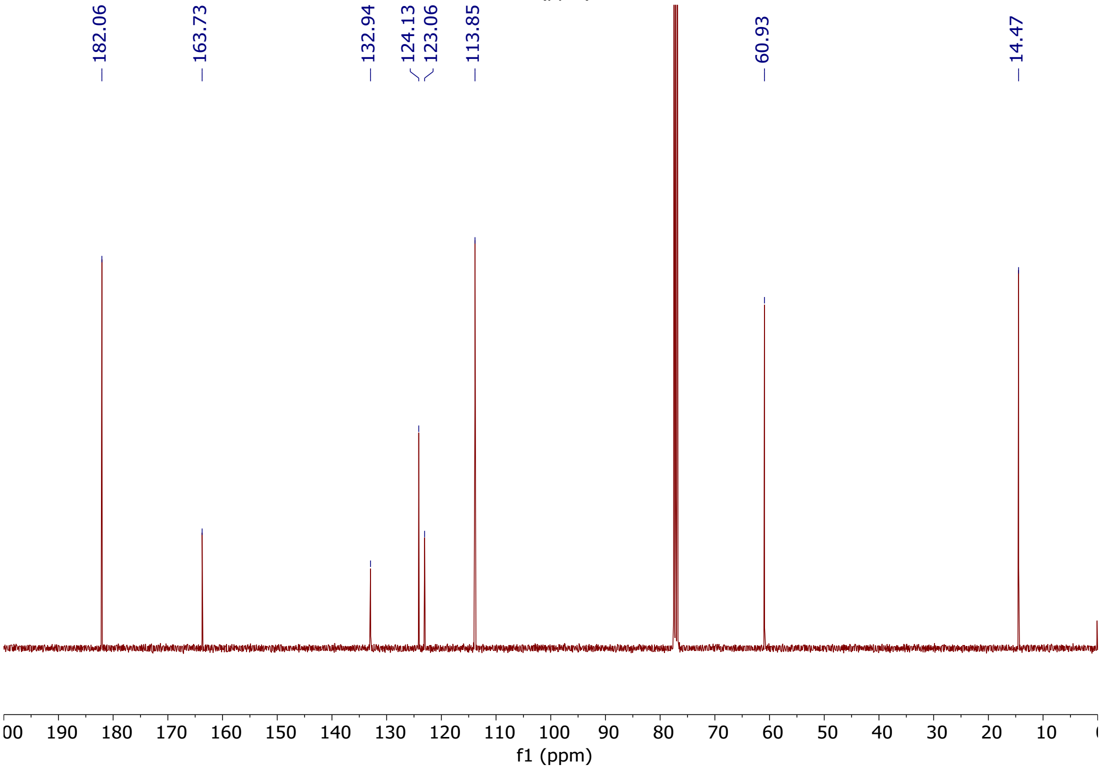

5-formyl-1*H*-pyrrole-2-carboxylic acid spectra in dmso-d_6

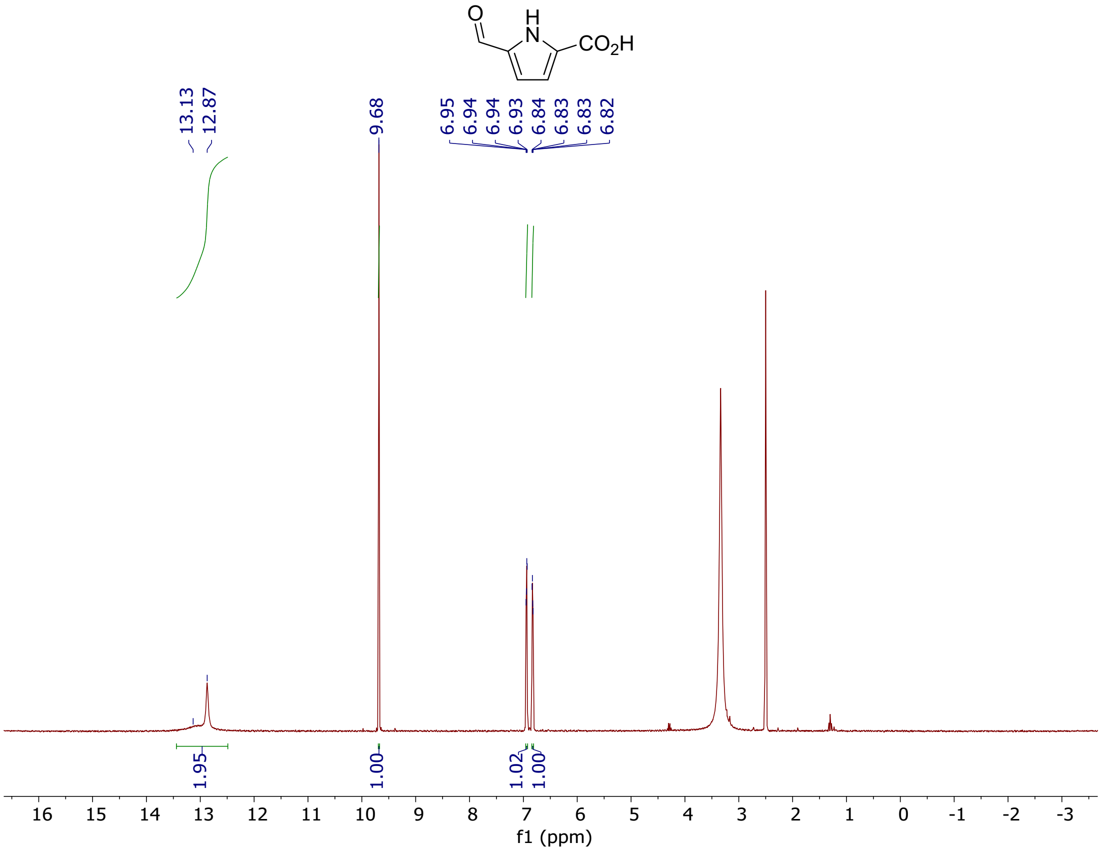

4-formyl-1*H*-pyrrole-2-carboxylic acid spectra in dmso-d$_6$

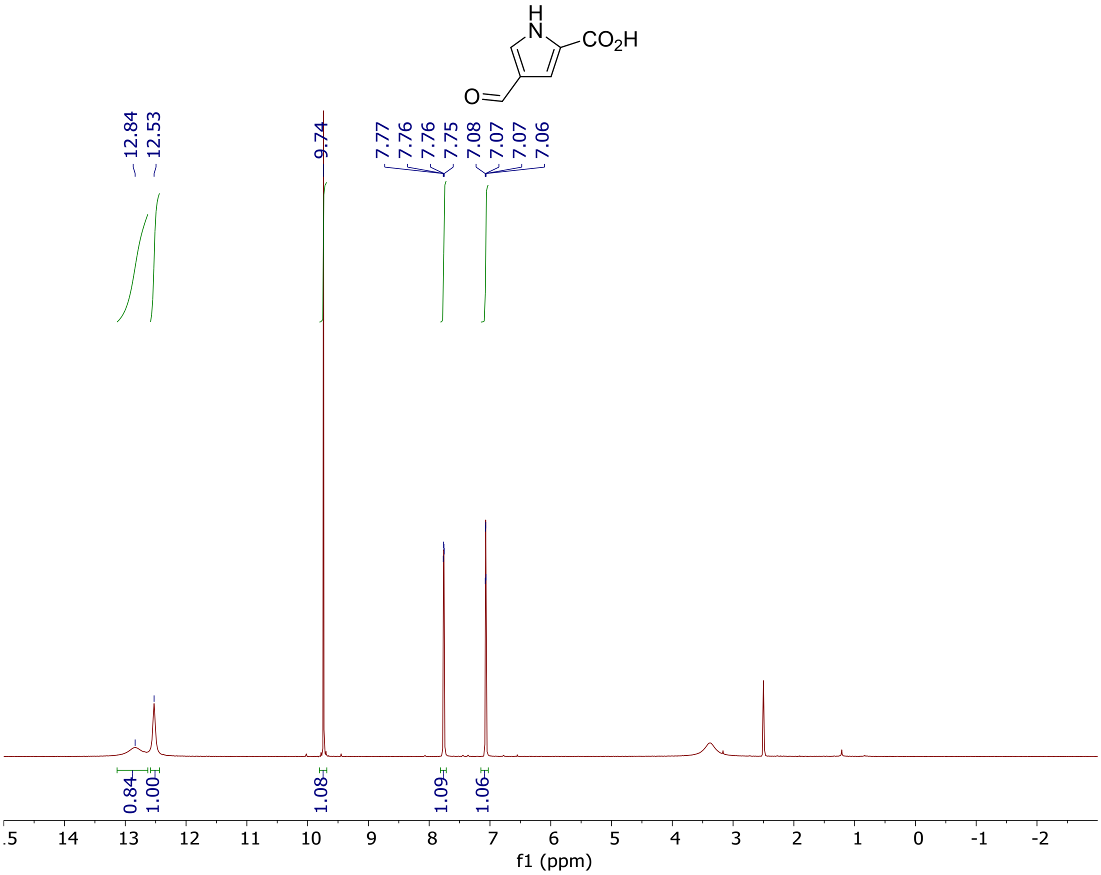

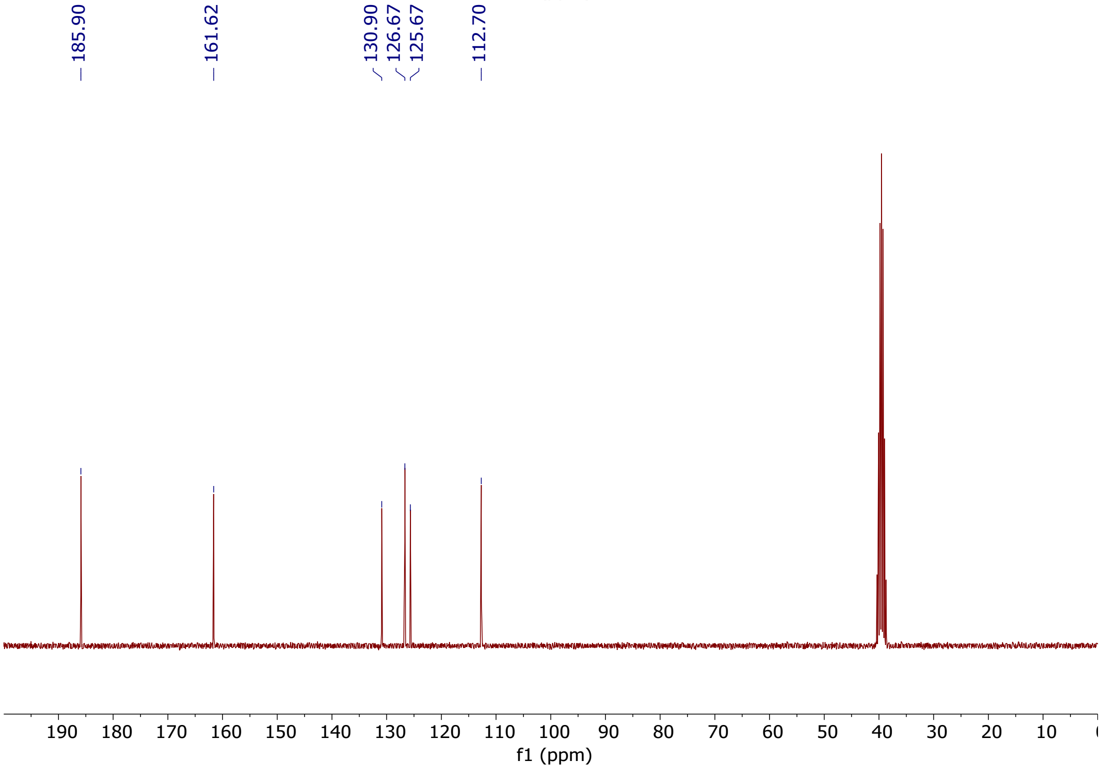

5-formyl-1*H*-pyrrole-3-carboxylic acid spectra in dmso-d_6

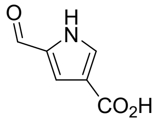

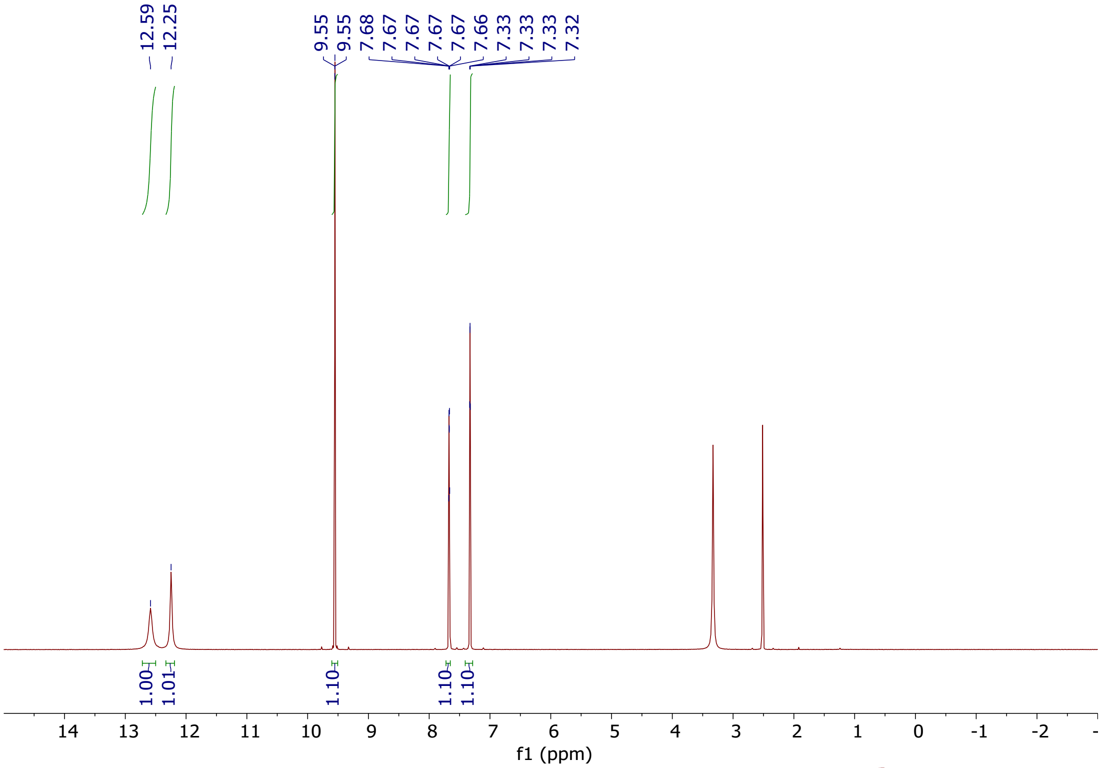

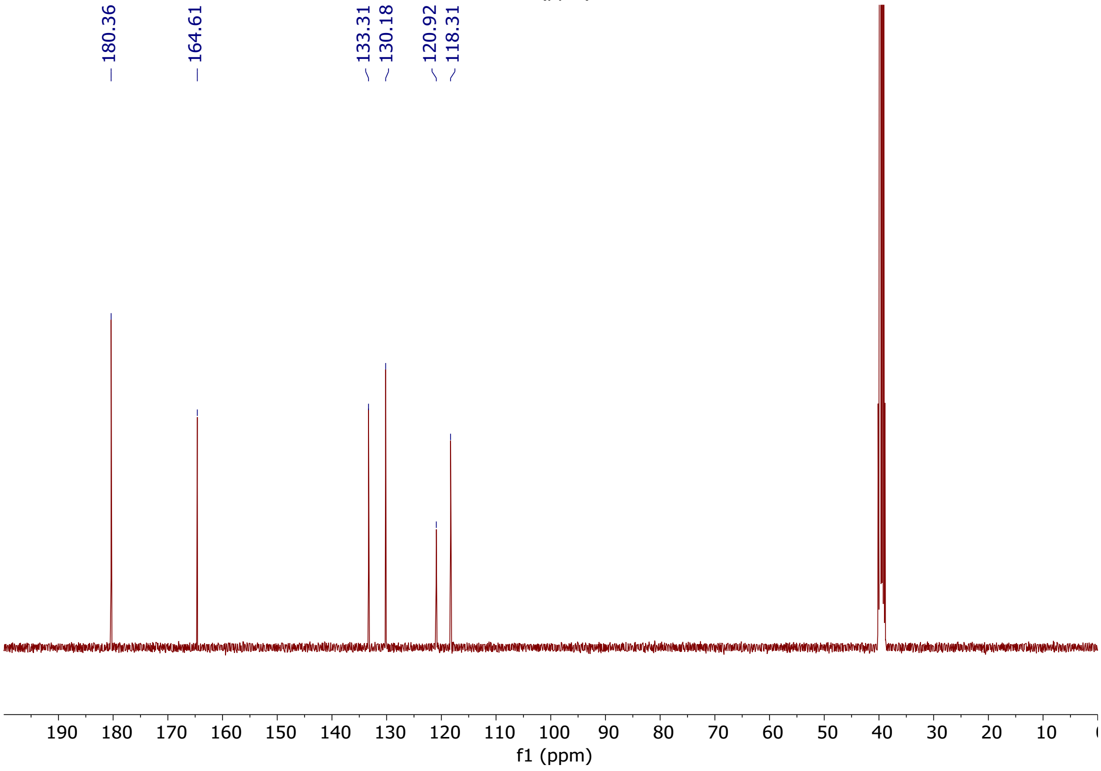

5-(morpholine-4-carbonyl)-1*H*-pyrrole-2-carbaldehyde spectra in dmso-d_6

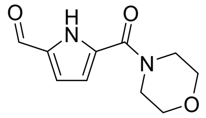

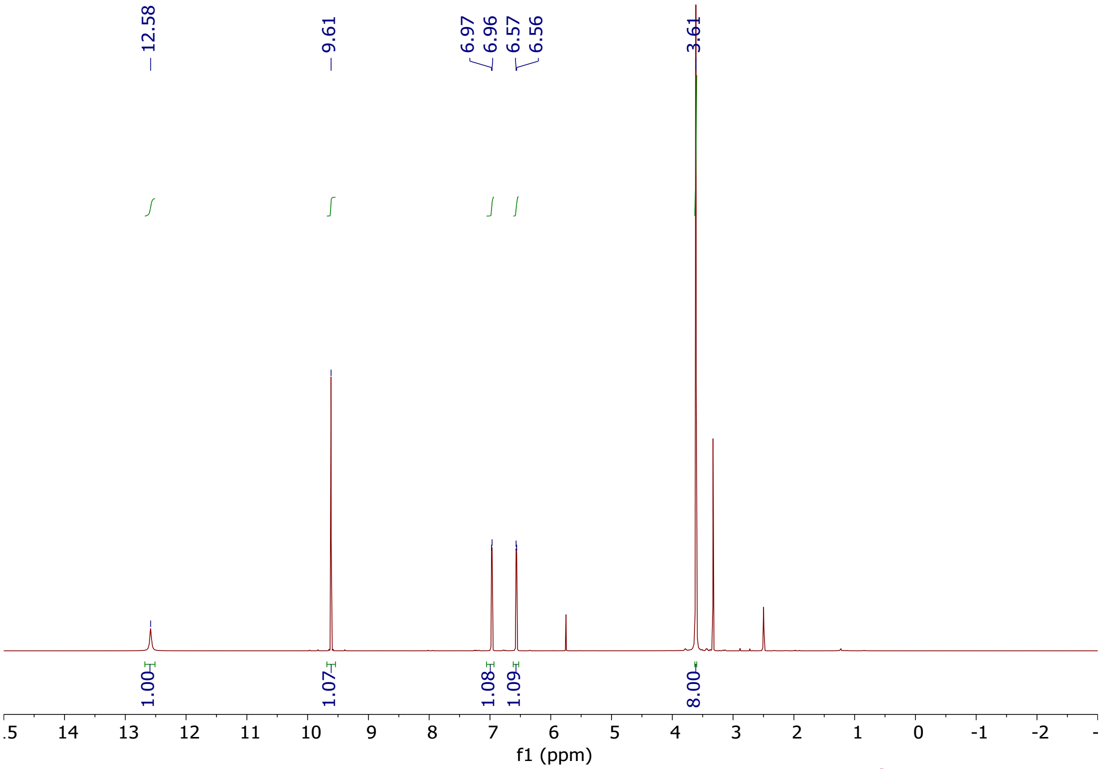

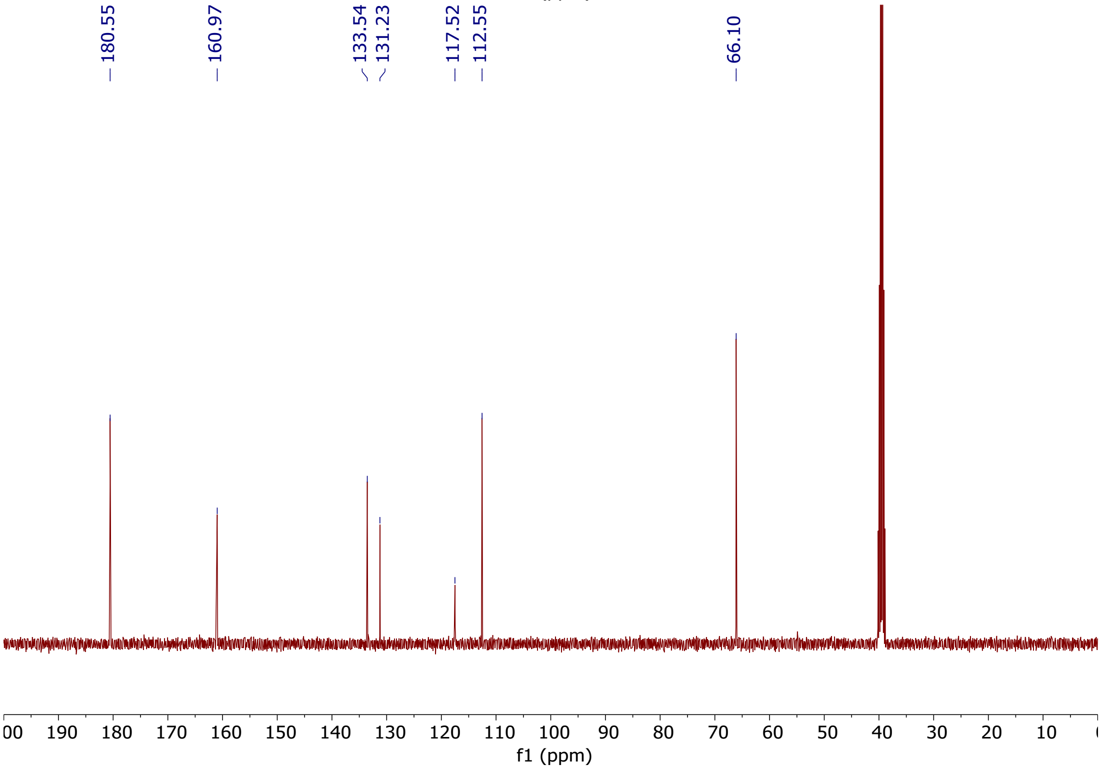

5-(morpholine-4-carbonyl)-1*H*-pyrrole-3-carbaldehyde spectra in $CDCl_3$

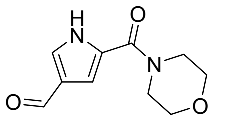

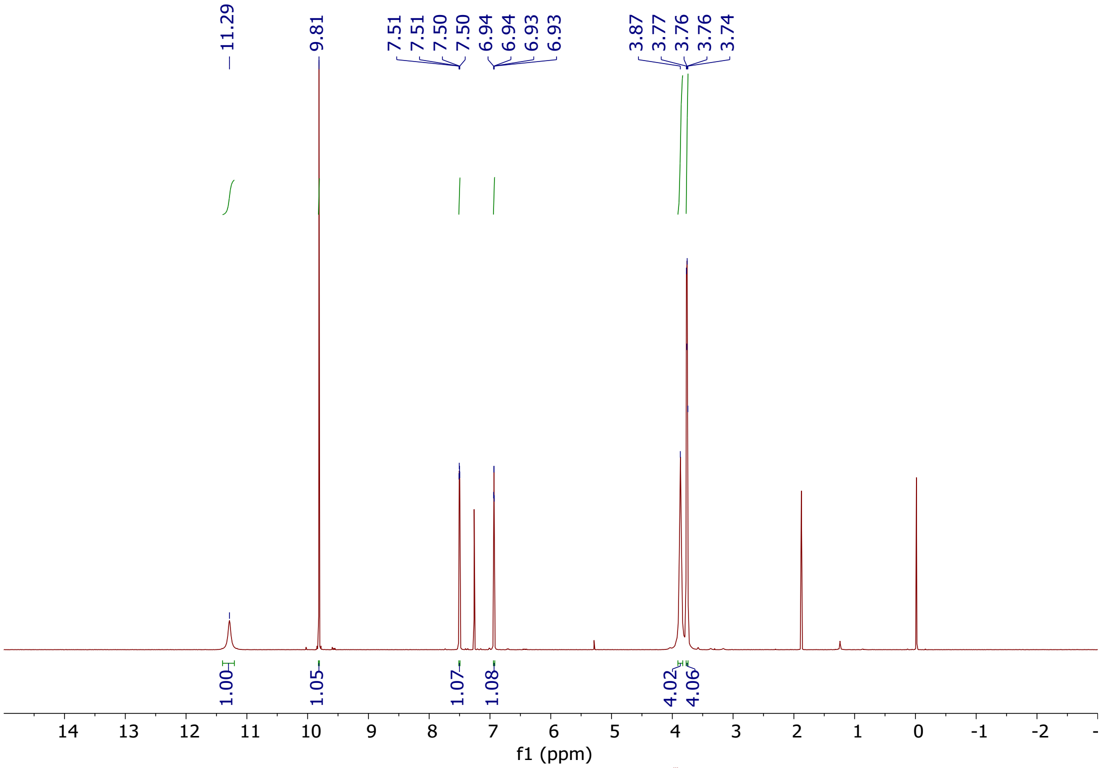

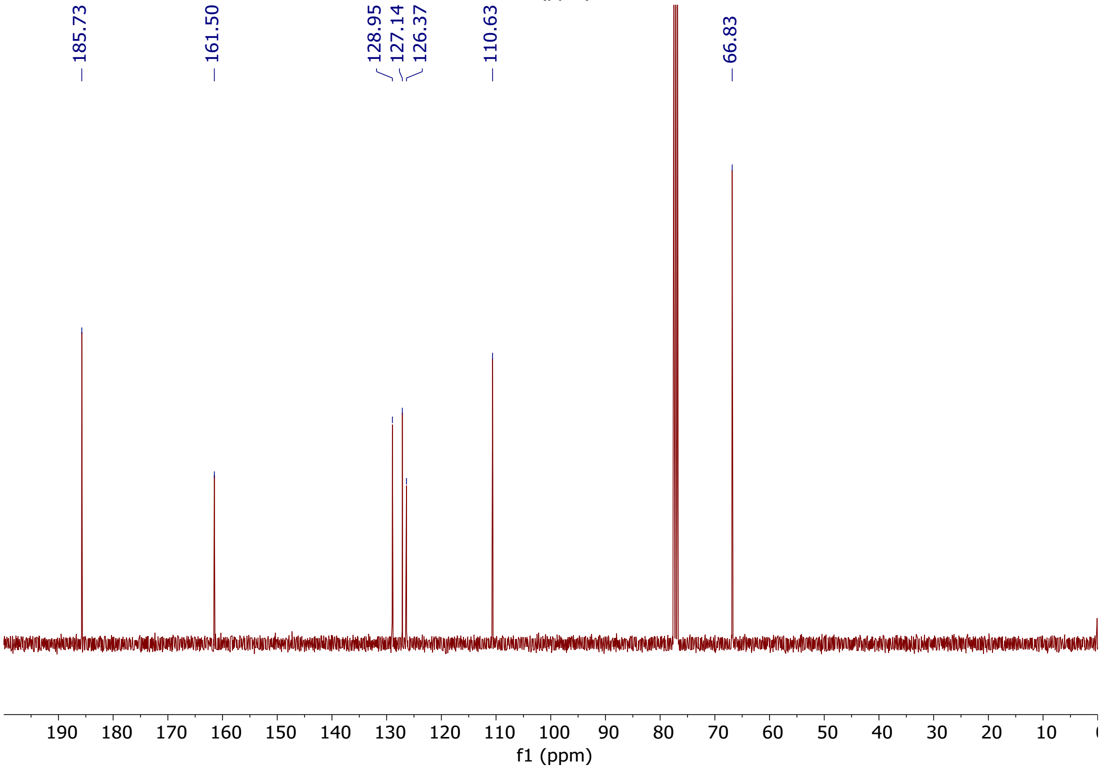

4-(morpholine-4-carbonyl)-1*H*-pyrrole-2-carbaldehyde spectra in dmso-d_6

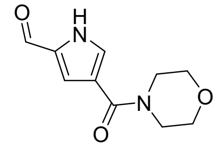

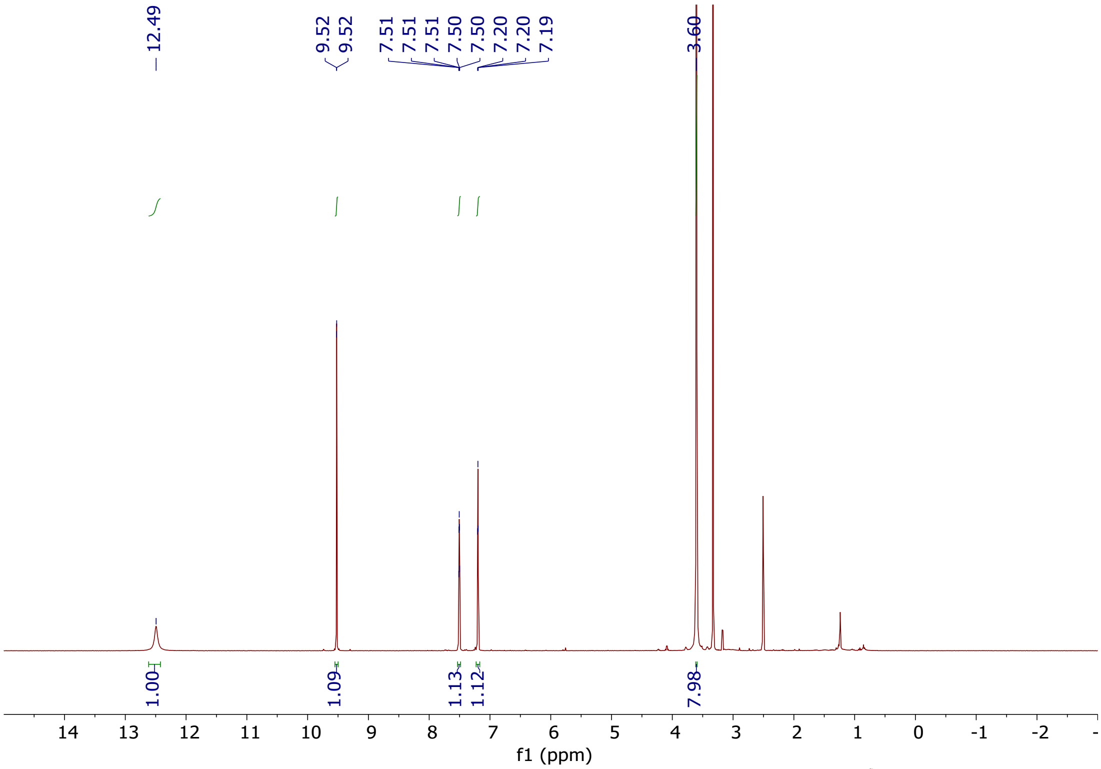

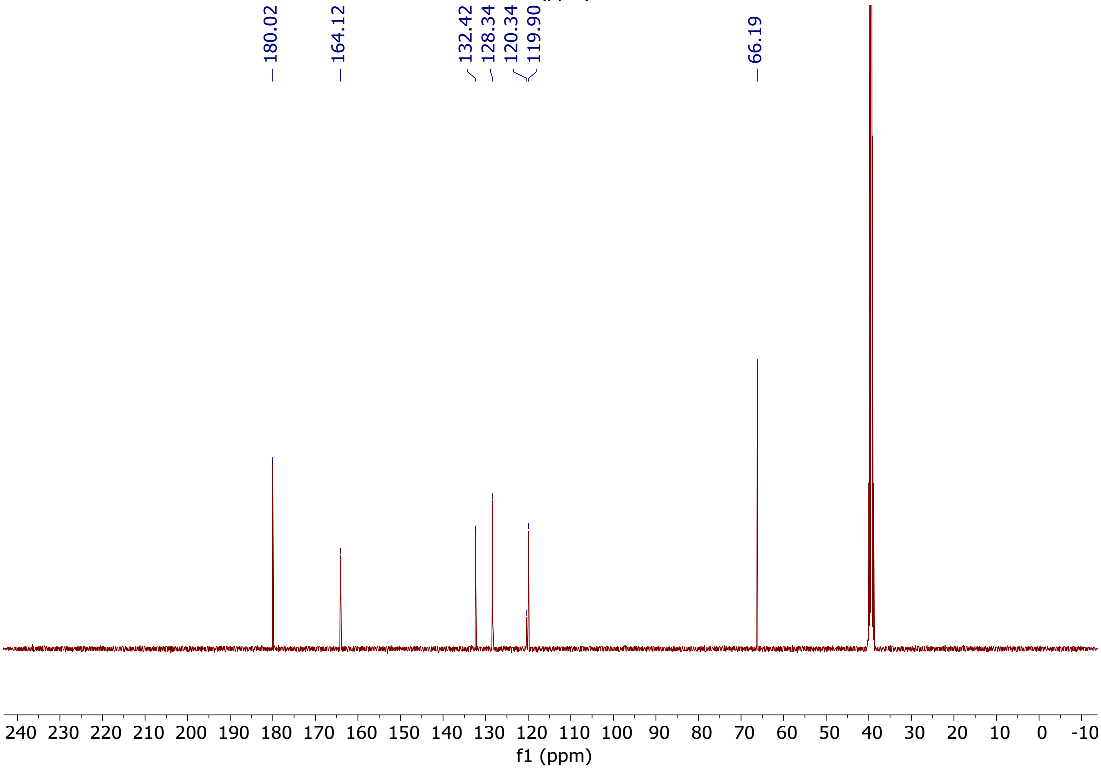

5-nitro-1*H*-pyrrole-2-carbaldehyde spectra in acetone-d_6

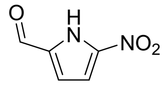

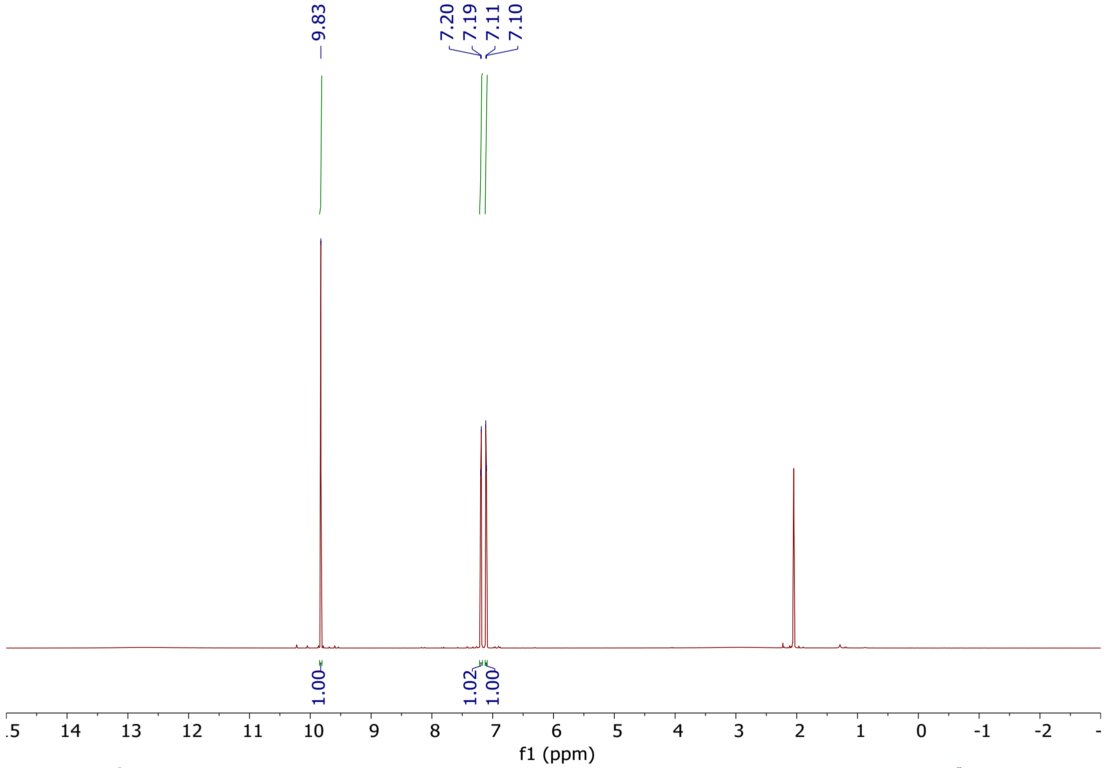

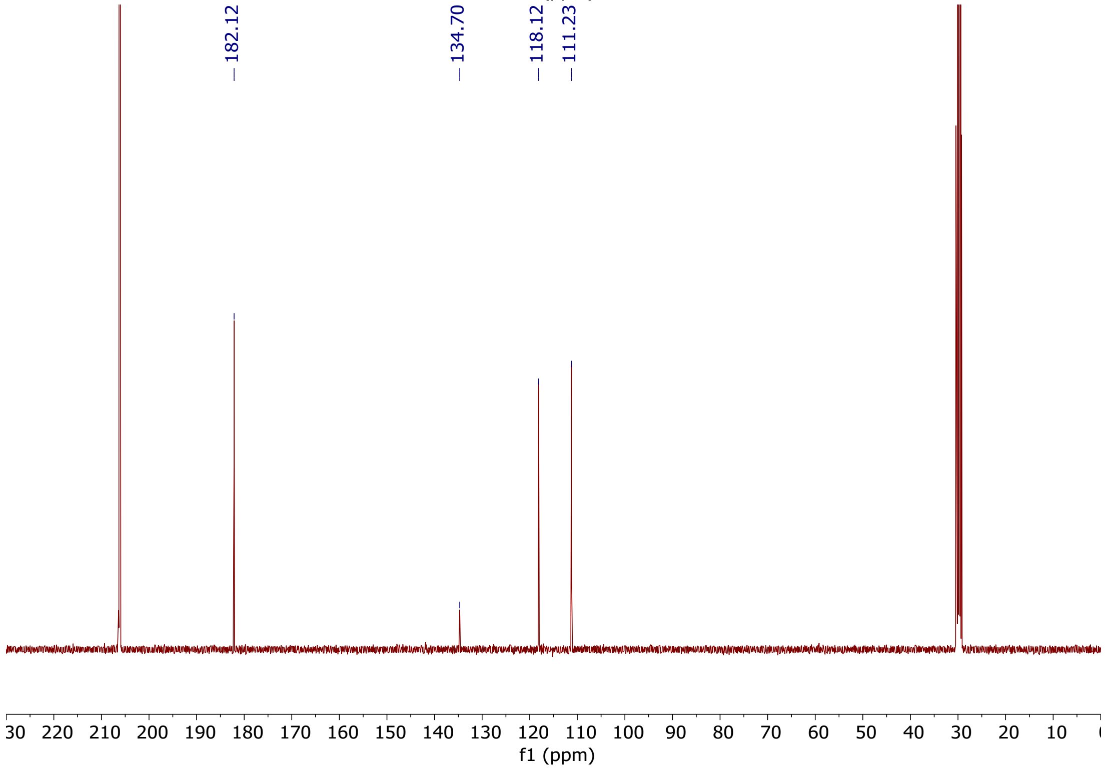

4-nitro-1*H*-pyrrole-2-carbaldehyde spectra in acetone-d_6

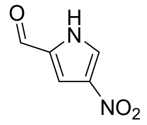

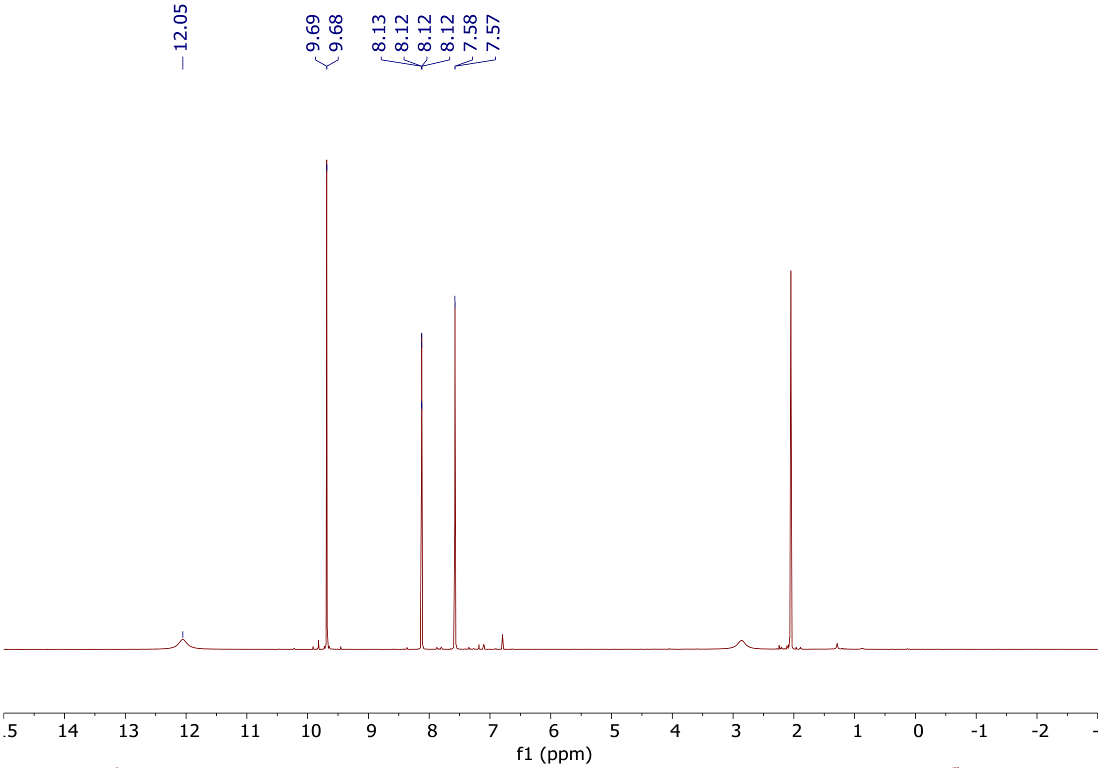

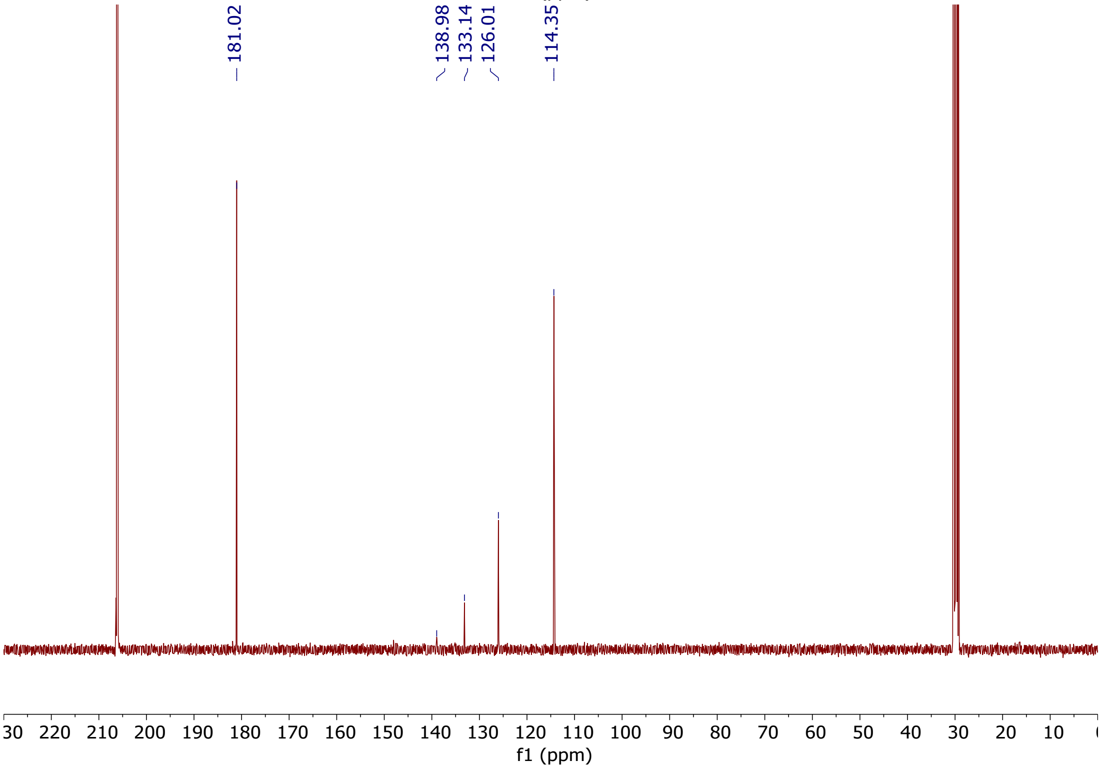

3,5-dimethyl-4-nitro-1*H*-pyrrole-2-carbaldehyde spectra in dmso-d_6

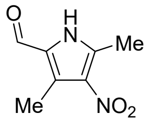

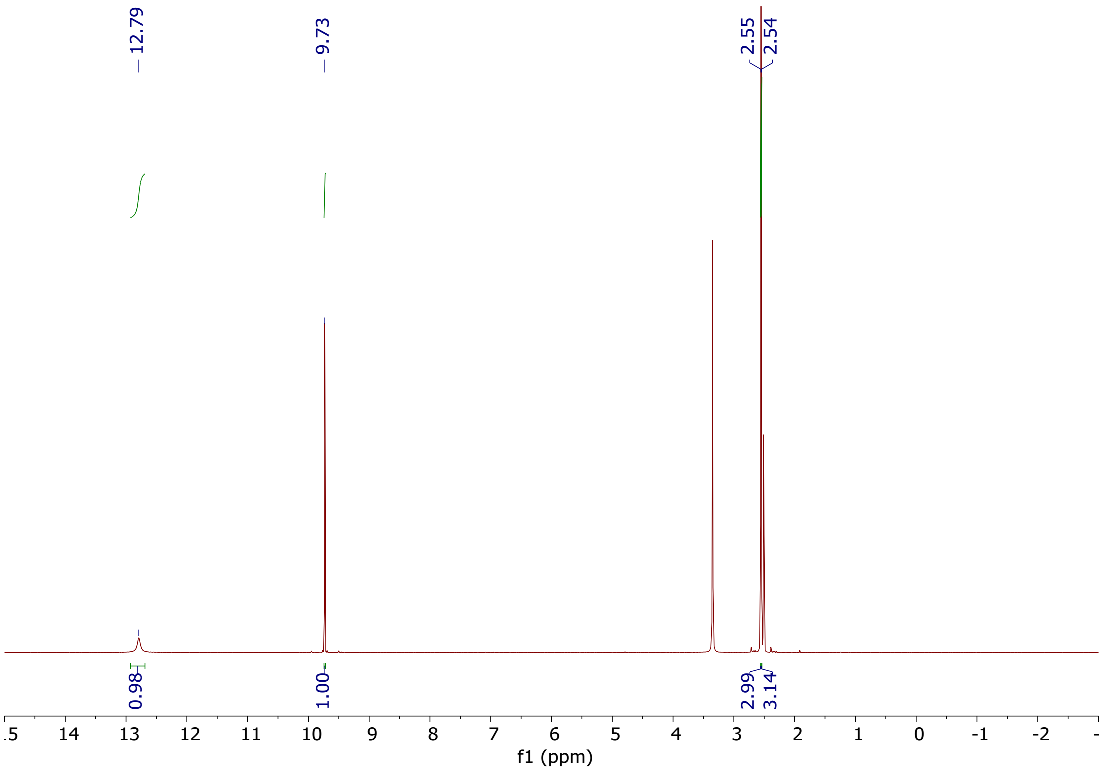

5-ethyl-1*H*-pyrrole-2-carbaldehyde spectra in $CDCl_3$

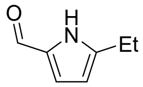

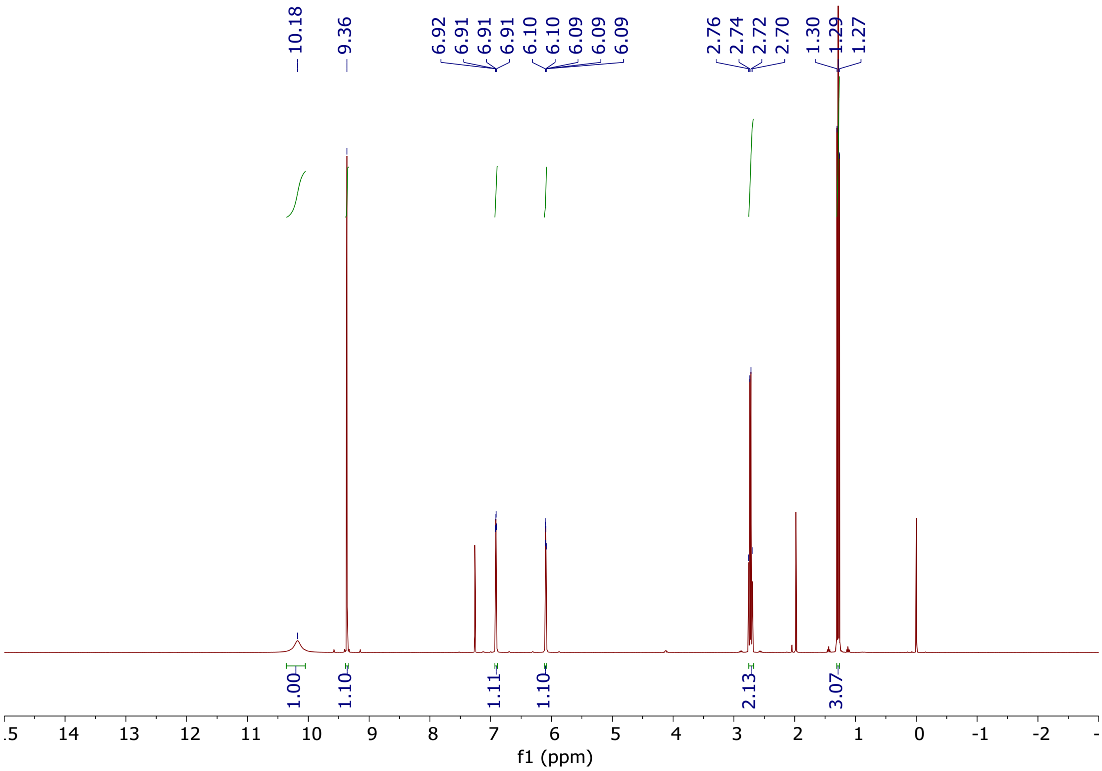

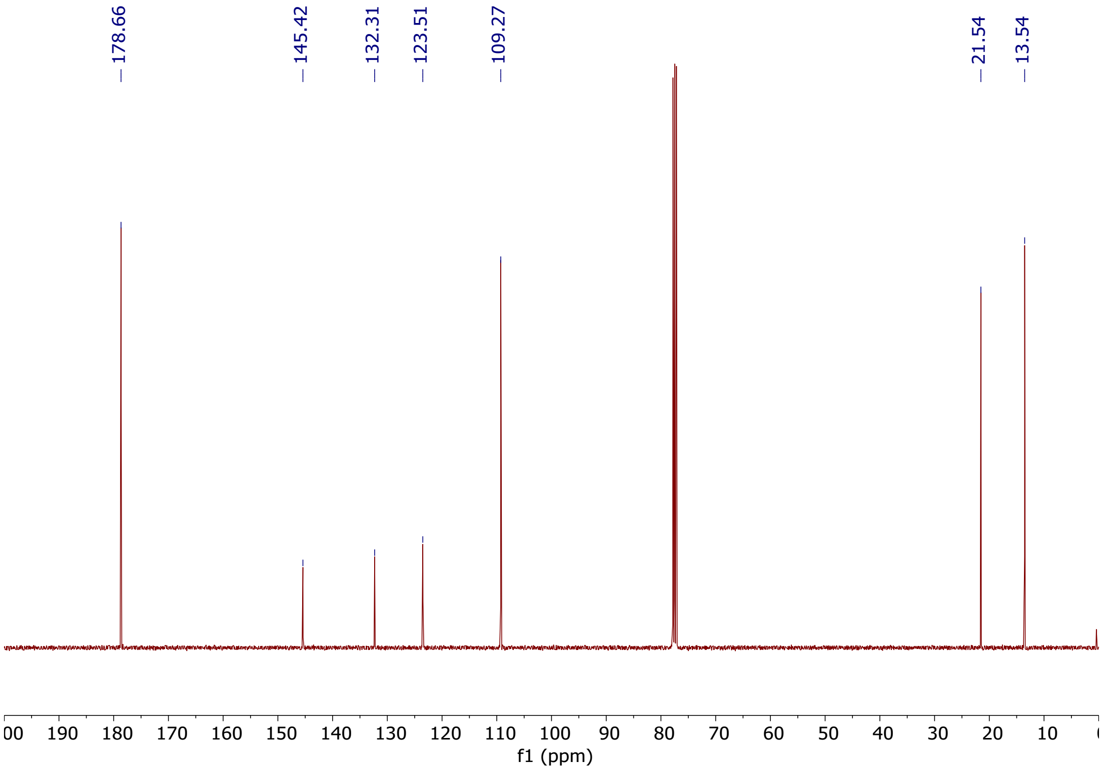

4-ethyl-1*H*-pyrrole-2-carbaldehyde spectra in $CDCl_3$

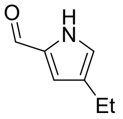

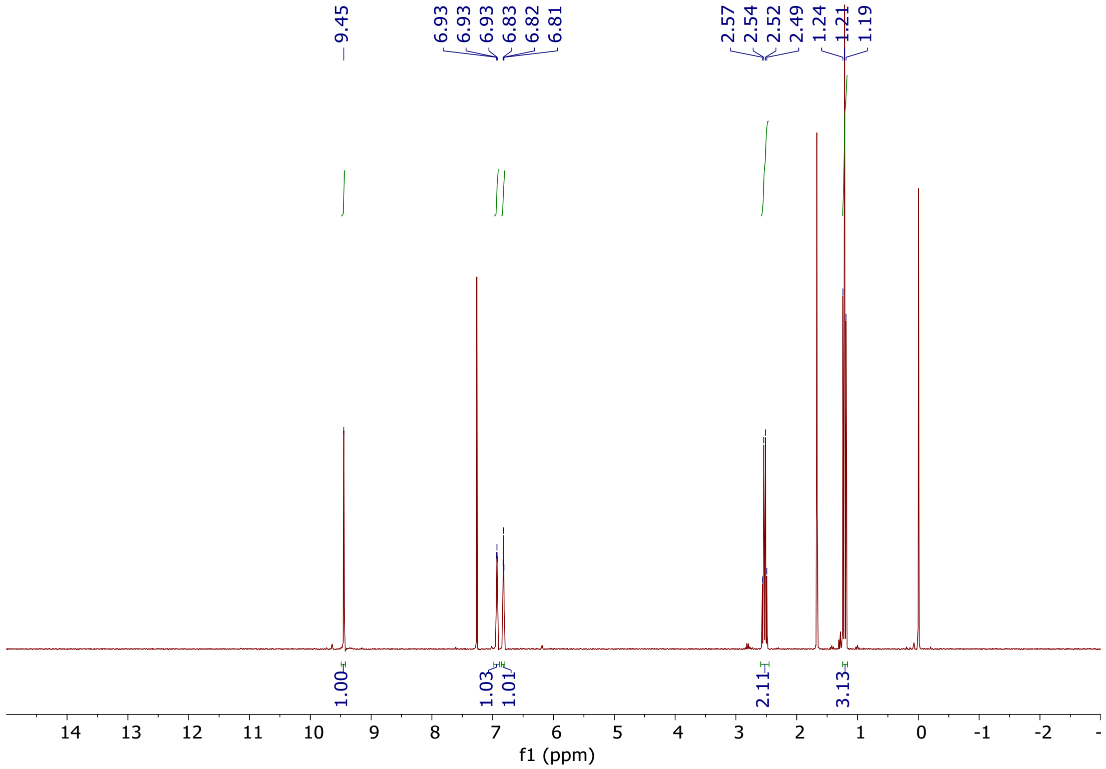

3-ethyl-1*H*-pyrrole-2-carbaldehyde spectra in $CDCl_3$

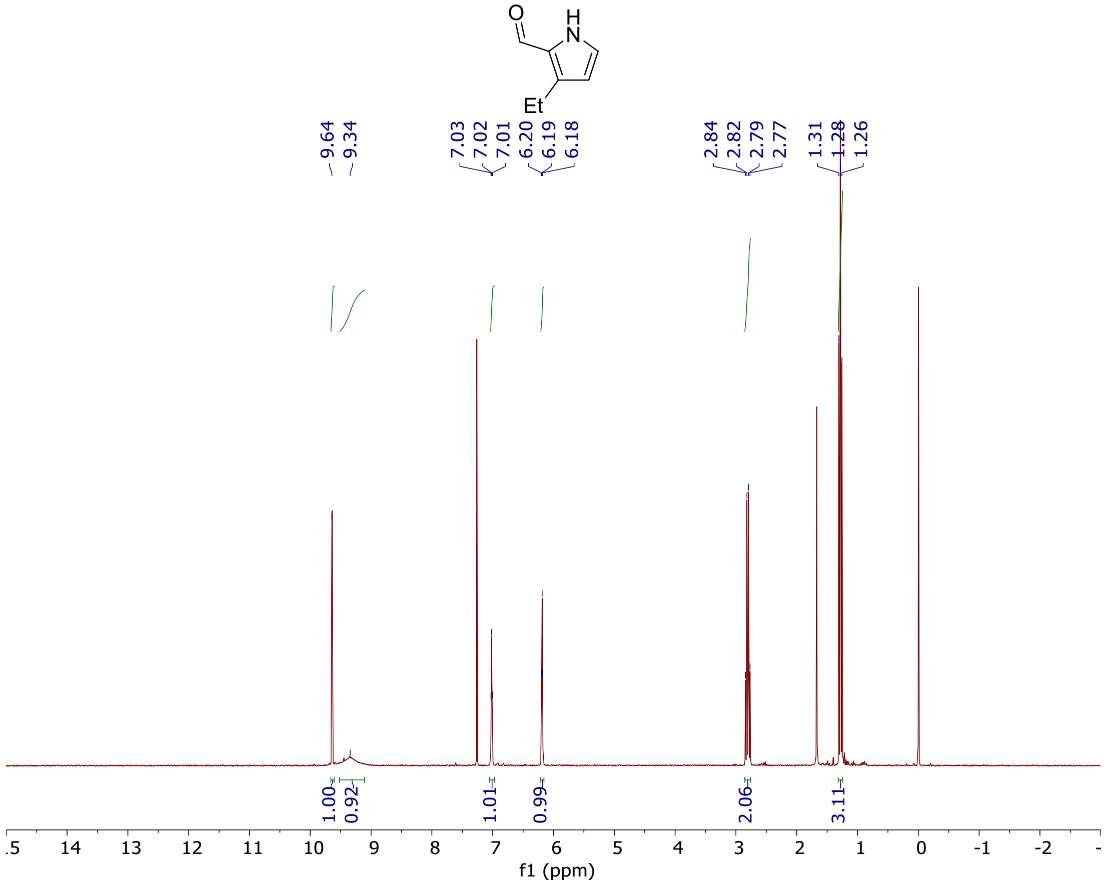

(*E*)-*N*’-((1*H*-pyrrol-2-yl)methylene)-5-bromo-3-phenyl-1*H*-indole-2-carbohydrazide (3a) spectra in dmso-d_6

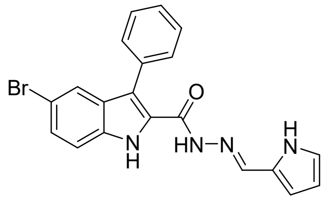

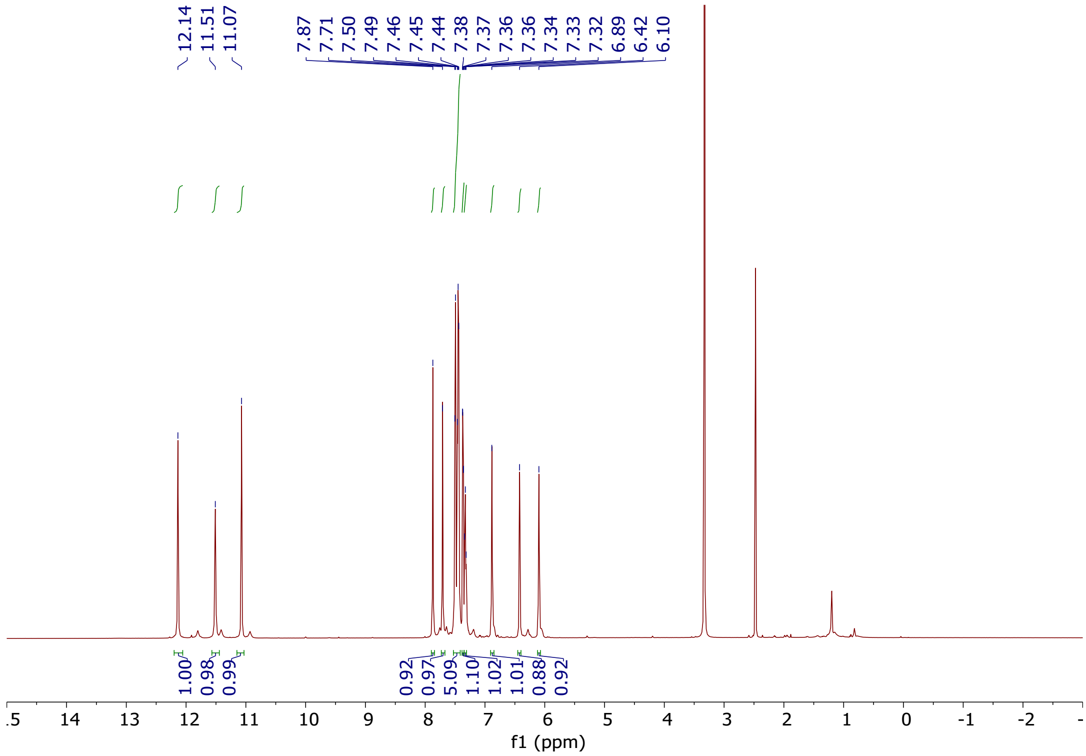

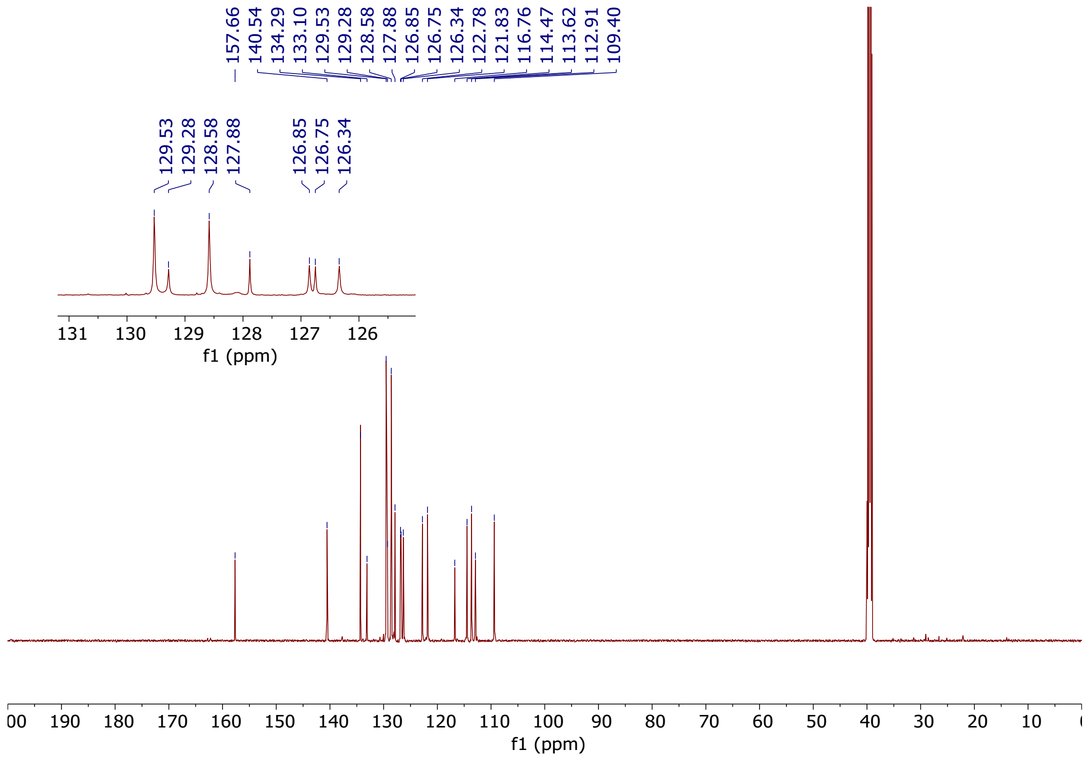

(*E*)-*N*'-((1*H*-pyrrol-3-yl)methylene)-5-bromo-3-phenyl-1*H*-indole-2-carbohydrazide (3b) spectra in dmso-d_6

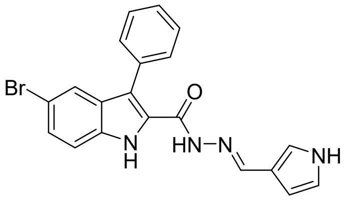

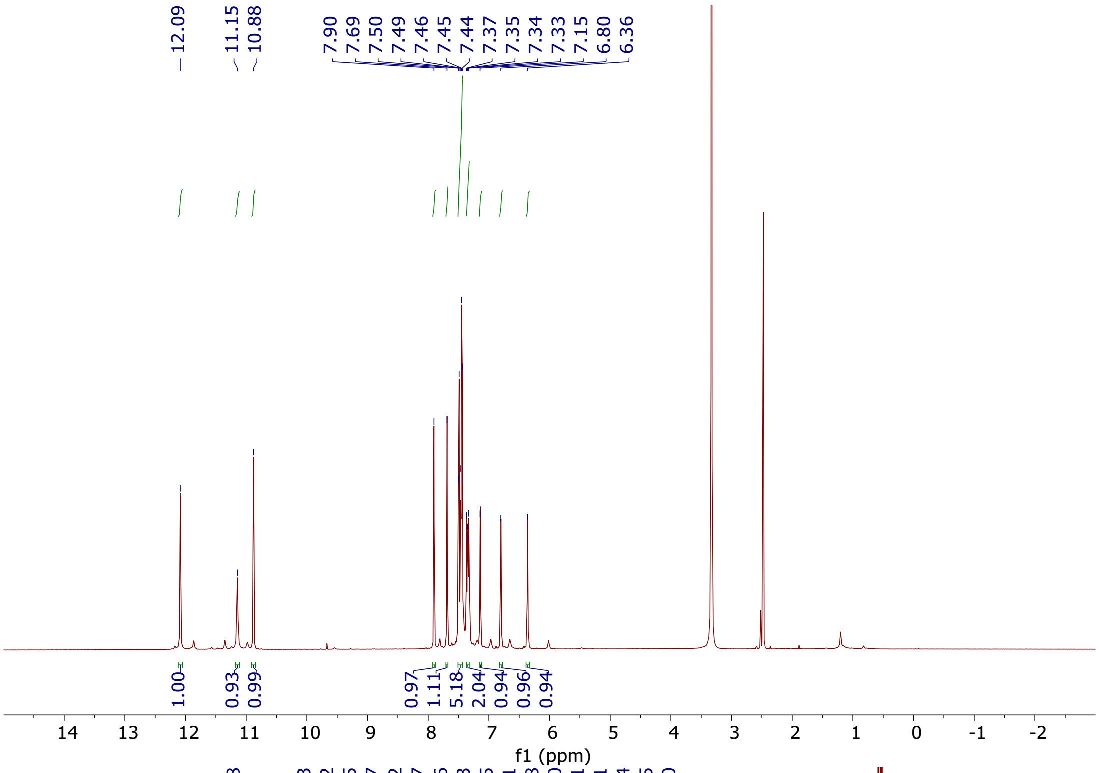

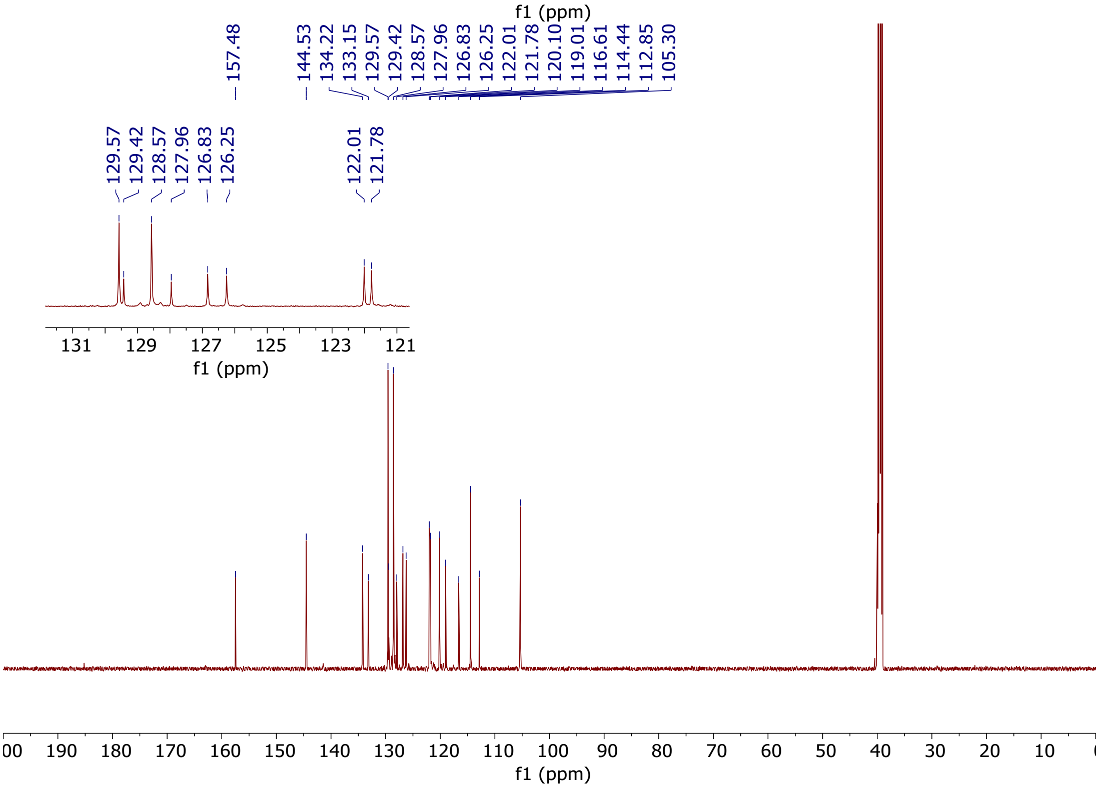

(*E*)-5-bromo-*N*'-((3,5-dimethyl-1*H*-pyrrol-2-yl)methylene)-3-phenyl-1*H*-2-carbohydrazide (3c) spectra in dmso-d_6

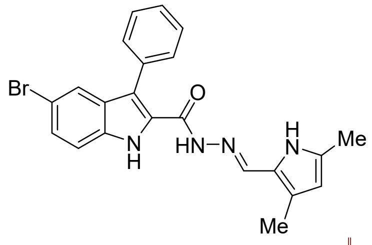

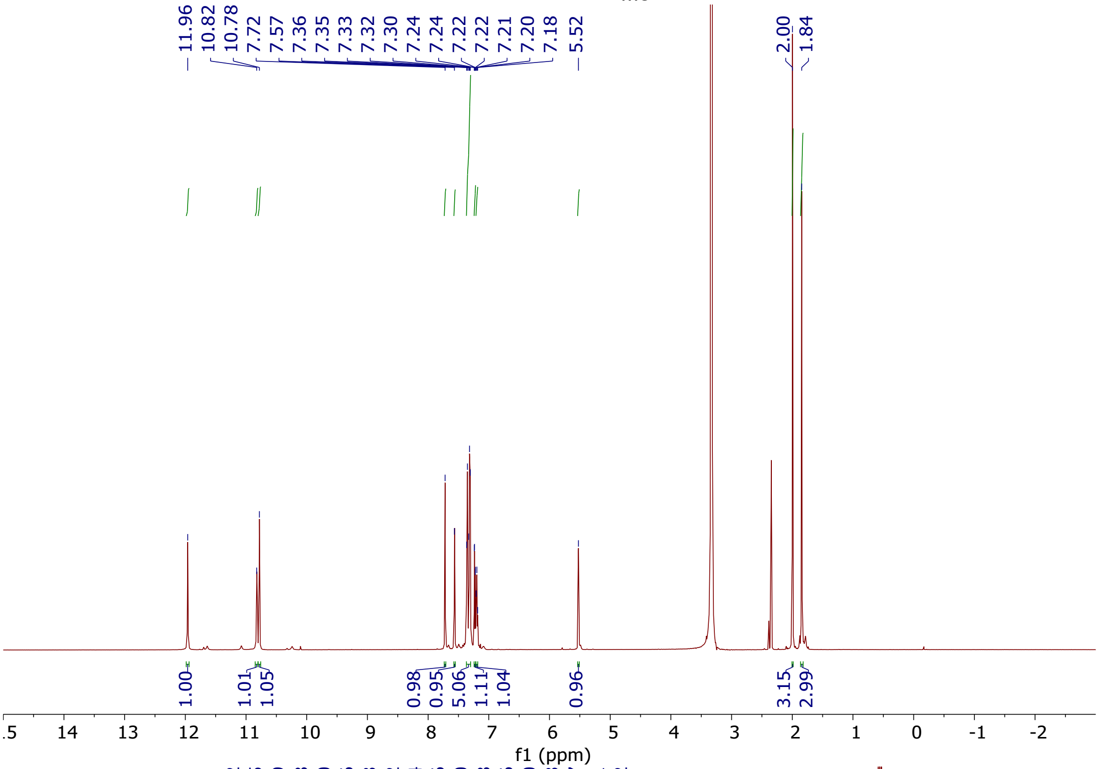

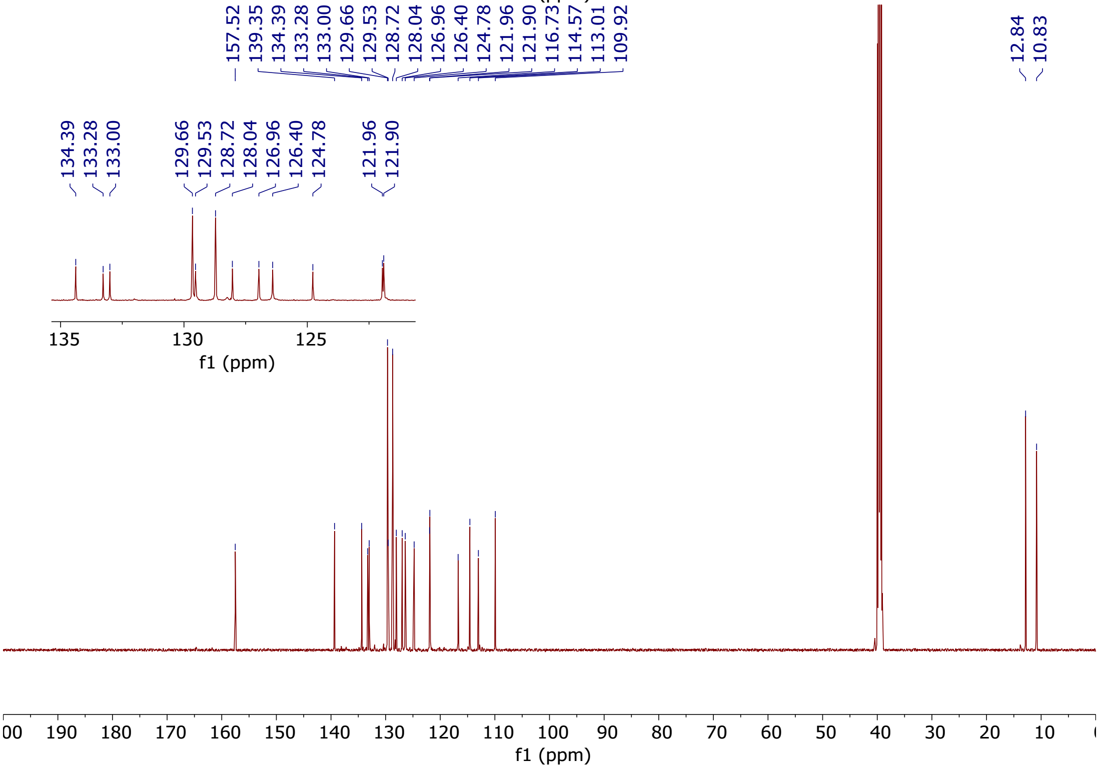

(*E*)-5-bromo-N’-((1-methyl-1*H*-pyrrol-2-yl)methylene)-3-phenyl-1*H*-indole-2-carbohydrazide (3d) spectra in dmso-d_6

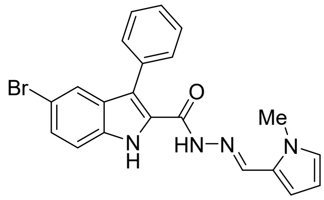

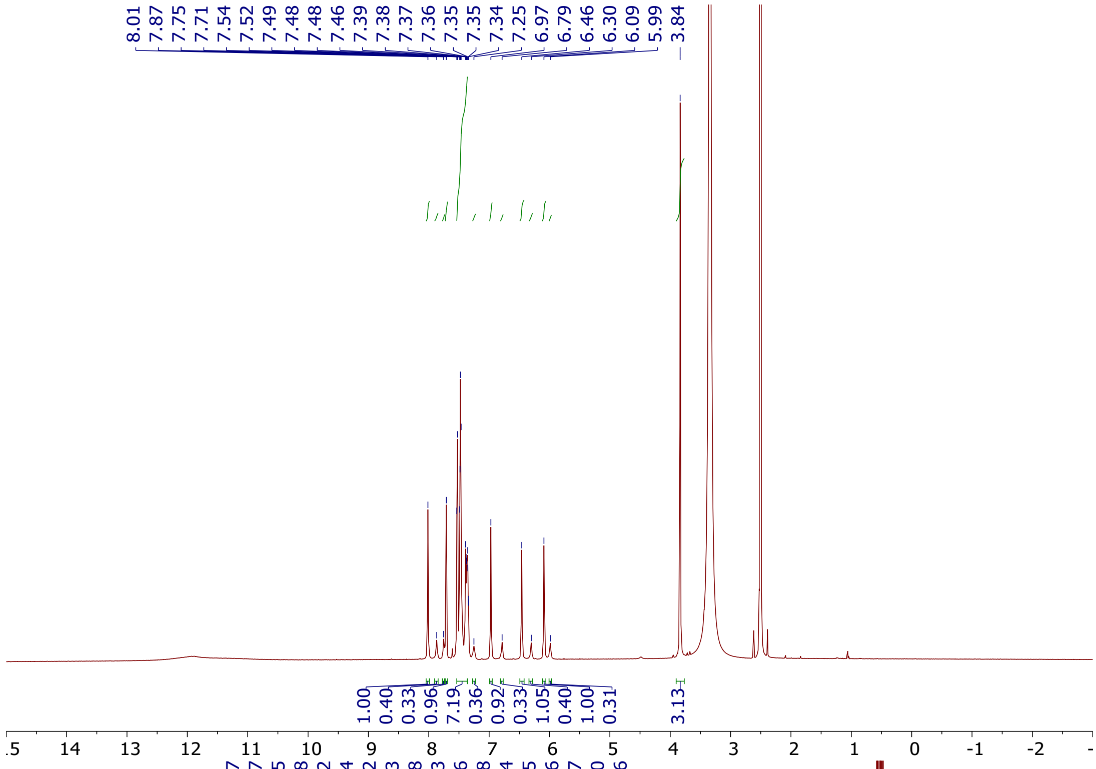

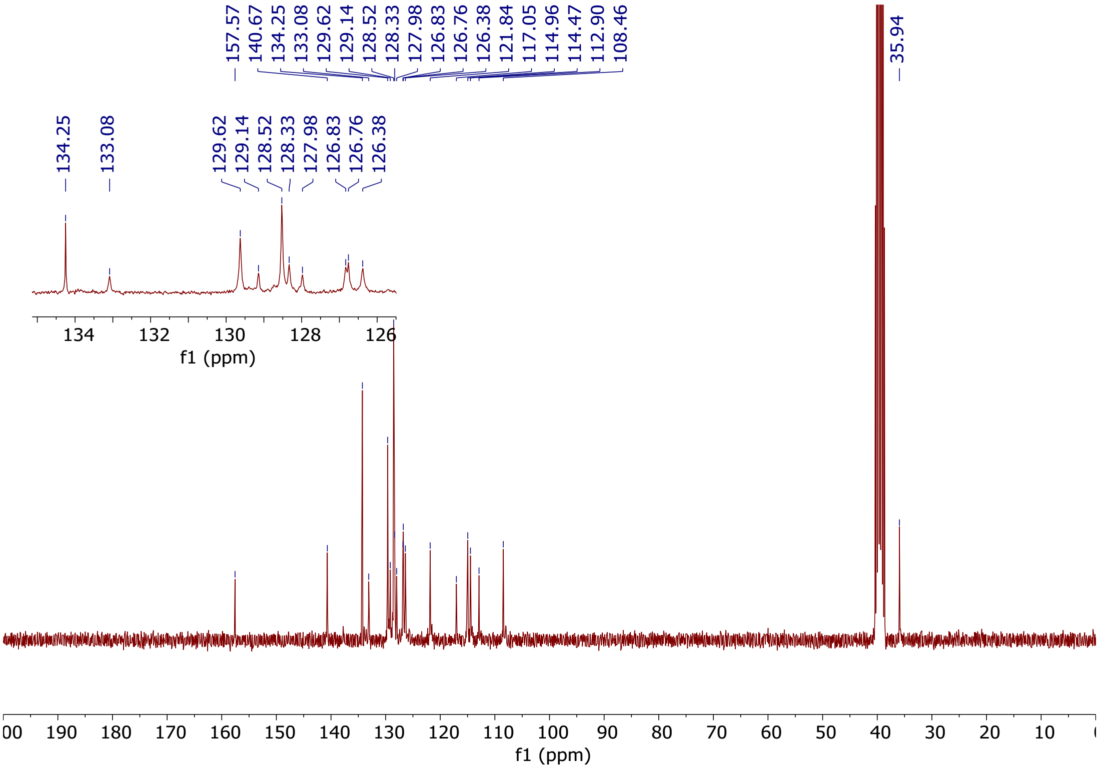

^{1}H NMR of compound (3d) at 90 ^{o}C in dmso-d_6

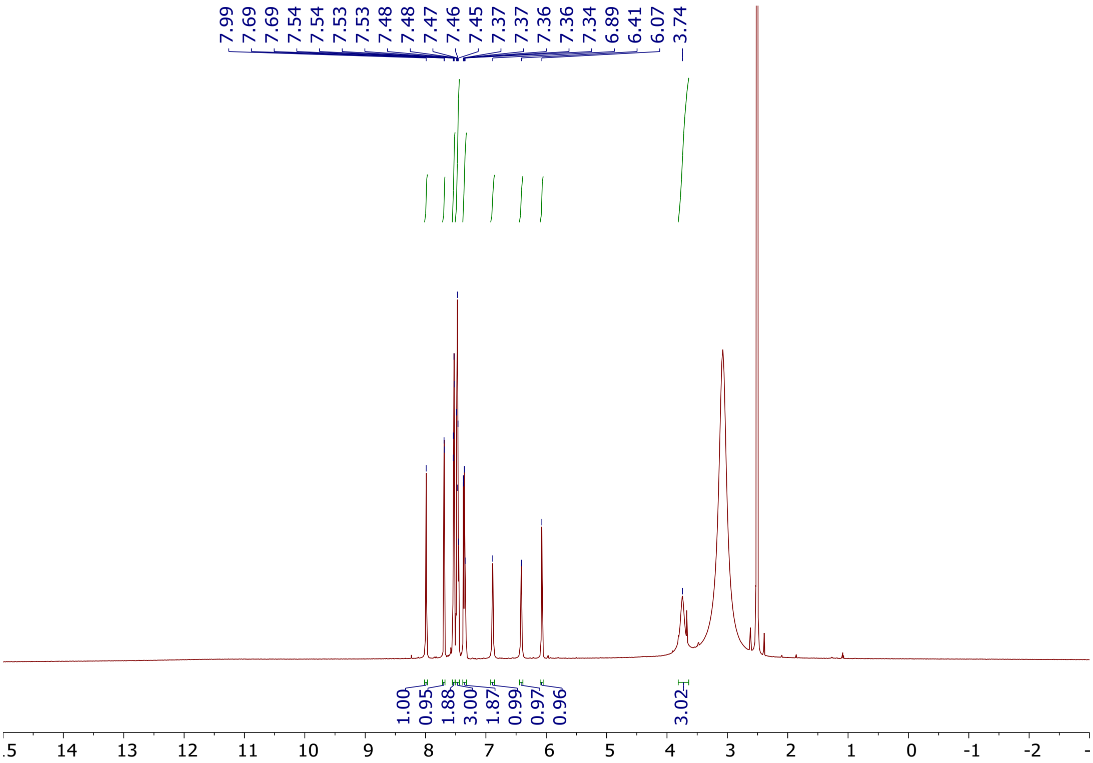

(*E*)-5-bromo-*N*'-((4-bromo-1*H*-pyrrol-2-yl)methylene)-3-phenyl-1*H*-indole-2-carbohydrazide (3e) spectra in dmso-d_6

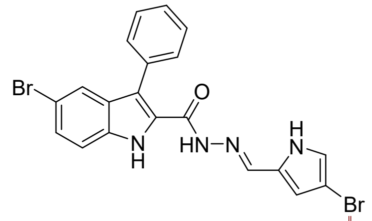

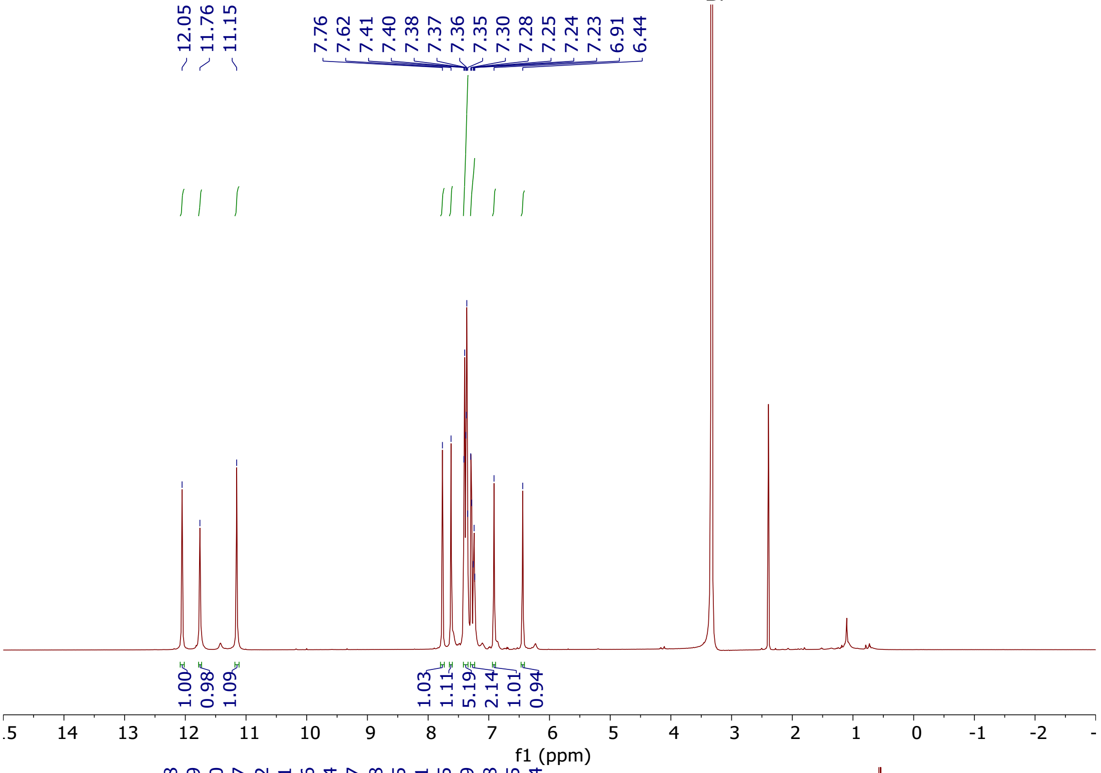

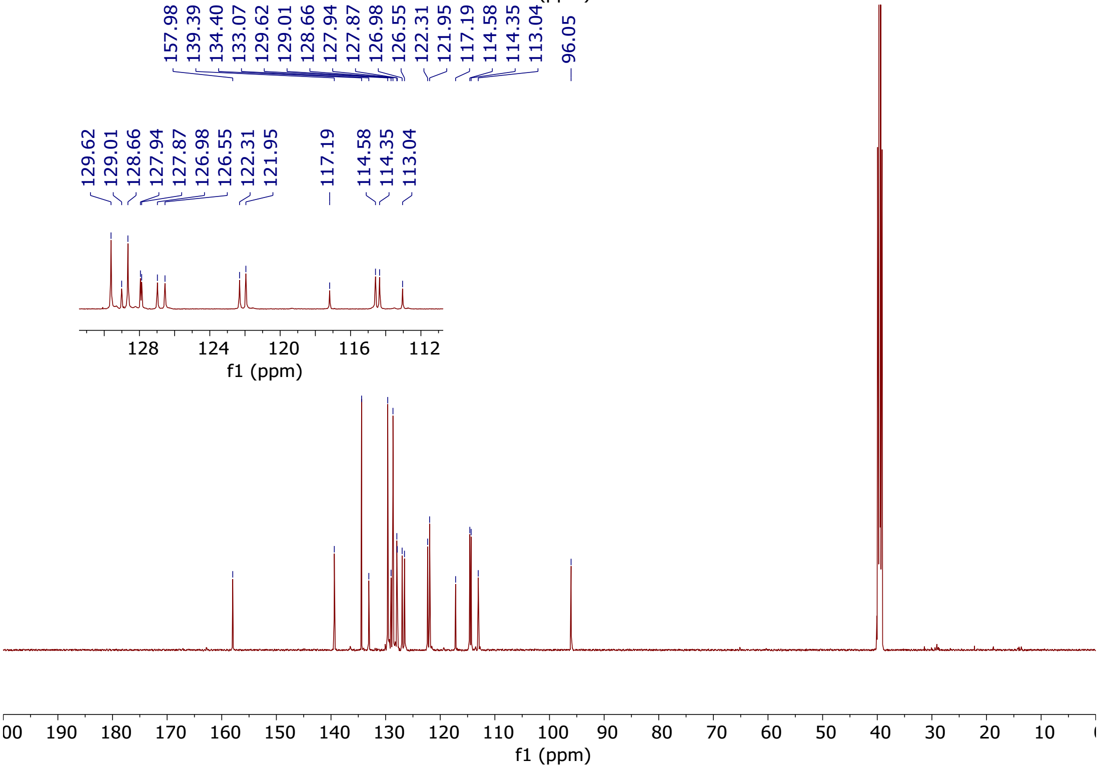

(*E*)-5-bromo-*N*'-((4,5-dibromo-1*H*-pyrrol-2-yl)methylene)-3-phenyl-1*H*-indole-2-carbohydrazide (3f) spectra in dmso-d_6

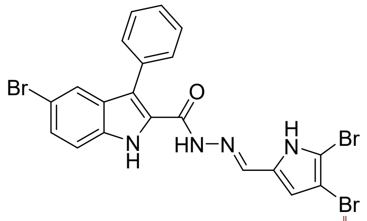

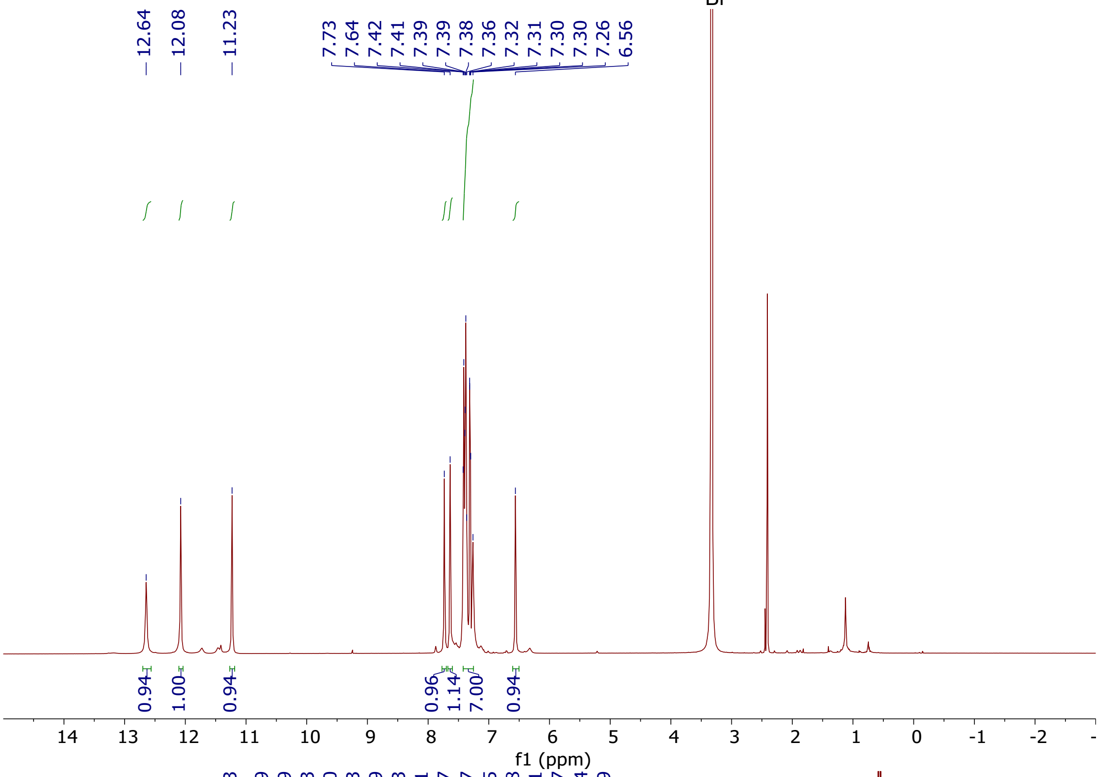

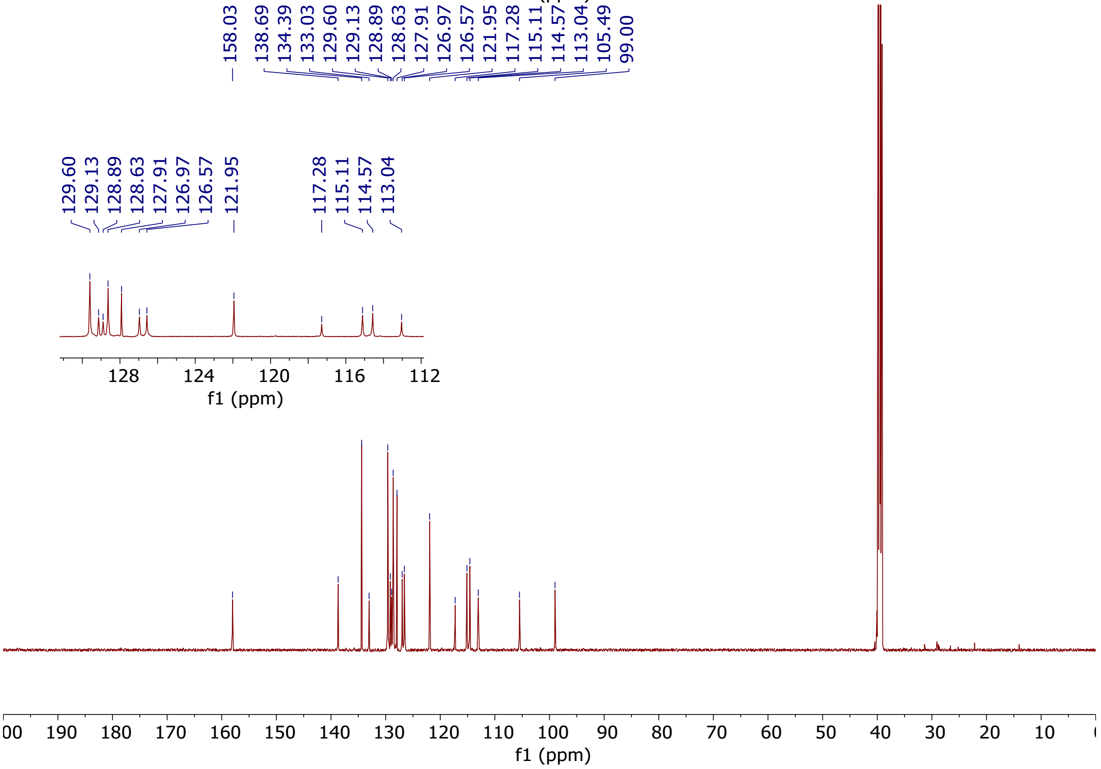

(*E*)-5-bromo-*N*'-((3-chloro-1*H*-pyrrol-2-yl)methylene)-3-phenyl-1*H*-indole-2-carbohydrazide (3g) spectra in dmso-d_6

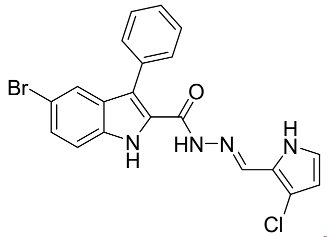

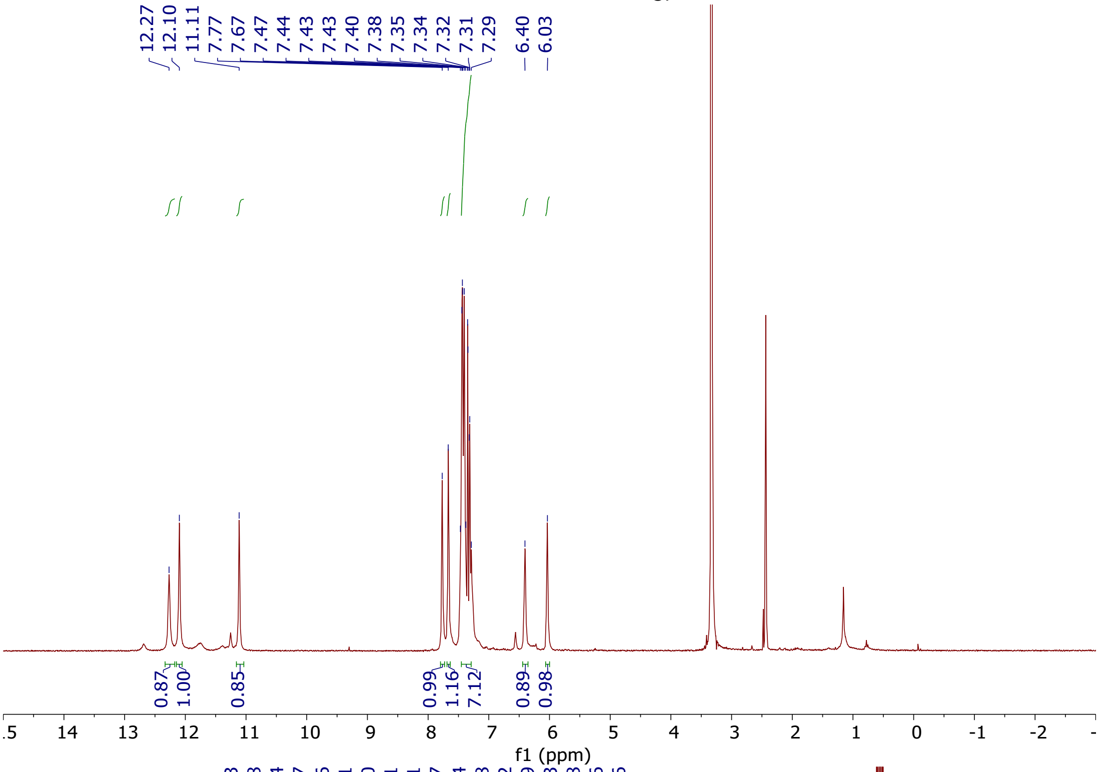

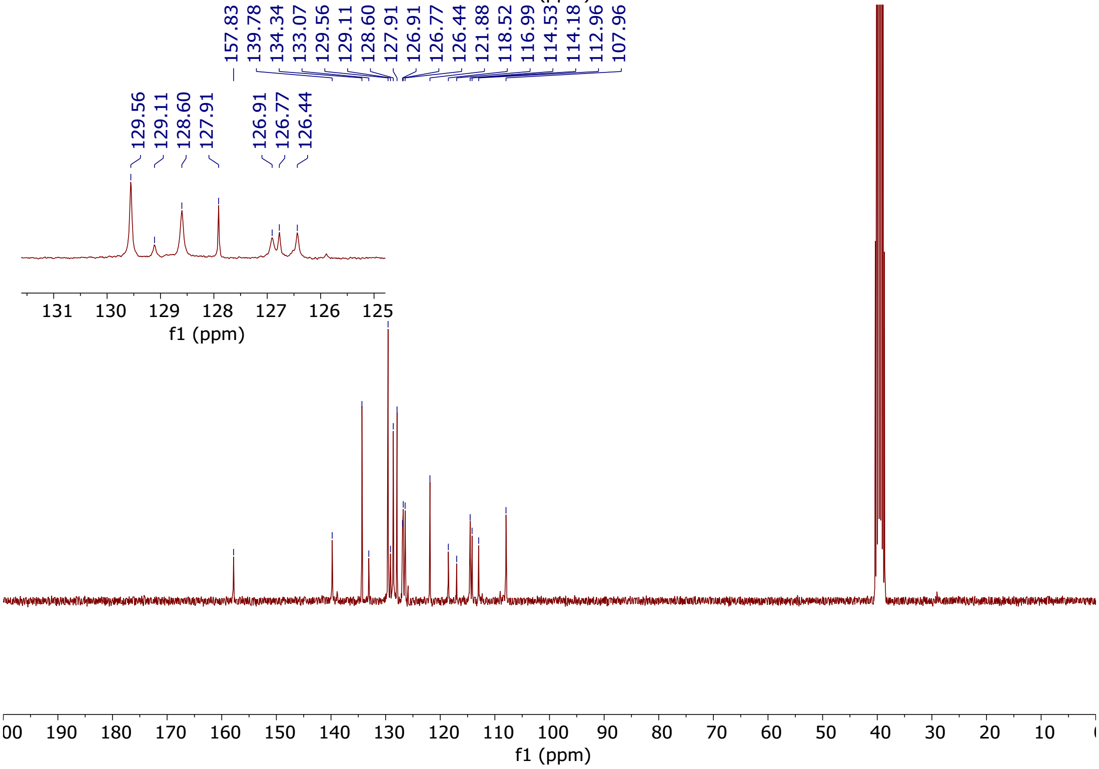

(*E*)-5-bromo-*N*'-((4-chloro-1*H*-pyrrol-2-yl)methylene)-3-phenyl-1*H*-indole-2-carbohydrazide (3h) spectra in dmso-d_6

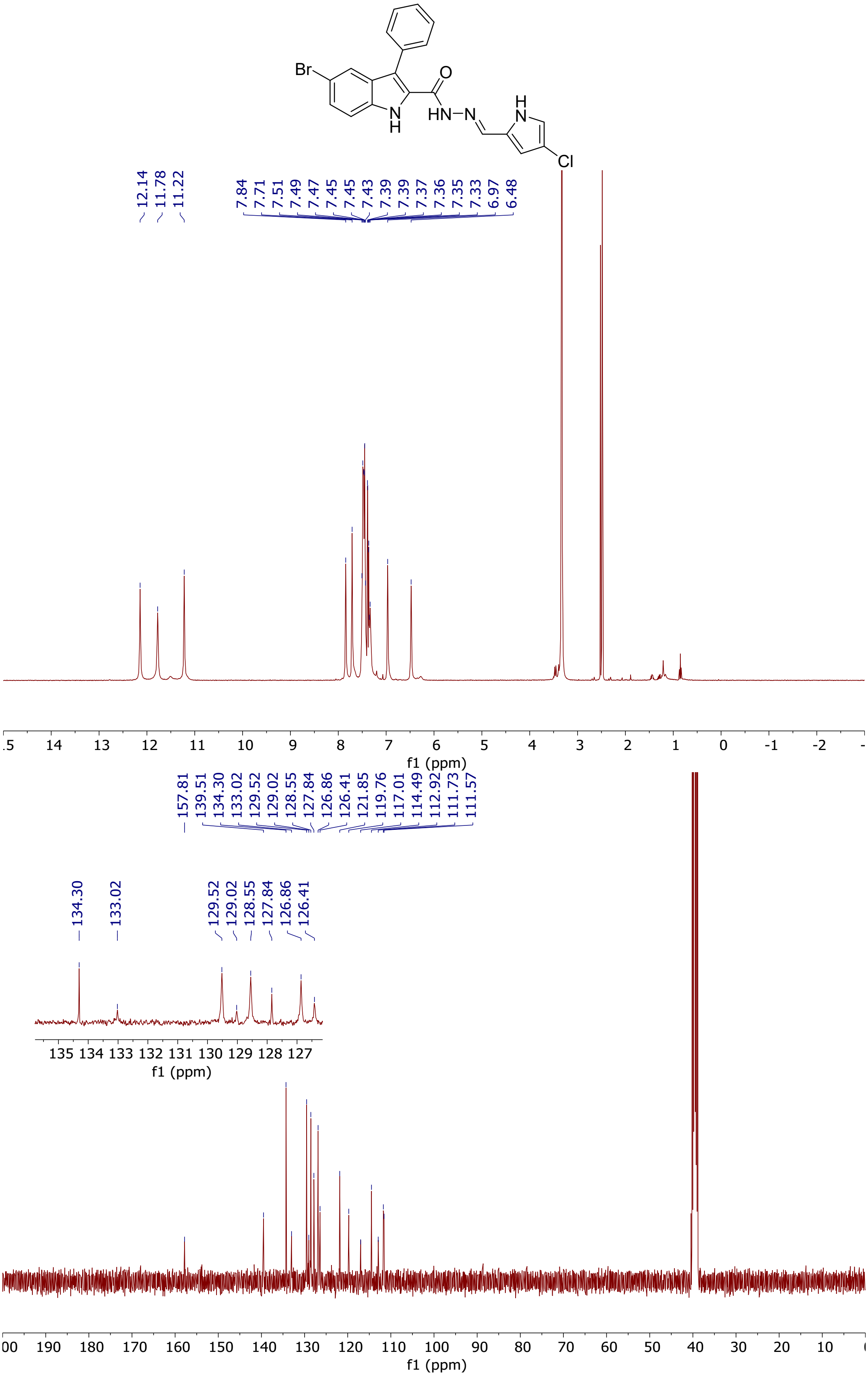

Ethyl (*E*)-5-((2-(5-bromo-3-phenyl-1*H*-indole-2-carbonyl)hydrazono)methyl)-1*H*-pyrrole-2-carboxylate (3i) spectra in dmso-d_6

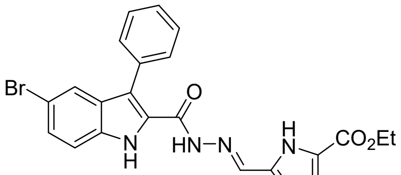

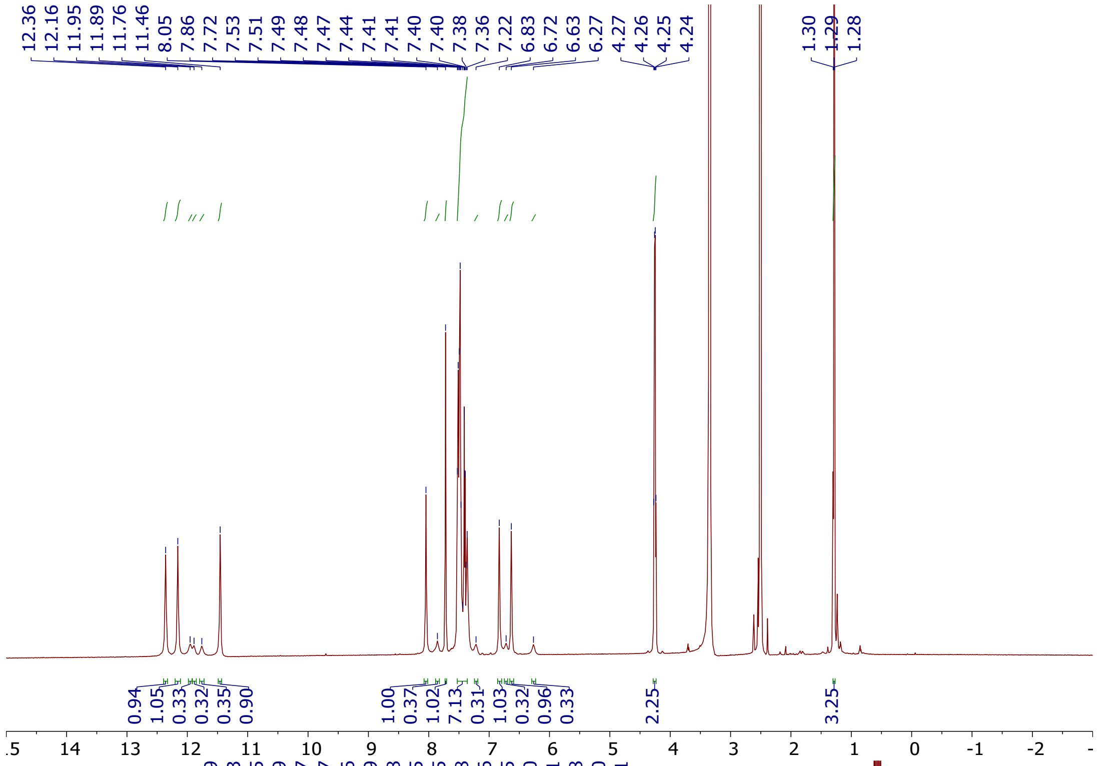

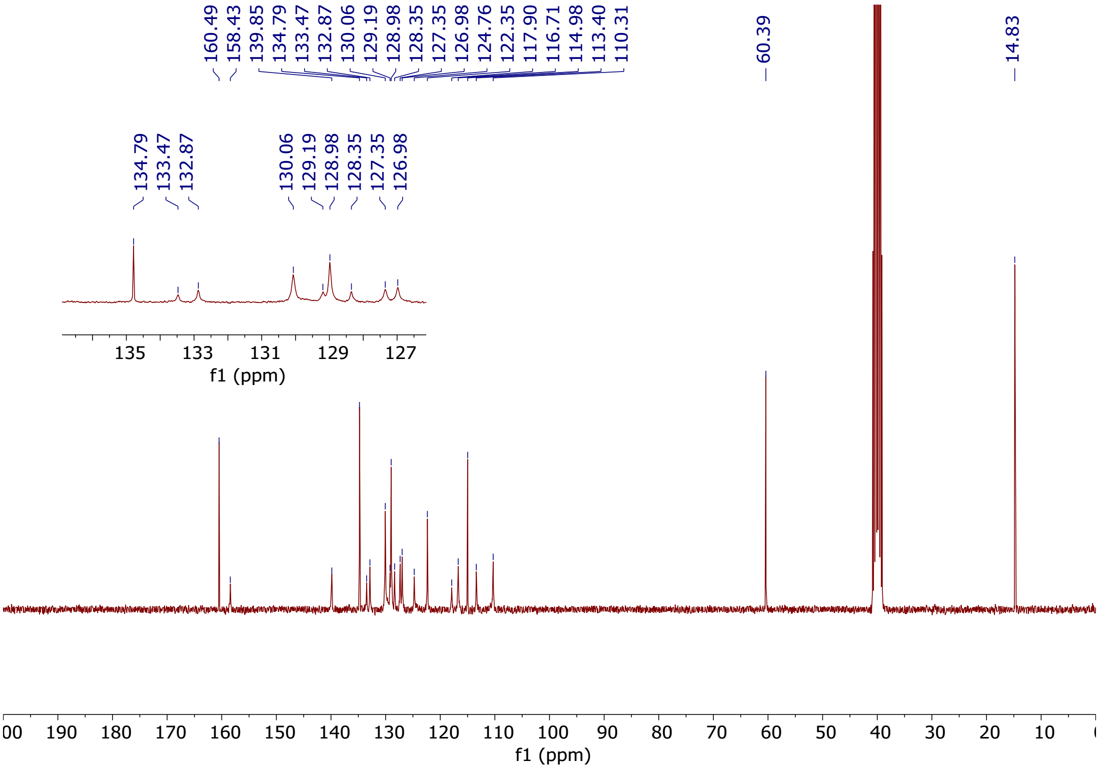

^1H NMR of compound (3i) at 90 °C in dmso-d_6

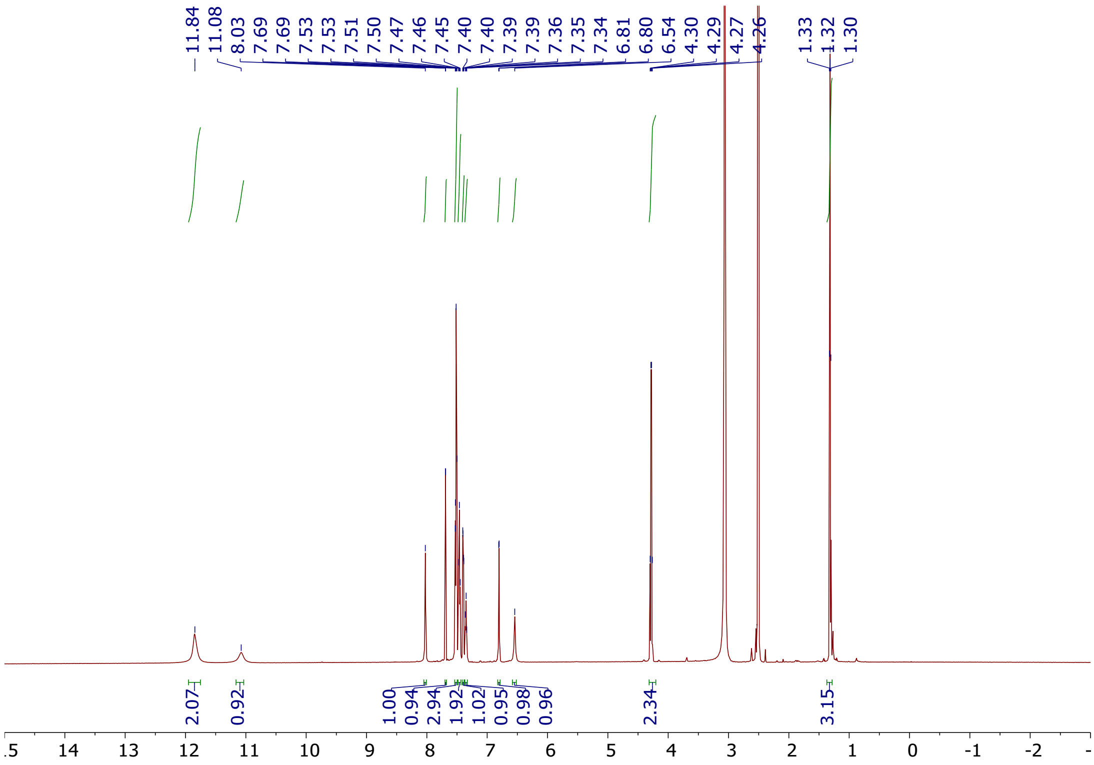

Ethyl (*E*)-5-((2-(5-bromo-3-phenyl-1*H*-indole-2-carbonyl)hydrazono)methyl)-1*H*-pyrrole-3-carboxylate (3j) spectra in dmso-d_6

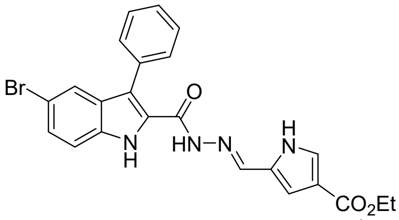

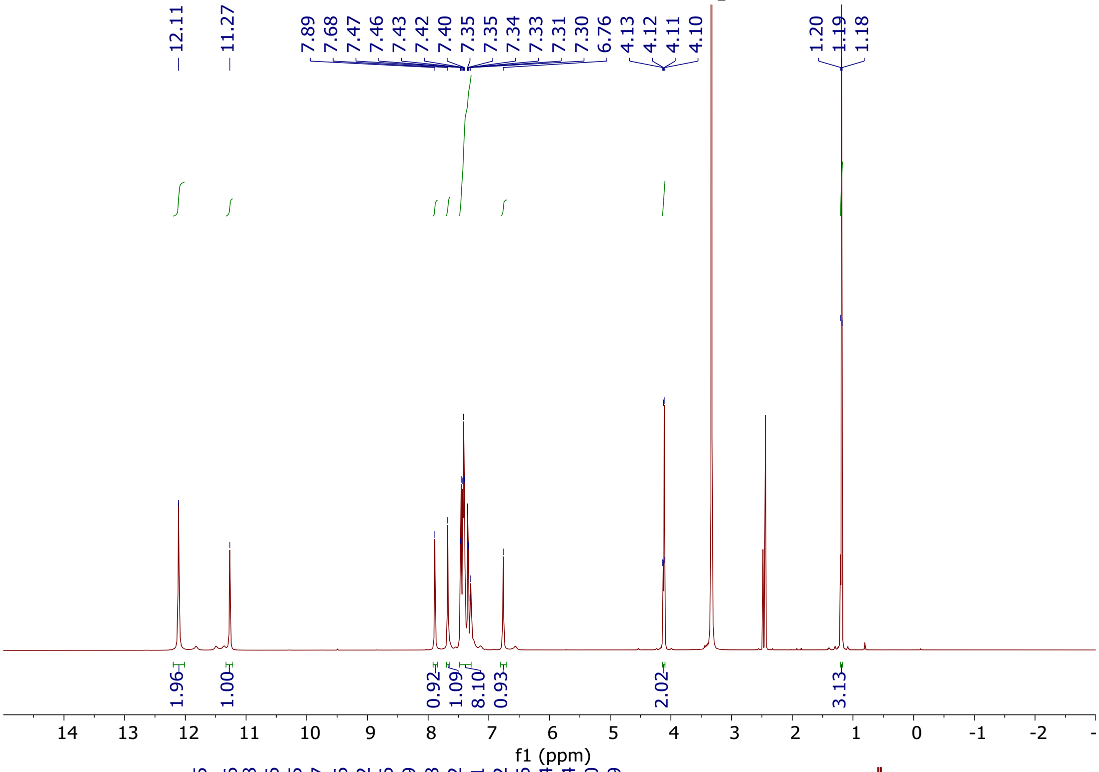

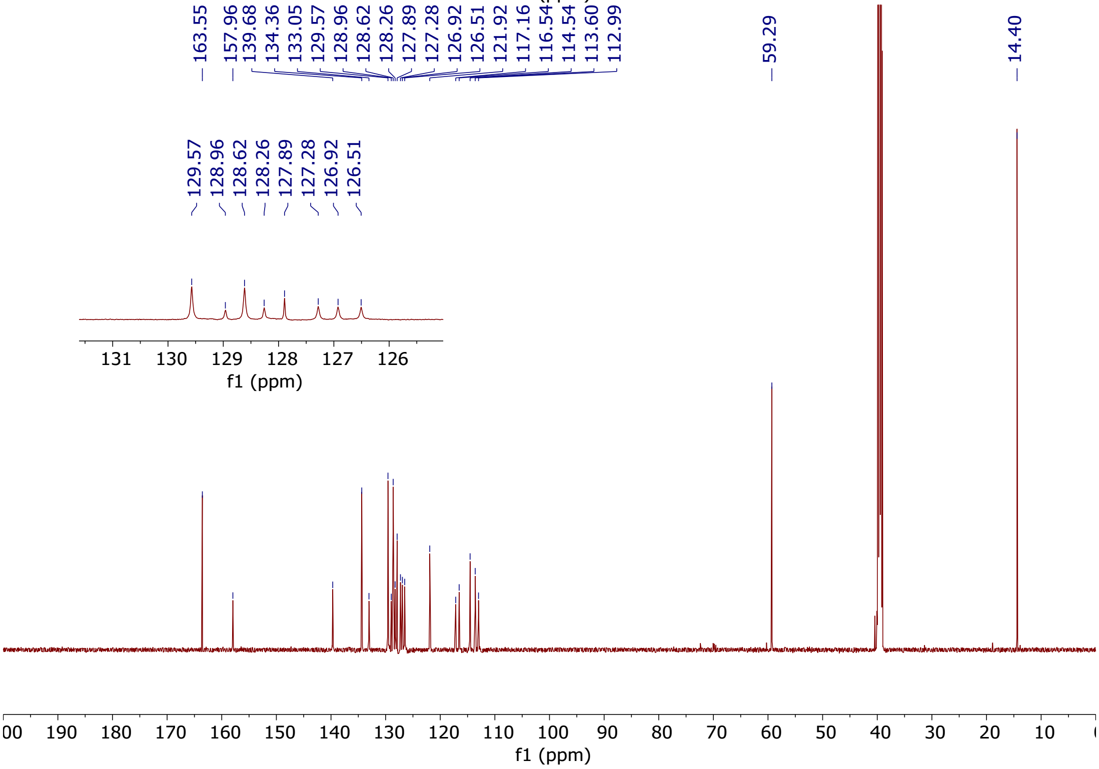

Ethyl (*E*)-2-((2-(5-bromo-3-phenyl-1*H*-indole-2-carbonyl)hydrazono)methyl)-1*H*-pyrrole-3-carboxylate (3k) spectra in dmso-d_6

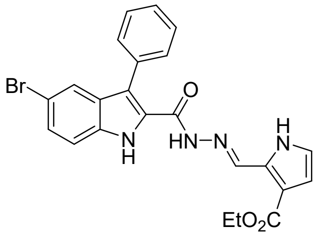

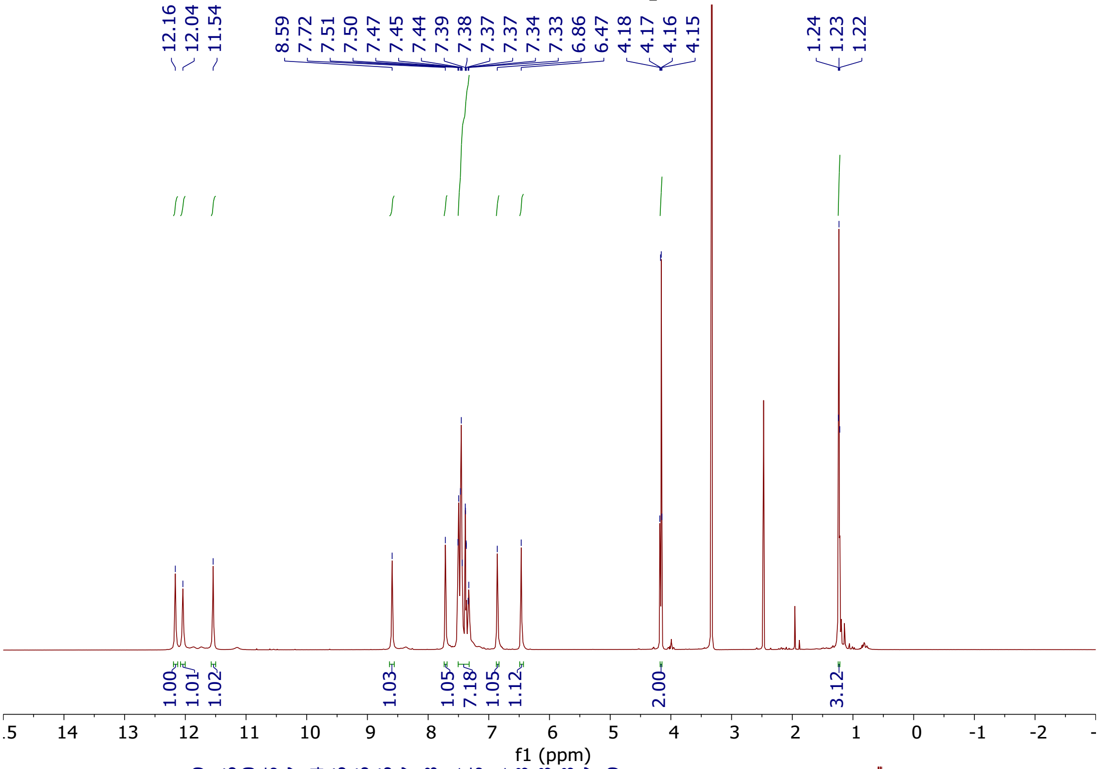

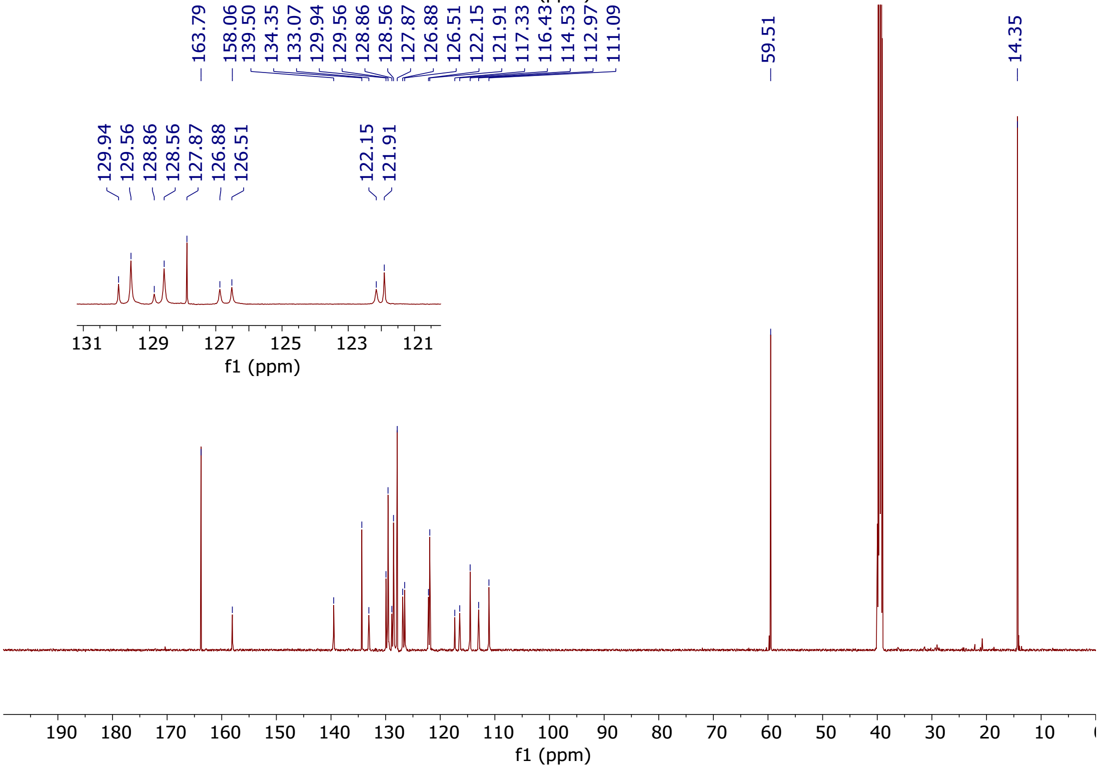

(*E*)-5-((2-(5-bromo-3-phenyl-1*H*-indole-2-carbonyl)hydrazono)methyl)-1*H*-pyrrole-2-carboxylic acid (3l) spectra in dmso-d$_6$

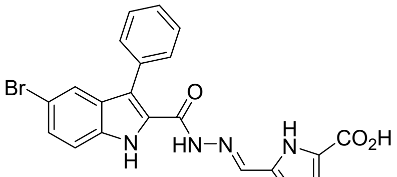

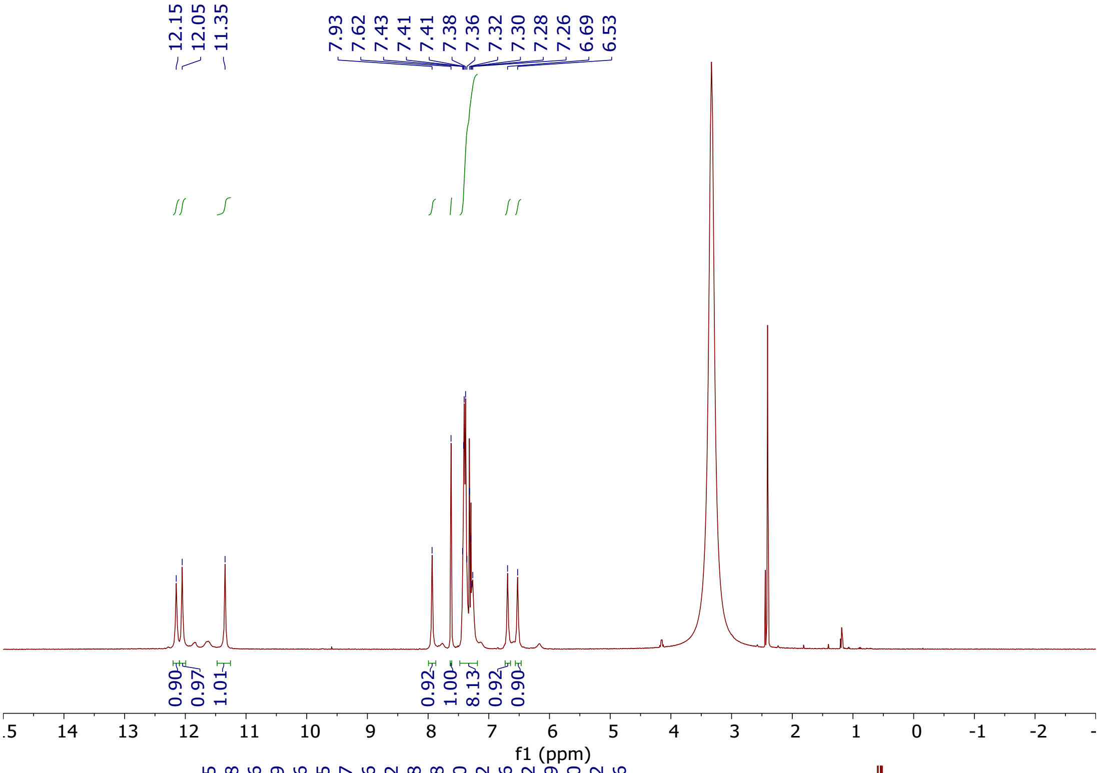

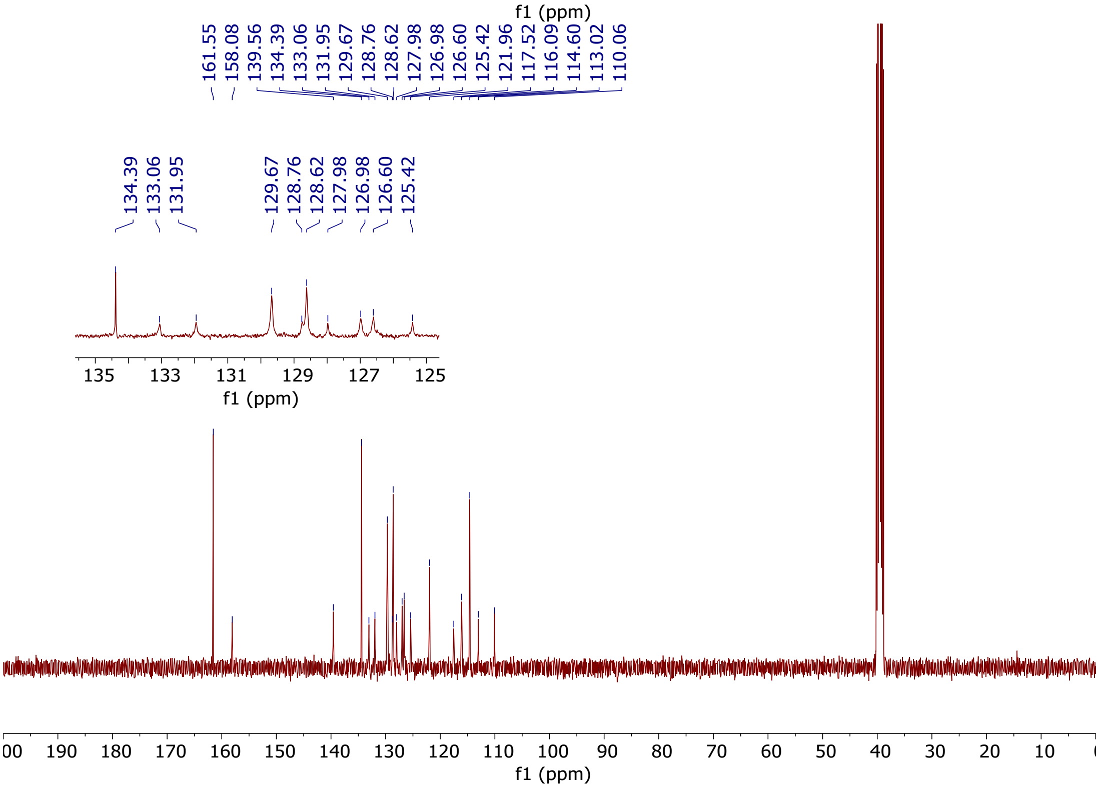

(*E*)-5-((2-(5-bromo-3-phenyl-1*H*-indole-2-carbonyl)hydrazono)methyl)-1*H*-pyrrole-3-carboxylic acid (3m) spectra in dmso-d_6

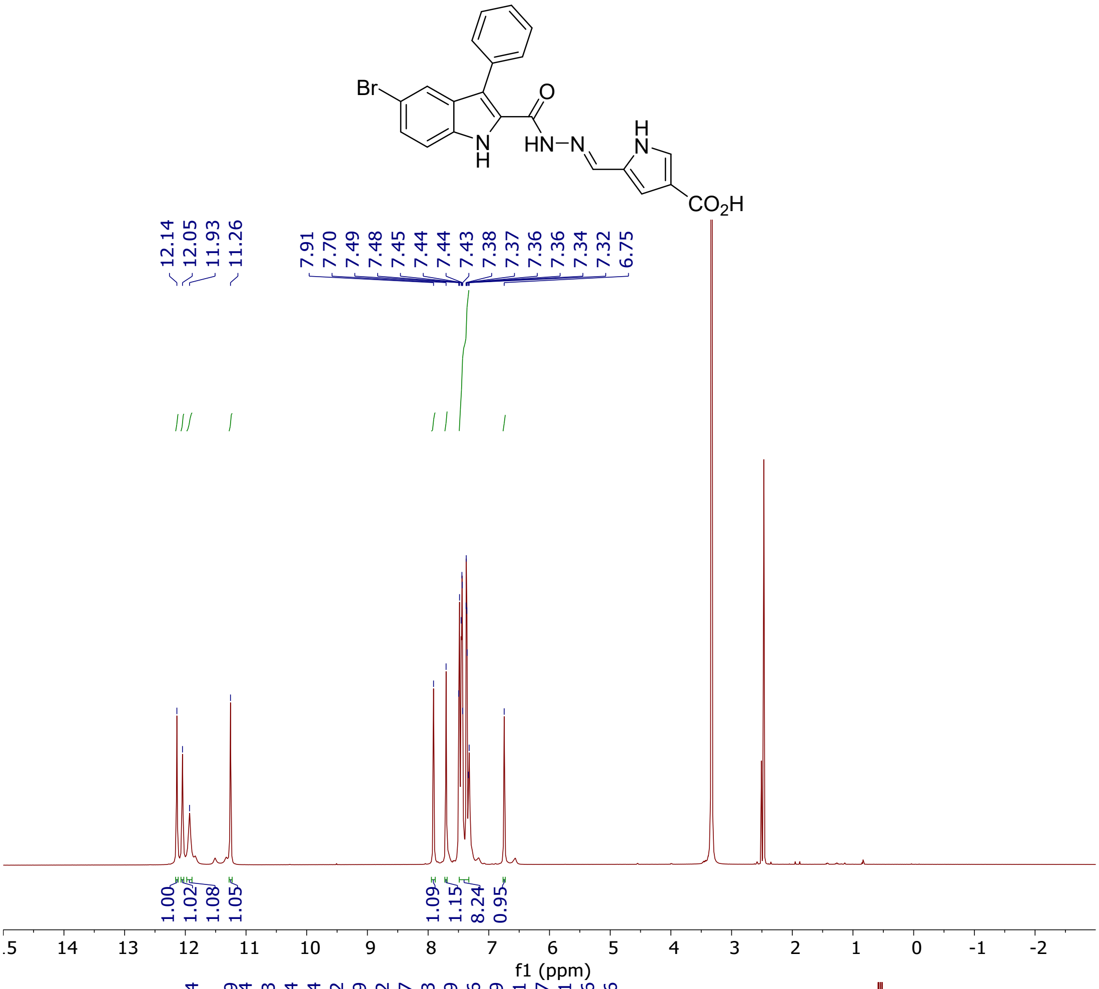

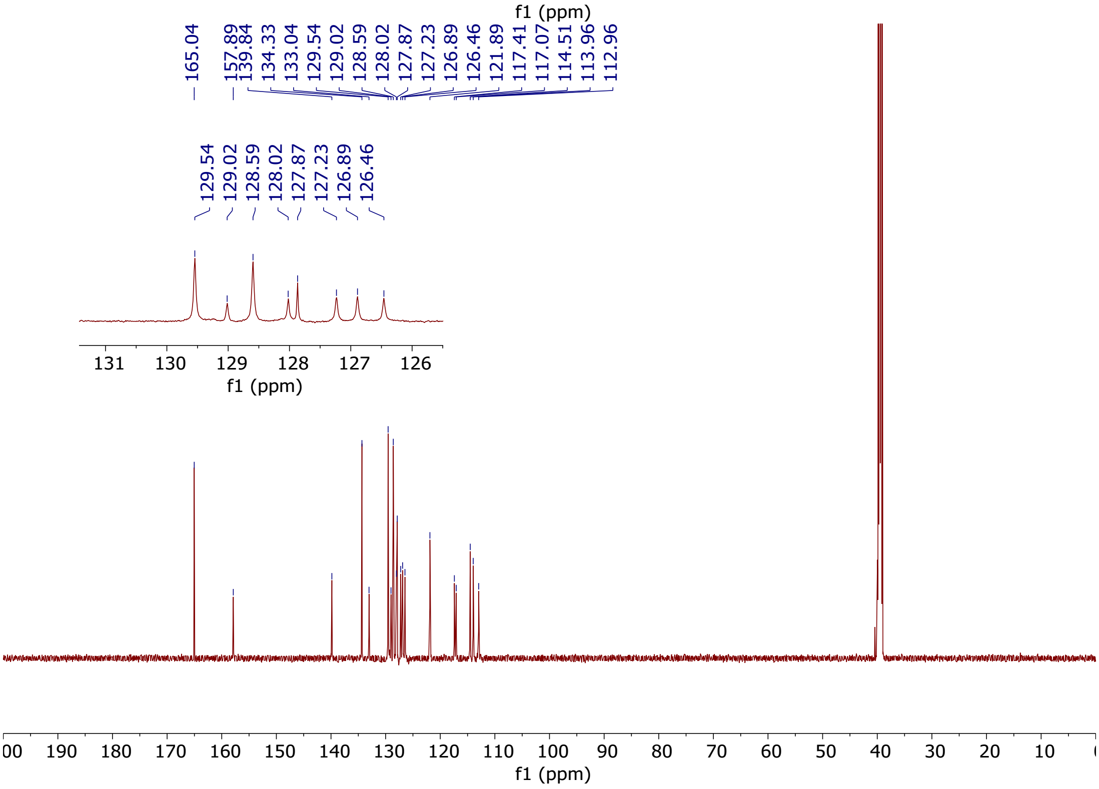

(*E*)-5-bromo-*N*'-((5-(morpholine-4-carbonyl)-1*H*-pyrrol-2-yl)methylene)-3-phenyl-1*H*-indole-2-carbohydrazide (3n) spectra in dmso-d_6

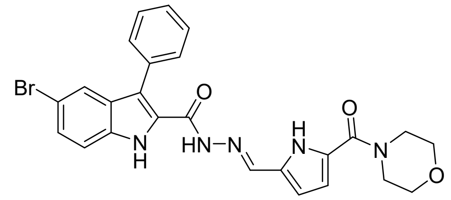

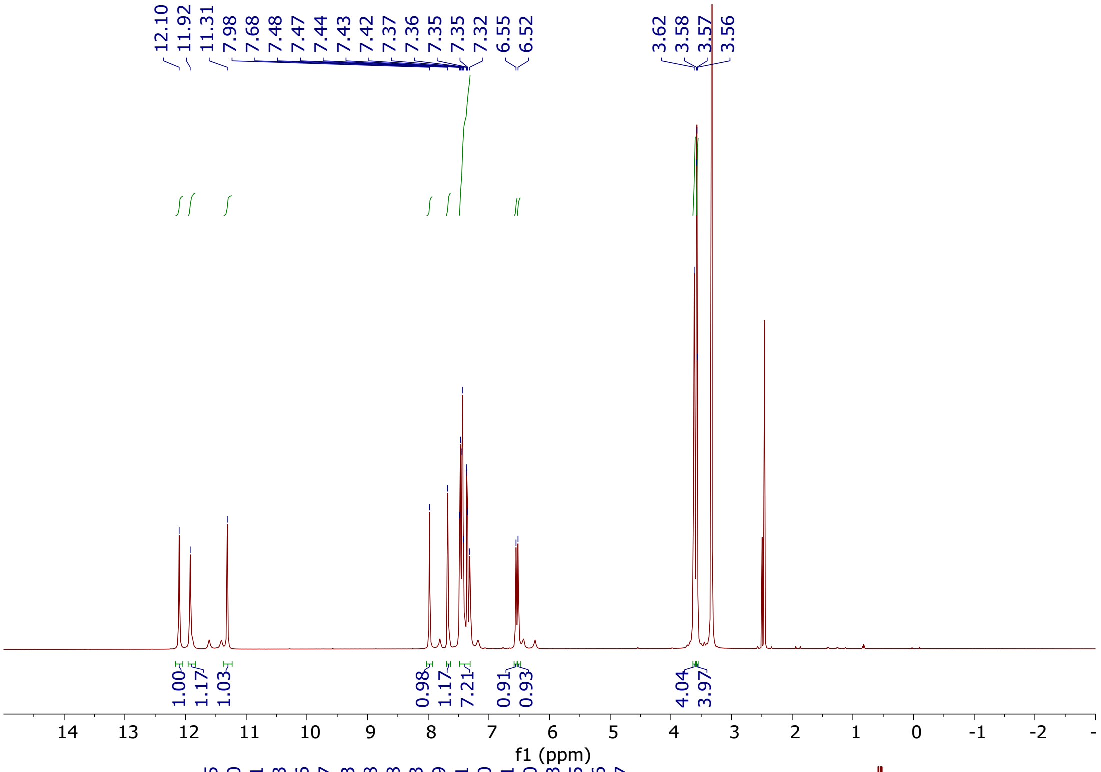

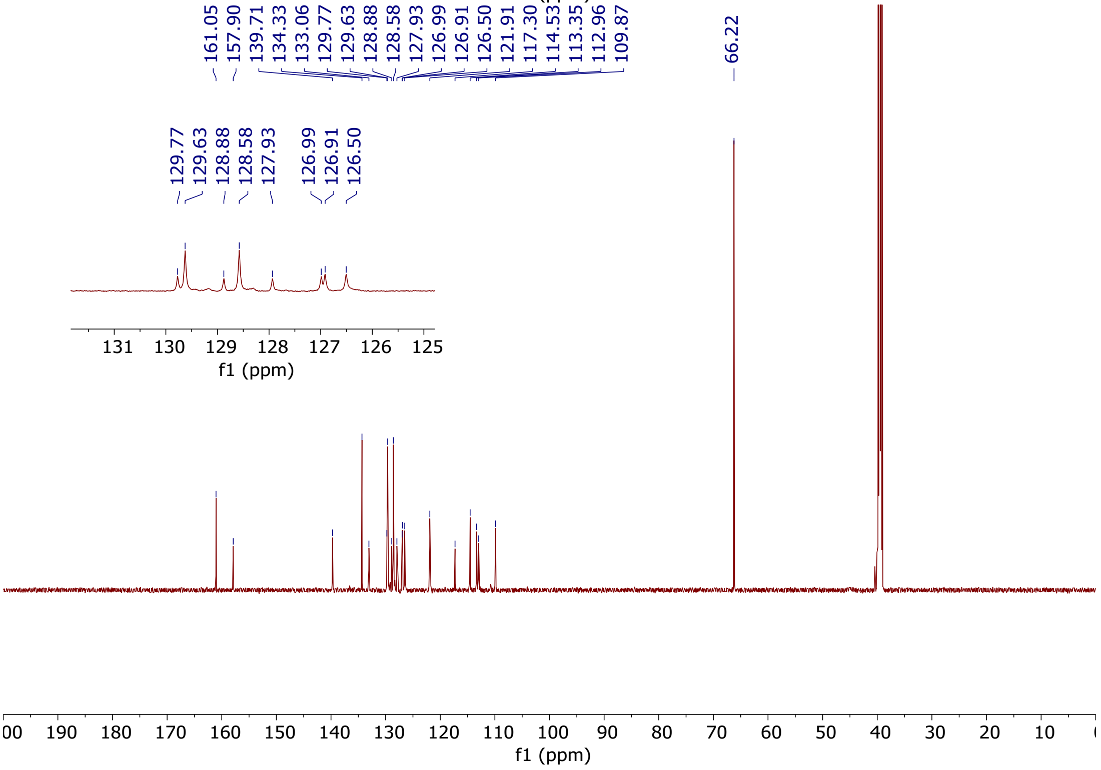

(*E*)-5-bromo-*N*'-((4-(morpholine-4-carbonyl)-1*H*-pyrrol-2-yl)methylene)-3-phenyl-1*H*-indole-2-carbohydrazide (3o) spectra in dmso-d_6

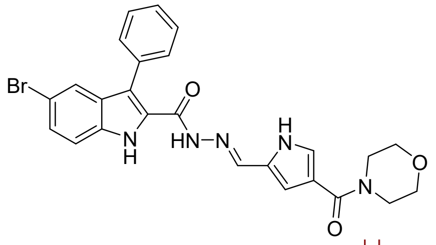

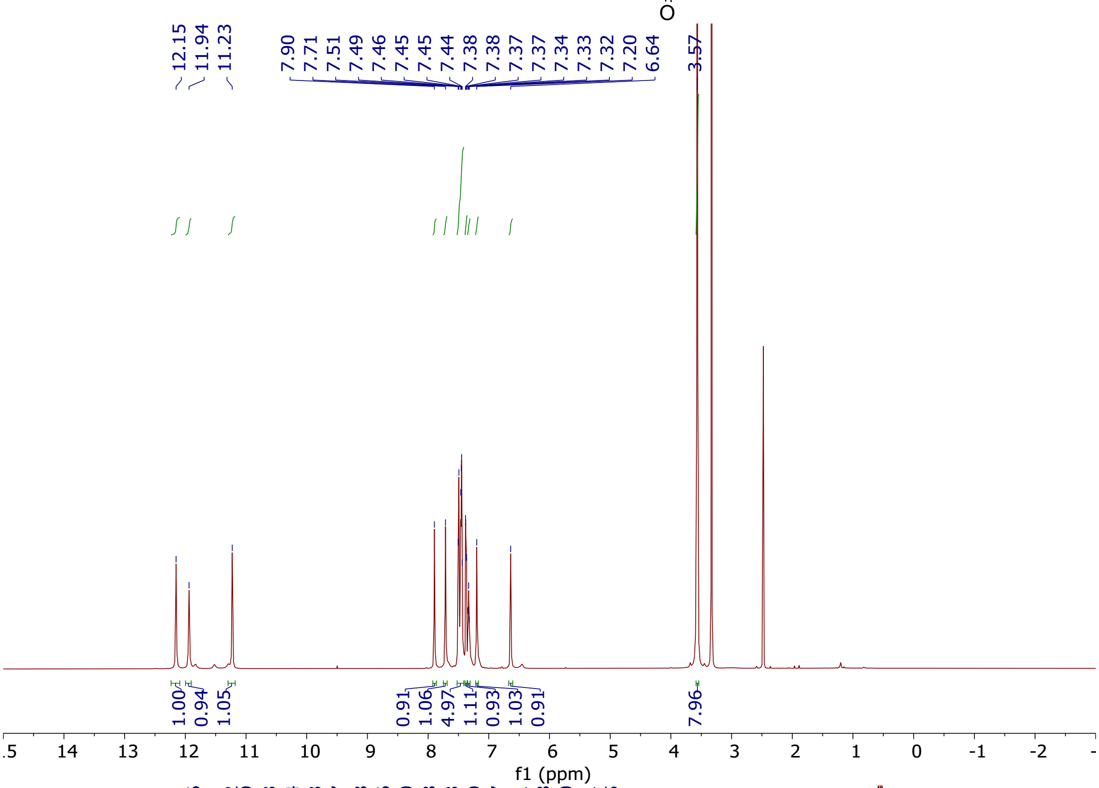

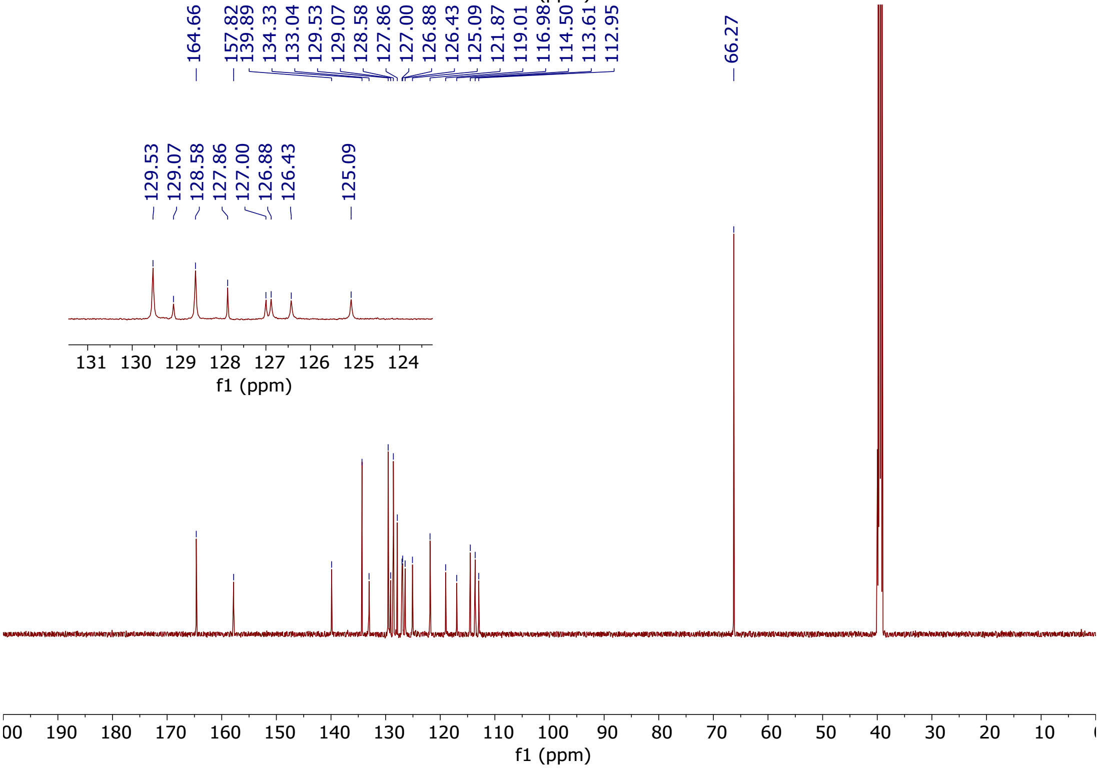

(*E*)-5-bromo-*N*'-((5-nitro-1*H*-pyrrol-2-yl)methylene)-3-phenyl-1*H*-indole-2-carbohydrazide (3p) spectra in dmso-d_6

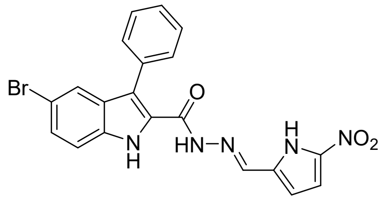

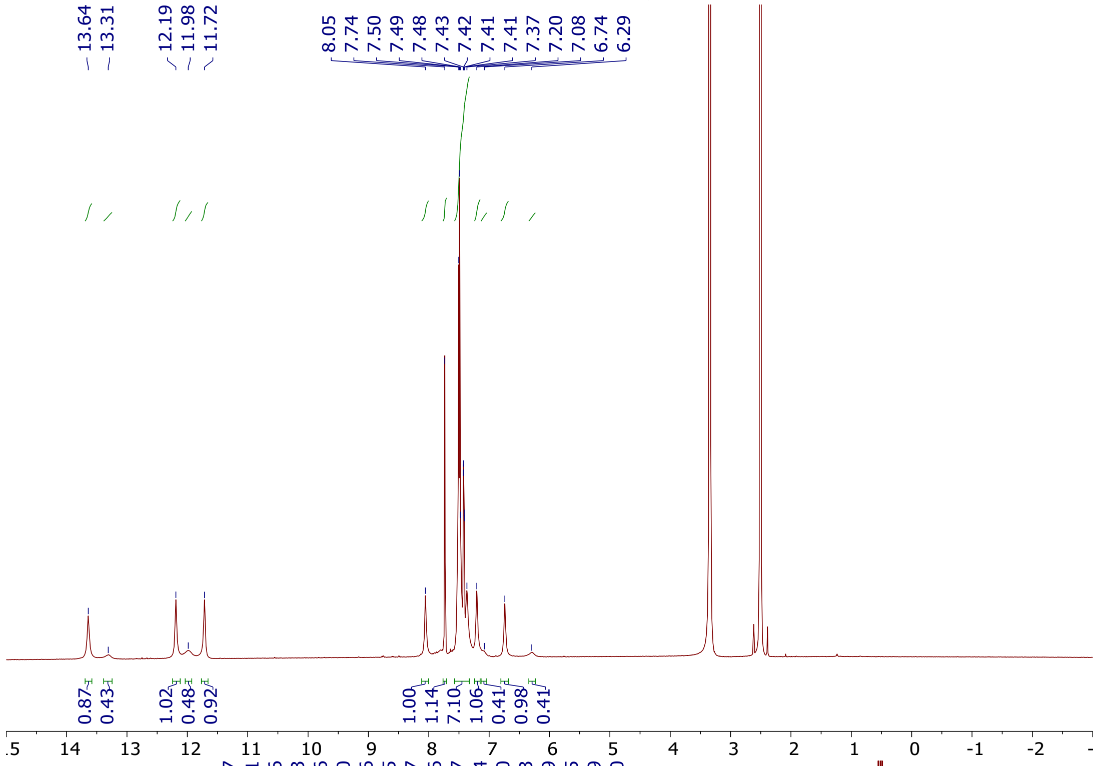

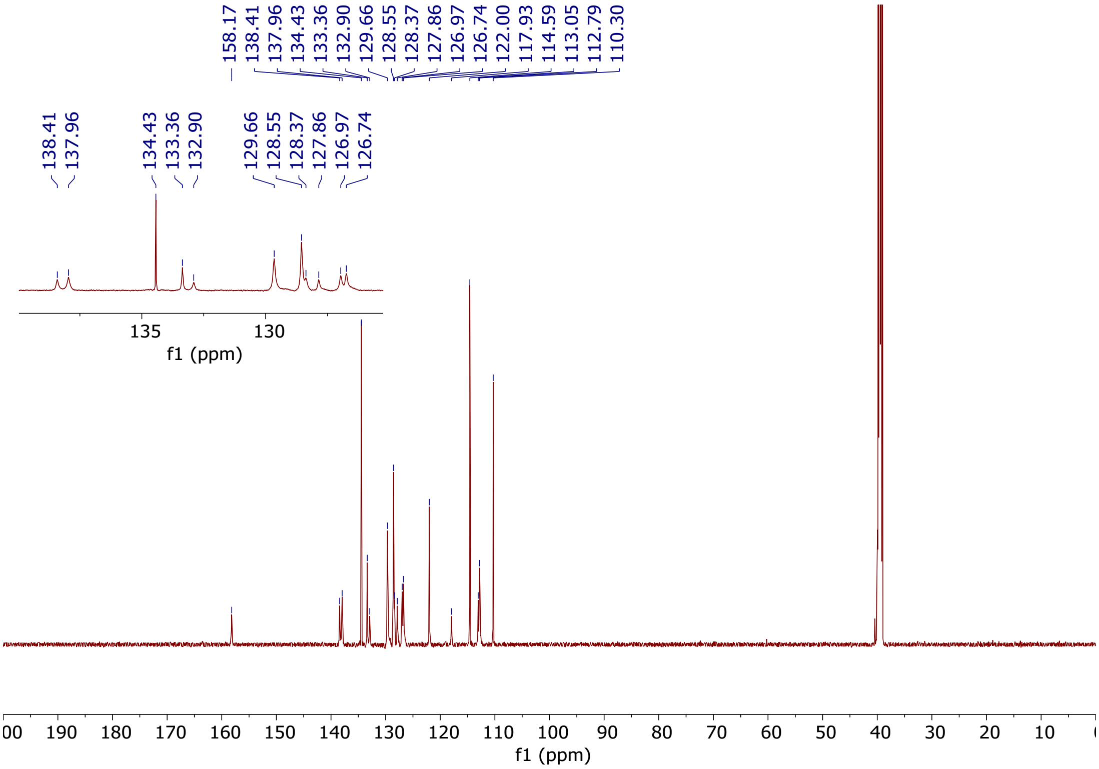

^{1}H NMR of compound (3p) at 90 °C in dmso-d_6

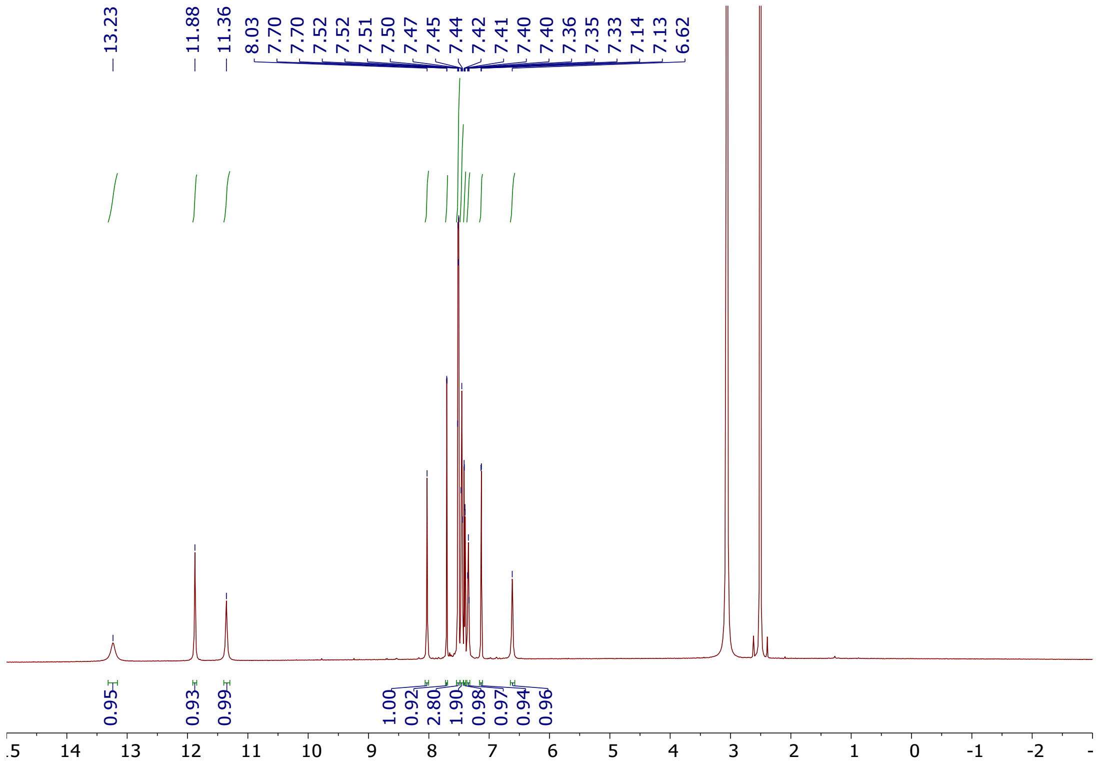

(*E*)-5-bromo-*N*’-((4-nitro-1*H*-pyrrol-2-yl)methylene)-3-phenyl-1*H*-indole-2-carbohydrazide (3q) spectra in dmso-d_6

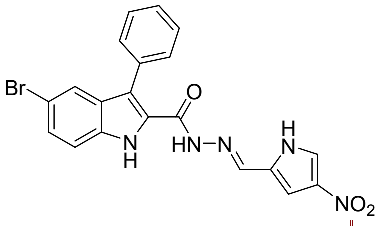

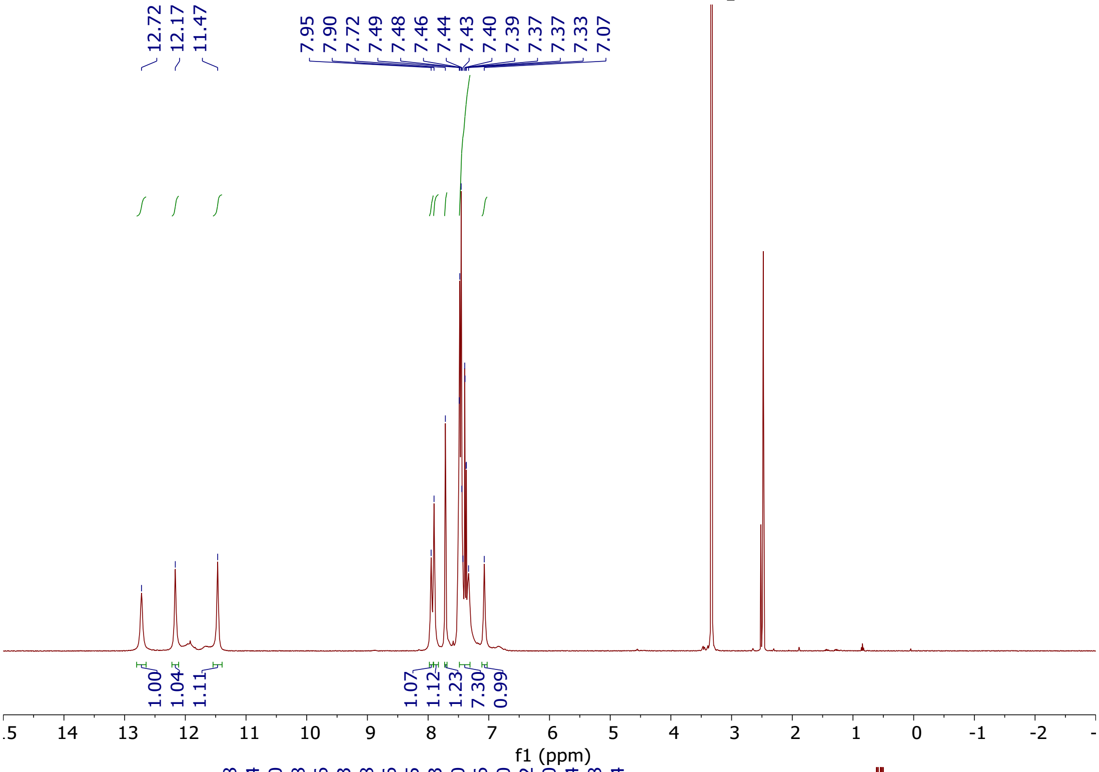

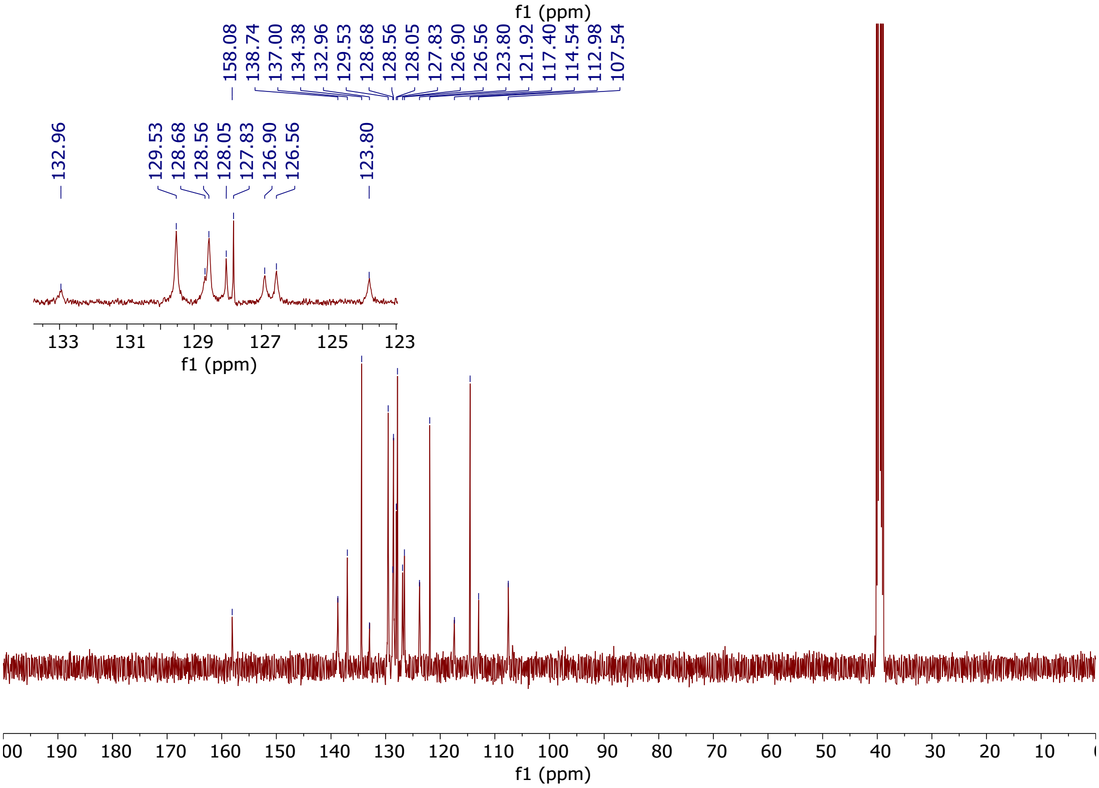

(*E*)-5-bromo-*N*'-((3,5-dimethyl-4-nitro-1*H*-pyrrol-2-yl)methylene)-3-phenyl-1*H*-indole-2-carbohydrazide (3r) spectra in dmso-d_6

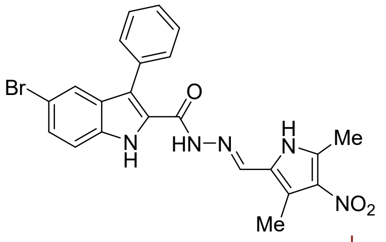

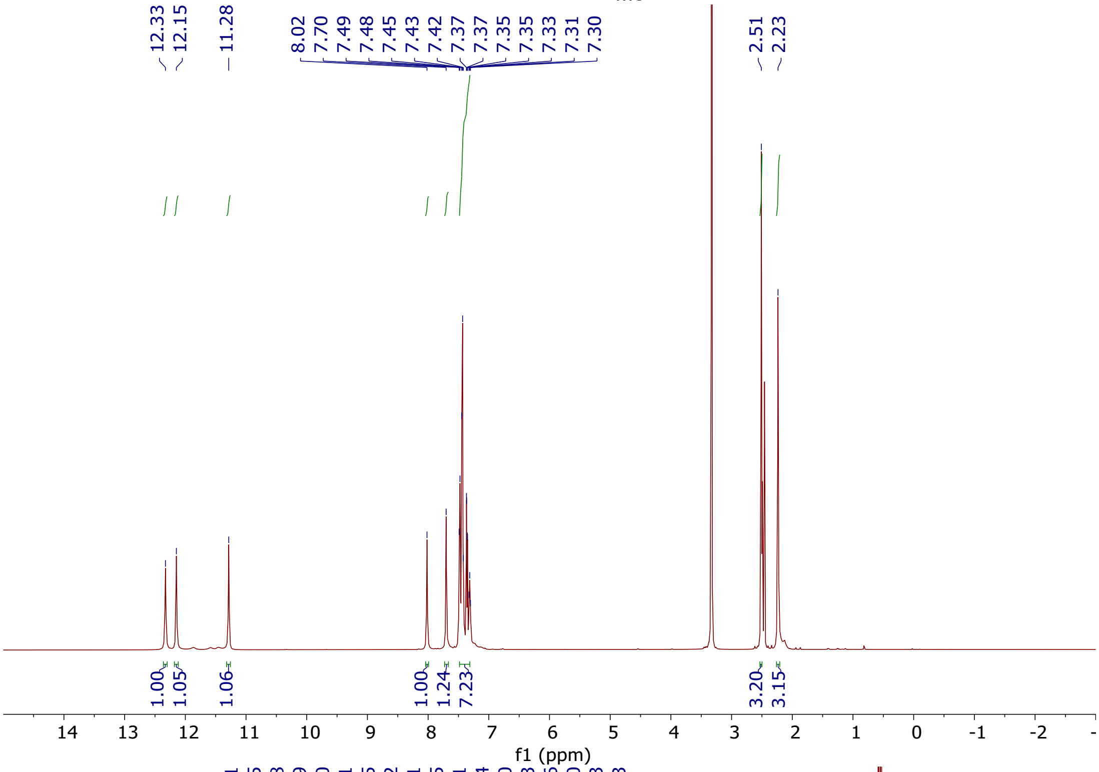

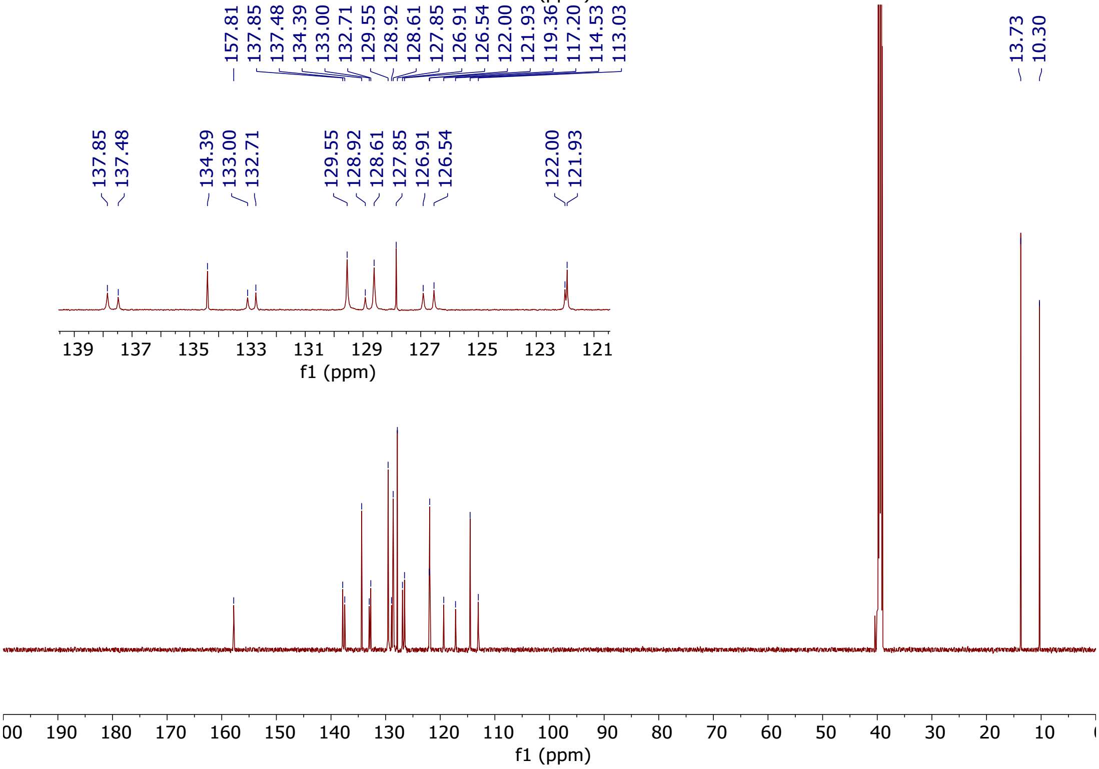

(*E*)-5-bromo-*N*'-((5-ethyl-1*H*-pyrrol-2-yl)methylene)-3-phenyl-1*H*-indole-2-carbohydrazide (3s) spectra in dmso-d$_6$

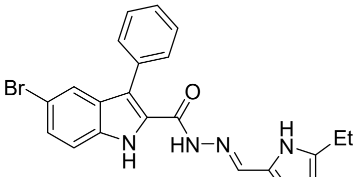

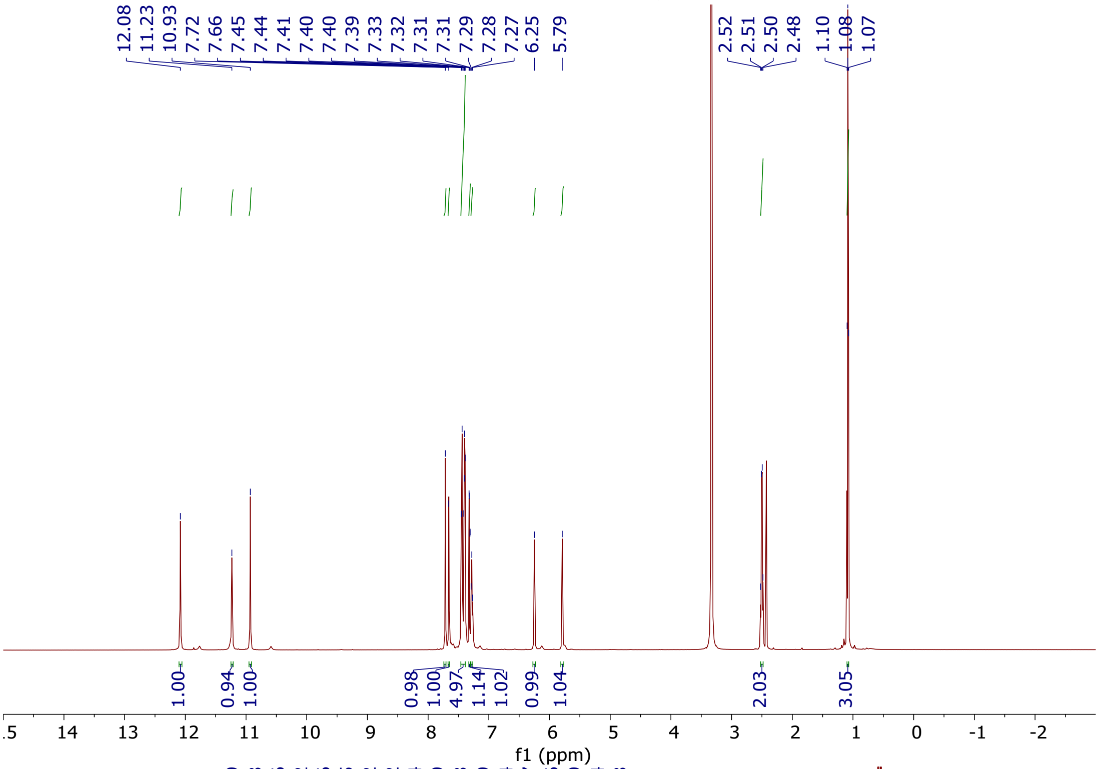

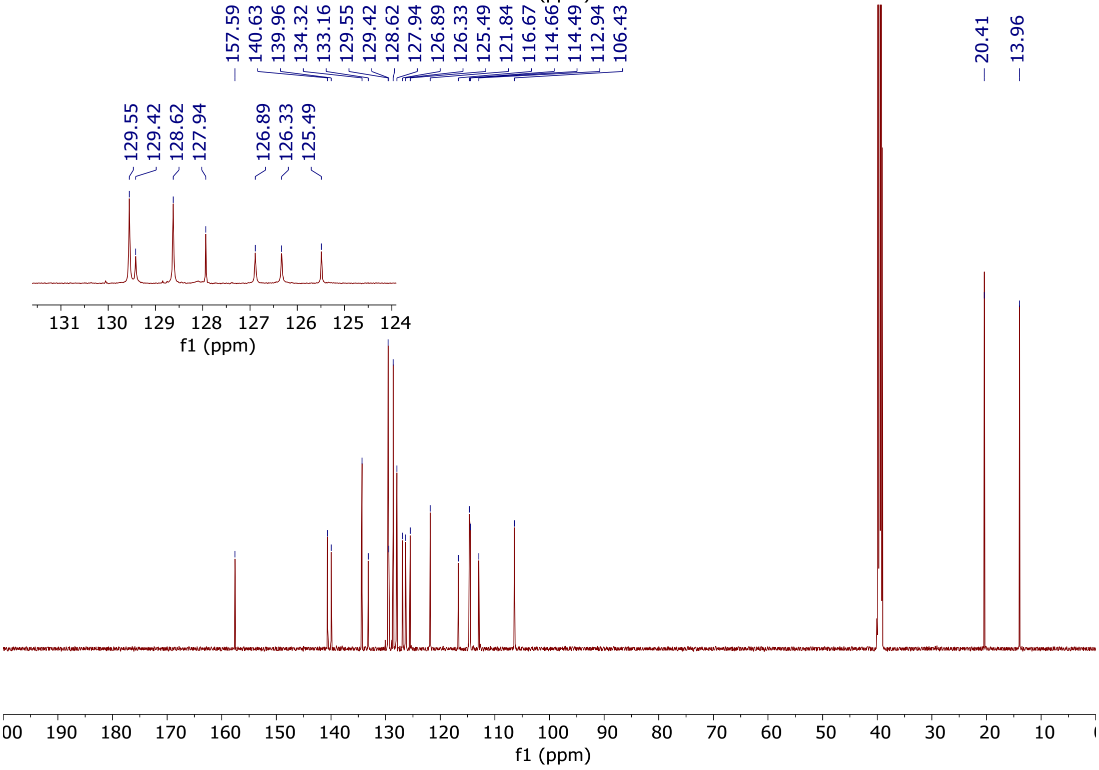

(*E*)-5-bromo-*N*'-((4-ethyl-1*H*-pyrrol-2-yl)methylene)-3-phenyl-1*H*-indole-2-carbohydrazide (3t) spectra in dmso-d$_6$

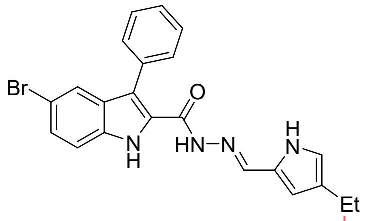

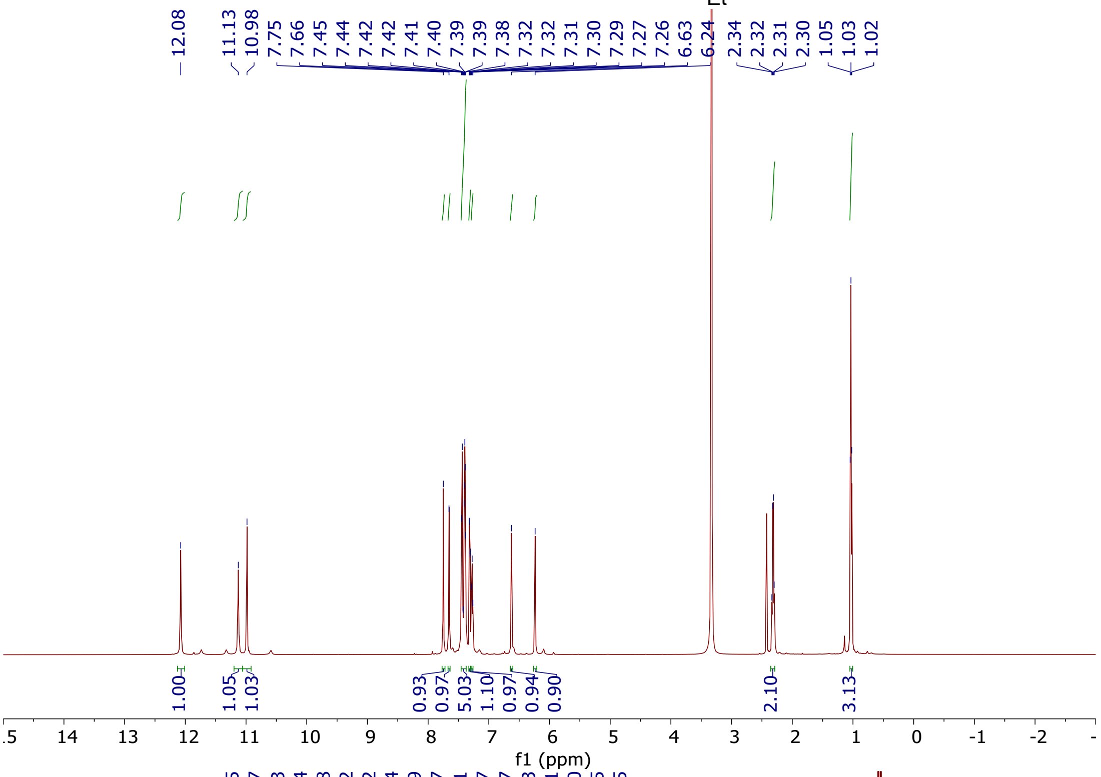

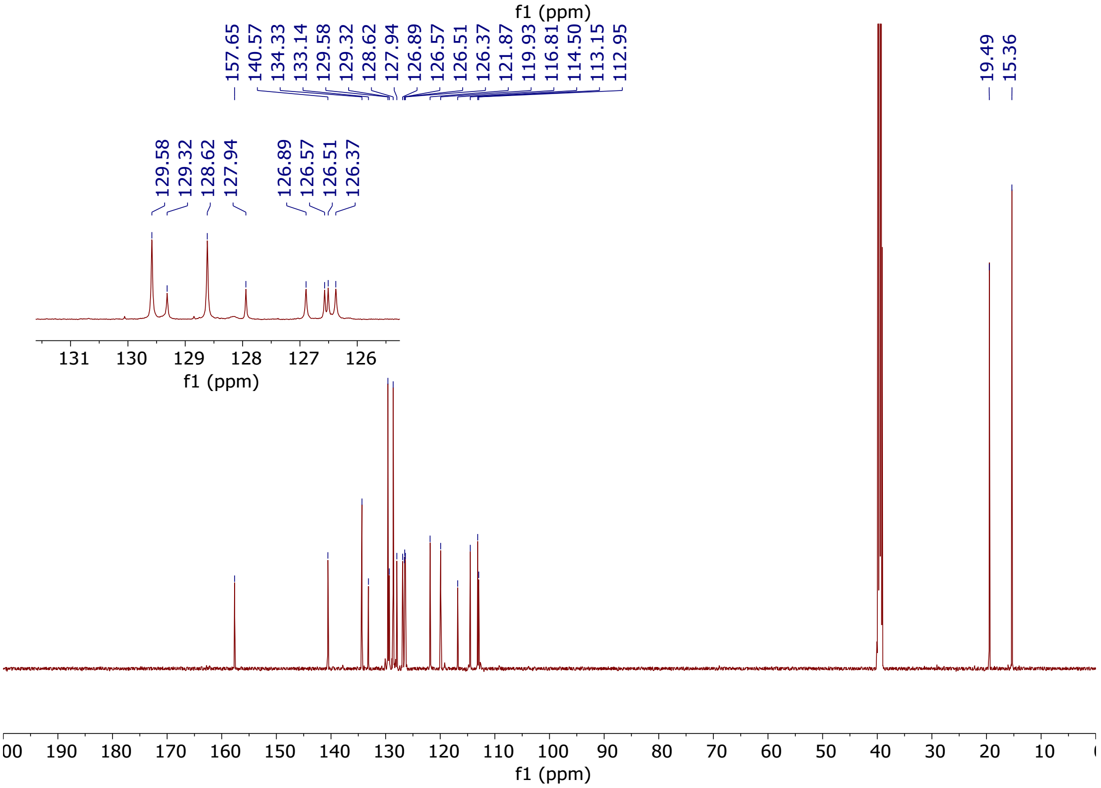

(*E*)-5-bromo-*N*'-((3-ethyl-1*H*-pyrrol-2-yl)methylene)-3-phenyl-1*H*-indole-2-carbohydrazide (3u) spectra in dmso-d$_6$

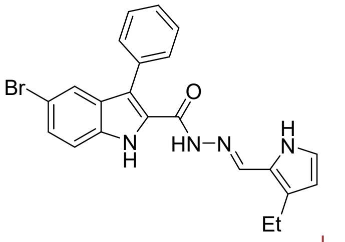

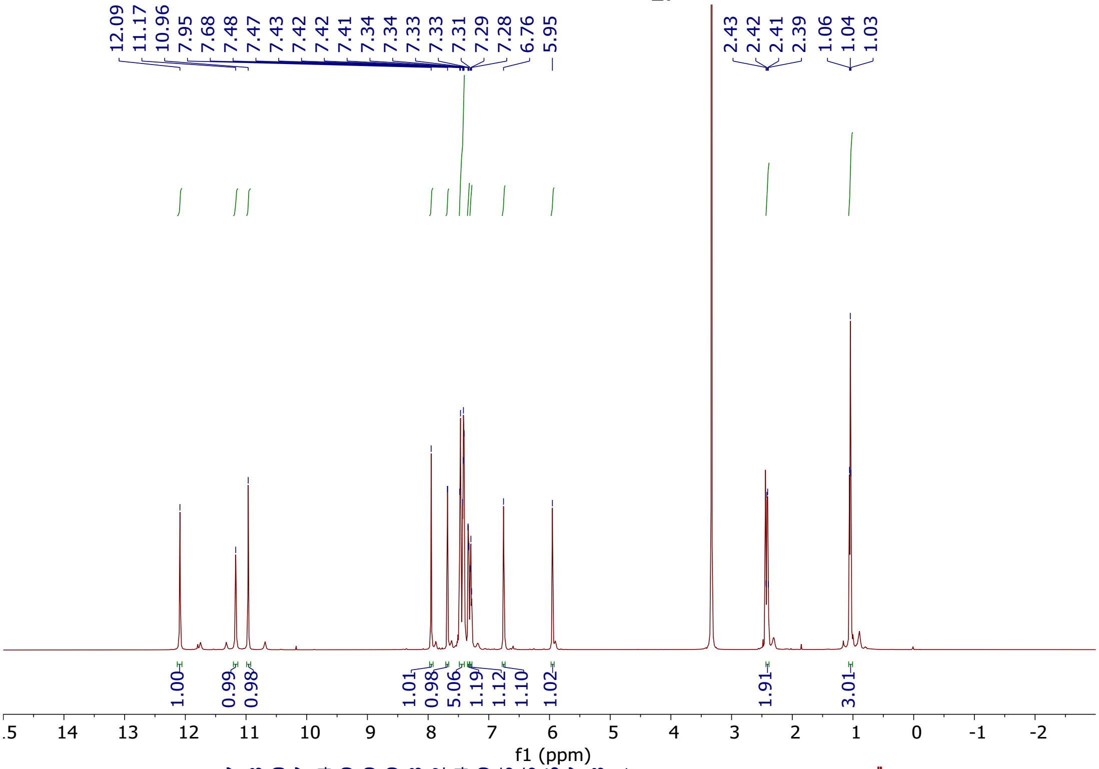

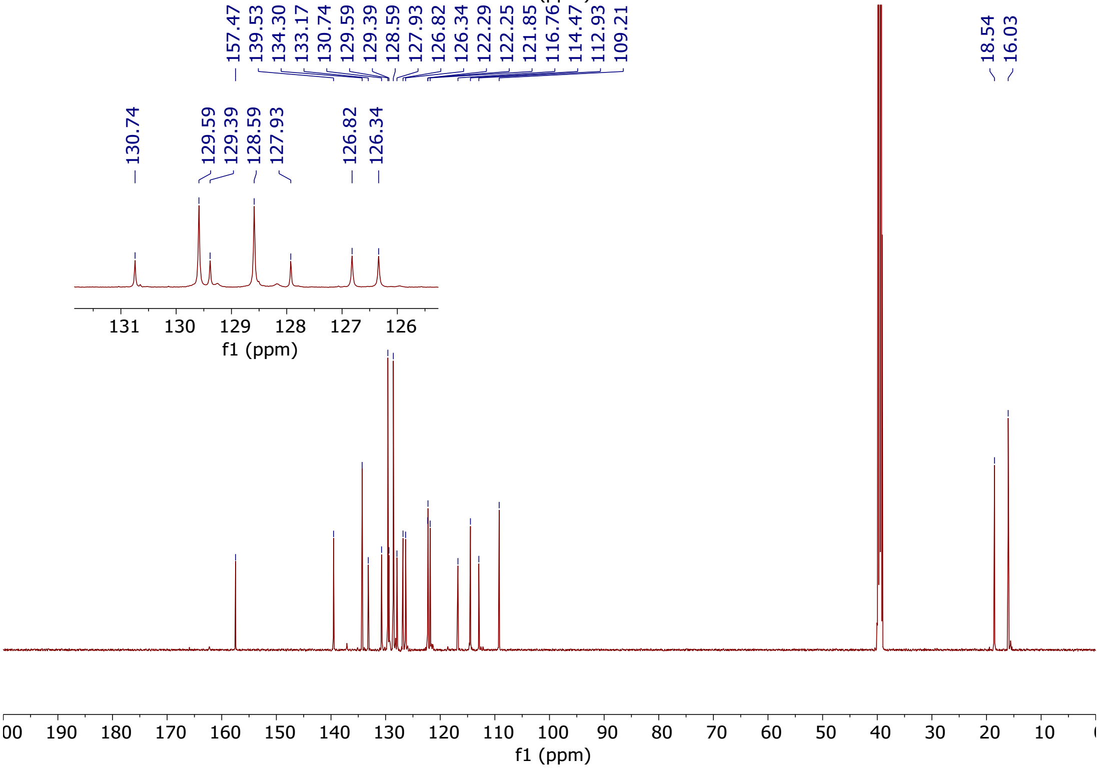

Ethyl (*E*)-4-((2-(5-bromo-3-phenyl-1*H*-indole-2-carbonyl)hydrazono)methyl)-1*H*-pyrrole-2-carboxylate (3v) spectra in dmso-d$_6$

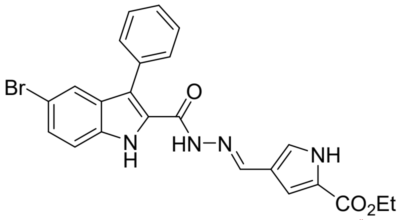

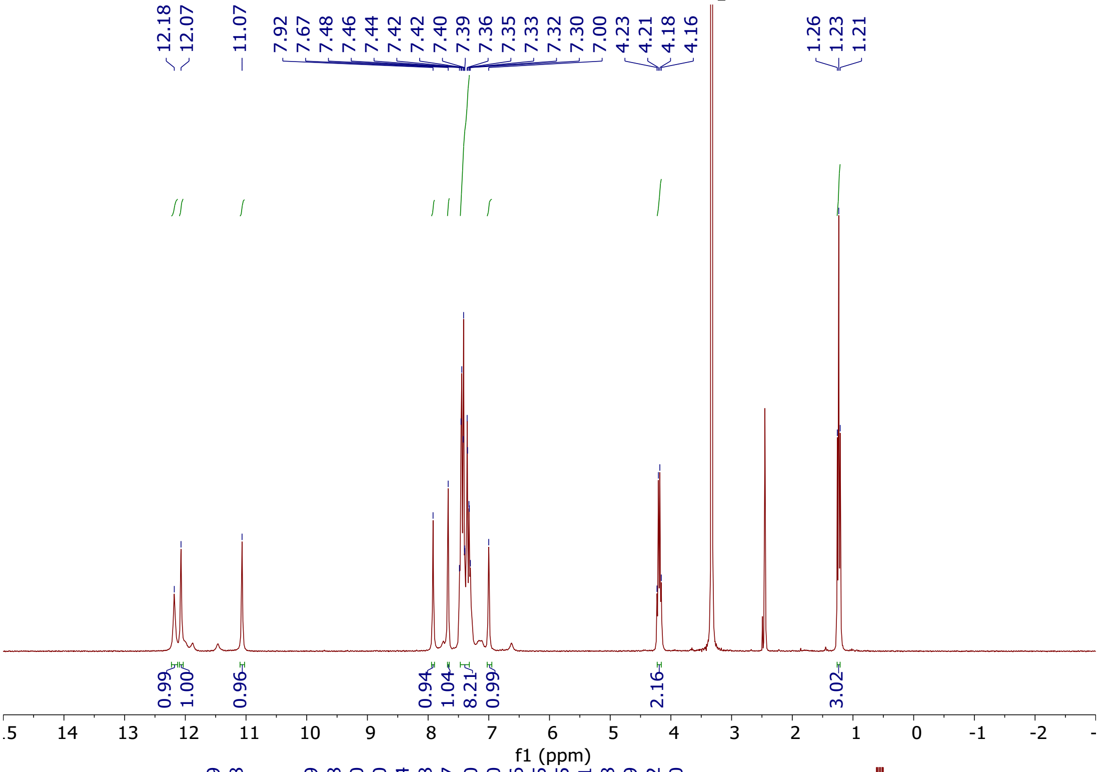

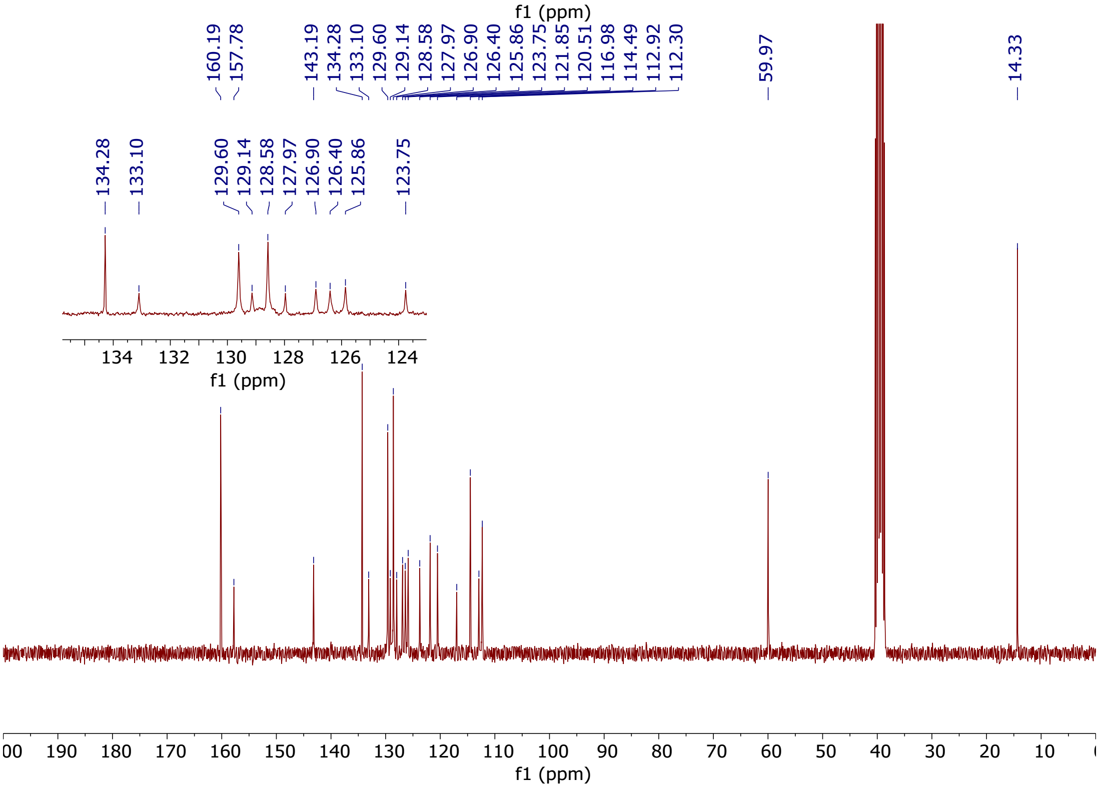

(*E*)-4-((2-(5-bromo-3-phenyl-1*H*-indole-2-carbonyl)hydrazono)methyl)-1*H*-pyrrole-2-carboxylic acid (3w) spectra in dmso-d_6

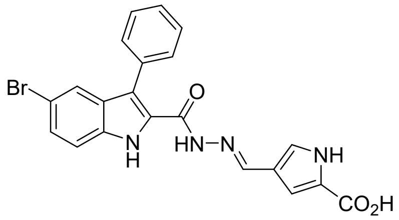

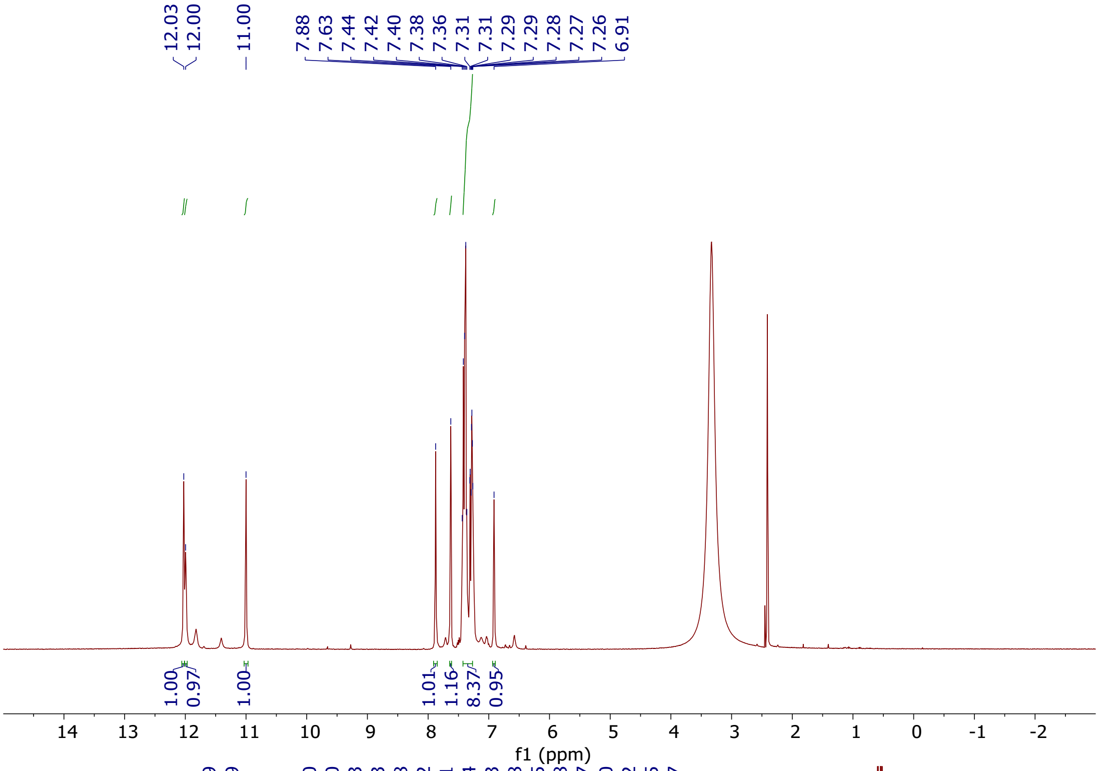

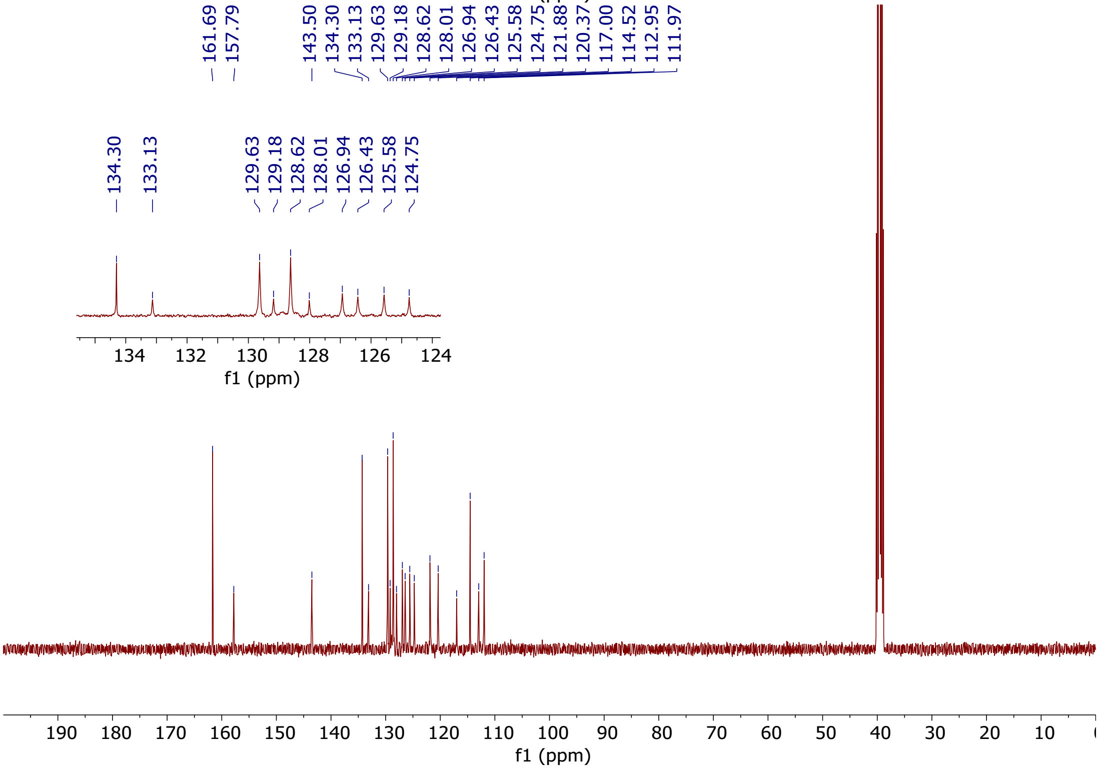

(*E*)-5-bromo-*N'*-((5-(morpholine-4-carbonyl)-1*H*-pyrrol-3-yl)methylene)-3-phenyl-1*H*-indole-2-carbohydrazide (3x) spectra in dmso-d_6

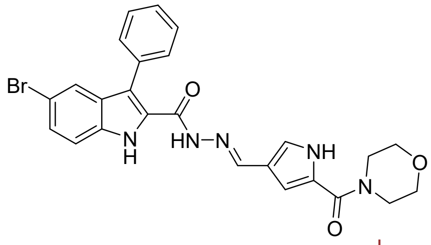

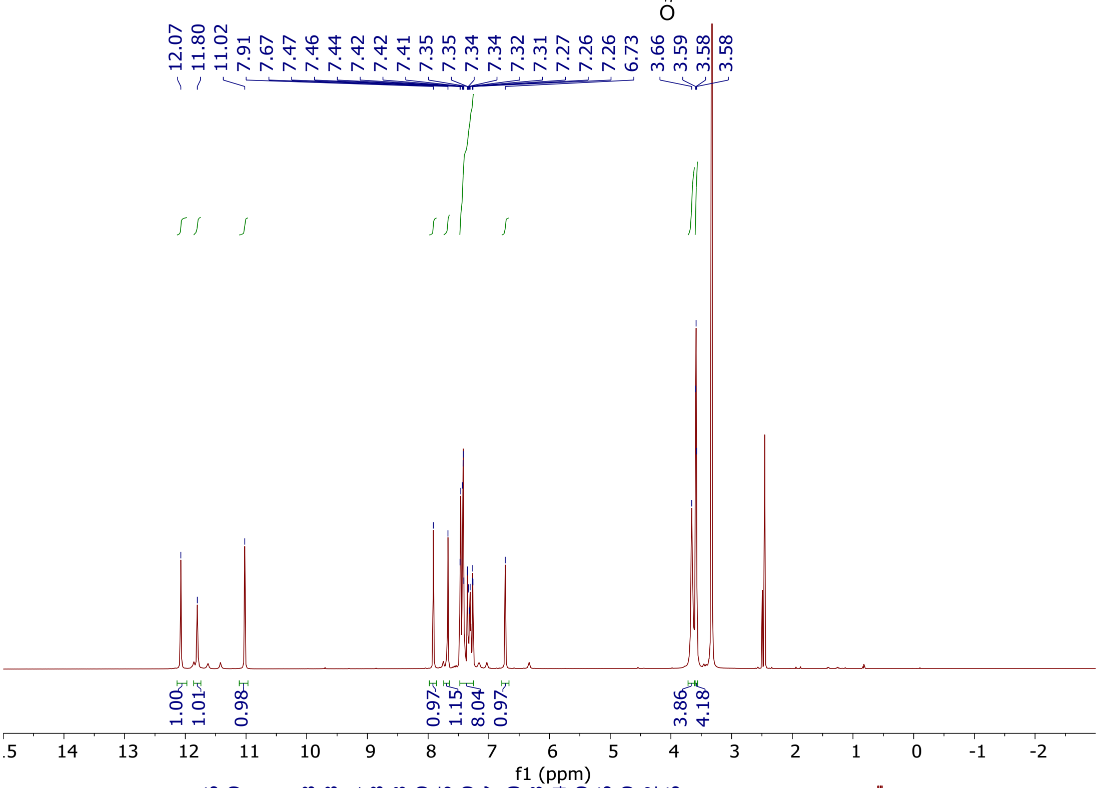

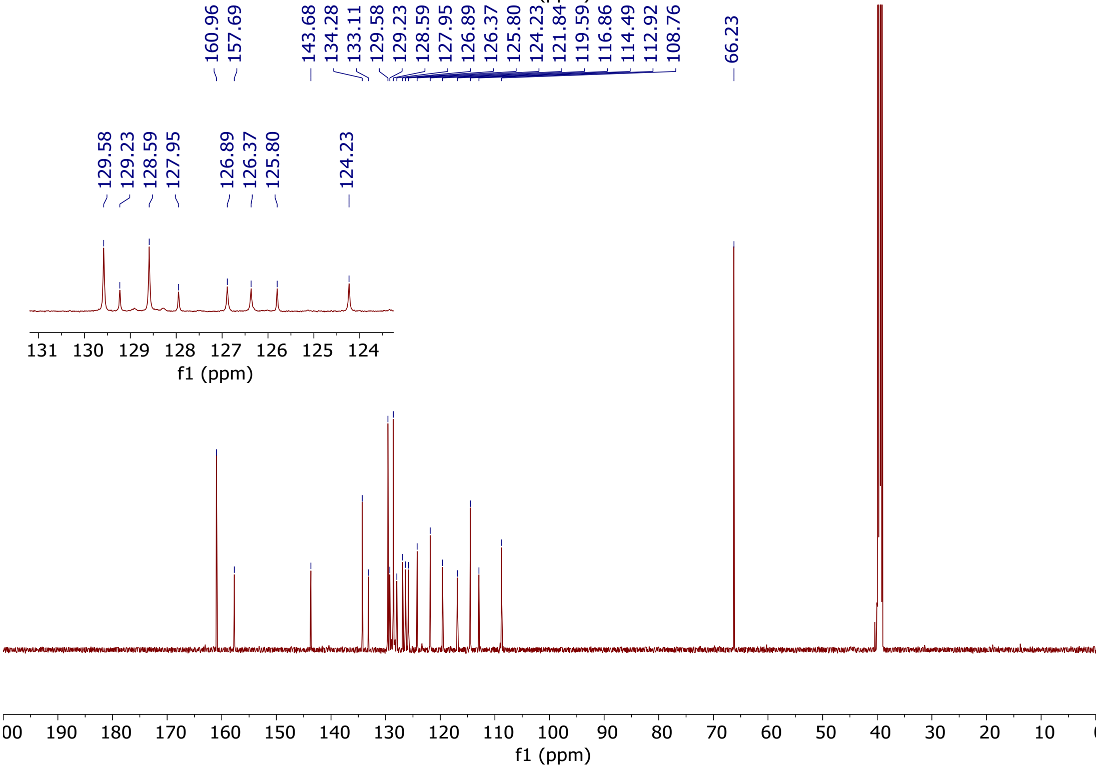

5-bromo-3-phenyl-1*H*-2-carbohydrazide (5) spectra in dmso-d$_6$

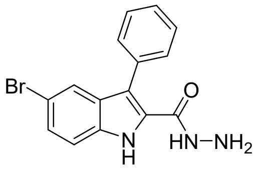

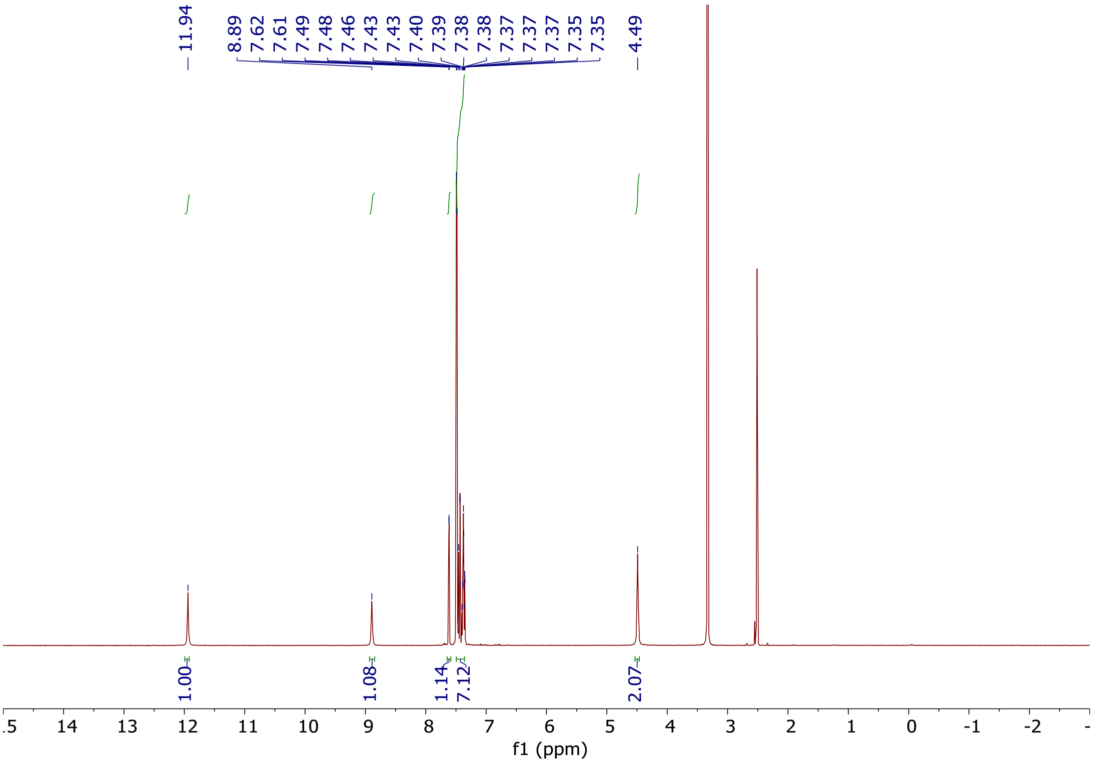

Figure S1. NCI60 one dose screen

Compound **3a**

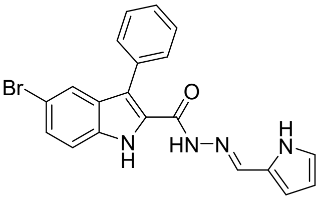

Developmental Therapeutics Program

One Dose Mean Graph

**NSC:** D-832482 / 1	**Conc:** 1.00E-5 Molar	**Test Date:** Aug 30, 2021
**Experiment ID:** 2108OS84		**Report Date:** Jan 25, 2022

Panel/Cell Line	Growth Percent
Leukemia	
CCRF-CEM	5.27
HL-60(TB)	-39.08
K-562	3.06
MOLT-4	5.94
RPMI-8226	-1.05
SR	1.89
Non-Small Cell Lung Cancer	
A549/ATCC	17.57
EKVX	24.44
HOP-62	22.15
HOP-92	15.54
NCI-H226	35.92
NCI-H322M	32.40
NCI-H460	2.17
NCI-H522	-20.17
Colon Cancer	
COLO 205	6.05
HCC-2998	17.18
HCT-116	0.02
HCT-15	9.42
HT29	6.16
KM12	10.36
SW-620	22.82
CNS Cancer	
SF-268	25.31
SF-295	7.67
SF-539	14.23
SNB-19	30.60
SNB-75	27.28
U251	21.80
Melanoma	
MALME-3M	45.12
M14	13.17
MDA-MB-435	2.21
SK-MEL-2	31.95
SK-MEL-28	61.18
SK-MEL-5	-34.25
UACC-257	30.29
UACC-62	13.40
Ovarian Cancer	
IGROV1	8.52
OVCAR-3	22.19
OVCAR-4	33.23
OVCAR-5	43.70
OVCAR-8	12.98
NCI/ADR-RES	14.76
SK-OV-3	36.74
Renal Cancer	
786-0	13.74
ACHN	12.18
CAKI-1	21.18
RXF 393	53.18
SN12C	28.66
TK-10	44.45
UO-31	12.28
Prostate Cancer	
PC-3	15.17
DU-145	20.63
Breast Cancer	
MCF7	3.98
MDA-MB-231/ATCC	13.98
HS 578T	29.49
BT-549	-12.45
T-47D	14.10
MDA-MB-468	-0.63
Mean	15.97
Delta	55.05
Range	100.26

Mean Growth Percent - Growth Percent

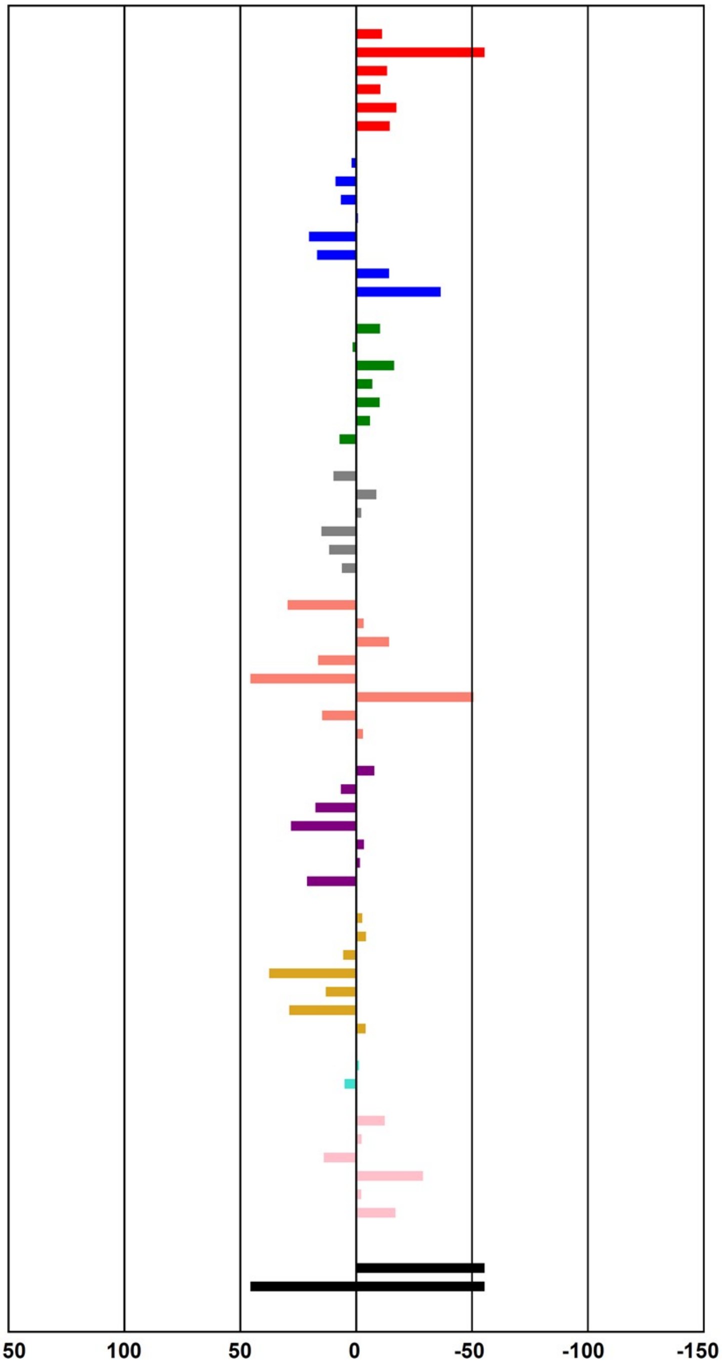

Compound **3b**

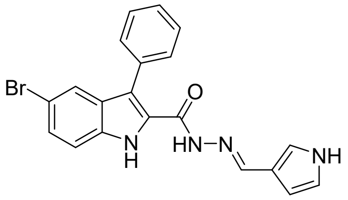

Developmental Therapeutics Program
One Dose Mean Graph

NSC: D-843103 / 1 | **Conc:** 1.00E-5 Molar | **Test Date:** Mar 20, 2023

Experiment ID: 2303OS10 | **Report Date:** Apr 24, 2023

Panel/Cell Line	Growth Percent
Leukemia	
CCRF-CEM	10.60
HL-60(TB)	-8.20
K-562	7.33
MOLT-4	13.28
RPMI-8226	11.94
SR	11.85
Non-Small Cell Lung Cancer	
A549/ATCC	27.71
EKVX	-3.01
HOP-62	50.21
HOP-92	55.51
NCI-H226	52.94
NCI-H23	15.96
NCI-H460	3.20
NCI-H522	-25.46
Colon Cancer	
COLO 205	-10.61
HCC-2998	20.29
HCT-116	8.13
HCT-15	7.26
HT29	8.01
KM12	8.22
SW-620	23.40
CNS Cancer	
SF-268	20.86
SF-295	4.74
SF-539	-20.84
SNB-19	43.16
SNB-75	18.40
U251	18.76
Melanoma	
LOX IMVI	12.64
MALME-3M	16.56
M14	34.26
MDA-MB-435	-29.41
SK-MEL-2	50.14
SK-MEL-28	48.99
SK-MEL-5	-9.09
UACC-257	67.41
UACC-62	43.83
Ovarian Cancer	
IGROV1	38.91
OVCAR-3	-2.36
OVCAR-4	53.80
OVCAR-5	32.37
OVCAR-8	6.73
NCI/ADR-RES	-35.86
SK-OV-3	57.14
Renal Cancer	
786-0	49.27
A498	80.76
ACHN	34.08
CAKI-1	30.90
RXF 393	-0.72
SN12C	15.23
TK-10	57.10
UO-31	26.66
Prostate Cancer	
PC-3	20.28
DU-145	8.66
Breast Cancer	
MCF7	10.62
MDA-MB-231/ATCC	33.30
HS 578T	5.02
BT-549	-6.95
T-47D	71.36
MDA-MB-468	-4.53
Mean	20.18
Delta	56.04
Range	116.62

Mean Growth Percent - Growth Percent

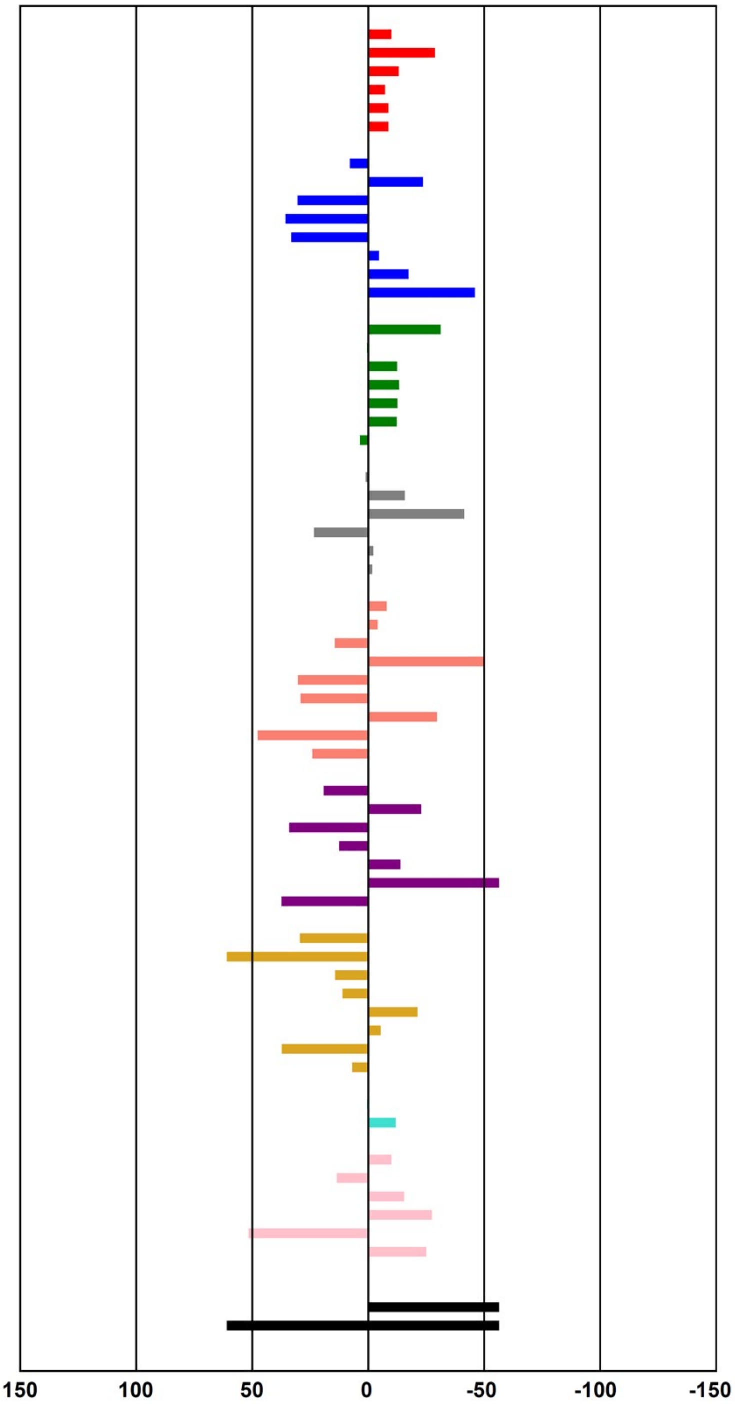

Compound **3c**

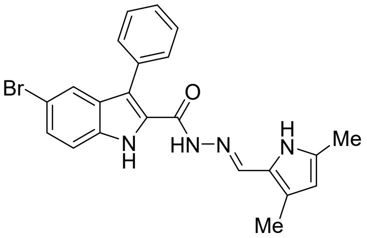

Developmental Therapeutics Program | **NSC:** D-843105 / 1 | **Conc:** 1.00E-5 Molar | **Test Date:** Mar 20, 2023

One Dose Mean Graph | **Experiment ID:** 2303OS10 | **Report Date:** Apr 24, 2023

Panel/Cell Line	Growth Percent
Leukemia	
CCRF-CEM	10.38
HL-60(TB)	-29.94
K-562	1.83
MOLT-4	-4.88
RPMI-8226	9.62
SR	-3.01
Non-Small Cell Lung Cancer	
A549/ATCC	16.15
HOP-62	22.47
HOP-92	10.79
NCI-H226	18.17
NCI-H23	0.47
NCI-H460	1.40
NCI-H522	-13.81
Colon Cancer	
COLO 205	2.58
HCC-2998	17.84
HCT-116	6.28
HCT-15	6.27
HT29	12.39
KM12	2.51
SW-620	13.08
CNS Cancer	
SF-268	20.71
SF-295	3.24
SF-539	7.09
SNB-19	19.66
SNB-75	-0.96
U251	7.16
Melanoma	
LOX IMVI	-0.57
MALME-3M	2.47
M14	29.28
MDA-MB-435	8.60
SK-MEL-2	21.05
SK-MEL-28	29.49
SK-MEL-5	-12.99
UACC-257	30.88
UACC-62	14.14
Ovarian Cancer	
IGROV1	27.09
OVCAR-3	20.93
OVCAR-4	17.30
OVCAR-5	29.41
OVCAR-8	8.09
NCI/ADR-RES	-27.09
SK-OV-3	21.34
Renal Cancer	
786-0	19.96
A498	37.96
ACHN	10.25
CAKI-1	18.62
RXF 393	-11.39
SN12C	-33.61
TK-10	37.26
UO-31	12.36
Prostate Cancer	
PC-3	7.50
DU-145	25.93
Breast Cancer	
MCF7	-20.33
MDA-MB-231/ATCC	16.84
HS 578T	1.68
BT-549	12.45
T-47D	11.09
MDA-MB-468	-8.76
Mean	8.87
Delta	42.48
Range	71.57

Mean Growth Percent - Growth Percent

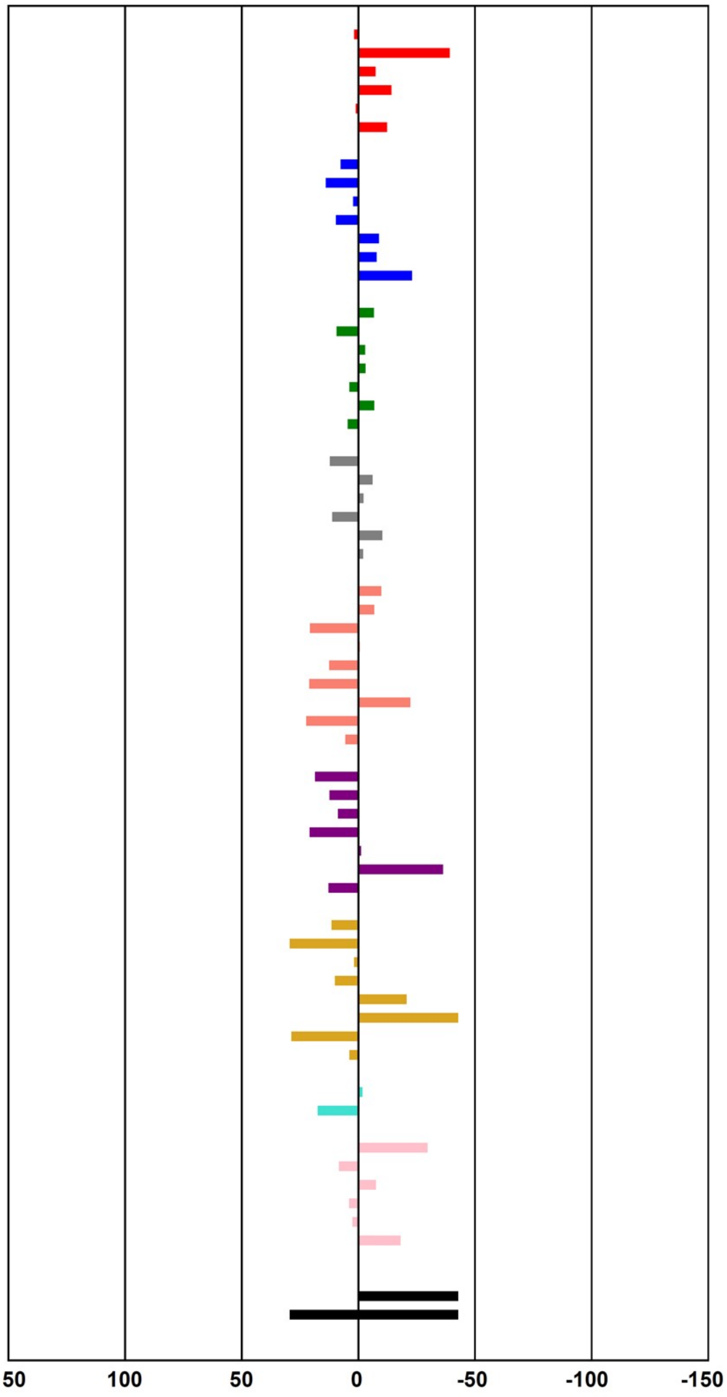

Compound **3d**

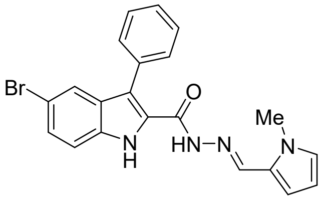

Developmental Therapeutics Program

One Dose Mean Graph

NSC: D-843112 / 1 | **Conc:** 1.00E-5 Molar | **Test Date:** Mar 20, 2023

Experiment ID: 2303OS10 | **Report Date:** Apr 24, 2023

Panel/Cell Line	Growth Percent
Leukemia	
CCRF-CEM	18.03
HL-60(TB)	6.85
K-562	15.05
MOLT-4	26.30
RPMI-8226	28.30
SR	23.61
Non-Small Cell Lung Cancer	
A549/ATCC	34.63
HOP-62	45.26
HOP-92	20.63
NCI-H226	29.07
NCI-H23	34.17
NCI-H460	10.45
NCI-H522	-30.64
Colon Cancer	
COLO 205	6.60
HCC-2998	2.95
HCT-116	21.30
HCT-15	3.92
HT29	7.45
KM12	13.73
SW-620	26.74
CNS Cancer	
SF-268	27.47
SF-295	1.97
SF-539	-13.62
SNB-19	46.15
SNB-75	1.33
U251	22.99
Melanoma	
LOX IMVI	22.45
MALME-3M	-26.15
M14	20.83
MDA-MB-435	-18.15
SK-MEL-2	12.80
SK-MEL-28	60.90
SK-MEL-5	10.46
UACC-257	59.20
UACC-62	48.50
Ovarian Cancer	
IGROV1	48.50
OVCAR-3	-8.15
OVCAR-4	46.22
OVCAR-5	43.23
OVCAR-8	21.14
NCI/ADR-RES	-14.62
SK-OV-3	48.79
Renal Cancer	
786-0	58.28
A498	86.25
ACHN	10.48
CAKI-1	43.42
RXF 393	-12.24
SN12C	26.90
TK-10	45.55
UO-31	38.65
Prostate Cancer	
PC-3	33.52
DU-145	15.05
Breast Cancer	
MCF7	7.52
MDA-MB-231/ATCC	44.14
HS 578T	-0.04
BT-549	41.84
T-47D	23.18
MDA-MB-468	-5.26
Mean	21.79
Delta	52.43
Range	116.89

Mean Growth Percent - Growth Percent

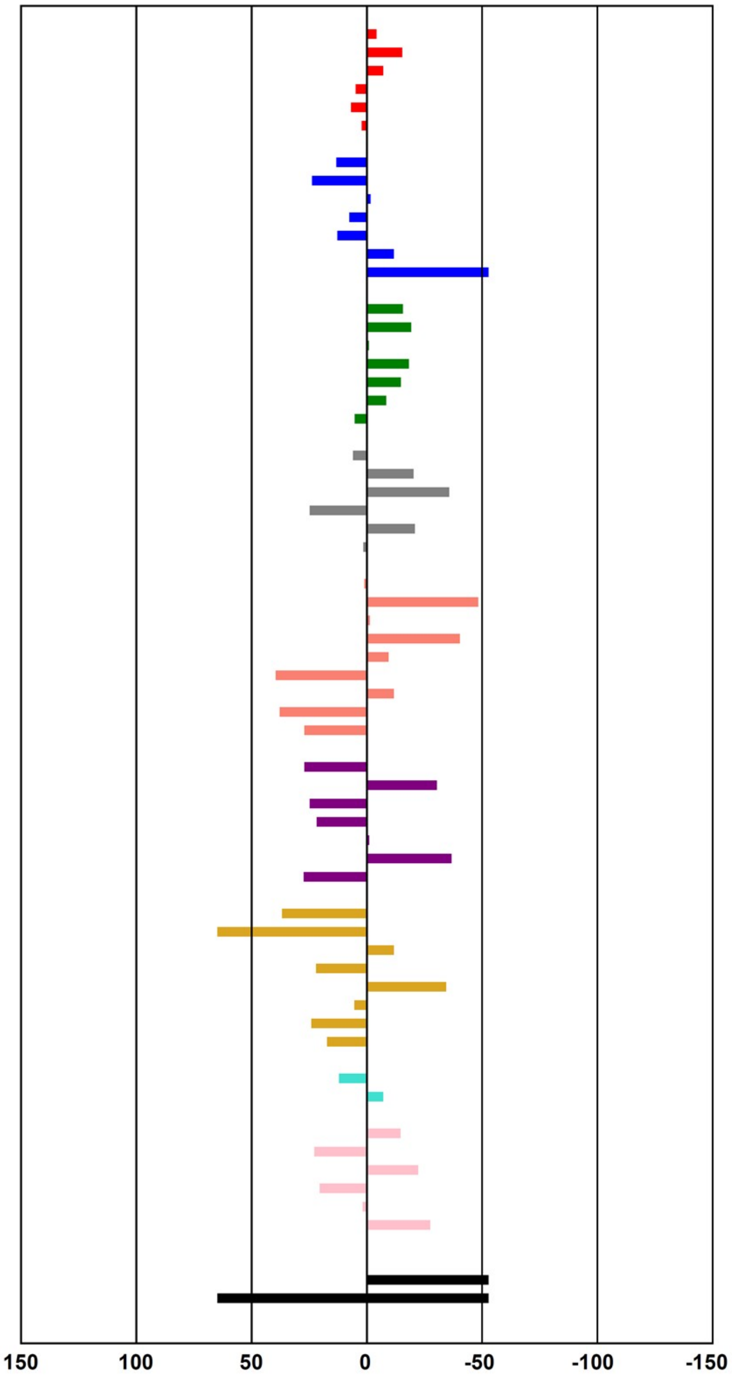

Compound **3e**

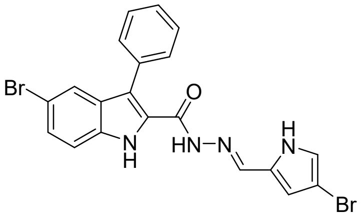

Developmental Therapeutics Program

One Dose Mean Graph

NSC: D-843104 / 1 | **Conc:** 1.00E-5 Molar | **Test Date:** Mar 20, 2023

Experiment ID: 2303OS10 | **Report Date:** Apr 24, 2023

Panel/Cell Line	Growth Percent
Leukemia	
CCRF-CEM	0.95
HL-60(TB)	-21.64
K-562	5.69
MOLT-4	-11.31
RPMI-8226	-16.62
SR	-11.68
Non-Small Cell Lung Cancer	
A549/ATCC	11.07
HOP-62	32.23
HOP-92	-5.68
NCI-H226	7.32
NCI-H23	1.42
NCI-H322M	3.94
NCI-H460	0.10
NCI-H522	-24.45
Colon Cancer	
COLO 205	0.52
HCC-2998	-24.60
HCT-116	4.80
HCT-15	-30.73
HT29	-4.75
KM12	-28.37
SW-620	7.06
CNS Cancer	
SF-268	14.42
SF-295	-11.98
SF-539	4.62
SNB-19	14.46
SNB-75	12.32
U251	0.87
Melanoma	
LOX IMVI	-75.05
MALME-3M	16.83
M14	18.97
MDA-MB-435	3.53
SK-MEL-2	12.17
SK-MEL-28	37.16
SK-MEL-5	-57.10
UACC-257	25.12
UACC-62	9.00
Ovarian Cancer	
IGROV1	14.45
OVCAR-3	-21.05
OVCAR-4	23.37
OVCAR-5	16.89
OVCAR-8	-8.45
NCI/ADR-RES	-41.21
SK-OV-3	41.32
Renal Cancer	
786-0	13.29
A498	42.05
ACHN	0.72
CAKI-1	4.62
RXF 393	-2.74
SN12C	-71.29
TK-10	22.83
UO-31	6.16
Prostate Cancer	
PC-3	11.08
DU-145	1.24
Breast Cancer	
MCF7	-5.01
MDA-MB-231/ATCC	2.84
HS 578T	0.06
BT-549	-27.80
T-47D	22.65
MDA-MB-468	-21.53
Mean	-.93
Delta	74.12
Range	117.10

Mean Growth Percent - Growth Percent

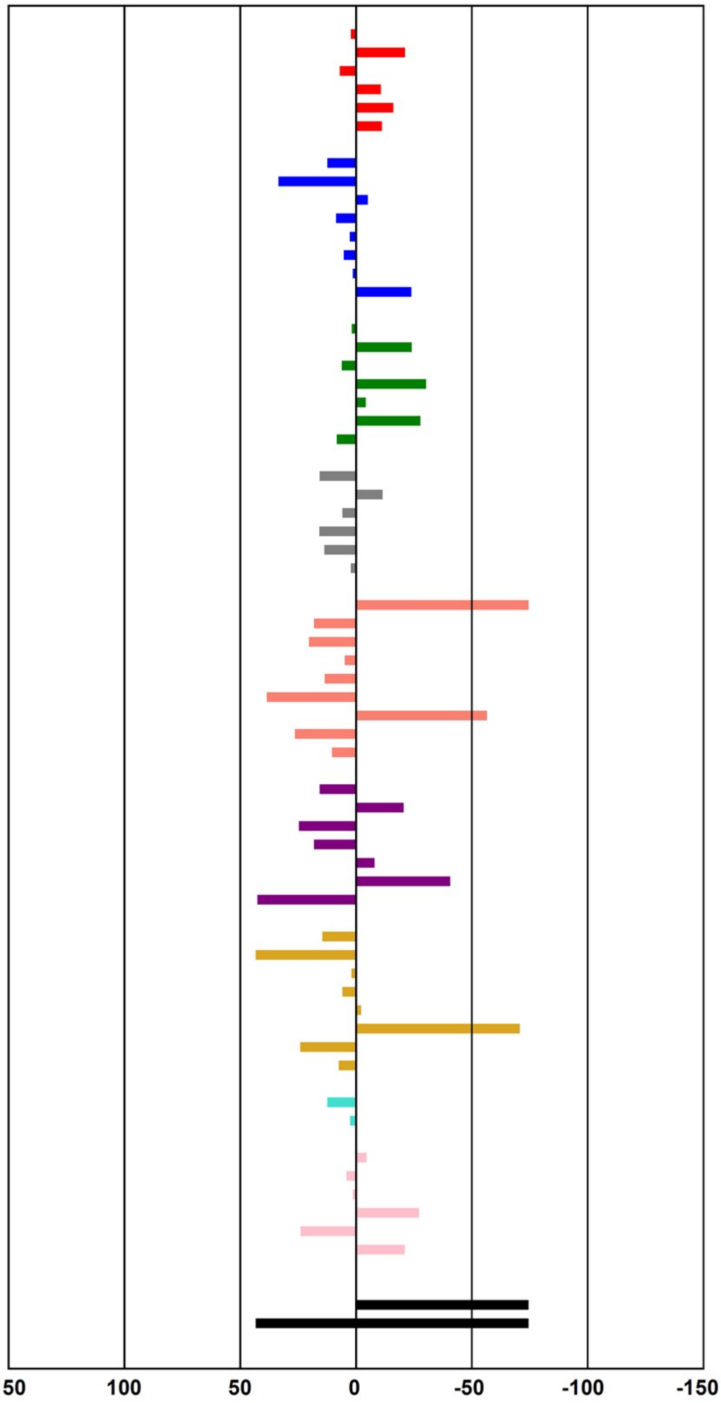

Compound **3f**

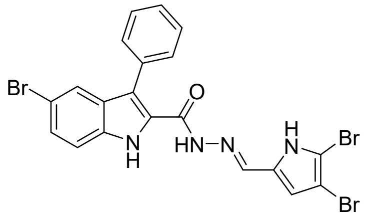

Developmental Therapeutics Program
One Dose Mean Graph

**NSC:** D-843111 / 1	**Conc:** 1.00E-5 Molar
**Test Date:** Mar 20, 2023	
**Experiment ID:** 2303OS10	**Report Date:** Apr 24, 2023

Panel/Cell Line	Growth Percent
**Leukemia**	
CCRF-CEM	13.52
HL-60(TB)	-6.85
K-562	6.49
MOLT-4	14.33
RPMI-8226	-0.74
SR	7.79
**Non-Small Cell Lung Cancer**	
A549/ATCC	13.01
HOP-62	22.37
HOP-92	10.06
NCI-H226	11.38
NCI-H23	4.96
NCI-H460	2.12
NCI-H522	-34.42
**Colon Cancer**	
COLO 205	-28.52
HCC-2998	-21.57
HCT-116	9.78
HCT-15	-14.96
HT29	-15.22
KM12	-27.44
SW-620	12.40
**CNS Cancer**	
SF-268	15.02
SF-295	-38.84
SF-539	-22.71
SNB-19	17.97
SNB-75	1.02
U251	0.83
**Melanoma**	
LOX IMVI	-48.20
MALME-3M	-3.27
M14	17.69
MDA-MB-435	2.21
SK-MEL-2	2.57
SK-MEL-28	32.62
SK-MEL-5	-65.05
UACC-257	14.22
UACC-62	13.49
**Ovarian Cancer**	
IGROV1	12.33
OVCAR-3	-24.68
OVCAR-4	16.63
OVCAR-5	28.17
OVCAR-8	0.70
NCI/ADR-RES	-27.98
SK-OV-3	35.63
**Renal Cancer**	
786-0	16.43
A498	36.83
ACHN	3.38
CAKI-1	11.17
RXF 393	-10.19
SN12C	-11.26
TK-10	26.17
UO-31	7.53
**Prostate Cancer**	
PC-3	18.61
DU-145	5.80
**Breast Cancer**	
MCF7	0.50
MDA-MB-231/ATCC	18.99
HS 578T	-1.02
BT-549	-5.84
T-47D	10.76
MDA-MB-468	-16.08
Mean	1.22
Delta	66.27
Range	101.88

Mean Growth Percent - Growth Percent

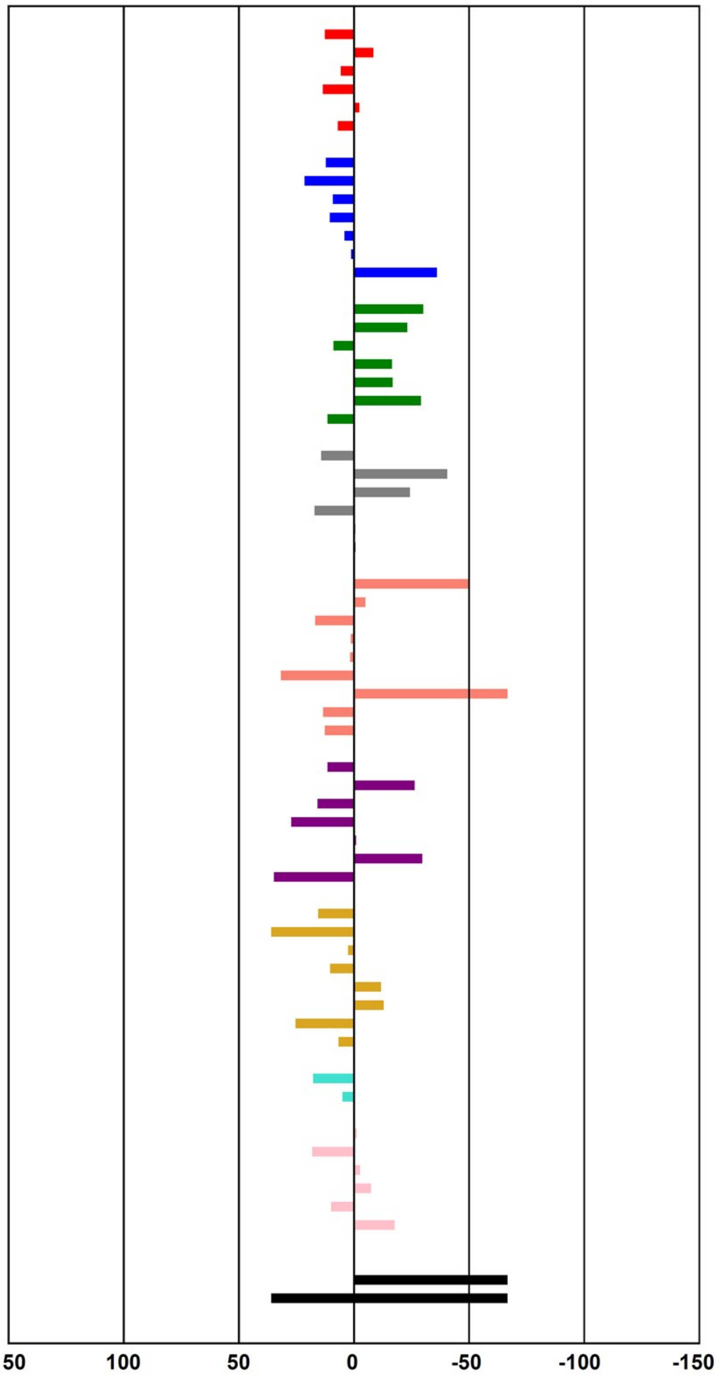

Compound **3g**

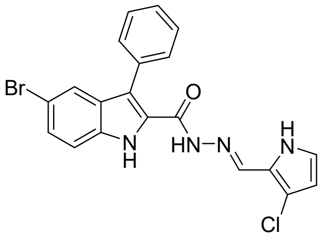

Developmental Therapeutics Program

One Dose Mean Graph

**NSC:** D-843106 / 1	**Conc:** 1.00E-5 Molar	**Test Date:** Mar 20, 2023
**Experiment ID:** 2303OS10		**Report Date:** Apr 24, 2023

**Panel/Cell Line**	**Growth Percent**
Leukemia	
CCRF-CEM	10.40
HL-60(TB)	-8.65
K-562	7.66
MOLT-4	9.70
RPMI-8226	1.80
SR	5.05
Non-Small Cell Lung Cancer	
A549/ATCC	18.82
HOP-62	28.21
HOP-92	18.13
NCI-H226	12.23
NCI-H23	8.71
NCI-H460	4.55
NCI-H522	-22.86
Colon Cancer	
COLO 205	0.60
HCC-2998	-8.53
HCT-116	8.72
HCT-15	-1.50
HT29	3.81
KM12	-10.17
SW-620	21.97
CNS Cancer	
SF-268	32.86
SF-295	-21.84
SF-539	-27.29
SNB-19	14.73
SNB-75	14.57
U251	8.58
Melanoma	
LOX IMVI	-70.73
MALME-3M	6.81
M14	22.29
MDA-MB-435	8.63
SK-MEL-2	18.55
SK-MEL-28	12.59
SK-MEL-5	-61.99
UACC-257	39.15
UACC-62	-11.01
Ovarian Cancer	
IGROV1	8.77
OVCAR-3	10.19
OVCAR-4	28.65
OVCAR-5	7.21
OVCAR-8	0.03
NCI/ADR-RES	-34.00
SK-OV-3	27.44
Renal Cancer	
786-0	11.77
A498	40.72
ACHN	-17.19
CAKI-1	7.75
RXF 393	7.75
SN12C	-75.52
TK-10	31.50
UO-31	6.74
Prostate Cancer	
PC-3	17.16
DU-145	3.21
Breast Cancer	
MCF7	1.43
MDA-MB-231/ATCC	-3.78
HS 578T	11.25
BT-549	-8.74
T-47D	29.26
MDA-MB-468	-18.41
Mean	3.24
Delta	78.76
Range	116.24

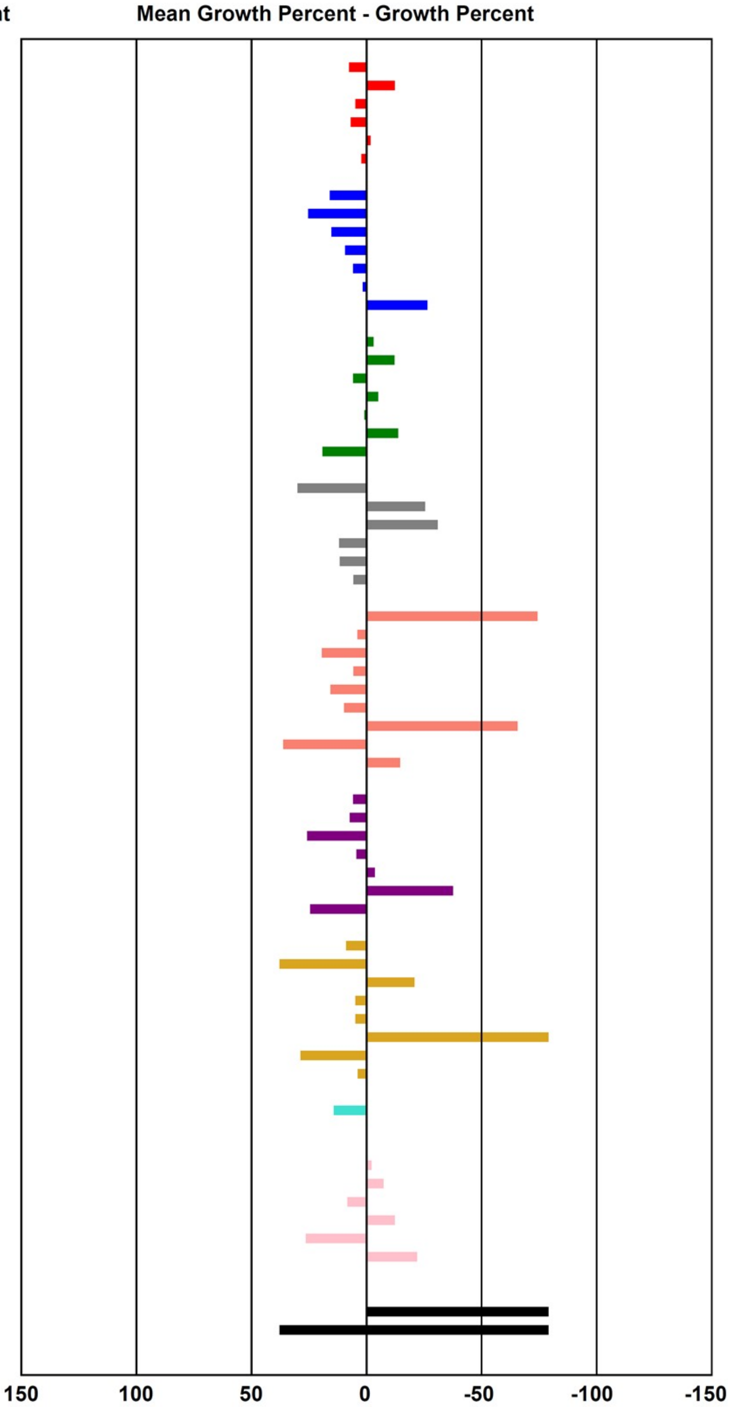

Compound **3h**

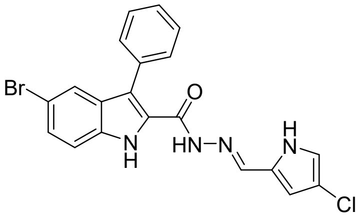

Developmental Therapeutics Program

One Dose Mean Graph

**NSC:** D-845424 / 1	**Conc:** 1.00E-5 Molar
**Test Date:** Jun 05, 2023	**Experiment ID:** 2306OS32
**Report Date:** Jul 16, 2023	

Panel/Cell Line	Growth Percent
Leukemia	
CCRF-CEM	1.71
HL-60(TB)	-30.35
K-562	1.36
MOLT-4	-0.92
RPMI-8226	-17.10
SR	-15.99
Non-Small Cell Lung Cancer	
A549/ATCC	8.30
EKVX	2.15
HOP-62	-9.31
HOP-92	-10.85
NCI-H226	-12.85
NCI-H23	-4.40
NCI-H322M	21.03
NCI-H460	1.89
NCI-H522	-33.46
Colon Cancer	
COLO 205	-46.39
HCC-2998	-33.93
HCT-116	5.55
HCT-15	-46.34
HT29	-64.25
KM12	-27.48
SW-620	8.98
CNS Cancer	
SF-268	7.92
SF-295	-6.11
SF-539	-22.00
SNB-19	8.05
SNB-75	14.94
U251	-65.46
Melanoma	
LOX IMVI	-34.80
MALME-3M	15.37
M14	8.46
MDA-MB-435	2.10
SK-MEL-2	12.51
SK-MEL-28	41.71
SK-MEL-5	-36.98
UACC-257	13.93
UACC-62	5.68
Ovarian Cancer	
IGROV1	6.30
OVCAR-3	-31.16
OVCAR-4	21.32
OVCAR-5	4.39
OVCAR-8	-46.38
NCI/ADR-RES	-35.91
SK-OV-3	22.53
Renal Cancer	
786-0	18.65
A498	48.15
ACHN	4.48
CAKI-1	12.61
RXF 393	1.42
SN12C	-27.51
TK-10	59.15
UO-31	1.50
Prostate Cancer	
PC-3	9.15
DU-145	-16.94
Breast Cancer	
MCF7	-13.44
MDA-MB-231/ATCC	-20.75
HS 578T	27.19
T-47D	18.46
MDA-MB-468	-13.22
Mean	-4.87
Delta	60.59
Range	124.61

Mean Growth Percent - Growth Percent

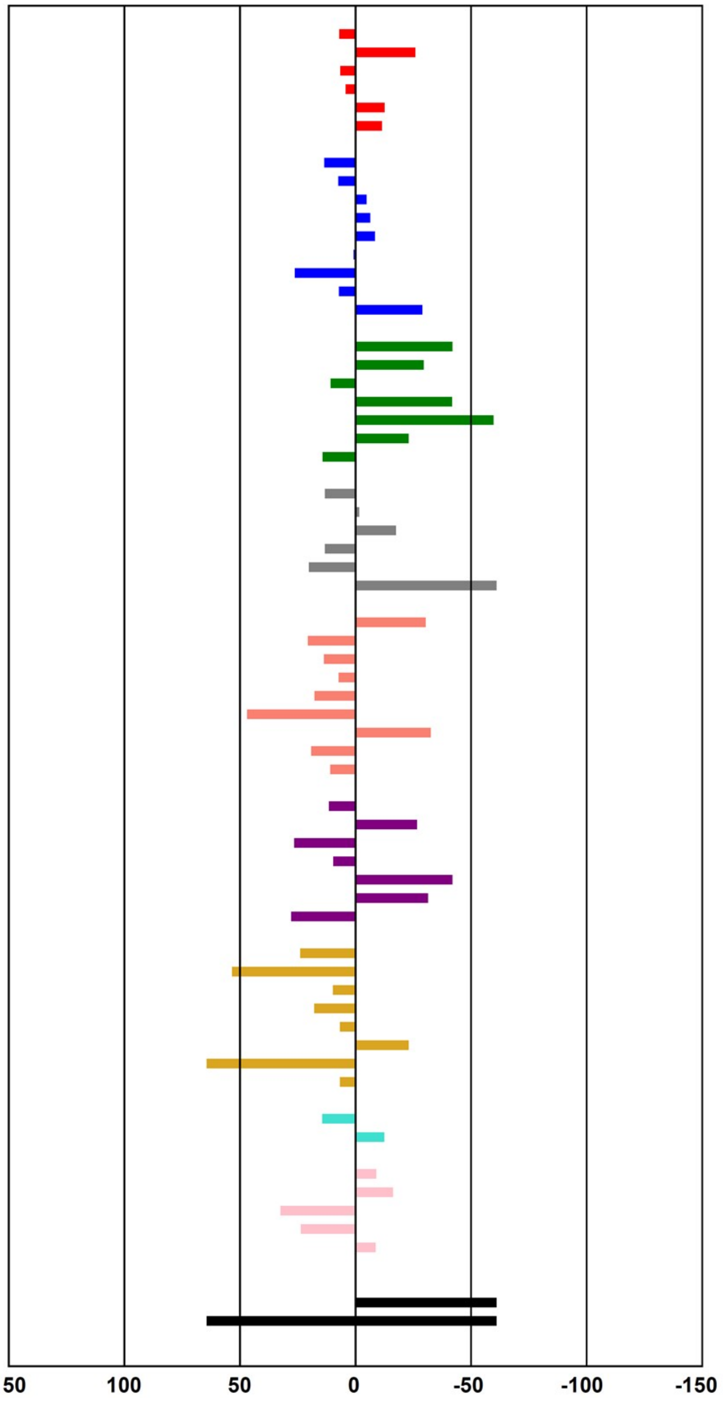

Compound **3i**

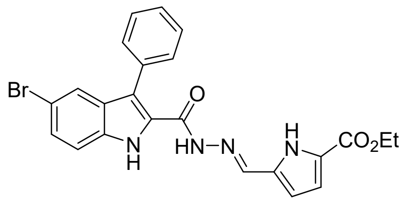

Developmental Therapeutics Program

One Dose Mean Graph

**NSC:** D-843107 / 1	**Conc:** 1.00E-5 Molar	**Test Date:** Mar 20, 2023	
**Experiment ID:** 2303OS10		**Report Date:** Apr 24, 2023	

Panel/Cell Line	Growth Percent
Leukemia	
CCRF-CEM	12.09
HL-60(TB)	1.13
K-562	10.45
MOLT-4	9.96
RPMI-8226	-0.39
SR	6.78
Non-Small Cell Lung Cancer	
A549/ATCC	27.57
HOP-62	51.78
HOP-92	37.24
NCI-H226	29.81
NCI-H23	16.20
NCI-H460	12.68
NCI-H522	-9.26
Colon Cancer	
COLO 205	9.53
HCC-2998	19.50
HCT-116	12.52
HCT-15	6.34
HT29	3.96
KM12	8.47
SW-620	32.74
CNS Cancer	
SF-268	37.74
SF-295	-16.28
SF-539	-4.90
SNB-19	20.43
SNB-75	51.25
U251	14.82
Melanoma	
LOX IMVI	1.86
MALME-3M	15.79
M14	31.49
MDA-MB-435	7.61
SK-MEL-2	26.40
SK-MEL-28	20.22
SK-MEL-5	-50.66
UACC-257	47.34
UACC-62	24.30
Ovarian Cancer	
IGROV1	26.38
OVCAR-3	17.38
OVCAR-4	42.65
OVCAR-5	33.25
OVCAR-8	9.65
NCI/ADR-RES	-25.61
SK-OV-3	45.34
Renal Cancer	
786-0	27.97
A498	78.68
ACHN	8.41
CAKI-1	27.97
RXF 393	-5.36
SN12C	2.20
TK-10	43.23
UO-31	23.81
Prostate Cancer	
PC-3	18.72
DU-145	18.84
Breast Cancer	
MCF7	4.85
MDA-MB-231/ATCC	30.87
HS 578T	16.85
BT-549	9.37
T-47D	42.38
MDA-MB-468	1.84
Mean	17.69
Delta	68.35
Range	129.34

Mean Growth Percent - Growth Percent

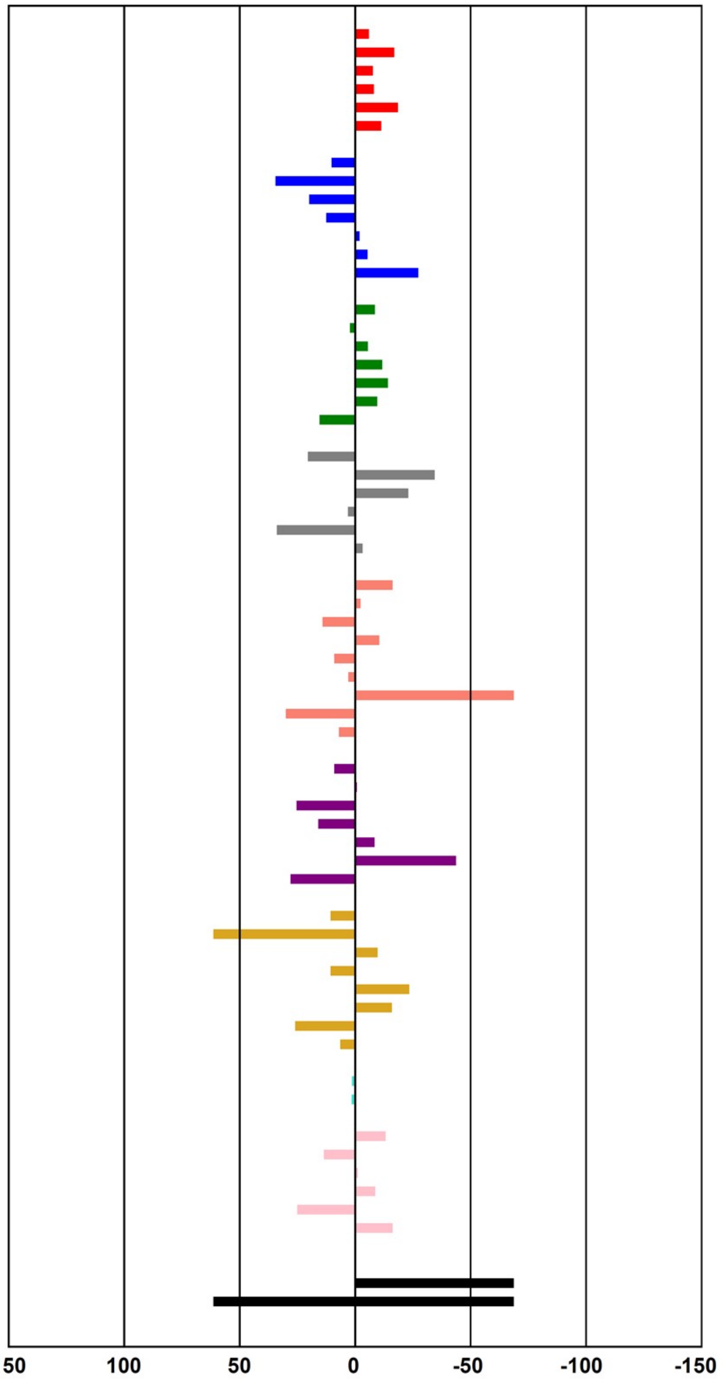

Compound **3j**

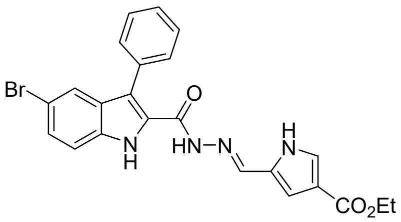

Developmental Therapeutics Program

One Dose Mean Graph

**NSC:** D-845426 / 1	**Conc:** 1.00E-5 Molar	**Test Date:** Jun 05, 2023
**Experiment ID:** 2306OS32		**Report Date:** Jul 16, 2023

Panel/Cell Line	Growth Percent
Leukemia	
CCRF-CEM	14.34
HL-60(TB)	-18.05
K-562	2.46
MOLT-4	10.77
RPMI-8226	3.75
SR	0.00
Non-Small Cell Lung Cancer	
A549/ATCC	31.68
EKVX	22.52
HOP-62	39.83
HOP-92	23.30
NCI-H226	8.04
NCI-H23	15.75
NCI-H322M	65.68
NCI-H460	9.80
NCI-H522	-18.44
Colon Cancer	
COLO 205	8.69
HCC-2998	3.43
HCT-116	20.00
HCT-15	5.94
HT29	0.76
KM12	17.02
SW-620	31.35
CNS Cancer	
SF-268	35.67
SF-295	14.82
SF-539	-89.78
SNB-19	15.77
SNB-75	36.83
U251	18.49
Melanoma	
LOX IMVI	17.59
MALME-3M	81.08
M14	18.10
MDA-MB-435	5.98
SK-MEL-2	33.06
SK-MEL-28	14.90
SK-MEL-5	-17.02
UACC-257	50.54
UACC-62	28.21
Ovarian Cancer	
IGROV1	37.48
OVCAR-3	12.99
OVCAR-4	47.48
OVCAR-5	12.82
OVCAR-8	10.51
NCI/ADR-RES	-36.41
SK-OV-3	-5.07
Renal Cancer	
786-0	27.86
A498	82.53
ACHN	22.71
CAKI-1	40.12
RXF 393	-2.39
SN12C	18.92
TK-10	-37.39
UO-31	31.20
Prostate Cancer	
PC-3	17.22
DU-145	20.23
Breast Cancer	
MCF7	9.28
MDA-MB-231/ATCC	3.32
HS 578T	35.89
T-47D	31.06
MDA-MB-468	20.76
Mean	16.34
Delta	106.12
Range	172.31

Mean Growth Percent - Growth Percent

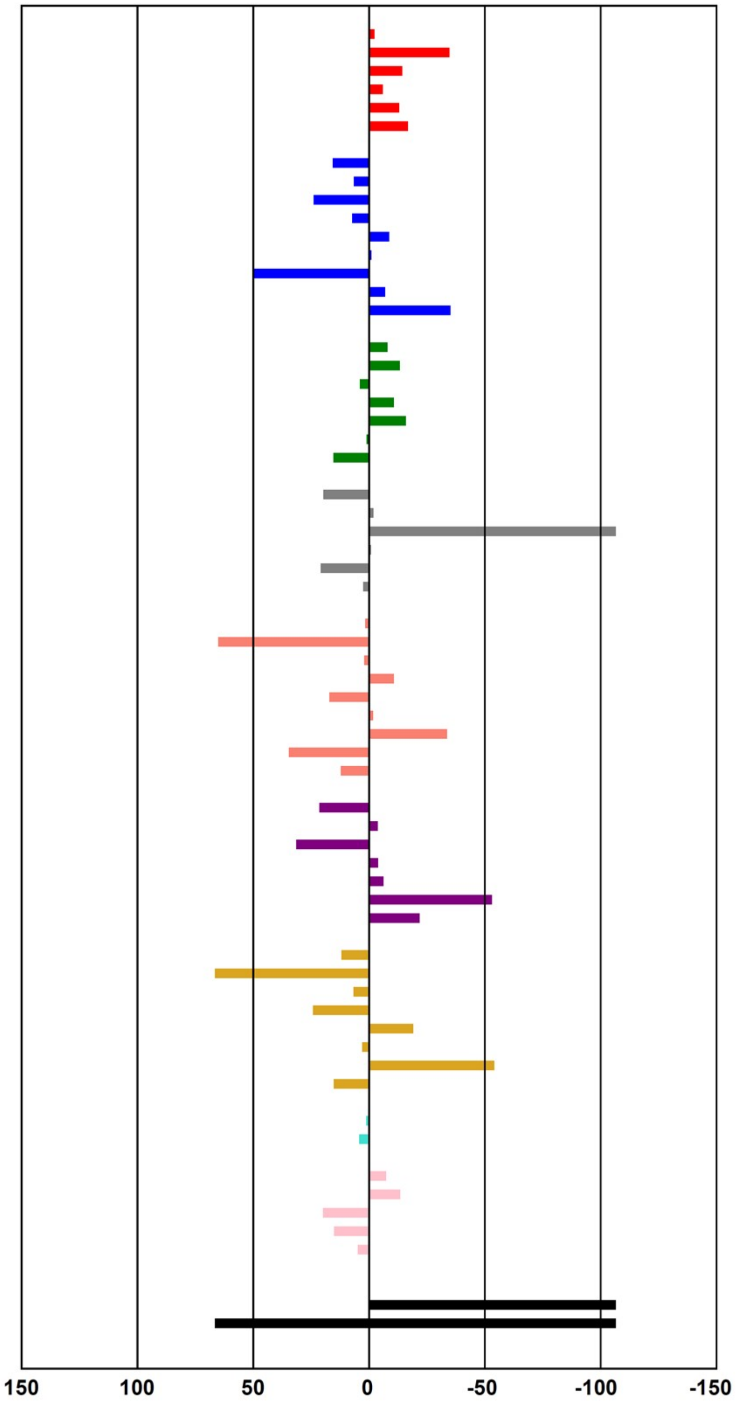

Compound **3k**

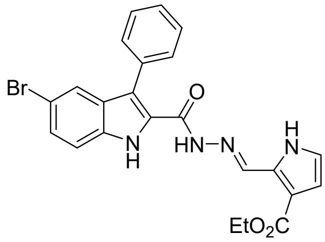

Developmental Therapeutics Program | **NSC:** D-845428 / 1 | **Conc:** 1.00E-5 Molar | **Test Date:** Jun 05, 2023

One Dose Mean Graph | **Experiment ID:** 2306OS32 | **Report Date:** Jul 16, 2023

Panel/Cell Line	Growth Percent
Leukemia	
CCRF-CEM	35.74
HL-60(TB)	30.70
K-562	22.92
MOLT-4	20.11
RPMI-8226	41.26
SR	18.78
Non-Small Cell Lung Cancer	
A549/ATCC	44.15
EKVX	38.66
HOP-62	50.35
HOP-92	81.49
NCI-H226	45.24
NCI-H23	50.30
NCI-H322M	67.50
NCI-H460	31.81
NCI-H522	58.29
Colon Cancer	
COLO 205	38.29
HCC-2998	56.00
HCT-116	48.47
HCT-15	18.34
HT29	43.07
KM12	41.30
SW-620	40.43
CNS Cancer	
SF-268	56.41
SF-295	27.03
SF-539	48.99
SNB-19	50.85
SNB-75	65.01
U251	46.68
Melanoma	
LOX IMVI	41.73
MALME-3M	94.35
M14	59.09
MDA-MB-435	56.66
SK-MEL-2	67.01
SK-MEL-28	72.55
SK-MEL-5	4.98
UACC-257	88.49
UACC-62	43.74
Ovarian Cancer	
IGROV1	47.61
OVCAR-3	65.59
OVCAR-4	51.42
OVCAR-5	80.59
OVCAR-8	51.42
NCI/ADR-RES	41.98
SK-OV-3	68.69
Renal Cancer	
786-0	77.02
A498	82.61
ACHN	39.12
CAKI-1	59.78
RXF 393	65.46
SN12C	41.60
TK-10	71.10
UO-31	40.81
Prostate Cancer	
PC-3	36.36
DU-145	63.03
Breast Cancer	
MCF7	21.99
MDA-MB-231/ATCC	55.53
HS 578T	67.17
T-47D	15.39
MDA-MB-468	20.62
Mean	49.35
Delta	44.37
Range	89.37

Mean Growth Percent - Growth Percent

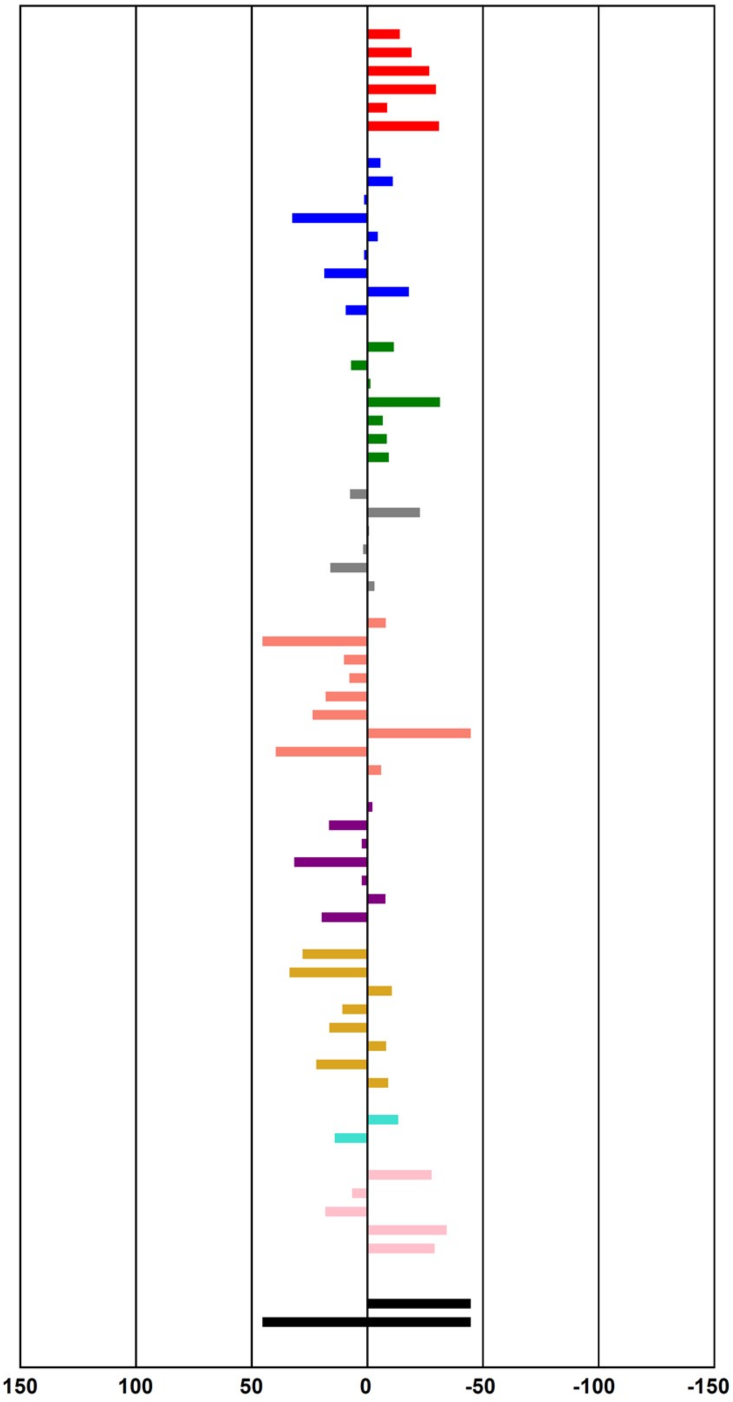

Compound **3l**

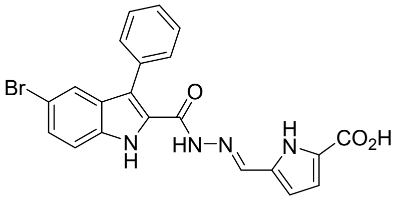

Developmental Therapeutics Program One Dose Mean Graph	NSC: D-843109 / 1	Conc: 1.00E-5 Molar	Test Date: Mar 20, 2023
	Experiment ID: 2303OS10		Report Date: Apr 24, 2023

Panel/Cell Line	Growth Percent
Leukemia	
CCRF-CEM	65.26
HL-60(TB)	77.06
K-562	63.91
MOLT-4	86.77
RPMI-8226	98.69
SR	56.88
Non-Small Cell Lung Cancer	
A549/ATCC	105.70
HOP-62	96.12
HOP-92	98.28
NCI-H226	92.19
NCI-H23	98.58
NCI-H460	112.05
NCI-H522	71.65
Colon Cancer	
COLO 205	100.70
HCC-2998	92.17
HCT-116	95.74
HCT-15	64.64
HT29	105.27
KM12	90.83
SW-620	96.58
CNS Cancer	
SF-268	89.63
SF-295	68.09
SF-539	85.94
SNB-19	77.42
SNB-75	109.39
U251	83.13
Melanoma	
LOX IMVI	66.90
MALME-3M	89.03
M14	90.30
MDA-MB-435	93.37
SK-MEL-2	95.20
SK-MEL-28	86.18
SK-MEL-5	100.58
UACC-257	102.99
UACC-62	68.48
Ovarian Cancer	
IGROV1	105.05
OVCAR-3	85.50
OVCAR-4	79.53
OVCAR-5	105.46
OVCAR-8	94.45
NCI/ADR-RES	75.24
SK-OV-3	103.32
Renal Cancer	
786-0	94.89
A498	117.60
ACHN	94.79
CAKI-1	88.56
RXF 393	97.22
SN12C	86.19
TK-10	115.91
UO-31	74.87
Prostate Cancer	
PC-3	88.33
DU-145	110.32
Breast Cancer	
MCF7	77.02
MDA-MB-231/ATCC	81.60
HS 578T	87.73
BT-549	97.57
T-47D	89.90
MDA-MB-468	65.59
Mean	89.52
Delta	32.64
Range	60.72

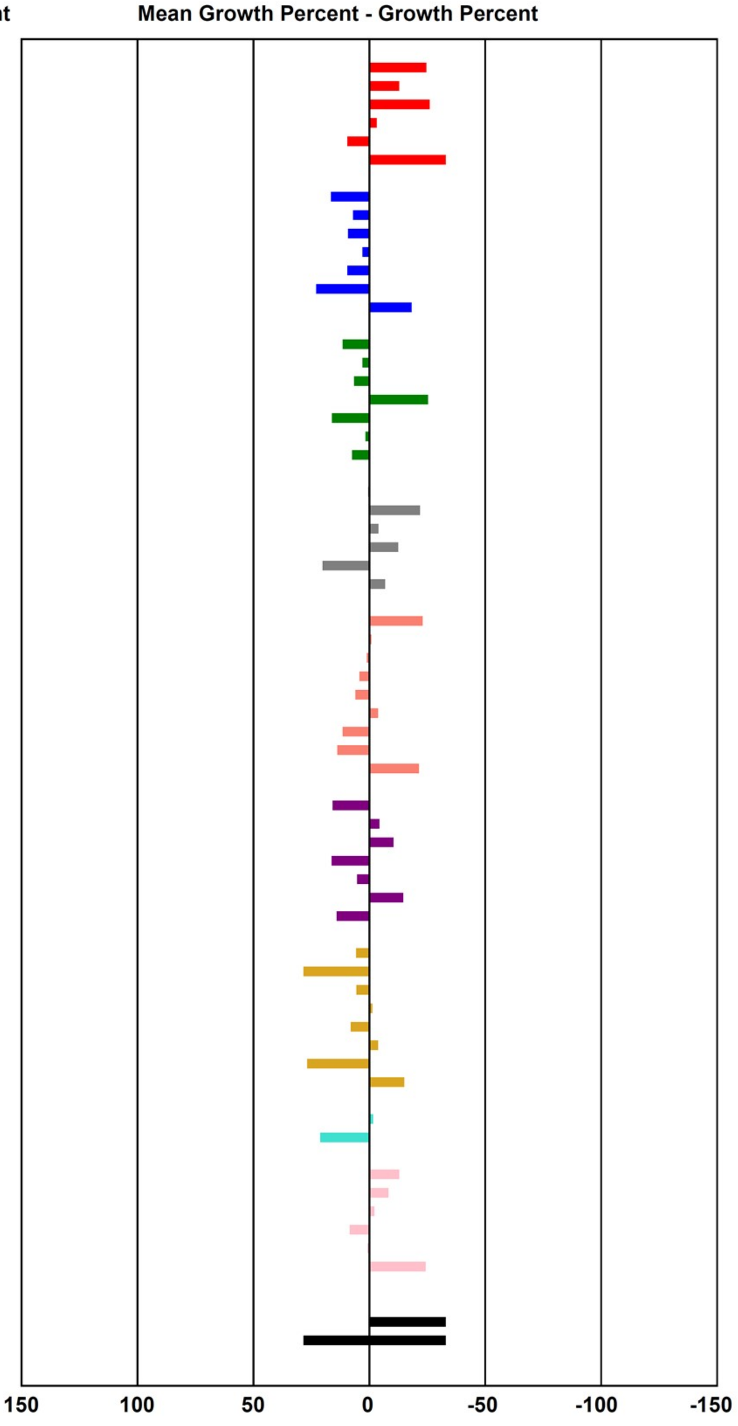

Compound **3m**

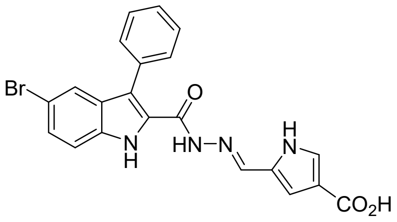

Developmental Therapeutics Program
One Dose Mean Graph

NSC: D-845429 / 1	Conc: 1.00E-5 Molar	Test Date: Jun 05, 2023
Experiment ID: 2306OS32		Report Date: Jul 16, 2023

Panel/Cell Line	Growth Percent
Leukemia	
CCRF-CEM	45.01
HL-60(TB)	70.81
K-562	46.23
MOLT-4	64.89
RPMI-8226	100.87
SR	40.10
Non-Small Cell Lung Cancer	
A549/ATCC	103.62
EKVX	88.86
HOP-62	72.38
HOP-92	48.67
NCI-H226	80.85
NCI-H23	95.42
NCI-H322M	99.62
NCI-H460	96.15
NCI-H522	68.53
Colon Cancer	
COLO 205	76.38
HCC-2998	69.97
HCT-116	88.94
HCT-15	59.68
HT29	105.91
KM12	93.20
SW-620	94.56
CNS Cancer	
SF-268	62.16
SF-295	85.35
SF-539	49.95
SNB-19	66.30
SNB-75	74.44
U251	66.58
Melanoma	
LOX IMVI	68.39
MALME-3M	93.25
M14	74.56
MDA-MB-435	89.67
SK-MEL-2	67.47
SK-MEL-28	51.55
SK-MEL-5	74.79
UACC-257	99.32
UACC-62	54.66
Ovarian Cancer	
IGROV1	94.07
OVCAR-3	57.74
OVCAR-4	68.30
OVCAR-5	99.67
OVCAR-8	77.85
NCI/ADR-RES	72.96
SK-OV-3	94.71
Renal Cancer	
786-0	85.95
A498	96.04
ACHN	84.60
CAKI-1	83.97
RXF 393	75.36
SN12C	68.71
TK-10	95.32
UO-31	91.58
Prostate Cancer	
PC-3	62.12
DU-145	97.05
Breast Cancer	
MCF7	62.36
MDA-MB-231/ATCC	68.34
HS 578T	73.38
T-47D	51.39
MDA-MB-468	29.20
Mean	75.93
Delta	46.73
Range	76.71

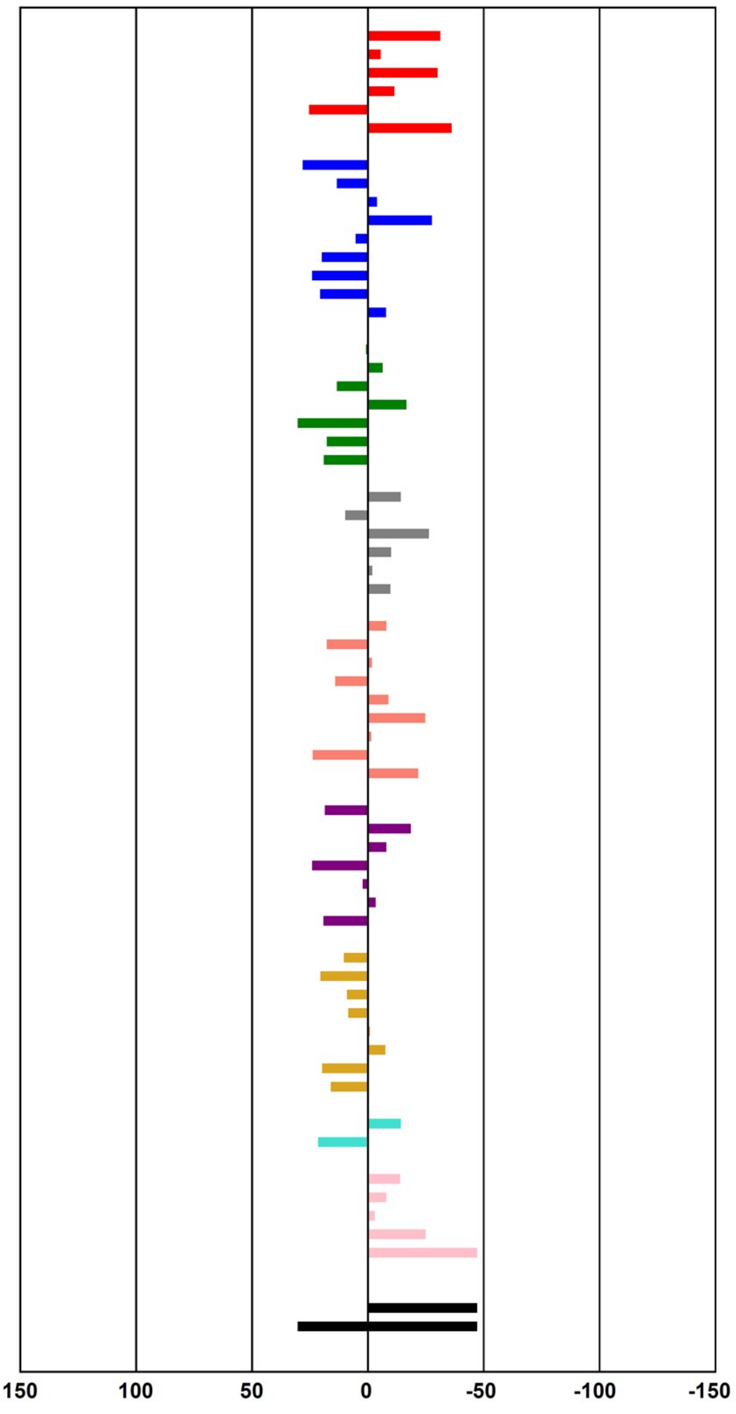

Compound **3n**

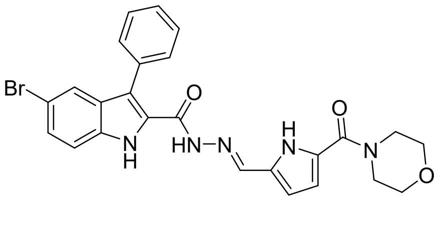

Developmental Therapeutics Program One Dose Mean Graph	NSC: D-845425 / 1	Conc: 1.00E-5 Molar	Test Date: Jun 05, 2023
	Experiment ID: 2306OS32		Report Date: Jul 16, 2023

Panel/Cell Line	Growth Percent
Leukemia	
CCRF-CEM	10.59
HL-60(TB)	-9.97
K-562	5.19
MOLT-4	9.68
RPMI-8226	11.01
SR	1.99
Non-Small Cell Lung Cancer	
A549/ATCC	28.73
EKVX	23.41
HOP-62	39.03
HOP-92	-7.11
NCI-H226	3.88
NCI-H23	14.29
NCI-H322M	62.78
NCI-H460	10.38
NCI-H522	-16.86
Colon Cancer	
COLO 205	15.83
HCC-2998	6.98
HCT-116	15.26
HCT-15	7.67
HT29	2.40
KM12	18.47
SW-620	34.87
CNS Cancer	
SF-268	32.00
SF-295	7.15
SF-539	2.13
SNB-19	24.08
SNB-75	18.28
U251	18.91
Melanoma	
LOX IMVI	16.03
MALME-3M	76.05
M14	34.48
MDA-MB-435	2.05
SK-MEL-2	15.34
SK-MEL-28	47.01
SK-MEL-5	-13.91
UACC-257	73.53
UACC-62	38.57
Ovarian Cancer	
IGROV1	44.37
OVCAR-3	11.90
OVCAR-4	40.51
OVCAR-5	42.54
OVCAR-8	8.82
NCI/ADR-RES	-36.23
SK-OV-3	60.17
Renal Cancer	
786-0	14.37
A498	73.29
ACHN	38.05
CAKI-1	39.01
RXF 393	-26.01
SN12C	24.27
TK-10	51.99
UO-31	40.64
Prostate Cancer	
PC-3	9.87
DU-145	18.53
Breast Cancer	
MCF7	8.75
MDA-MB-231/ATCC	17.48
HS 578T	29.68
T-47D	32.02
MDA-MB-468	16.26
Mean	21.03
Delta	57.26
Range	112.28

Mean Growth Percent - Growth Percent

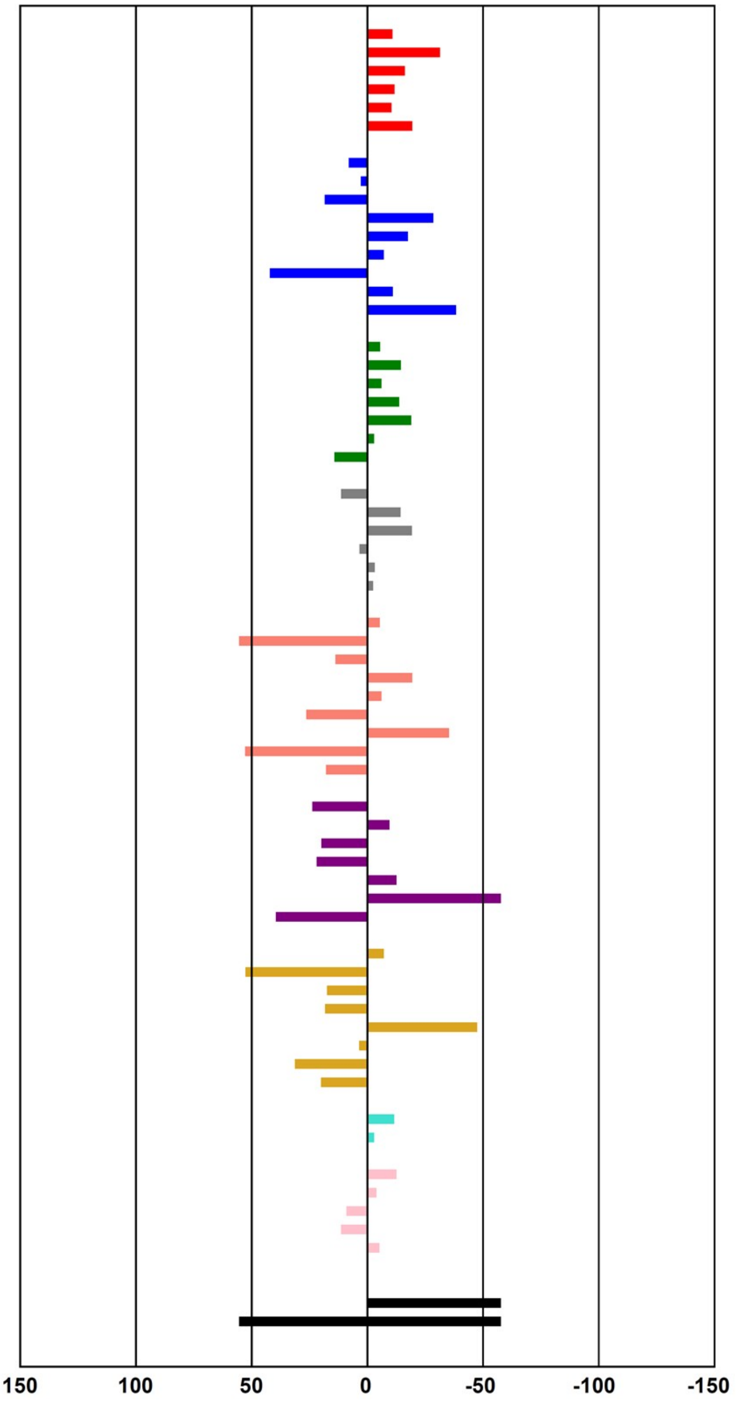

Compound **3o**

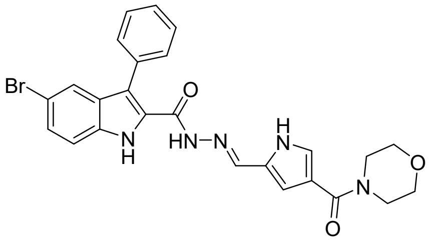

Developmental Therapeutics Program One Dose Mean Graph	NSC: D-845431 / 1	Conc: 1.00E-5 Molar	Test Date: Jun 12, 2023
	Experiment ID: 2306OS36		Report Date: Jul 18, 2023

Panel/Cell Line	Growth Percent
Leukemia	
CCRF-CEM	15.34
HL-60(TB)	14.03
K-562	20.31
MOLT-4	15.43
RPMI-8226	22.02
SR	15.48
Non-Small Cell Lung Cancer	
A549/ATCC	58.15
EKVX	31.41
HOP-62	54.25
HOP-92	41.39
NCI-H226	48.55
NCI-H23	51.14
NCI-H460	47.98
NCI-H522	46.10
Colon Cancer	
COLO 205	80.75
HCC-2998	55.51
HCT-116	36.11
HCT-15	27.31
HT29	70.58
KM12	51.39
SW-620	75.29
CNS Cancer	
SF-268	47.09
SF-295	35.07
SF-539	51.98
SNB-19	32.91
U251	21.75
Melanoma	
LOX IMVI	32.82
M14	42.03
MDA-MB-435	46.82
SK-MEL-2	69.46
SK-MEL-28	59.50
SK-MEL-5	1.89
UACC-257	78.91
UACC-62	26.78
Ovarian Cancer	
OVCAR-3	53.96
OVCAR-4	33.51
OVCAR-5	82.58
OVCAR-8	54.02
NCI/ADR-RES	53.40
SK-OV-3	81.63
Renal Cancer	
786-0	44.62
A498	61.25
ACHN	60.68
CAKI-1	52.84
RXF 393	18.90
SN12C	54.93
TK-10	79.67
Prostate Cancer	
PC-3	24.14
DU-145	62.59
Breast Cancer	
MCF7	25.73
MDA-MB-231/ATCC	51.21
HS 578T	47.34
BT-549	48.61
T-47D	56.16
MDA-MB-468	-9.08
Mean	44.80
Delta	53.88
Range	91.66

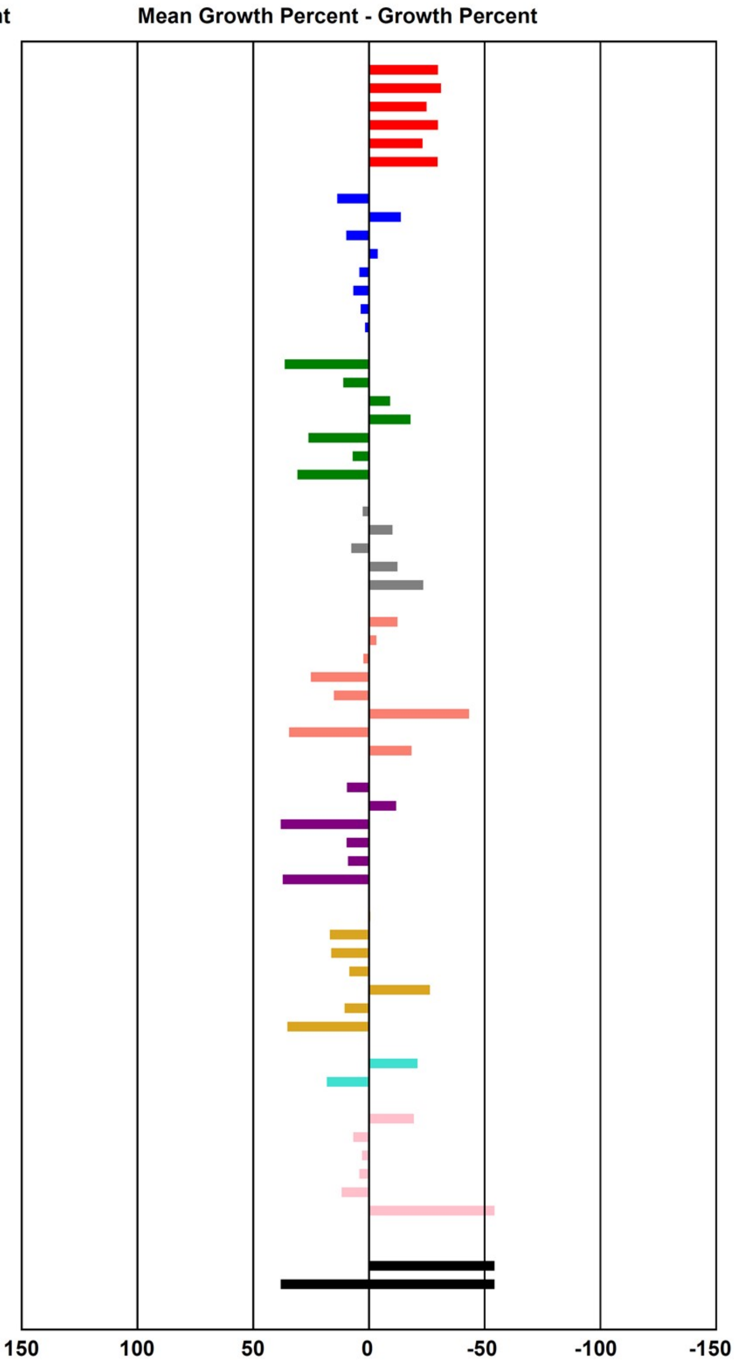

Compound **3p**

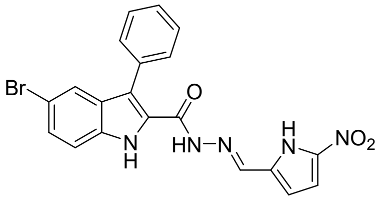

Developmental Therapeutics Program
One Dose Mean Graph

NSC: D-845422 / 1 | **Conc:** 1.00E-5 Molar | **Test Date:** Jun 05, 2023
Experiment ID: 2306OS32 | **Report Date:** Jul 16, 2023

Panel/Cell Line	Growth Percent
Leukemia	
CCRF-CEM	5.79
HL-60(TB)	-14.01
K-562	5.37
MOLT-4	6.03
RPMI-8226	-9.11
SR	2.98
Non-Small Cell Lung Cancer	
A549/ATCC	14.15
EKVX	13.16
HOP-62	14.91
HOP-92	-9.49
NCI-H226	-12.09
NCI-H23	2.69
NCI-H322M	53.42
NCI-H460	7.49
NCI-H522	-34.97
Colon Cancer	
COLO 205	-24.27
HCC-2998	2.96
HCT-116	12.42
HCT-15	3.20
HT29	2.64
KM12	4.14
SW-620	27.86
CNS Cancer	
SF-268	22.80
SF-295	-4.92
SF-539	3.73
SNB-19	13.59
SNB-75	6.35
U251	6.46
Melanoma	
LOX IMVI	6.80
MALME-3M	57.75
M14	23.83
MDA-MB-435	18.28
SK-MEL-2	-1.31
SK-MEL-28	42.52
SK-MEL-5	-48.69
UACC-257	22.98
UACC-62	27.08
Ovarian Cancer	
IGROV1	26.87
OVCAR-3	17.57
OVCAR-4	30.92
OVCAR-5	56.19
OVCAR-8	-13.19
NCI/ADR-RES	-36.29
SK-OV-3	32.84
Renal Cancer	
786-0	21.49
A498	68.79
ACHN	12.89
CAKI-1	18.89
RXF 393	11.51
SN12C	25.21
TK-10	-0.57
UO-31	20.38
Prostate Cancer	
PC-3	11.46
DU-145	13.65
Breast Cancer	
MCF7	3.71
MDA-MB-231/ATCC	4.22
HS 578T	37.34
T-47D	30.67
MDA-MB-468	-17.71
Mean	11.01
Delta	59.70
Range	117.48

Mean Growth Percent - Growth Percent

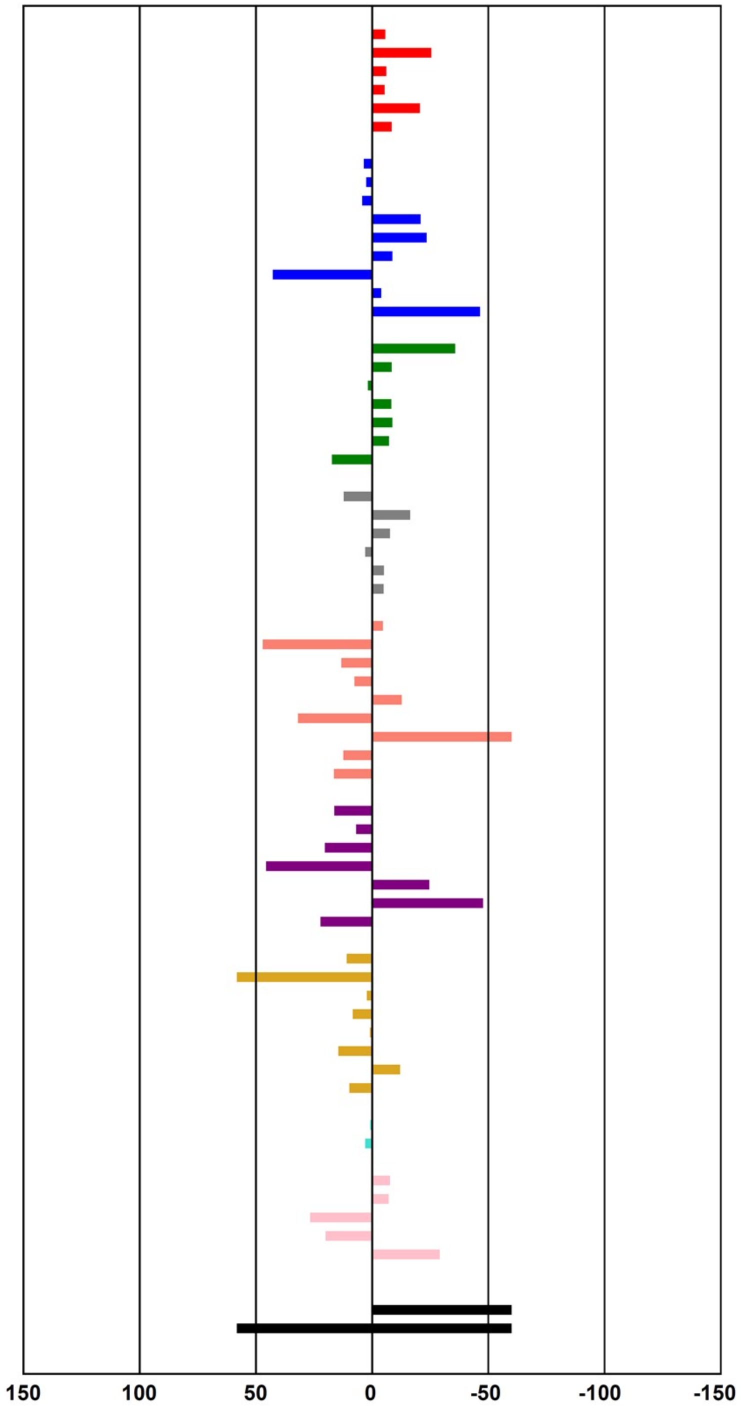

Compound **3q**

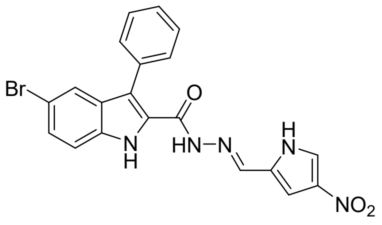

Developmental Therapeutics Program One Dose Mean Graph	NSC: D-845423 / 1	Conc: 1.00E-5 Molar	Test Date: Jun 05, 2023
	Experiment ID: 2306OS32		Report Date: Jul 16, 2023

Panel/Cell Line	Growth Percent
Leukemia	
CCRF-CEM	13.40
HL-60(TB)	-1.13
K-562	8.78
MOLT-4	23.30
RPMI-8226	21.26
SR	11.20
Non-Small Cell Lung Cancer	
A549/ATCC	41.49
EKVX	40.87
HOP-62	35.58
HOP-92	53.58
NCI-H226	35.65
NCI-H23	30.03
NCI-H322M	60.55
NCI-H460	12.68
NCI-H522	0.81
Colon Cancer	
COLO 205	9.57
HCC-2998	53.64
HCT-116	25.79
HCT-15	10.26
HT29	4.38
KM12	17.04
SW-620	24.39
CNS Cancer	
SF-268	30.58
SF-295	17.29
SF-539	10.67
SNB-19	14.33
SNB-75	-1.73
U251	14.65
Melanoma	
LOX IMVI	16.08
MALME-3M	42.47
M14	20.22
MDA-MB-435	-39.71
SK-MEL-2	44.01
SK-MEL-28	23.38
SK-MEL-5	-0.38
UACC-257	37.21
UACC-62	23.67
Ovarian Cancer	
IGROV1	52.77
OVCAR-3	11.55
OVCAR-4	49.80
OVCAR-5	38.48
OVCAR-8	35.69
NCI/ADR-RES	-2.06
SK-OV-3	47.80
Renal Cancer	
786-0	32.86
A498	76.90
ACHN	27.25
CAKI-1	42.65
RXF 393	28.25
SN12C	45.56
TK-10	114.02
UO-31	44.13
Prostate Cancer	
PC-3	33.42
DU-145	24.89
Breast Cancer	
MCF7	8.05
MDA-MB-231/ATCC	41.77
HS 578T	33.28
T-47D	41.87
MDA-MB-468	-9.85
Mean	27.20
Delta	66.91
Range	153.73

Mean Growth Percent - Growth Percent

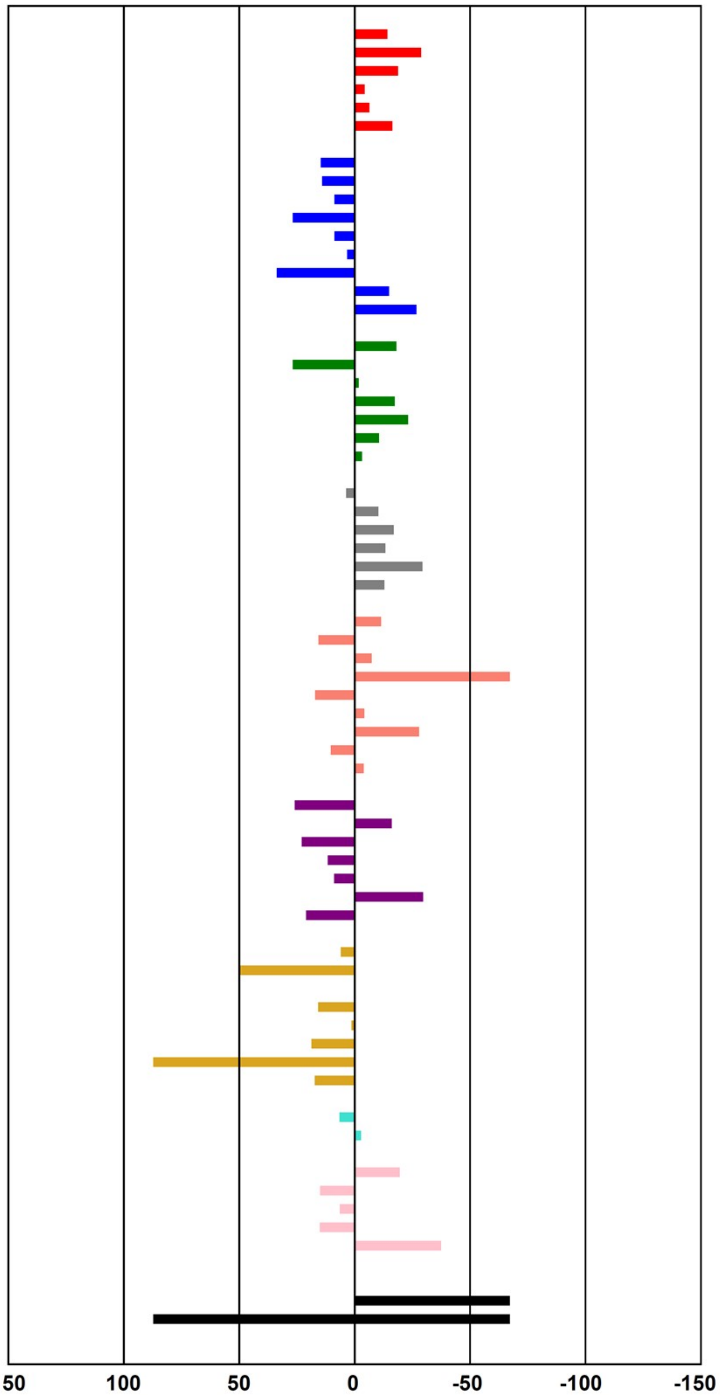

Compound **3r**

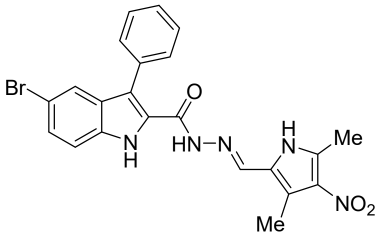

Developmental Therapeutics Program | **NSC:** D-845430 / 1 | **Conc:** 1.00E-5 Molar | **Test Date:** Jun 05, 2023

One Dose Mean Graph | **Experiment ID:** 2306OS32 | **Report Date:** Jul 16, 2023

Panel/Cell Line	Growth Percent
**Leukemia**	
CCRF-CEM	1.38
HL-60(TB)	-17.20
K-562	-9.46
MOLT-4	2.47
RPMI-8226	2.69
SR	-20.99
**Non-Small Cell Lung Cancer**	
A549/ATCC	3.34
EKVX	5.20
HOP-62	-73.99
HOP-92	-57.45
NCI-H226	-1.11
NCI-H23	-26.53
NCI-H322M	7.66
NCI-H460	-0.29
NCI-H522	-28.80
**Colon Cancer**	
COLO 205	-74.33
HCC-2998	-26.36
HCT-116	-52.23
HCT-15	-44.01
HT29	-70.82
KM12	-31.11
SW-620	17.17
**CNS Cancer**	
SF-268	17.00
SF-295	-18.02
SF-539	13.38
SNB-19	11.42
SNB-75	8.15
U251	-41.90
**Melanoma**	
LOX IMVI	-90.72
MALME-3M	18.34
M14	21.35
MDA-MB-435	4.89
SK-MEL-2	-7.88
SK-MEL-28	13.31
SK-MEL-5	-71.50
UACC-257	13.01
UACC-62	-0.58
**Ovarian Cancer**	
IGROV1	13.68
OVCAR-3	-32.48
OVCAR-4	13.38
OVCAR-5	-16.81
OVCAR-8	8.04
NCI/ADR-RES	-40.06
SK-OV-3	0.32
**Renal Cancer**	
786-0	1.27
A498	41.75
ACHN	4.55
CAKI-1	19.75
RXF 393	-19.26
SN12C	-48.81
TK-10	35.35
UO-31	17.01
**Prostate Cancer**	
PC-3	-28.42
DU-145	14.43
**Breast Cancer**	
MCF7	-7.20
MDA-MB-231/ATCC	10.69
HS 578T	42.82
T-47D	-36.67
MDA-MB-468	-17.47
Mean	-10.66
Delta	80.06
Range	133.54

Mean Growth Percent - Growth Percent

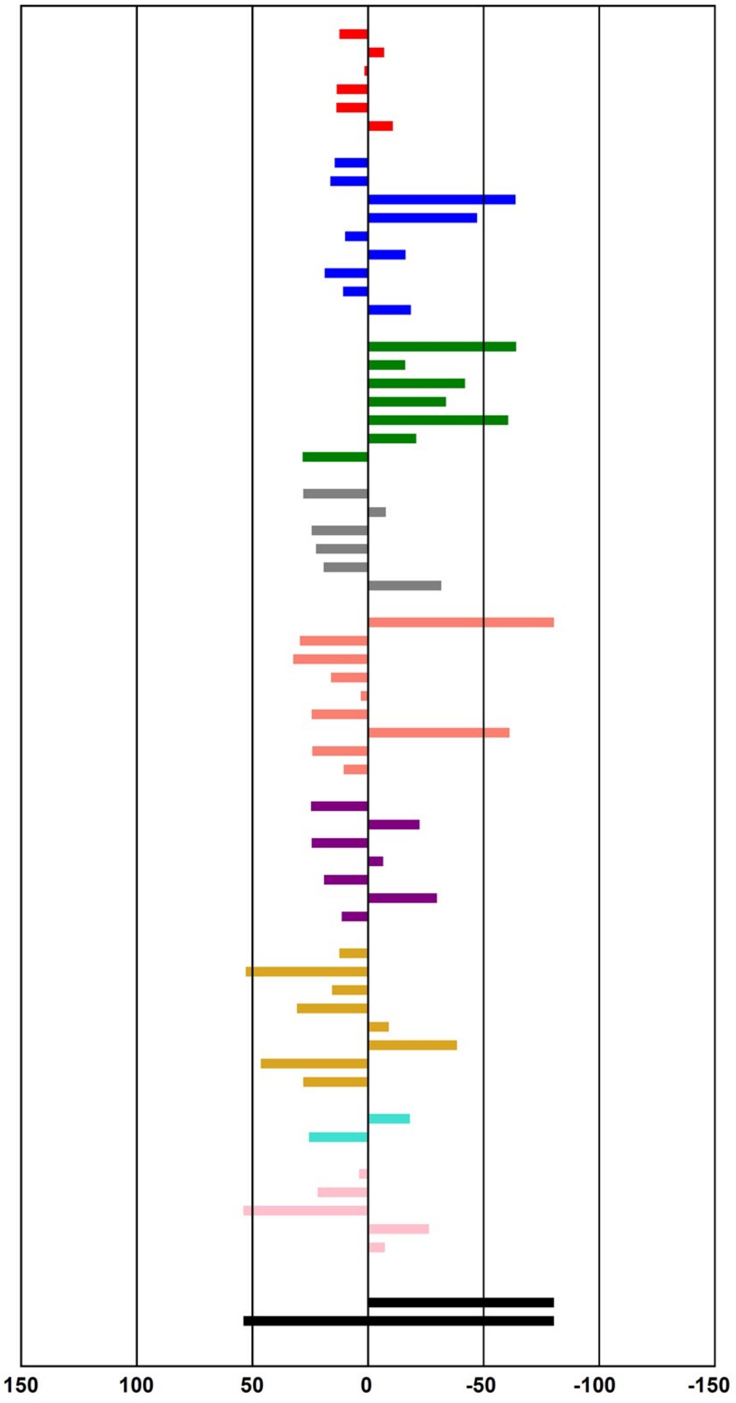

Compound **3s**

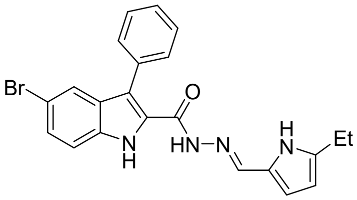

Developmental Therapeutics Program | **NSC:** D-847584 / 1 | **Conc:** 1.00E-5 Molar | **Test Date:** Aug 14, 2023

One Dose Mean Graph | **Experiment ID:** 2308OS60 | **Report Date:** Sep 07, 2023

Panel/Cell Line	Growth Percent
Leukemia	
CCRF-CEM	2.11
HL-60(TB)	-26.99
K-562	-2.16
MOLT-4	0.97
RPMI-8226	6.45
SR	-12.97
Non-Small Cell Lung Cancer	
A549/ATCC	11.38
EKVX	23.88
HOP-62	32.28
HOP-92	16.42
NCI-H226	19.00
NCI-H23	18.05
NCI-H322M	32.43
NCI-H460	4.52
NCI-H522	-1.18
Colon Cancer	
COLO 205	1.69
HCC-2998	3.82
HCT-116	5.44
HCT-15	7.89
HT29	3.05
KM12	-1.80
SW-620	23.21
CNS Cancer	
SF-268	27.43
SF-295	8.27
SF-539	8.14
SNB-19	22.60
SNB-75	46.64
U251	8.57
Melanoma	
LOX IMVI	8.89
MALME-3M	43.70
M14	25.58
MDA-MB-435	13.48
SK-MEL-2	44.02
SK-MEL-28	58.75
SK-MEL-5	-41.85
UACC-257	40.32
UACC-62	20.03
Ovarian Cancer	
IGROV1	11.92
OVCAR-3	17.41
OVCAR-4	38.20
OVCAR-5	24.07
OVCAR-8	8.21
NCI/ADR-RES	-15.48
SK-OV-3	52.34
Renal Cancer	
786-0	12.52
A498	53.64
ACHN	10.65
CAKI-1	3.59
SN12C	6.52
TK-10	37.86
UO-31	13.75
Prostate Cancer	
PC-3	17.42
DU-145	10.84
Breast Cancer	
MCF7	13.58
MDA-MB-231/ATCC	3.36
HS 578T	9.86
BT-549	11.88
T-47D	42.41
MDA-MB-468	-12.09
Mean	14.82
Delta	56.67
Range	100.60

Mean Growth Percent - Growth Percent

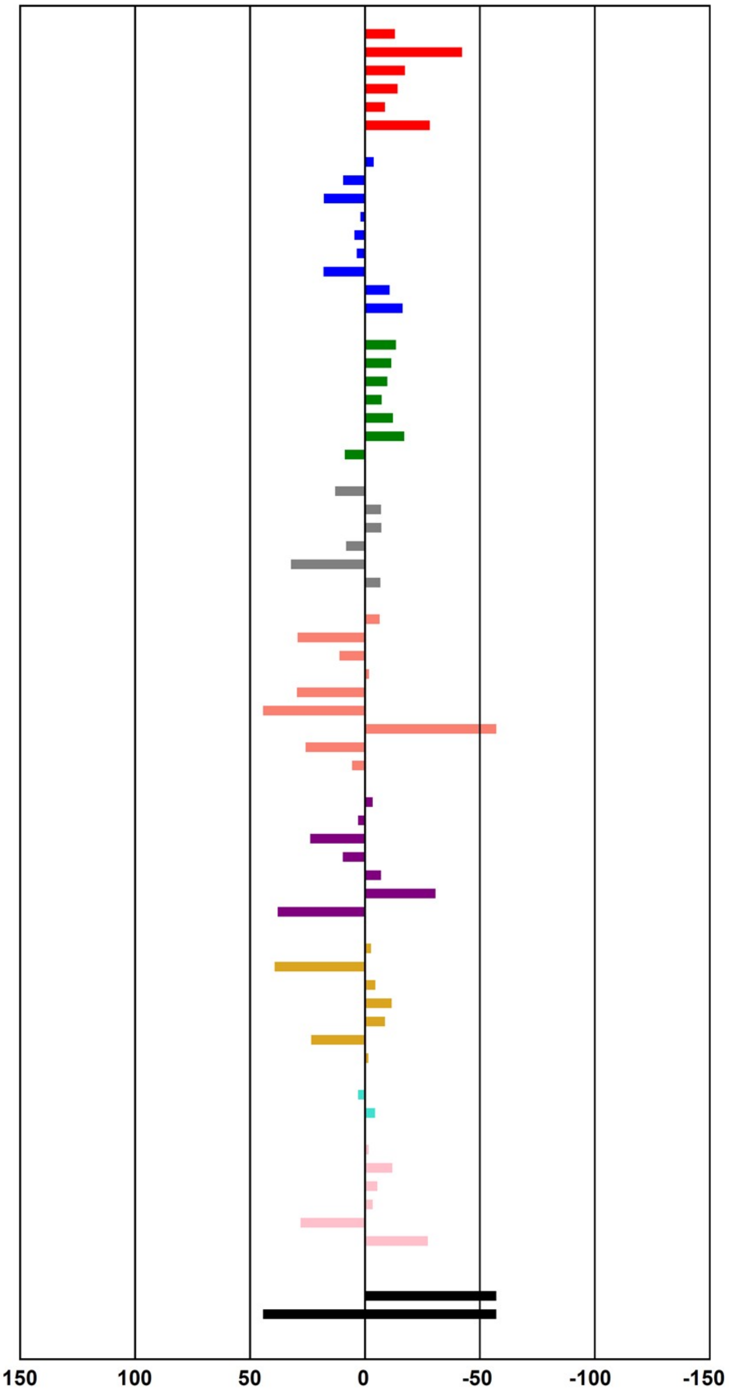

Compound **3t**

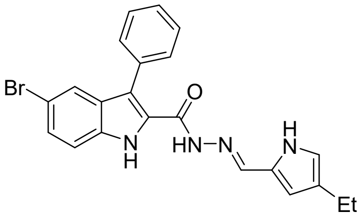

Developmental Therapeutics Program One Dose Mean Graph	NSC: D-847585 / 1	Conc: 1.00E-5 Molar	Test Date: Aug 14, 2023
	Experiment ID: 2308OS60		Report Date: Sep 07, 2023

Panel/Cell Line	Growth Percent
**Leukemia**	
CCRF-CEM	-8.75
HL-60(TB)	-32.19
K-562	-12.00
MOLT-4	-6.19
RPMI-8226	1.01
SR	-25.07
**Non-Small Cell Lung Cancer**	
A549/ATCC	4.32
EKVX	14.39
HOP-62	-4.14
HOP-92	-24.69
NCI-H226	-15.57
NCI-H23	2.86
NCI-H322M	27.66
NCI-H460	-15.70
NCI-H522	-8.86
**Colon Cancer**	
COLO 205	-23.33
HCC-2998	-4.36
HCT-116	11.00
HCT-15	5.04
HT29	1.72
KM12	-13.54
SW-620	11.49
**CNS Cancer**	
SF-268	7.63
SF-295	3.37
SF-539	-26.11
SNB-19	11.28
SNB-75	16.25
U251	-17.58
**Melanoma**	
LOX IMVI	-56.08
MALME-3M	14.17
M14	11.05
MDA-MB-435	18.33
SK-MEL-2	34.44
SK-MEL-28	49.63
SK-MEL-5	-28.95
UACC-257	35.89
UACC-62	-4.08
**Ovarian Cancer**	
IGROV1	8.23
OVCAR-3	-22.98
OVCAR-4	32.03
OVCAR-5	21.62
OVCAR-8	-22.76
NCI/ADR-RES	-41.06
SK-OV-3	27.35
**Renal Cancer**	
786-0	2.46
A498	81.41
ACHN	-0.99
CAKI-1	-26.11
SN12C	-15.18
TK-10	41.82
UO-31	-6.65
**Prostate Cancer**	
PC-3	8.42
DU-145	6.84
**Breast Cancer**	
MCF7	6.86
MDA-MB-231/ATCC	-2.61
HS 578T	0.23
BT-549	-13.82
T-47D	20.47
MDA-MB-468	-41.40
Mean	.31
Delta	56.39
Range	137.49

Mean Growth Percent - Growth Percent

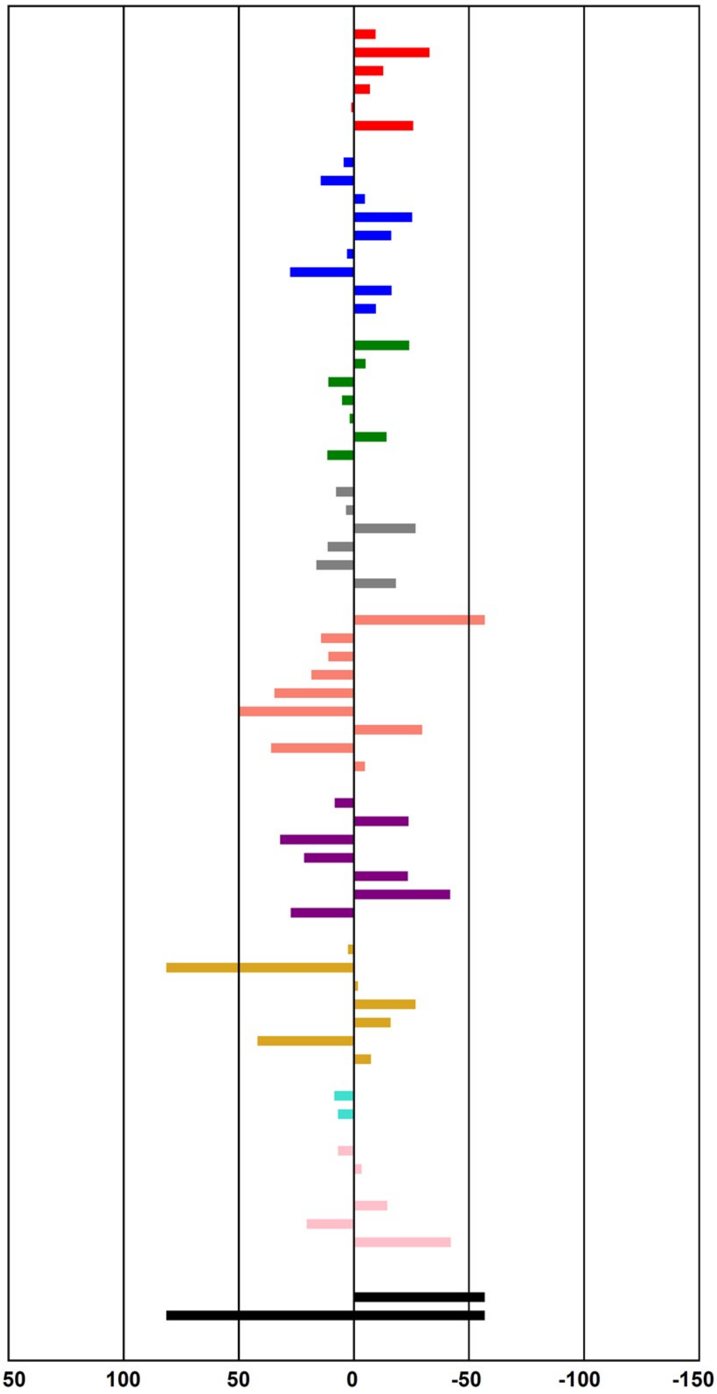

Compound **3u**

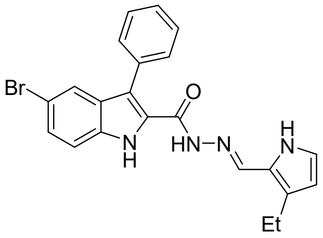

Developmental Therapeutics Program

One Dose Mean Graph

**NSC:** D-847586 / 1	**Conc:** 1.00E-5 Molar	**Test Date:** Aug 14, 2023
**Experiment ID:** 2308OS60		**Report Date:** Sep 07, 2023

Panel/Cell Line	Growth Percent
Leukemia	
CCRF-CEM	-10.62
HL-60(TB)	-20.36
K-562	0.57
MOLT-4	-5.68
RPMI-8226	22.55
SR	-28.22
Non-Small Cell Lung Cancer	
A549/ATCC	20.64
EKVX	23.32
HOP-62	22.71
HOP-92	60.75
NCI-H226	0.33
NCI-H23	19.37
NCI-H322M	43.79
NCI-H460	5.17
NCI-H522	16.20
Colon Cancer	
COLO 205	-12.83
HCC-2998	35.01
HCT-116	22.32
HCT-15	22.58
HT29	25.13
KM12	10.90
SW-620	30.95
CNS Cancer	
SF-268	20.97
SF-295	20.41
SF-539	6.05
SNB-19	22.82
SNB-75	48.31
U251	17.59
Melanoma	
LOX IMVI	2.13
MALME-3M	28.07
M14	6.36
MDA-MB-435	26.23
SK-MEL-2	53.38
SK-MEL-28	34.39
SK-MEL-5	8.98
UACC-257	37.75
UACC-62	6.99
Ovarian Cancer	
IGROV1	38.07
OVCAR-3	31.59
OVCAR-4	35.51
OVCAR-5	51.60
OVCAR-8	20.05
NCI/ADR-RES	-19.56
SK-OV-3	60.91
Renal Cancer	
786-0	22.18
A498	104.29
ACHN	14.70
CAKI-1	22.45
SN12C	-18.13
TK-10	63.77
UO-31	12.09
Prostate Cancer	
PC-3	21.91
DU-145	32.97
Breast Cancer	
MCF7	13.33
MDA-MB-231/ATCC	18.72
HS 578T	41.83
BT-549	39.73
T-47D	23.90
MDA-MB-468	15.34
Mean	21.90
Delta	50.12
Range	132.51

Mean Growth Percent - Growth Percent

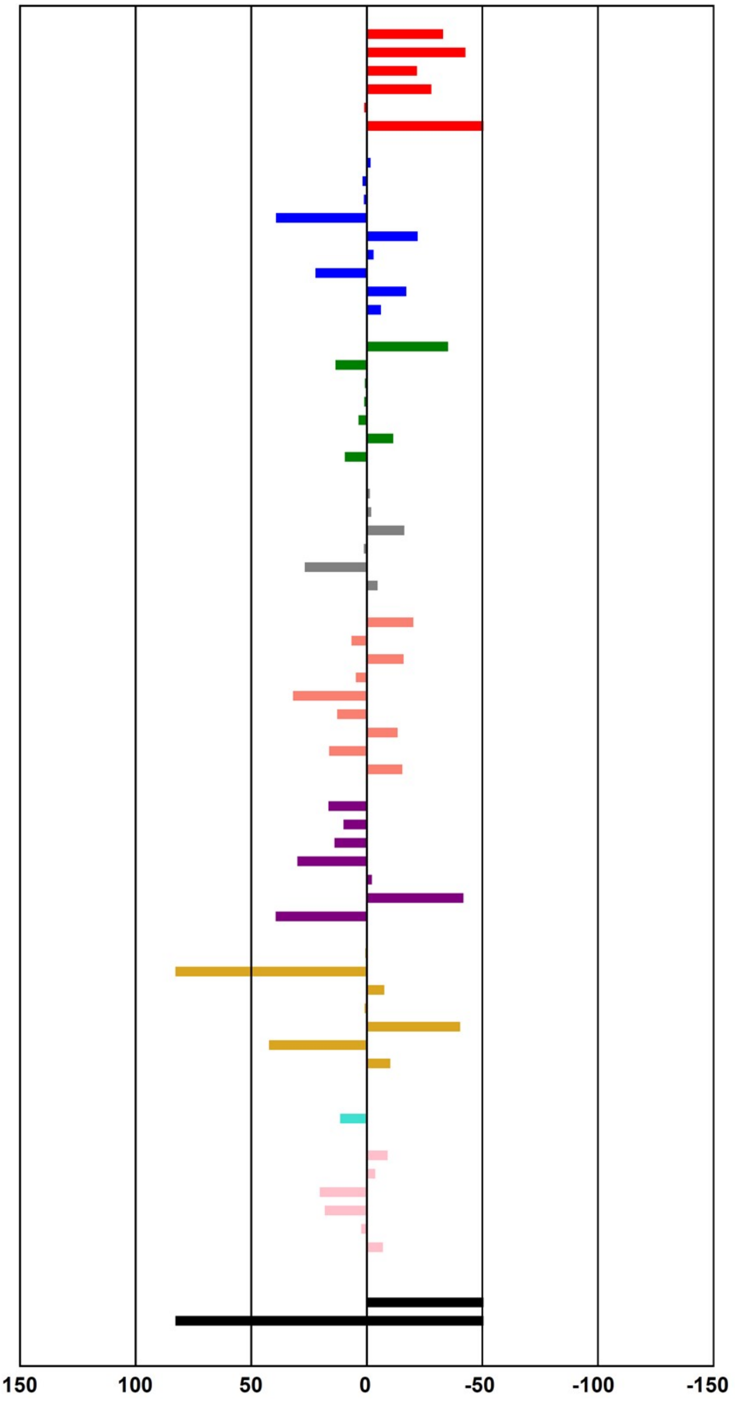

Compound **3v**

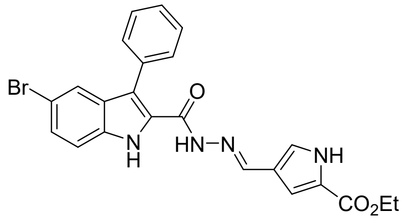

Developmental Therapeutics Program | **NSC:** D-843108 / 1 | **Conc:** 1.00E-5 Molar | **Test Date:** Mar 20, 2023

One Dose Mean Graph | **Experiment ID:** 2303OS10 | **Report Date:** Apr 24, 2023

Panel/Cell Line	Growth Percent
Leukemia	
CCRF-CEM	19.34
HL-60(TB)	6.00
K-562	16.50
MOLT-4	24.04
RPMI-8226	25.68
SR	19.25
Non-Small Cell Lung Cancer	
A549/ATCC	36.15
HOP-62	43.25
HOP-92	75.26
NCI-H226	53.37
NCI-H23	28.47
NCI-H460	27.07
NCI-H522	-11.94
Colon Cancer	
COLO 205	11.44
HCC-2998	45.46
HCT-116	26.82
HCT-15	16.78
HT29	11.31
KM12	16.71
SW-620	43.07
CNS Cancer	
SF-268	53.56
SF-295	5.68
SF-539	-14.87
SNB-19	45.04
SNB-75	32.26
U251	27.81
Melanoma	
LOX IMVI	33.89
MALME-3M	23.44
M14	33.84
MDA-MB-435	-15.44
SK-MEL-2	40.37
SK-MEL-28	55.18
SK-MEL-5	-3.99
UACC-257	70.07
UACC-62	42.18
Ovarian Cancer	
IGROV1	45.81
OVCAR-3	14.55
OVCAR-4	63.24
OVCAR-5	42.34
OVCAR-8	28.50
NCI/ADR-RES	-9.07
SK-OV-3	60.51
Renal Cancer	
786-0	52.59
A498	93.14
ACHN	39.35
CAKI-1	41.18
RXF 393	-14.90
SN12C	30.24
TK-10	52.50
UO-31	36.77
Prostate Cancer	
PC-3	32.04
DU-145	16.59
Breast Cancer	
MCF7	14.67
MDA-MB-231/ATCC	33.45
HS 578T	11.33
BT-549	45.92
T-47D	40.40
MDA-MB-468	-8.29
Mean	29.76
Delta	45.20
Range	108.58

Mean Growth Percent - Growth Percent

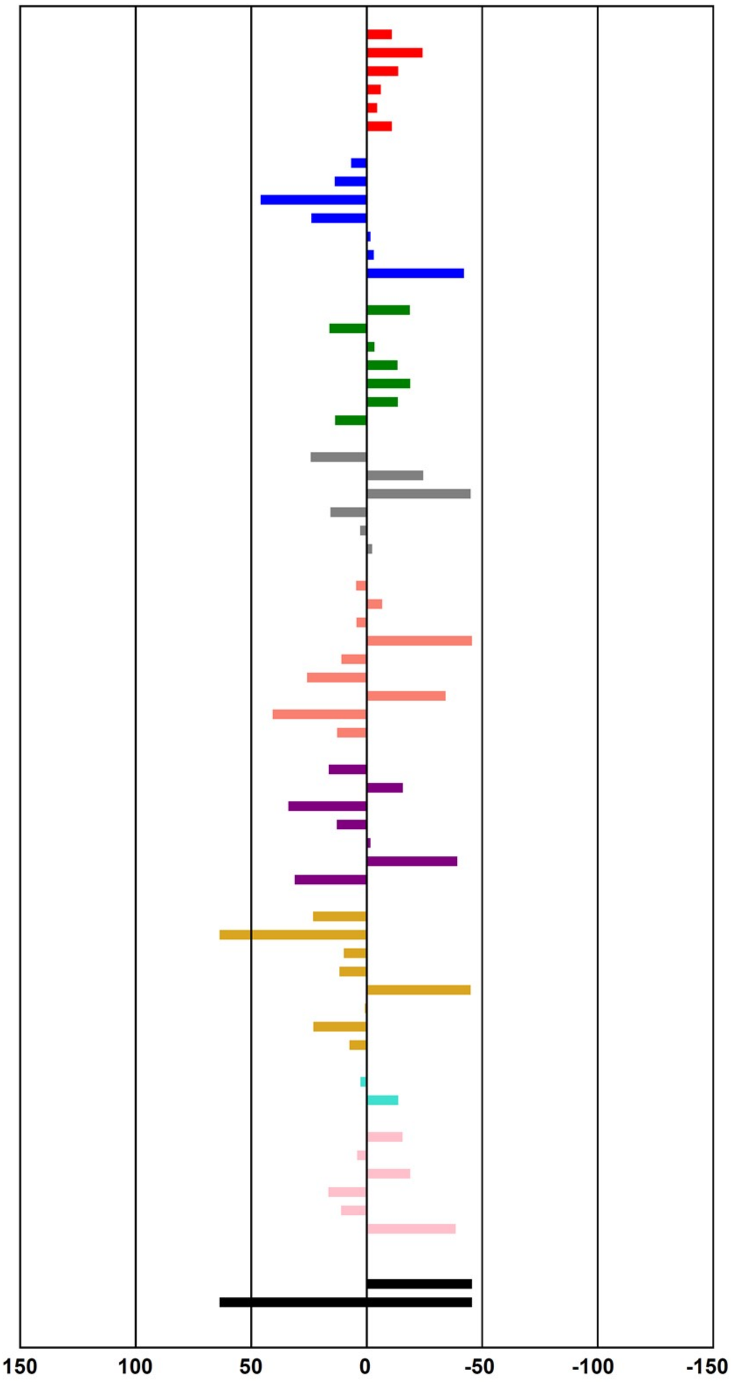

Compound **3w**

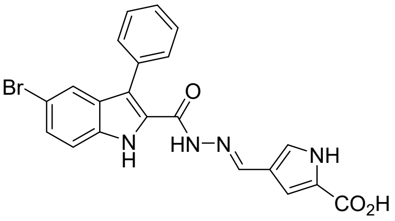

Developmental Therapeutics Program	NSC: D-843110 / 1	Conc: 1.00E-5 Molar	Test Date: Mar 20, 2023
One Dose Mean Graph	Experiment ID: 2303OS10		Report Date: Apr 24, 2023

Panel/Cell Line	Growth Percent
Leukemia	
CCRF-CEM	39.09
HL-60(TB)	29.87
K-562	36.41
MOLT-4	67.72
RPMI-8226	84.10
SR	37.02
Non-Small Cell Lung Cancer	
A549/ATCC	94.89
HOP-62	82.68
HOP-92	75.80
NCI-H226	78.99
NCI-H23	89.70
NCI-H460	95.90
NCI-H522	33.49
Colon Cancer	
COLO 205	66.38
HCC-2998	72.82
HCT-116	80.14
HCT-15	40.57
HT29	95.61
KM12	77.93
SW-620	82.80
CNS Cancer	
SF-268	59.35
SF-295	43.16
SF-539	67.43
SNB-19	61.49
SNB-75	66.52
U251	67.03
Melanoma	
LOX IMVI	49.73
MALME-3M	76.23
M14	80.47
MDA-MB-435	70.86
SK-MEL-2	78.77
SK-MEL-28	79.33
SK-MEL-5	75.43
UACC-257	96.67
UACC-62	38.92
Ovarian Cancer	
IGROV1	87.52
OVCAR-3	31.14
OVCAR-4	56.18
OVCAR-5	88.63
OVCAR-8	69.37
NCI/ADR-RES	38.27
SK-OV-3	89.69
Renal Cancer	
786-0	74.76
A498	100.31
ACHN	68.31
CAKI-1	70.42
RXF 393	66.62
SN12C	61.32
TK-10	92.50
UO-31	53.20
Prostate Cancer	
PC-3	60.87
DU-145	89.90
Breast Cancer	
MCF7	37.95
MDA-MB-231/ATCC	62.39
HS 578T	64.68
BT-549	99.95
T-47D	60.17
MDA-MB-468	19.00
Mean	67.53
Delta	48.53
Range	81.31

Mean Growth Percent - Growth Percent

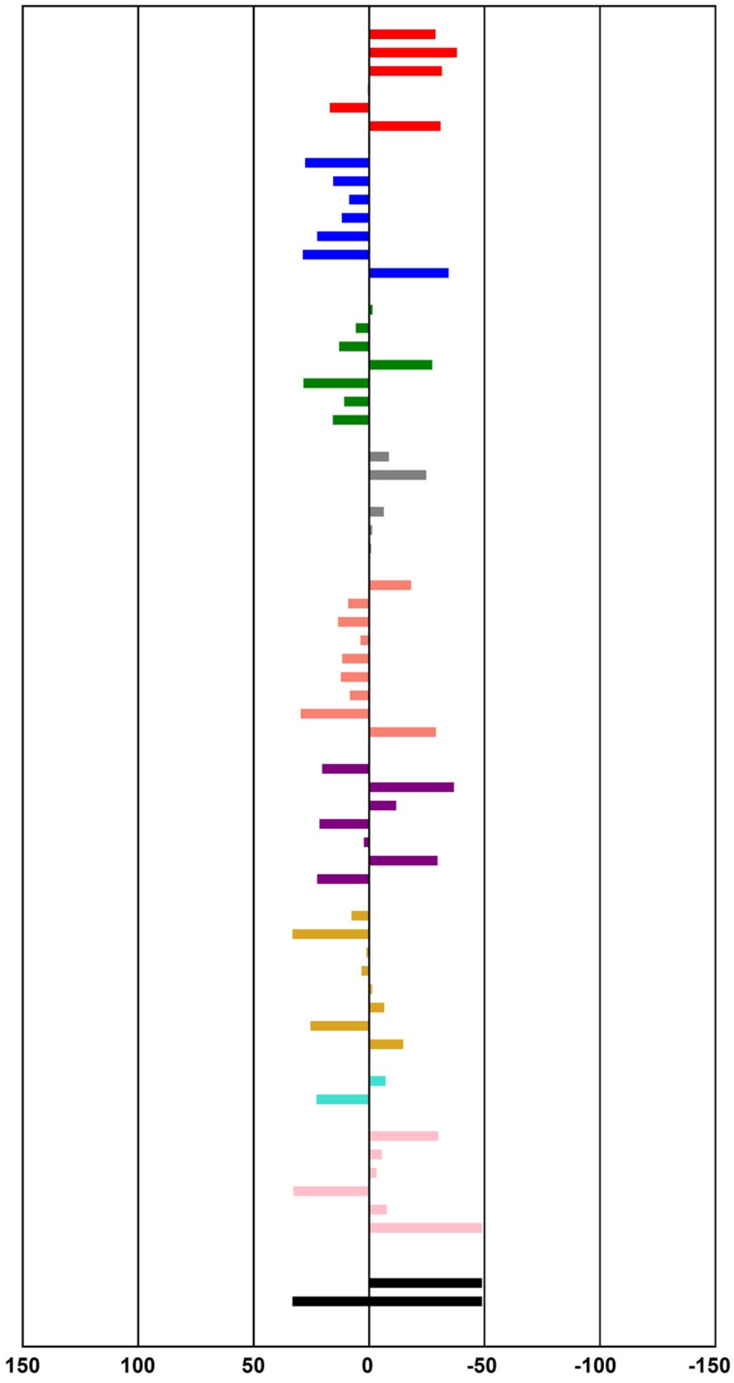

Compound **3x**

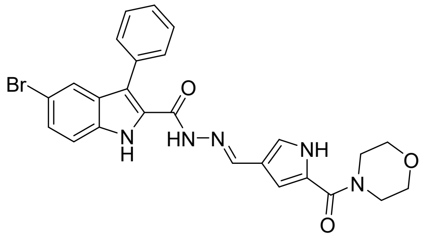

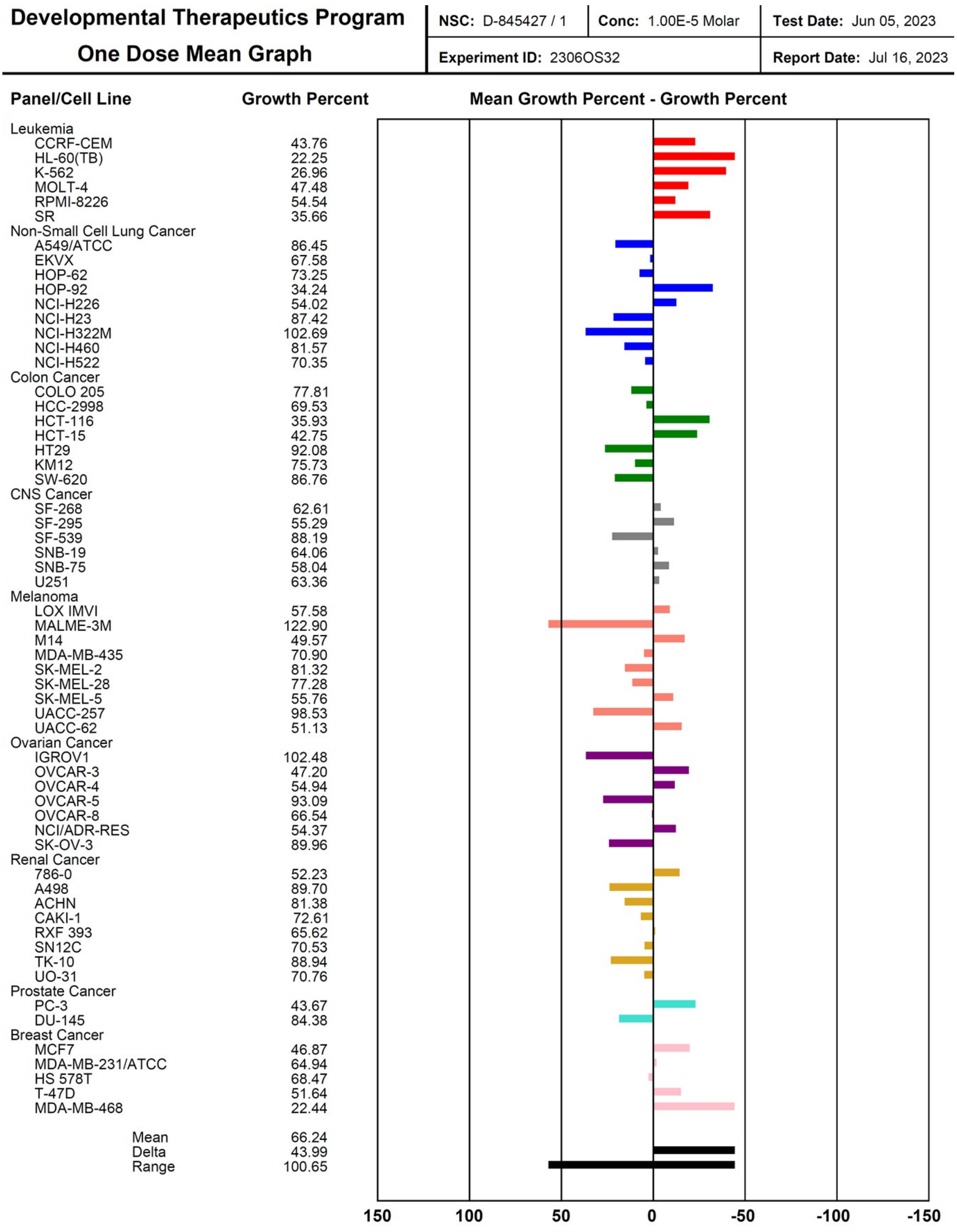

Table S2. GI_{50} and LC_{50} screen for compounds **3a–j**, **3n**, **and 3p–v**

Disease	Cell line	GI50, μM									
		3a	3b	3c	3d	3e	3f	3g	3h	3i	3j
**leukemia**	CCRF-CEM	0.345	0.821	3.07	0.429	0.341	2.89	1.53	0.132	3.04	2.41
	HL-60 (TB)	0.242	0.598	2.4	0.317	0.327	2.38	0.891	0.185	1.84	1.5
	K-562	0.202	0.429	2.43	0.327	0.393	1.93	0.413	0.234	1.08	1.9
	MOLT-4	0.384	1.52	2.29	0.438	0.375	3.38	1.71	0.283	2.49	2.1
	RPMI-8226	0.337	1.43	2.43	0.61	0.346	2.27	0.855	0.446	2.28	2.52
	SR	0.160							0.179		0.948
**NSC lung**	A549/ATCC	0.357	1.61	3.82	0.649	0.395	3.11	0.838	0.43	3.25	2.7

**cancer**	EKVX	1.23	4.41	3.24	0.641	0.62	3.45	2.66	1.28	4.29	4.57
	HOP-62	0.684	2.01	2.97	0.57	0.546	3.52	3.8	0.408	3.65	2.78
	HOP-92	0.992	1.87	2.61	0.429	1.51	1.88	2.79	1.19	2.33	4.56
	NCI-H226	0.323	2.52	2.58	0.603	0.382	2.28	1.44	0.604	3.13	3.07
	NCI-H23	0.504	1.87	2.59	0.447	0.298	2.62	0.884	0.317	2.59	3.06
	NCI-H322M	0.661	2.38	2.69	1.98	0.578	3.73	3.14	0.768	3.3	5.63
	NCI-H460	0.318	0.657	2.83	0.442	0.353	2.53	0.448	0.281	2.8	2.41
	NCI-H522	0.045	0.925	2.61	0.217	0.185	0.623	0.233	0.211	0.46	2.27
**Colon cancer**	COLO 205	0.261	1.23	3.25	0.366	0.192	3.36	1.66	0.251	3.15	2.49
	HCC-2998	0.415	1.55	3.55	0.402	0.296	2.3	1.23	0.224	2.51	2.18
	HCT-116	0.28	0.991	4.02	0.427	0.242	3.24	0.467	0.331	3.24	2.95
	HCT-15	0.269	0.918	2.91	0.228	0.28	2.7	0.465	0.302	2.48	2.93
	HT29	0.329	0.68	3.53	0.327	0.345	2.33	0.401	0.287	2.32	2.59
	KM12	0.388	0.996	2.96	0.357	0.301	2.46	0.488	0.328	2.22	2.21
	SW-620	0.352	0.548	3.06	0.432	0.405	2.96	0.499	0.328	3.93	3.36
**CNS cancer**	SF-268	0.995	2.41	2.98	0.547	0.529	2.57	1.76	0.388	2.68	2.29
	SF-295	0.224	1.94	2.76	0.321	0.254	2.2	0.501	0.237	1.8	2.34
	SF-539	0.202	0.869	2.93	0.217	0.214	1.78	0.378	0.231	1.6	2.26
	SNB-19	0.416	2.55	3.86	0.449	0.330	3.47	1.06	0.638	2.98	4.51
	SNB-75	0.136	0.88	2.68	0.259	0.475	1.08	0.325	0.247	0.399	1.09
	U251	0.375	2.08	3.21	0.477	0.418	2.95	0.63	0.349	2.82	2.66
**Melanoma**	LOX IMVI	0.244	0.916	2.43	0.494	0.36	2.31	0.555	0.37	2.26	2.82
	MALME-3M	0.320	3.46	3.16	0.409	0.665	2.97	0.596	0.645	3.83	6.06
	M14	0.304	1.58	4.45	0.324	0.27	2.81	0.689	0.305	2.73	2.39
	MDA-MB-435	0.105	0.463	2.84	0.151	0.207	1.01	0.222	0.185	0.464	1.09
	SK-MEL-2	0.357	2.48	3.37	0.366	0.322	2.68	1.07	0.26	2.38	2.8
	SK-MEL-28	0.488	5.75	3.81	4.7	0.612	5.1	0.909	0.582	2.65	4.56
	SK-MEL-5	0.302	1.1	2.52	0.196	0.195	1.72	0.427	0.228	1.48	1.44
	UACC-257	0.647	8.22	4.35	0.416	1.09	3.24	0.738	0.856	2.97	2.53
	UACC-62	0.099	1.03	2.36	0.283	0.439	1.2	0.411	0.559	0.614	2.92
**Ovarian Cancer**	IGROV1	0.404	1.27	2.47	0.621	0.44	0.53	0.734	0.538	2.02	3.91
	OVCAR-3	0.423	1.09	2.53	0.246	0.302	2.3	0.409	0.27	2.63	2.13
	OVCAR-4	0.814	4.82	3.55	0.637	1.35	5.2	3.09	0.555	4.26	2.28
	OVCAR-5	0.437	1.74	3.63	0.472	0.346	3.6	1.1	0.473	3.43	3.87
	OVCAR-8	0.323	1.21	3.1	0.39	0.304	1.98	0.481	0.214	2.49	2.37
	NCI/ADR-RES	0.44	0.636	2.11	0.186	0.197	1.14	0.219	0.2	0.897	1.89
	SK-OV-3	0.702	5.59	6.81	1.61	0.966	4.76	11.6	0.519	4.01	4.53
**Renal Cancer**	786-0	0.791	5.46	5.19	29.4	0.727	4.91	2.58	0.459	4.48	3.29
	A498	2.57	12.7	8.25	>100	5.47	11.1	10.6	1.45	10.5	3.53
	ACHN	0.355	1.71	3.03	0.175	0.418	2.59	0.627	0.493	1.86	3.39
	CAKI-1	0.257	0.776	3.17	0.255	0.329	1.38	0.445	0.253	0.961	1.46
	RXF 393	0.339	1.78	2.26	0.28	0.497	1.55	0.346	0.219	1.69	1.36
	SN12C	0.371	1.49	2.58	0.497	0.310	2.15	1.34	0.391	2.86	2.49
	TK-10	2.46	11.1	7.32	1.01	4.21	6.8	7.16	3.91	6.92	7.35
	UO-31	0.431	1.66	1.77	0.33	0.618	1.94	0.577		1.48	
**Prostate Cancer**	PC-3	0.344	2	2.06	0.564	0.382	2.29	0.826	0.426	2.28	3.76
	DU-145	0.429	1.95	4.07	0.399	0.352	3.18	1.58	0.352	3.32	3.48
**Breast Cancer**	MCF7	0.136	0.436	2.3	0.072	0.294	1.99	0.376	0.309	1.54	2.76
	MDA-MB-231/ATCC	0.396	1.62	2.78	0.237	0.215	2.49	2.07	0.296	2.63	2.85
	HS 578T	0.452	1.31	2.31	0.22	0.34	1.82	0.59	0.284	1.21	2.36
	BT-549	0.375	4.53	5.04	2.81	0.314	3.69	3.56	0.255	3.47	2.23
	T-47D	0.341	3.49	4.68	0.344	1.4	3.84	2.5	0.546	4.2	2.91
	MDA-MB-468	0.209	0.593	2.00	0.155	0.231	2.09	0.297	0.251	1.63	2.47

**Disease**	**Cell line**	**GI_{50}, μM**							
		**3n**	**3p**	**3q**	**3r**	**3s**	**3t**	**3u**	**3v**
**leukemia**	CCRF-CEM	3.45	0.382	0.799	1.89	0.375	0.368	2.35	3.7
	HL-60 (TB)	1.84	0.374	0.633	2.31	0.235	0.237	2.03	4.68
	K-562	1.27	0.365	0.387	2.69	0.338	0.348	2.27	4.59
	MOLT-4	1.5	0.537	0.657	2.29	0.464	0.399	2.37	3.91
	RPMI-8226	4.5	0.468	3.71	2.70	0.375	0.343	2.04	3.84
	SR	0.405	0.354	0.405	2.04	0.419	0.446	2.02	
**NSC lung cancer**	A549/ATCC	3.88	2.23	1.79	3.19	0.399	0.397	2	4.37
	EKVX	7.51	5.37	>100	3.34	0.453	0.448	2.79	7.14
	HOP-62	3.36	0.606	1.26	2.09	0.861	1.35	3.77	6.66

	HOP-92	6.14	1.79	11.6	2.51	2.01	2.57	3.8	7.99
	NCI-H226	3.39	2.43	3.39	3.78	0.506	0.33	1.97	4.21
	NCI-H23	2.83	1.45	2.57	2.46	0.484	0.408	2.66	3.42
	NCI-H322M	13.9	11	46.4	4.22	1.73	2.31	3.93	4.42
	NCI-H460	2.49	0.421	0.328	1.87	0.396	0.346	1.23	3.49
	NCI-H522	1.6	0.588	0.722	2.43	0.183	0.206	2.3	2.41
**Colon cancer**	COLO 205	3.12	1.52	1.28	1.9	0.316	0.207	2.96	3.51
	HCC-2998	2.31	1.67	3.66	2.41	1.45	0.342	4.11	4.17
	HCT-116	2.79	0.439	0.491	1.98	0.325	0.385	2.5	3.42
	HCT-15	2.62	0.607	0.504	2.41	0.349	0.319	1.03	3.21
	HT29	2.57	0.55	0.469	1.84	0.298	0.308	2.17	3.33
	KM12	2.91	1.17	1.39	1.92	0.289	0.325	3.17	3.37
	SW-620	3.26	0.249	0.38	2.06	0.558	0.431	3.82	4.41
**CNS cancer**	SF-268	2.51	1.47	0.774	2.37	1.47	0.861	2.86	6.04
	SF-295	1.63	0.413	0.623	2.32	0.246	0.319	2.29	3.04
	SF-539	2.23	0.389	0.822	3.51	0.225	0.203	3.17	2.02
	SNB-19	5.66	3.34	6.25	3.45	1.82	0.396	4.5	4.08
	SNB-75	0.809	0.482	0.242	2.19	0.285	0.44	4.94	1.95
	U251	3.1	0.987	0.574	1.85	0.329	0.373	2.15	3.8
**Melanoma**	LOX IMVI	3.06	0.519	0.647	1.63	0.722	0.427	2.42	4.31
	MALME-3M	11.7	3.23	0.83	1.99	0.88	1.72	3.5	4.32
	M14	1.92	0.652	0.574	2.54	0.318	0.337	1.62	3.54
	MDA-MB-435	0.392	0.253	0.226	1.77	0.227	0.23	4.12	1.54
	SK-MEL-2	2.8	2.18	1.3	2.11	0.647	0.454	6.77	4.37
	SK-MEL-28	6.1	2.24	0.89	3.56	0.979	0.839	3.46	4.73
	SK-MEL-5	1.48	1.02	0.671	1.5	0.381	0.279	2.54	2.01
	UACC-257	11.9	3.12	2.58	3.13	1.32	1.23	3.78	11.7
	UACC-62	0.864	0.451	0.538	2.9	0.381	0.442	2.48	3.18
**Ovarian Cancer**	IGROV1	6.69	1.48	2.06	3.83	0.612	0.888	3.52	3.75
	OVCAR-3	2.57	0.602	0.397	1.77	0.356	0.316	3.2	3.23
	OVCAR-4	2.93	0.679	1.06	1.97	2.41	2.17	3.33	9.00
	OVCAR-5	5.78	2.87	5.37	3.53	0.573	0.337	6.77	3.49
	OVCAR-8	2.86	1.02	10.2	3.08	0.337	0.316	3.02	3.65
	NCI/ADR-RES	1.92	0.285	0.434	1.89	0.253	0.247	2.68	2.23
	SK-OV-3	5.68	2.95	3.14	3.23	0.514	0.922	2.59	1.31
**Renal Cancer**	786-0	2.86	0.803	2.21	2.35	0.801	0.646	3.51	6.01
	A498	3.53	2.6	10.7	2.35	5.59	13.7	1.45	12.9
	ACHN	4.56	1.26	0.791	3.3	0.861	0.628	3.27	4.87
	CAKI-1	1.46	0.411	0.53	2.29	1.04	1.05	6.04	3.2
	RXF 393	1.06	0.411	0.734	1.7	0.194	0.217	1.19	2.56
	SN12C	3.14	1.24	2.58	1.74	0.392	0.372	2.26	3.63
	TK-10	10.4	12.2	76	5.1	2.91	2.83	5.05	12.5
	UO-31					0.644	1	2.94	4.21
**Prostate Cancer**	PC-3	3.44	1.2	0.724	2.85	0.532	0.529	3.09	4.07
	DU-145	3.72	1.84	3.87	3.48	0.418	0.38	4.3	3.36
**Breast Cancer**	MCF7	2.46	0.394	0.354	2.57	0.369	0.375	2.63	3.03
	MDA-MB-231/ATCC	4.81	2.14	17.4	3.56	0.63	0.311	5.02	3.27
	HS 578T	1.04	0.27	0.486	2.43	0.294	0.3	3.6	3.11
	BT-549	2.36	1.52	2.47	2.74	0.979	0.39	2.7	13.4
	T-47D	3.1	2.4	2.21	2.51	0.681	1.59	2.17	5.88
	MDA-MB-468	1.98	0.873	0.503	1.94	0.248	0.263	2.72	2.6

**Disease**	**Cell line**	**LC_{50}, µM**									
		**3a**	**3b**	**3c**	**3d**	**3e**	**3f**	**3g**	**3h**	**3i**	**3j**
**leukemia**	CCRF-CEM	>100	>100	>100	>100	>100	>100	>100	>100	>100	>100
	HL-60 (TB)	>100	>100	>100	>100	>100	88.8	>100	**0.886**	>100	**9.88**
	K-562	>100	97	>100	>100	>100	76.4	>100	**5.64**	>100	57.1
	MOLT-4	>100	>100	>100	>100	>100	>100	>100		>100	53.4
	RPMI-8226	>100	>100	>100	>100	>100	>100	>100	>100	>100	>100
	SR	>100							**4.6**		>100
**NSC lung cancer**	A549/ATCC	33.6	52.6	45.4	>100	56.4	37.6	53.4	47.4	79.4	51.8
	EKVX	33.4	41.7	35.4	>100	34.3	44.8	38.1	>100	>100	43.1
	HOP-62	32.6	61.4	69.3	>100	>100	>100	60.8	34.7	>100	54.4

	HOP-92	26.4	50.3	43.5	>100	26.3	46	50.1	55.4	>100	58.6
	NCI-H226	23.3	51.2	34.4	>100	29.9	32.5	32.1	>100	45.4	41.3
	NCI-H23	37.7	33.9	35.2	>100	26.9	34	34.4	36.5	43.2	46.4
	NCI-H322M	36.5	37.2	38.7	>100	38.8	62.8	48.8	92.3	>100	50
	NCI-H460	12.5	46	37.9	>100	43.2	32.7	34.3	**9.24**	41.4	23.1
	NCI-H522	22	24	32.7	>100	19.1	23.2	20.4	>100	82.2	33
**Colon cancer**	COLO 205	35.1	20.4	52.3	>100	**0.884**	48.8	40.3	**1.88**	36.9	55.2
	HCC-2998	19.5	32.7	37.2	>100	15.6	24	28.6	**4.2**	36.4	15.9
	HCT-116	32.3	>100	>100	>100	85	>100	79.9	29.1	>100	45.6
	HCT-15	32.3	34	37.2	>100	13.5	33.1	34.3	21.9	40.5	51.9
	HT29	**9.31**	34.5	46.6	>100	**8.24**	20.8	58.5	**7.2**	22.9	27.5
	KM12	56.5	37.8	35.4	>100	16.3	35.3	34.6	20.7	38	33.9
	SW-620	37.4	49.4	42	>100	59.4	41.9	50.2	80.9	53.6	76.2
**CNS cancer**	SF-268	56.4	59.1	53.2	>100	52.9	65.3	53	77.3	>100	46.3
	SF-295	23.2	33.8	29.5	>100	25.9	26.9	26	58.5	38.1	24.4
	SF-539	14.6	17	32.4	>100	25.5	15.7	18.5	22.1	26.2	19.4
	SNB-19	35	39.8	39.4	>100	34.5	39.3	35.4	61.9	62.8	46.1
	SNB-75	16	32	43.9	>100	51	64.4	38.6	75.5	>100	35.8
	U251	12.4	40.8	35.7	>100	10.1	36	37.7	24.9	39.4	40.6
**Melanoma**	LOX IMVI	19	38.7	30.2	>100	**6.98**	31.8	29.4	16.7	35.2	40.9
	MALME-3M	30.9	48.3	38.5	>100	32.5	42.4	48.4	46.1	54.8	54.9
	M14	41.4	45.8	49.9	>100	20.2	66.1	45.4	50.3	71.9	50.3
	MDA-MB-435		15.8	34		24.3	19.6	25.5		27.6	16.1
	SK-MEL-2	38.8	38.4	41.7	>100	38.1	36.3	38.7	47.9	55	32.8
	SK-MEL-28	37.4	44.4	38.8	>100	43.9	47	47.5	47.9	94.5	45.8
	SK-MEL-5	15.7	14.6	28.8	>100	**1.22**	**8.65**	63	**3.08**	**7.68**	10.1
	UACC-257	47.1	47.8	49.2	>100	47	45.7	52	58.8	87.4	51.8
	UACC-62	**8.03**	41.3	32.4	>100	17.7	38	37	66.3	48.5	42.6
**Ovarian Cancer**	IGROV1	14.6	43.6	38.4	>100	36.2	36.1	43	44.1	41.5	46.1
	OVCAR-3	35.3	31.9	36.4	>100	17.3	24.5	34.9	19.7	40.7	23.1
	OVCAR-4	33.6	45.2	40.7	>100	40.5	45.8	43.5	34.1	>100	34.8
	OVCAR-5	30.8	38.5	37.6	>100	26.5	40.2	35.7	36	38	42.9
	OVCAR-8	**6.55**	38.6	45	>100	18.2	17.2	27.7	**0.988**	47.6	23.7
	NCI/ADR-RES	59.2	27.1	19.3	40.9	**0.971**	**9.73**	10.5		**9.2**	10.4
	SK-OV-3	31.8	71.9	70.8	>100	53.8	72.5	64.1	45.4	>100	45.3
**Renal Cancer**	786-0	23.9	77.2	63.9	>100	55.9	64.1	50.2	39.8	>100	44.7
	A498	41.6	54.6	46.9	>100	44.5	49.4	49.3	42.1	55.2	43.4
	ACHN	21.2	39	34.2	>100	23.5	35.5	34.5	32.1	73.8	36.4
	CAKI-1	30.9	44.1	39.1	>100	29	40.9	36.9	27.5	74.2	40.5
	RXF 393	35.1	46.5	35	>100	37.9	34.3	42.1	34.3	66.4	22
	SN12C	**3.97**	37.7	32.3	>100	**5.49**	30.7	29.2	15.3	36.2	33.3
	TK-10	37.1	58	45.2	>100	44.9	49.5	49.5	83.2	>100	46.2
	UO-31	20.2	41	33.8	>100	28.1	38.5	35.7		>100	
**Prostate Cancer**	PC-3	29.6	>100	47.6	>100	38.1	63.3	63.6	47.6	>100	>100
	DU-145	33.3	34.7	38.9	>100	29.7	33.9	33.1	27.2	38.7	35.6
**Breast Cancer**	MCF7	29.1	49.8	37.2	>100	30.7	33.7	42.2	53.5	>100	50.2
	MDA-MB-231/ATCC	21	26.4	32.8	>100	14.3	26.3	28.2	26.9	33.2	36.8
	HS 578T	>100	>100	>100	>100	>100	>100	>100	>100	>100	>100
	BT-549	**6.39**	52.3	47.9	>100	36.2	90.8	46.8	18.1	>100	29.5
	T-47D	51.3	>100	77.6	>100	82.5	74.3	81.9	92.6	>100	86.9
	MDA-MB-468	42.1	24.2	26.4	>100	13	22.8	24.7	27.8	36.6	24.2

**Disease**	**Cell line**	**LC_{50}, μM**							
		**3n**	**3p**	**3q**	**3r**	**3s**	**3t**	**3u**	**3v**
**leukemia**	CCRF-CEM	>100	>100	>100	>100	>100	>100	63.8	>100
	HL-60 (TB)	**8.56**	>100	>100	>100	>100	91.9	24.3	>100
	K-562	42.7	>100	>100	>100	>100	>100	56.2	>100
	MOLT-4	20.8	>100	>100	>100	>100	94.2	55.1	>100
	RPMI-8226	>100	>100	>100	>100	>100	>100	78.6	>100
	SR	38.9	>100	>100	>100	>100	>100	>100	
**NSC lung cancer**	A549/ATCC	>100	44.9	>100	35.6	70.3	38.8	41.5	44.2
	EKVX	>100	45.7	>100	34.9	43	35.6	37.7	60.7
	HOP-62	69.2	39.5	>100	11.7	46.2	37.5	45.8	69.2

	HOP-92	>100	47.3	>100	45.8	40.1	33	44.3	>100
	NCI-H226	81.7	46.9	>100	40.6	32.3	28	37.7	46.2
	NCI-H23	69.8	35.4	>100	26.3	42.6	37.4	38.3	40.4
	NCI-H322M	>100	53.3	>100	89.5	64.6	49.8	43.4	76.1
	NCI-H460	54.1	45.9	>100	**8.05**	43.5	20.2	36.7	34.3
	NCI-H522	58.8	19.8	>100	29.6	28	29.5	34.1	61.2
**Colon**	COLO 205	37.5	**8.08**	>100	**7.51**	26.1		41.3	34.8
**cancer**	HCC-2998	42.9	19.6	>100	25.4	35.1	30.4	42.4	42.1
	HCT-116	66.7	33.8	>100	10.4	31.9	40.3	44	50.5
	HCT-15	>100	35.7	>100	26.4	37.4	35.6	38.8	35.7
	HT29	38.3	43.9	>100	**6.67**	33.8	21	39.3	35.3
	KM12	43.6	34.2	>100	11.6	37.5	34.4	42.3	35.5
	SW-620	>100	48.4	>100	19.1	43.2	42.5	43.3	40.1
**CNS**	SF-268	>100	44.2	>100	33.1	83	51.9	42.7	>100
**cancer**	SF-295	31	29.9	>100	21.2	31.9	24.7	37.1	37.2
	SF-539	34.9	28.1	>100	35.7	29.8	19.6	35.6	13.5
	SNB-19	>100	42.4	>100	43.1	51.8	41.3	42.2	43.4
	SNB-75	88.9	40.5	>100	21.5	54.1	37.8	43.4	90.6
	U251	84.7	39.2	66.8	13.4	36.7	38.2	37.8	37.6
**Melanom**	LOX IMVI	71.2	35.8	>100	**5.73**	36.1	**8.27**	32.3	41.1
**a**	MALME-3M	>100	56.2	>100	28.5	46.5	48	40	52.8
	M14	64.2	37	>100	36.3	38.7	38.4	23.7	41.3
	MDA-MB-435	55.4	29.8	**8.78**	17.9	34	38.7	41.6	**9.53**
	SK-MEL-2	48.3	36.5	>100	22.6	45.9	43.8	45.8	47.3
	SK-MEL-28	>100	43.5	>100	38.7	49.8	46.6	40.1	48.5
	SK-MEL-5	17	**8.69**	69.9	**5.87**	10.1	**6.96**	27.2	17.9
	UACC-257	94.4	48.2	>100	48.1	64.7	41.3	40.8	77.8
	UACC-62	63.7	40.9	>100	37.5	38.1	32.1	30.8	40.7
**Ovarian**	IGROV1	>100	88.8	>100	41.2	43.3	48.6	44	38.9
**Cancer**	OVCAR-3	47.4	34.8	>100	10.4	36.1	41.7	44	33.4
	OVCAR-4	>100	35.9	>100	18.8	53.7	43	43.7	52.8
	OVCAR-5	>100	40.9	>100	41.5	38.7	37.2	45.4	37.4
	OVCAR-8	43.1	39	>100	36.5	25.2	26	39.8	38.5
	NCI/ADR-RES	84.1	16.2	>100	**9.15**	30.2	22	34.6	21.2
	SK-OV-3	>100	43	>100	39.2	50.1	35.4	42.4	80.3
**Renal**	786-0	80.7	41.4	>100	17	47.4	38.5	44.3	53.3
**Cancer**	A498	59.9	43.7	>100	27.6	47.2	54.5	55.4	52.1
	ACHN	>100	38	>100	33.4	36.6	35.5	36.7	49.9
	CAKI-1	>100	34.9	>100	33.1	56.2	34.4	45.3	40.2
	RXF 393	14.7	41.2	>100	15.9	28.9	21	18.7	33
	SN12C	79.7	36.7	>100	**6.73**	30.3	30	34	38.1
	TK-10	>100	53.8	>100	41.2	46.3	42.1	41.6	80.7
	UO-31					59.9	40.7	39.7	61.9
**Prostate**	PC-3	>100	78.5	>100	45.5	>100	57	47.6	>100
**Cancer**	DU-145	>100	32.5	>100	34.9	36.7	39.5	42.3	33.4
**Breast**	MCF7	77.8	39	>100	29.9	45.9	33.8	37.5	40.3
**Cancer**	MDA-MB-231/ATCC	83.2	46.6	>100	40.5	48.4	42.4	44.7	33.8
	HS 578T	>100	94.3	>100	97.2	>100	87.3	61.8	>100
	BT-549	55.2	28.4	>100	30.6	30.3	23.9	34.2	60.2
	T-47D	>100	59.6	>100	33.5	52.7	39.3	41.9	>100
	MDA-MB-468	75.5	34.7	>100	26.1	25.9	**9.22**	32.1	34

Figure S3. NCI60 five dose dcreen

Compound **3a**

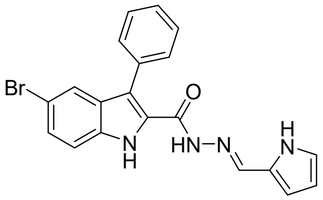
Br
O
HN–N
H
N
N
H

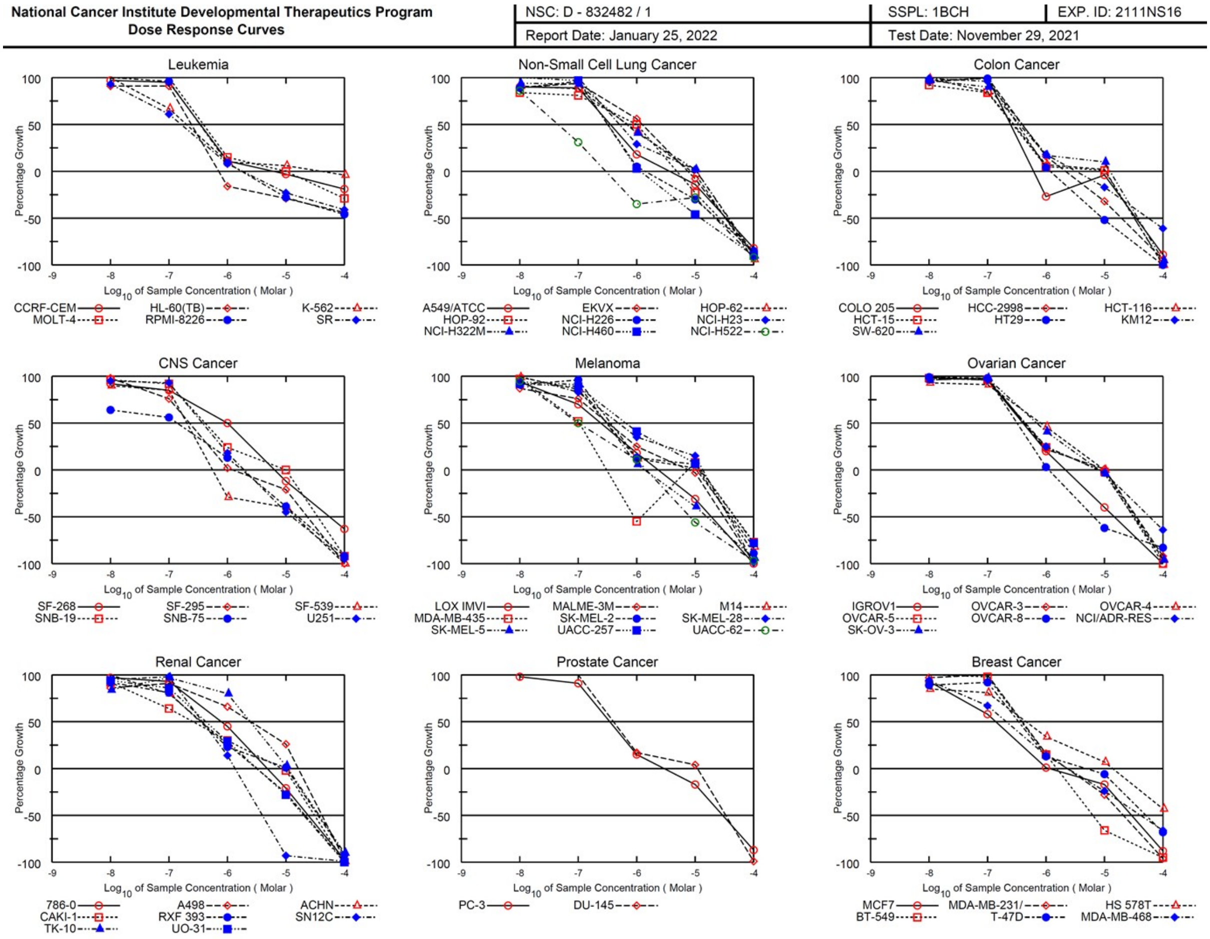
National Cancer Institute Developmental Therapeutics Program
Dose Response Curves
NSC: D - 832482 / 1
Report Date: January 25, 2022
SSPL: 1BCH
EXP. ID: 2111NS16
Test Date: November 29, 2021
Leukemia
Non-Small Cell Lung Cancer
Colon Cancer
CNS Cancer
Melanoma
Ovarian Cancer
Renal Cancer
Prostate Cancer
Breast Cancer
Percentage Growth
Log10 of Sample Concentration (Molar)
CCRF-CEM
HL-60(TB)
K-562
MOLT-4
RPMI-8226
SR
A549/ATCC
EKVX
HOP-62
HOP-92
NCI-H226
NCI-H23
NCI-H322M
NCI-H460
NCI-H522
COLO 205
HCC-2998
HCT-116
HCT-15
HT29
KM12
SW-620
SF-268
SF-295
SF-539
SNB-19
SNB-75
U251
LOX IMVI
MALME-3M
M14
MDA-MB-435
SK-MEL-2
SK-MEL-28
SK-MEL-5
UACC-257
UACC-62
IGROV1
OVCAR-3
OVCAR-4
OVCAR-5
OVCAR-8
NCI/ADR-RES
SK-OV-3
786-0
A498
ACHN
CAKI-1
RXF 393
SN12C
TK-10
UO-31
PC-3
DU-145
MCF7
MDA-MB-231/
HS 578T
BT-549
T-47D
MDA-MB-468

National Cancer Institute Developmental Therapeutics Program
In-Vitro Testing Results

NSC : D - 832482 / 1	Experiment ID : 2111NS16	Test Type : 08	Units : Molar
Report Date : January 25, 2022	Test Date : November 29, 2021	QNS :	MC :
COMI : TO10	Stain Reagent : SRB Dual-Pass Related	SSPL : 1BCH	

			Log10 Concentration												
	Time		Mean Optical Densities					Percent Growth							
Panel/Cell Line	Zero	Ctrl	-8.0	-7.0	-6.0	-5.0	-4.0	-8.0	-7.0	-6.0	-5.0	-4.0	GI50	TGI	LC50
**Leukemia**															
CCRF-CEM	0.530	2.650	2.588	2.551	0.765	0.514	0.428	97	95	11	-3	-19	3.45E-7	6.04E-6	> 1.00E-4
HL-60(TB)	0.572	2.412	2.250	2.251	0.478	0.404	0.322	91	91	-16	-29	-44	2.42E-7	7.04E-7	> 1.00E-4
K-562	0.154	1.729	1.752	1.215	0.318	0.242	0.149	101	67	10	6	-4	2.02E-7	4.06E-5	> 1.00E-4
MOLT-4	0.590	2.538	2.546	2.533	0.876	0.595	0.417	100	100	15	0	-29	3.84E-7	1.02E-5	> 1.00E-4
RPMI-8226	0.581	2.495	2.487	2.412	0.755	0.418	0.316	100	96	9	-28	-46	3.37E-7	1.75E-6	> 1.00E-4
SR	0.371	1.766	1.662	1.219	0.482	0.284	0.220	93	61	8	-23	-41	1.60E-7	1.79E-6	> 1.00E-4
**Non-Small Cell Lung Cancer**															
A549/ATCC	0.337	2.042	1.870	1.856	0.649	0.290	0.059	90	89	18	-14	-82	3.57E-7	3.69E-6	3.36E-5
EKVX	1.027	1.909	1.810	1.859	1.518	0.949	0.118	89	94	56	-8	-89	1.23E-6	7.58E-6	3.34E-5
HOP-62	0.707	1.838	1.731	1.698	1.189	0.685	0.039	91	88	43	-3	-94	6.84E-7	8.55E-6	3.26E-5
HOP-92	1.244	1.912	1.805	1.783	1.577	0.962	0.156	84	81	50	-23	-88	9.92E-7	4.87E-6	2.64E-5
NCI-H226	0.822	1.462	1.391	1.440	0.855	0.579	0.123	89	96	5	-30	-85	3.23E-7	1.40E-6	2.33E-5
NCI-H23	0.419	1.396	1.394	1.402	0.698	0.436	0.050	100	101	29	2	-88	5.04E-7	1.05E-5	3.77E-5
NCI-H322M	0.810	1.944	1.877	1.860	1.272	0.832	0.078	94	93	41	2	-90	6.61E-7	1.05E-5	3.65E-5
NCI-H460	0.323	2.992	3.028	2.919	0.407	0.176	0.033	101	97	3	-46	-90	3.18E-7	1.16E-6	1.25E-5
NCI-H522	1.038	2.388	2.194	1.458	0.675	0.745	0.084	86	31	-35	-28	-92	4.50E-8	2.95E-7	2.20E-5
**Colon Cancer**															
COLO 205	0.628	2.557	2.621	2.656	0.458	0.605	0.071	103	105	-27	-4	-89	2.61E-7	6.23E-7	3.51E-5
HCC-2998	0.495	1.561	1.509	1.621	0.662	0.337	0.030	95	106	16	-32	-94	4.15E-7	2.13E-6	1.95E-5
HCT-116	0.226	2.402	2.386	2.078	0.370	0.268	-0.019	99	85	7	2	-100	2.80E-7	1.04E-5	3.23E-5
HCT-15	0.217	1.403	1.307	1.216	0.273	0.228	0.002	92	84	5	1	-99	2.69E-7	1.02E-5	3.23E-5
HT29	0.373	2.314	2.255	2.292	0.460	0.180	-0.001	97	99	4	-52	-100	3.29E-7	1.20E-6	9.31E-6
KM12	0.833	3.347	3.327	3.242	1.286	0.695	0.325	99	96	18	-17	-61	3.88E-7	3.31E-6	5.65E-5
SW-620	0.333	2.464	2.395	2.241	0.699	0.542	0.018	97	90	17	10	-95	3.52E-7	1.24E-5	3.74E-5
**CNS Cancer**															
SF-268	1.079	2.908	2.762	2.631	1.992	0.953	0.403	92	85	50	-12	-63	9.95E-7	6.45E-6	5.64E-5
SF-295	0.472	1.834	1.802	1.505	0.501	0.372	-0.012	98	76	2	-21	-100	2.24E-7	1.23E-6	2.32E-5
SF-539	0.563	1.785	1.665	1.598	0.402	0.337	-0.014	90	85	-29	-40	-100	2.02E-7	5.58E-7	1.46E-5
SNB-19	0.527	1.785	1.737	1.688	0.829	0.533	0.041	96	92	24	0	-92	4.16E-7	1.01E-5	3.50E-5
SNB-75	2.216	3.108	2.790	2.714	2.329	1.346	0.175	64	56	13	-39	-92	1.36E-7	1.75E-6	1.60E-5
U251	0.581	2.798	2.685	2.650	0.978	0.318	0.029	95	93	18	-45	-95	3.75E-7	1.92E-6	1.24E-5
**Melanoma**															
LOX IMVI	0.163	1.211	1.149	0.899	0.351	0.113	-0.001	94	70	18	-31	-100	2.44E-7	2.34E-6	1.90E-5
MALME-3M	0.726	1.433	1.344	1.261	0.902	0.703	0.010	87	76	25	-3	-99	3.20E-7	7.71E-6	3.09E-5
M14	0.470	1.929	1.910	1.723	0.639	0.495	0.084	99	86	12	2	-82	3.04E-7	1.05E-5	4.14E-5
MDA-MB-435	0.779	3.060	3.000	1.976	0.354	0.960	0.176	97	52	-55	8	-77	1.05E-7	.	.
SK-MEL-2	1.232	2.657	2.530	2.596	1.416	1.315	0.136	91	96	13	6	-89	3.57E-7	1.15E-5	3.88E-5
SK-MEL-28	0.523	1.667	1.677	1.470	0.925	0.700	0.006	101	83	35	15	-99	4.88E-7	1.37E-5	3.74E-5
SK-MEL-5	0.640	2.673	2.555	2.484	0.760	0.389	0.039	94	91	6	-39	-94	3.02E-7	1.35E-6	1.57E-5
UACC-257	0.948	2.522	2.404	2.347	1.593	1.065	0.210	92	89	41	7	-78	6.47E-7	1.22E-5	4.71E-5
UACC-62	0.837	2.807	2.699	1.817	1.051	0.365	0.025	95	50	11	-56	-97	9.86E-8	1.45E-6	8.03E-6
**Ovarian Cancer**															
IGROV1	0.501	2.080	2.069	2.022	0.815	0.300	-0.003	99	96	20	-40	-100	4.04E-7	2.14E-6	1.46E-5
OVCAR-3	1.057	2.865	2.796	2.825	1.447	1.076	0.083	96	98	22	1	-92	4.23E-7	1.03E-5	3.53E-5
OVCAR-4	0.841	1.937	1.866	1.837	1.345	0.837	0.045	93	91	46	0	-95	8.14E-7	9.77E-6	3.36E-5
OVCAR-5	0.567	1.640	1.618	1.598	0.826	0.554	-0.006	98	96	24	-2	-100	4.37E-7	8.19E-6	3.08E-5
OVCAR-8	0.512	2.500	2.474	2.467	0.580	0.195	0.086	99	98	3	-62	-83	3.23E-7	1.13E-6	6.55E-6
NCI/ADR-RES	0.370	1.358	1.334	1.318	0.613	0.358	0.134	98	96	25	-3	-64	4.40E-7	7.64E-6	5.92E-5
SK-OV-3	0.967	1.876	1.844	1.858	1.343	0.930	0.043	96	98	41	-4	-96	7.02E-7	8.21E-6	3.18E-5
**Renal Cancer**															
786-0	0.578	2.426	2.377	2.291	1.413	0.455	0.017	97	93	45	-21	-97	7.91E-7	4.77E-6	2.39E-5
A498	1.326	2.103	1.993	2.030	1.843	1.530	0.042	86	91	66	26	-97	2.57E-6	1.63E-5	4.16E-5
ACHN	0.286	1.117	1.148	0.948	0.500	0.211	0.004	104	80	26	-26	-99	3.55E-7	3.11E-6	2.12E-5
CAKI-1	0.680	2.230	2.111	1.668	1.148	0.667	-0.015	92	64	30	-2	-100	2.57E-7	8.67E-6	3.09E-5
RXF 393	0.651	1.193	1.147	1.090	0.773	0.657	0.049	92	81	23	1	-92	3.39E-7	1.03E-5	3.51E-5
SN12C	0.520	1.823	1.759	1.802	0.696	0.039	0.006	95	98	14	-93	-99	3.71E-7	1.34E-6	3.97E-6
TK-10	1.216	2.045	1.915	2.017	1.882	1.239	0.123	84	97	80	3	-90	2.46E-6	1.07E-5	3.71E-5
UO-31	0.500	2.092	1.996	1.874	0.963	0.360	-0.021	94	86	29	-28	-100	4.31E-7	3.23E-6	2.02E-5
**Prostate Cancer**															
PC-3	0.628	2.212	2.175	2.066	0.861	0.521	0.082	98	91	15	-17	-87	3.44E-7	2.90E-6	2.96E-5
DU-145	0.333	1.647	1.668	1.732	0.559	0.380	0.004	102	106	17	4	-99	4.29E-7	1.08E-5	3.33E-5
**Breast Cancer**															
MCF7	0.186	0.894	0.841	0.595	0.191	0.154	0.023	93	58	1	-17	-88	1.36E-7	1.10E-6	2.91E-5
MDA-MB-231/ATCC	0.554	1.370	1.345	1.368	0.689	0.398	0.023	97	100	16	-28	-96	3.96E-7	2.34E-6	2.10E-5
HS 578T	1.525	2.757	2.578	2.519	1.942	1.613	0.874	85	81	34	7	-43	4.52E-7	1.39E-5	> 1.00E-4
BT-549	1.016	2.015	2.031	1.992	1.163	0.350	0.052	102	98	15	-66	-95	3.75E-7	1.52E-6	6.39E-6
T-47D	1.368	3.119	2.934	2.975	1.602	1.281	0.440	89	92	13	-6	-68	3.41E-7	4.74E-6	5.13E-5
MDA-MB-468	0.702	1.384	1.346	1.158	0.801	0.537	0.239	94	67	14	-24	-66	2.09E-7	2.40E-6	4.21E-5

Compound **3b**

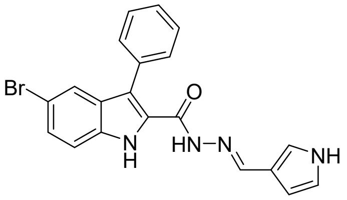

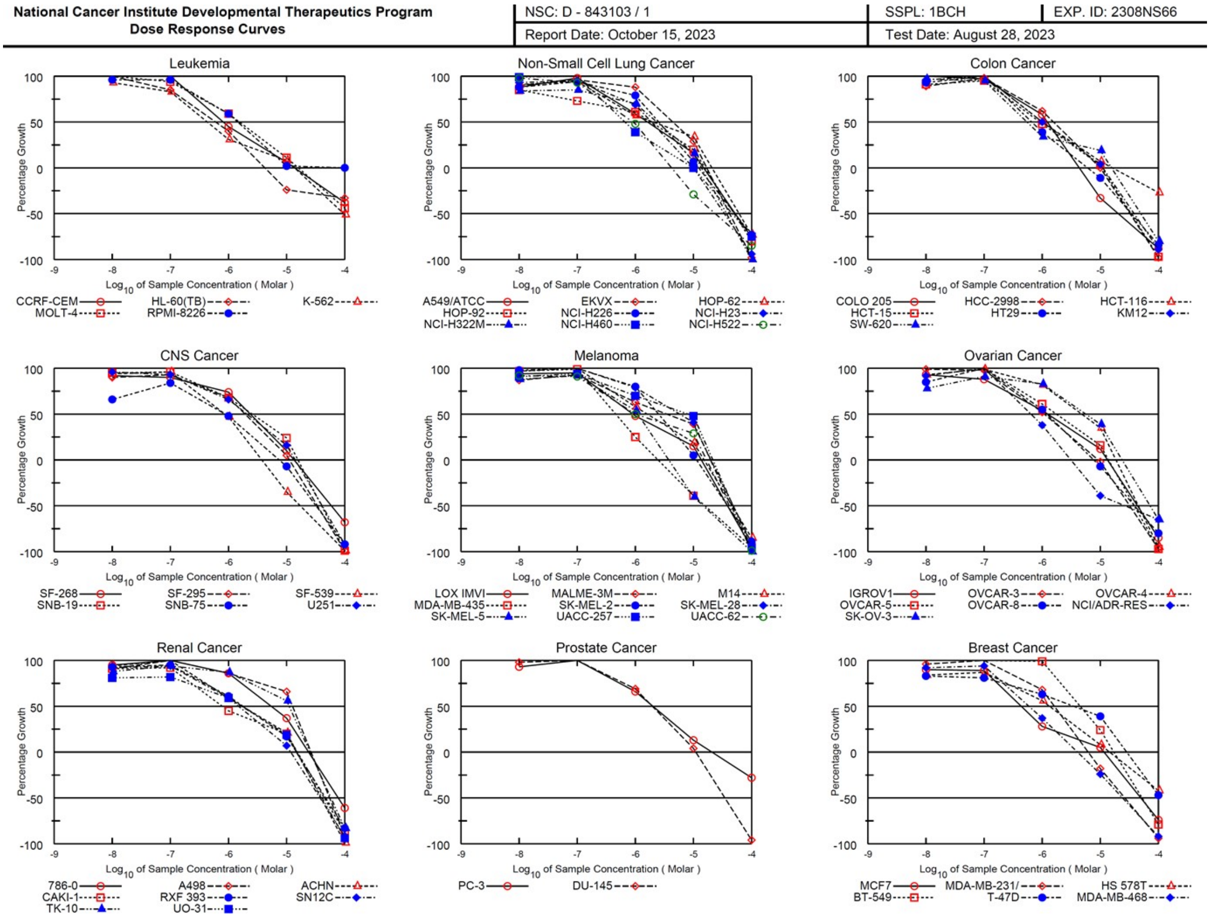
National Cancer Institute Developmental Therapeutics Program
Dose Response Curves
NSC: D - 843103 / 1
Report Date: October 15, 2023
SSPL: 1BCH
EXP. ID: 2308NS66
Test Date: August 28, 2023
Leukemia
CCRF-CEM HL-60(TB) K-562 MOLT-4 RPMI-8226
Non-Small Cell Lung Cancer
A549/ATCC EKVX HOP-62 HOP-92 NCI-H226 NCI-H23 NCI-H322M NCI-H460 NCI-H522
Colon Cancer
COLO 205 HCC-2998 HCT-116 HCT-15 HT29 KM12 SW-620
CNS Cancer
SF-268 SF-295 SF-539 SNB-19 SNB-75 U251
Melanoma
LOX IMVI MALME-3M M14 MDA-MB-435 SK-MEL-2 SK-MEL-28 SK-MEL-5 UACC-257 UACC-62
Ovarian Cancer
IGROV1 OVCAR-3 OVCAR-4 OVCAR-5 OVCAR-8 NCI/ADR-RES SK-OV-3
Renal Cancer
786-0 A498 ACHN CAKI-1 RXF 393 SN12C TK-10 UO-31
Prostate Cancer
PC-3 DU-145
Breast Cancer
MCF7 MDA-MB-231/ HS 578T BT-549 T-47D MDA-MB-468
Percentage Growth
Log10 of Sample Concentration (Molar)

National Cancer Institute Developmental Therapeutics Program
In-Vitro Testing Results

NSC : D - 843103 / 1	Experiment ID : 2308NS66	Test Type : 08	Units : Molar
Report Date : October 15, 2023	Test Date : August 28, 2023	QNS :	MC :
COMI : TO41	Stain Reagent : SRB Dual-Pass Related	SSPL : 1BCH	

			Log10 Concentration												
	Time		Mean Optical Densities					Percent Growth							
Panel/Cell Line	Zero	Ctrl	-8.0	-7.0	-6.0	-5.0	-4.0	-8.0	-7.0	-6.0	-5.0	-4.0	GI50	TGI	LC50
Leukemia															
CCRF-CEM	0.489	2.589	2.613	2.596	1.440	0.625	0.306	101	100	45	6	-38	8.21E-7	1.40E-5	> 1.00E-4
HL-60(TB)	0.609	2.652	2.719	2.338	1.428	0.462	0.406	103	85	40	-24	-33	5.98E-7	4.21E-6	> 1.00E-4
K-562	0.255	2.249	2.115	1.902	0.875	0.411	0.126	93	83	31	8	-51	4.29E-7	1.36E-5	9.70E-5
MOLT-4	0.491	2.462	2.510	2.337	1.644	0.718	0.275	102	94	59	11	-44	1.52E-6	1.61E-5	> 1.00E-4
RPMI-8226	0.768	2.466	2.399	2.398	1.766	0.807	0.773	96	96	59	2	0	1.43E-6	> 1.00E-4	> 1.00E-4
Non-Small Cell Lung Cancer															
A549/ATCC	0.294	2.261	2.025	2.228	1.451	0.614	0.072	88	98	59	16	-76	1.61E-6	1.50E-5	5.26E-5
EKVX	0.960	2.556	2.402	2.489	2.367	1.423	0.017	90	96	88	29	-98	4.41E-6	1.69E-5	4.17E-5
HOP-62	0.633	2.490	2.327	2.387	1.694	1.258	0.174	91	94	57	34	-73	2.01E-6	2.07E-5	6.14E-5
HOP-92	1.139	1.844	1.739	1.656	1.569	1.284	0.228	85	73	61	20	-80	1.87E-6	1.60E-5	5.03E-5
NCI-H226	1.037	1.914	1.804	1.858	1.726	1.101	0.276	88	94	79	7	-73	2.52E-6	1.23E-5	5.12E-5
NCI-H23	0.591	1.937	1.838	1.876	1.517	0.590	0.035	93	95	69	0	-94	1.87E-6	9.92E-6	3.39E-5
NCI-H322M	0.811	2.219	1.999	2.008	1.803	1.039	0.003	84	85	70	16	-100	2.38E-6	1.38E-5	3.72E-5
NCI-H460	0.259	2.709	2.690	2.706	1.211	0.262	0.064	99	100	39	0	-75	6.57E-7	1.00E-5	4.60E-5
NCI-H522	1.295	3.048	3.009	2.924	2.145	0.924	0.198	98	93	48	-29	-85	9.25E-7	4.25E-6	2.40E-5
Colon Cancer															
COLO 205	0.551	2.564	2.582	2.500	1.726	0.369	0.068	101	97	58	-33	-88	1.23E-6	4.35E-6	2.04E-5
HCC-2998	0.668	2.576	2.367	2.539	1.844	0.677	0.016	89	98	62	0	-98	1.55E-6	1.01E-5	3.27E-5
HCT-116	0.301	2.705	2.879	2.991	1.498	0.465	0.219	107	112	50	7	-27	9.91E-7	1.59E-5	> 1.00E-4
HCT-15	0.311	2.525	2.330	2.416	1.380	0.385	0.009	91	95	48	3	-97	9.18E-7	1.08E-5	3.40E-5
HT29	0.217	1.692	1.590	1.732	0.798	0.194	0.035	93	103	39	-11	-84	6.80E-7	6.14E-6	3.45E-5
KM12	0.494	2.606	2.527	2.617	1.548	0.570	0.054	96	101	50	4	-89	9.96E-7	1.09E-5	3.78E-5
SW-620	0.268	2.095	2.036	2.008	0.890	0.606	0.053	97	95	34	19	-80	5.48E-7	1.54E-5	4.94E-5
CNS Cancer															
SF-268	1.072	2.934	2.776	2.750	2.458	1.266	0.344	92	90	74	10	-68	2.41E-6	1.36E-5	5.91E-5
SF-295	1.309	3.247	3.056	3.110	2.630	1.412	0.008	90	93	68	5	-99	1.94E-6	1.12E-5	3.38E-5
SF-539	0.818	2.463	2.367	2.400	1.591	0.530	0.005	94	96	47	-35	-99	8.69E-7	3.73E-6	1.70E-5
SNB-19	0.564	2.009	1.931	1.944	1.546	0.908	0.004	95	96	68	24	-99	2.55E-6	1.56E-5	3.98E-5
SNB-75	1.773	2.796	2.445	2.631	2.264	1.648	0.142	66	84	48	-7	-92	8.80E-7	7.45E-6	3.20E-5
U251	0.353	1.923	1.854	1.820	1.386	0.608	0.028	96	93	66	16	-92	2.08E-6	1.41E-5	4.08E-5
Melanoma															
LOX IMVI	0.406	2.908	2.748	2.783	1.612	0.793	0.017	94	95	48	15	-96	9.16E-7	1.38E-5	3.87E-5
MALME-3M	0.616	1.675	1.536	1.596	1.286	1.025	0.056	87	93	63	39	-91	3.46E-6	1.99E-5	4.83E-5
M14	0.601	2.419	2.467	2.615	1.649	0.947	0.088	103	111	58	19	-85	1.58E-6	1.52E-5	4.58E-5
MDA-MB-435	0.676	3.019	2.946	3.007	1.265	0.414	0.034	97	99	25	-39	-95	4.63E-7	2.47E-6	1.58E-5
SK-MEL-2	0.850	1.947	1.929	1.967	1.725	0.900	0.096	98	102	80	5	-89	2.48E-6	1.12E-5	3.84E-5
SK-MEL-28	0.751	2.026	1.992	2.058	1.761	1.271	0.005	97	102	79	41	-99	5.75E-6	1.95E-5	4.44E-5
SK-MEL-5	0.904	3.255	2.965	3.078	2.170	0.540	0.004	88	92	54	-40	-100	1.10E-6	3.73E-6	1.46E-5
UACC-257	0.919	2.466	2.329	2.368	2.001	1.664	0.034	91	94	70	48	-96	8.22E-6	2.15E-5	4.78E-5
UACC-62	0.974	2.837	2.679	2.678	1.910	1.517	0.006	92	91	50	29	-99	1.03E-6	1.68E-5	4.13E-5
Ovarian Cancer															
IGROV1	0.436	2.066	1.953	1.877	1.322	0.636	0.065	93	88	54	12	-85	1.27E-6	1.34E-5	4.36E-5
OVCAR-3	0.626	2.045	2.033	2.017	1.365	0.613	0.018	99	98	52	-2	-97	1.09E-6	9.12E-6	3.19E-5
OVCAR-4	0.715	1.957	1.866	1.950	1.727	1.154	0.036	93	99	82	35	-95	4.82E-6	1.87E-5	4.52E-5
OVCAR-5	0.447	1.562	1.559	1.657	1.126	0.623	0.015	100	109	61	16	-97	1.74E-6	1.38E-5	3.85E-5
OVCAR-8	0.337	2.001	1.759	1.998	1.254	0.312	0.068	85	100	55	-7	-80	1.21E-6	7.61E-6	3.86E-5
NCI/ADR-RES	0.357	1.372	1.295	1.373	0.741	0.219	0.126	92	100	38	-39	-65	6.36E-7	3.11E-6	2.71E-5
SK-OV-3	0.838	2.026	1.759	1.918	1.819	1.301	0.294	78	91	83	39	-65	5.59E-6	2.37E-5	7.19E-5
Renal Cancer															
786-0	0.777	2.913	2.815	2.919	2.622	1.568	0.303	95	100	86	37	-61	5.46E-6	2.39E-5	7.72E-5
A498	1.209	2.189	2.104	2.199	2.049	1.858	0.104	91	101	86	66	-91	1.27E-5	2.63E-5	5.46E-5
ACHN	0.374	1.724	1.649	1.724	1.168	0.657	0.003	94	100	59	21	-99	1.71E-6	1.49E-5	3.90E-5
CAKI-1	0.495	1.853	1.727	1.740	1.104	0.761	0.058	91	92	45	20	-88	7.76E-7	1.52E-5	4.41E-5
RXF 393	0.750	1.549	1.497	1.512	1.238	0.887	0.125	93	95	61	17	-83	1.78E-6	1.48E-5	4.65E-5
SN12C	0.631	2.236	2.107	2.136	1.579	0.743	0.051	92	94	59	7	-92	1.49E-6	1.18E-5	3.77E-5
TK-10	1.084	2.126	2.003	2.068	1.987	1.669	0.186	88	94	87	56	-83	1.11E-5	2.53E-5	5.80E-5
UO-31	0.664	2.093	1.828	1.842	1.504	0.936	0.043	81	82	59	19	-94	1.66E-6	1.47E-5	4.10E-5
Prostate Cancer															
PC-3	0.562	2.300	2.178	2.295	1.707	0.793	0.405	93	100	66	13	-28	2.00E-6	2.10E-5	> 1.00E-4
DU-145	0.375	1.742	1.719	1.765	1.318	0.425	0.016	98	102	69	4	-96	1.95E-6	1.09E-5	3.47E-5
Breast Cancer															
MCF7	0.450	2.371	2.187	2.156	0.990	0.542	0.118	90	89	28	5	-74	4.36E-7	1.15E-5	4.98E-5
MDA-MB-231/ATCC	0.566	1.153	1.128	1.170	0.965	0.466	0.032	96	103	68	-18	-94	1.62E-6	6.22E-6	2.64E-5
HS 578T	1.374	2.554	2.366	2.400	2.029	1.473	0.798	84	87	56	8	-42	1.31E-6	1.47E-5	> 1.00E-4
BT-549	1.398	2.755	3.081	2.905	2.737	1.730	0.291	124	111	99	24	-79	4.53E-6	1.72E-5	5.23E-5
T-47D	0.747	2.102	1.874	1.847	1.605	1.274	0.393	83	81	63	39	-47	3.49E-6	2.82E-5	> 1.00E-4
MDA-MB-468	0.773	1.624	1.558	1.577	1.088	0.590	0.062	92	94	37	-24	-92	5.93E-7	4.06E-6	2.42E-5

Compound **3c**

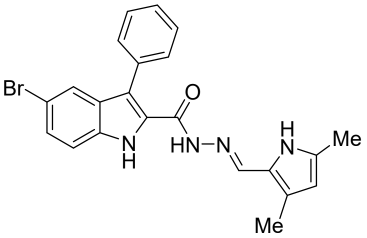

Br
O
HN−N
H
N
Me
N
H
Me

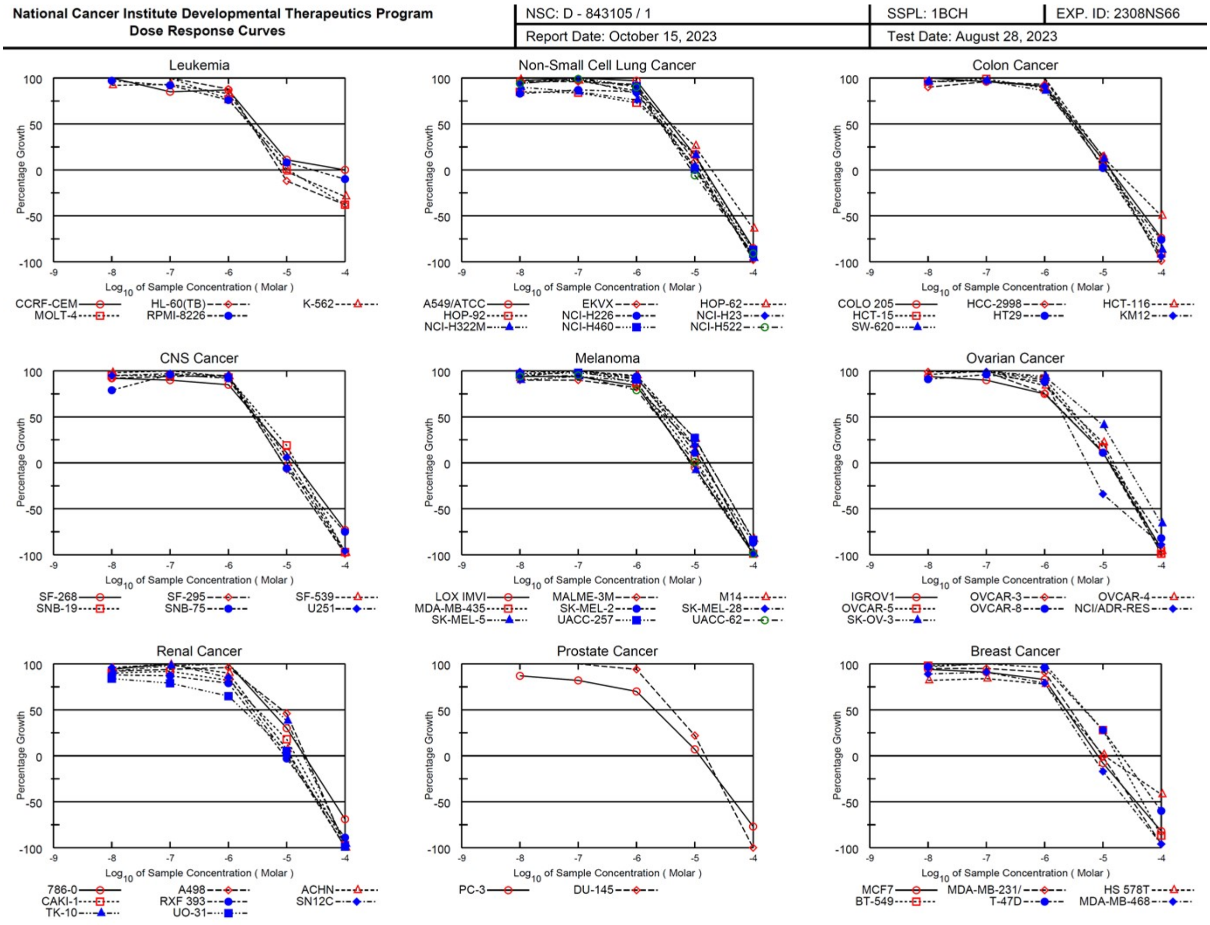

National Cancer Institute Developmental Therapeutics Program
Dose Response Curves
NSC: D - 843105 / 1
Report Date: October 15, 2023
SSPL: 1BCH
EXP. ID: 2308NS66
Test Date: August 28, 2023
Leukemia
Non-Small Cell Lung Cancer
Colon Cancer
CNS Cancer
Melanoma
Ovarian Cancer
Renal Cancer
Prostate Cancer
Breast Cancer
Percentage Growth
Log10 of Sample Concentration (Molar)
CCRF-CEM
HL-60(TB)
K-562
MOLT-4
RPMI-8226
A549/ATCC
EKVX
HOP-62
HOP-92
NCI-H226
NCI-H23
NCI-H322M
NCI-H460
NCI-H522
COLO 205
HCC-2998
HCT-116
HCT-15
HT29
KM12
SW-620
SF-268
SF-295
SF-539
SNB-19
SNB-75
U251
LOX IMVI
MALME-3M
M14
MDA-MB-435
SK-MEL-2
SK-MEL-28
SK-MEL-5
UACC-257
UACC-62
IGROV1
OVCAR-3
OVCAR-4
OVCAR-5
OVCAR-8
NCI/ADR-RES
SK-OV-3
786-0
A498
ACHN
CAKI-1
RXF 393
SN12C
TK-10
UO-31
PC-3
DU-145
MCF7
MDA-MB-231/
HS 578T
BT-549
T-47D
MDA-MB-468

National Cancer Institute Developmental Therapeutics Program
In-Vitro Testing Results

NSC : D - 843105 / 1	Experiment ID : 2308NS66	Test Type : 08	Units : Molar
Report Date : October 15, 2023	Test Date : August 28, 2023	QNS :	MC :
COMI : To43	Stain Reagent : SRB Dual-Pass Related	SSPL : 1BCH	

			Log10 Concentration												
	Time		Mean Optical Densities					Percent Growth							
Panel/Cell Line	Zero	Ctrl	-8.0	-7.0	-6.0	-5.0	-4.0	-8.0	-7.0	-6.0	-5.0	-4.0	GI50	TGI	LC50
**Leukemia**															
CCRF-CEM	0.489	2.664	2.700	2.345	2.371	0.739	0.496	102	85	87	11	0	3.07E-6	> 1.00E-4	> 1.00E-4
HL-60(TB)	0.609	2.344	2.337	2.363	2.135	0.538	0.375	100	101	88	-12	-38	2.40E-6	7.64E-6	> 1.00E-4
K-562	0.255	2.045	1.904	1.928	1.733	0.250	0.182	92	93	83	-2	-29	2.43E-6	9.48E-6	> 1.00E-4
MOLT-4	0.491	2.118	2.223	2.224	1.750	0.511	0.304	106	107	77	1	-38	2.29E-6	1.07E-5	> 1.00E-4
RPMI-8226	0.768	2.610	2.550	2.456	2.172	0.922	0.693	97	92	76	8	-10	2.43E-6	2.89E-5	> 1.00E-4
**Non-Small Cell Lung Cancer**															
A549/ATCC	0.294	1.931	1.876	2.007	1.883	0.561	0.046	97	105	97	16	-85	3.82E-6	1.45E-5	4.54E-5
EKVX	0.960	2.552	2.490	2.493	2.442	1.098	0.018	96	96	93	9	-98	3.24E-6	1.21E-5	3.54E-5
HOP-62	0.633	2.170	2.141	2.131	1.955	1.031	0.226	98	97	86	26	-64	3.97E-6	1.94E-5	6.93E-5
HOP-92	1.139	1.692	1.612	1.604	1.545	1.234	0.137	85	84	73	17	-88	2.61E-6	1.46E-5	4.35E-5
NCI-H226	1.037	1.779	1.653	1.686	1.670	1.031	0.076	83	87	85	0	-93	2.58E-6	9.83E-6	3.44E-5
NCI-H23	0.591	1.941	1.854	1.933	1.706	0.641	0.033	94	99	83	4	-95	2.59E-6	1.09E-5	3.52E-5
NCI-H322M	0.811	2.367	2.212	2.131	1.991	1.055	0.032	90	85	76	16	-96	2.69E-6	1.38E-5	3.87E-5
NCI-H460	0.259	2.523	2.528	2.600	2.313	0.272	0.034	100	103	91	1	-87	2.83E-6	1.01E-5	3.79E-5
NCI-H522	1.295	3.028	2.930	3.014	2.854	1.217	0.111	94	99	90	-6	-91	2.61E-6	8.65E-6	3.27E-5
**Colon Cancer**															
COLO 205	0.551	2.505	2.507	2.422	2.327	0.765	0.144	100	96	91	11	-74	3.25E-6	1.35E-5	5.23E-5
HCC-2998	0.668	2.575	2.388	2.489	2.445	0.948	0.008	90	96	93	15	-99	3.55E-6	1.35E-5	3.72E-5
HCT-116	0.301	2.498	2.764	2.734	2.594	0.616	0.151	112	111	104	14	-50	4.02E-6	1.67E-5	> 1.00E-4
HCT-15	0.311	2.500	2.413	2.484	2.257	0.423	0.027	96	99	89	5	-91	2.91E-6	1.13E-5	3.72E-5
HT29	0.217	1.583	1.748	1.620	1.700	0.240	0.053	112	103	109	2	-76	3.53E-6	1.05E-5	4.66E-5
KM12	0.494	2.409	2.338	2.348	2.238	0.569	0.029	96	97	91	4	-94	2.96E-6	1.10E-5	3.54E-5
SW-620	0.268	1.932	1.873	1.883	1.703	0.462	0.034	96	97	86	12	-87	3.06E-6	1.31E-5	4.20E-5
**CNS Cancer**															
SF-268	1.072	2.816	2.673	2.635	2.555	1.265	0.289	92	90	85	11	-73	2.98E-6	1.35E-5	5.32E-5
SF-295	1.309	3.287	3.127	3.163	3.182	1.222	0.015	92	94	95	-7	-99	2.76E-6	8.60E-6	2.95E-5
SF-539	0.818	2.411	2.380	2.437	2.312	0.819	0.016	98	102	94	0	-98	2.93E-6	1.00E-5	3.24E-5
SNB-19	0.564	1.977	1.904	1.904	1.882	0.839	0.016	95	95	93	19	-97	3.86E-6	1.47E-5	3.94E-5
SNB-75	1.773	2.753	2.551	2.716	2.671	1.674	0.449	79	96	92	-6	-75	2.68E-6	8.75E-6	4.39E-5
U251	0.353	1.823	1.746	1.776	1.747	0.448	0.015	95	97	95	6	-96	3.21E-6	1.16E-5	3.57E-5
**Melanoma**															
LOX IMVI	0.406	2.825	2.672	2.682	2.447	0.386	0.005	94	94	84	-5	-99	2.43E-6	8.81E-6	3.02E-5
MALME-3M	0.616	1.715	1.607	1.602	1.519	0.811	0.013	90	90	82	18	-98	3.16E-6	1.42E-5	3.85E-5
M14	0.601	2.421	2.307	2.453	2.325	1.070	0.104	94	102	95	26	-83	4.45E-6	1.73E-5	4.99E-5
MDA-MB-435	0.676	2.923	2.846	2.929	2.634	0.793	0.009	97	100	87	5	-99	2.84E-6	1.12E-5	3.40E-5
SK-MEL-2	0.850	1.956	1.975	1.984	1.888	0.968	0.110	102	103	94	11	-87	3.37E-6	1.28E-5	4.17E-5
SK-MEL-28	0.751	2.060	2.043	2.162	1.960	1.006	0.012	99	108	92	19	-98	3.81E-6	1.46E-5	3.88E-5
SK-MEL-5	0.904	3.243	3.001	3.102	2.984	0.833	0.005	90	94	89	-8	-99	2.52E-6	8.30E-6	2.88E-5
UACC-257	0.919	2.386	2.330	2.358	2.249	1.315	0.145	96	98	91	27	-84	4.35E-6	1.75E-5	4.92E-5
UACC-62	0.974	2.843	2.724	2.748	2.452	0.994	0.011	94	95	79	1	-99	2.36E-6	1.02E-5	3.24E-5
**Ovarian Cancer**															
IGROV1	0.436	2.136	2.026	1.958	1.719	0.618	0.030	94	90	75	11	-93	2.47E-6	1.27E-5	3.84E-5
OVCAR-3	0.626	1.961	1.944	1.951	1.642	0.778	0.013	99	99	76	11	-98	2.53E-6	1.27E-5	3.64E-5
OVCAR-4	0.715	1.862	1.812	1.887	1.684	0.966	0.029	96	102	84	22	-96	3.55E-6	1.53E-5	4.07E-5
OVCAR-5	0.447	1.546	1.605	1.628	1.463	0.630	0.004	105	107	92	17	-99	3.63E-6	1.39E-5	3.76E-5
OVCAR-8	0.337	1.989	1.837	1.928	1.783	0.522	0.059	91	96	88	11	-82	3.10E-6	1.32E-5	4.50E-5
NCI/ADR-RES	0.357	1.306	1.304	1.301	1.214	0.235	0.039	100	99	90	-34	-89	2.11E-6	5.30E-6	1.93E-5
SK-OV-3	0.838	1.821	1.859	1.843	1.763	1.243	0.284	104	102	94	41	-66	6.81E-6	2.42E-5	7.08E-5
**Renal Cancer**															
786-0	0.777	2.726	2.615	2.740	2.727	1.363	0.238	94	101	100	30	-69	5.19E-6	2.01E-5	6.39E-5
A498	1.209	2.109	2.039	2.057	2.071	1.622	0.037	92	94	96	46	-97	8.25E-6	2.09E-5	4.69E-5
ACHN	0.374	1.749	1.677	1.740	1.608	0.473	.	95	99	90	7	-100	3.03E-6	1.17E-5	3.42E-5
CAKI-1	0.495	1.741	1.620	1.647	1.521	0.717	0.017	90	92	82	18	-97	3.17E-6	1.43E-5	3.91E-5
RXF 393	0.750	1.433	1.351	1.343	1.290	0.728	0.080	88	87	79	-3	-89	2.26E-6	9.21E-6	3.50E-5
SN12C	0.631	2.180	2.124	2.228	1.940	0.642	0.007	96	103	85	1	-99	2.58E-6	1.02E-5	3.23E-5
TK-10	1.084	2.177	2.093	2.159	2.484	1.497	0.042	92	98	128	38	-96	7.32E-6	1.91E-5	4.52E-5
UO-31	0.664	2.093	1.863	1.788	1.590	0.739	0.006	84	79	65	5	-99	1.77E-6	1.12E-5	3.38E-5
**Prostate Cancer**															
PC-3	0.562	2.463	2.220	2.129	1.889	0.691	0.129	87	82	70	7	-77	2.06E-6	1.20E-5	4.76E-5
DU-145	0.375	1.592	1.644	1.647	1.520	0.641	0.001	104	104	94	22	-100	4.07E-6	1.51E-5	3.89E-5
**Breast Cancer**															
MCF7	0.450	2.370	2.248	2.190	2.041	0.415	0.082	94	91	83	-8	-82	2.30E-6	8.21E-6	3.72E-5
MDA-MB-231/ATCC	0.566	1.122	1.094	1.096	1.071	0.559	0.025	95	95	91	-1	-96	2.78E-6	9.67E-6	3.28E-5
HS 578T	1.374	2.554	2.342	2.362	2.294	1.387	0.791	82	84	78	1	-42	2.31E-6	1.06E-5	> 1.00E-4
BT-549	1.398	2.712	2.691	2.714	2.743	1.764	0.189	98	100	102	28	-87	5.04E-6	1.75E-5	4.79E-5
T-47D	0.747	1.937	1.897	1.950	1.887	1.075	0.302	97	101	96	28	-60	4.68E-6	2.07E-5	7.76E-5
MDA-MB-468	0.773	1.477	1.397	1.413	1.326	0.645	0.031	89	91	79	-17	-96	2.00E-6	6.70E-6	2.64E-5

Compound **3d**

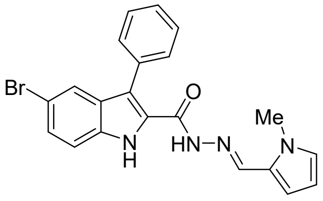
Br
O
Me
N
HN–N
N
H

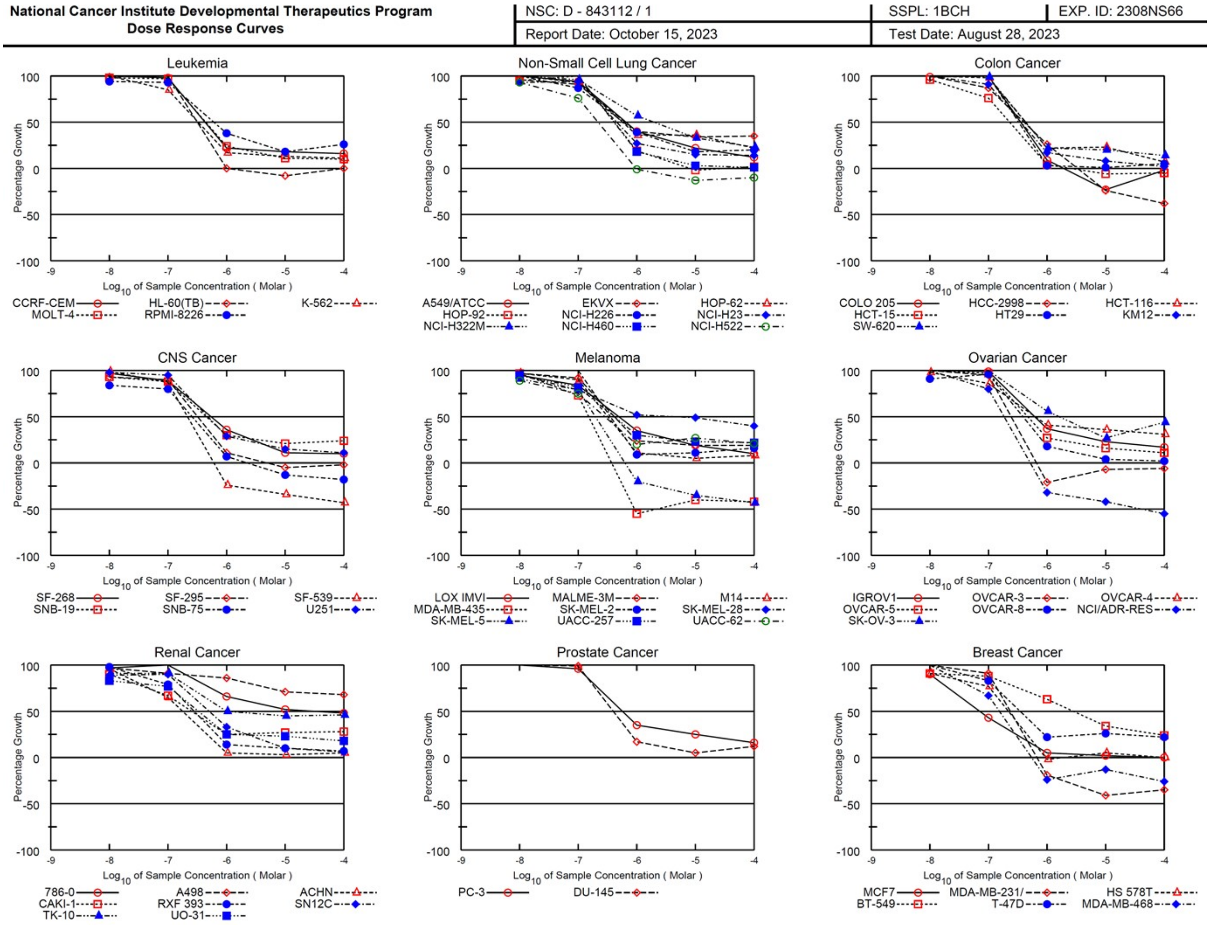
National Cancer Institute Developmental Therapeutics Program
Dose Response Curves
NSC: D - 843112 / 1
Report Date: October 15, 2023
SSPL: 1BCH
EXP. ID: 2308NS66
Test Date: August 28, 2023
Leukemia
Non-Small Cell Lung Cancer
Colon Cancer
CNS Cancer
Melanoma
Ovarian Cancer
Renal Cancer
Prostate Cancer
Breast Cancer
Percentage Growth
Log10 of Sample Concentration (Molar)
CCRF-CEM
HL-60(TB)
K-562
MOLT-4
RPMI-8226
A549/ATCC
EKVX
HOP-62
HOP-92
NCI-H226
NCI-H23
NCI-H322M
NCI-H460
NCI-H522
COLO 205
HCC-2998
HCT-116
HCT-15
HT29
KM12
SW-620
SF-268
SF-295
SF-539
SNB-19
SNB-75
U251
LOX IMVI
MALME-3M
M14
MDA-MB-435
SK-MEL-2
SK-MEL-28
SK-MEL-5
UACC-257
UACC-62
IGROV1
OVCAR-3
OVCAR-4
OVCAR-5
OVCAR-8
NCI/ADR-RES
SK-OV-3
786-0
A498
ACHN
CAKI-1
RXF 393
SN12C
TK-10
UO-31
PC-3
DU-145
MCF7
MDA-MB-231/
HS 578T
BT-549
T-47D
MDA-MB-468

National Cancer Institute Developmental Therapeutics Program
In-Vitro Testing Results

NSC : D - 843112 / 1	Experiment ID : 2308NS66	Test Type : 08	Units : Molar
Report Date : October 15, 2023	Test Date : August 28, 2023	QNS :	MC :
COMI : To50	Stain Reagent : SRB Dual-Pass Related	SSPL : 1BCH	

			Log10 Concentration													
	Time		Mean Optical Densities					Percent Growth								
Panel/Cell Line	Zero	Ctrl	-8.0	-7.0	-6.0	-5.0	-4.0	-8.0	-7.0	-6.0	-5.0	-4.0	GI50	TGI	LC50	
**Leukemia**																
CCRF-CEM	0.489	2.664	2.846	2.624	0.969	0.887	0.831	108	98	22	18	16	4.29E-7	> 1.00E-4	> 1.00E-4	
HL-60(TB)	0.609	2.344	2.320	2.341	0.615	0.562	0.609	99	100	0	-8	0	3.17E-7	1.00E-4	> 1.00E-4	
K-562	0.255	2.045	2.063	1.786	0.552	0.487	0.455	101	85	17	13	11	3.27E-7	> 1.00E-4	> 1.00E-4	
MOLT-4	0.491	2.118	2.092	2.068	0.877	0.677	0.653	98	97	24	11	10	4.38E-7	> 1.00E-4	> 1.00E-4	
RPMI-8226	0.768	2.610	2.504	2.474	1.474	1.095	1.251	94	93	38	18	26	6.10E-7	> 1.00E-4	> 1.00E-4	
**Non-Small Cell Lung Cancer**																
A549/ATCC	0.294	1.931	2.029	1.839	0.945	0.648	0.484	106	94	40	22	12	6.49E-7	> 1.00E-4	> 1.00E-4	
EKVX	0.960	2.552	2.487	2.406	1.601	1.498	1.514	96	91	40	34	35	6.41E-7	> 1.00E-4	> 1.00E-4	
HOP-62	0.633	2.170	2.262	2.062	1.188	1.182	0.974	106	93	36	36	22	5.70E-7	> 1.00E-4	> 1.00E-4	
HOP-92	1.139	1.692	1.665	1.711	1.244	1.122	1.149	95	103	19	-2	2	4.29E-7	.	> 1.00E-4	
NCI-H226	1.037	1.779	1.777	1.686	1.330	1.174	1.183	100	87	39	18	20	6.03E-7	> 1.00E-4	> 1.00E-4	
NCI-H23	0.591	1.941	1.847	1.854	0.951	0.798	0.783	93	94	27	15	14	4.47E-7	> 1.00E-4	> 1.00E-4	
NCI-H322M	0.811	2.367	2.414	2.300	1.700	1.326	1.172	103	96	57	33	23	1.98E-6	> 1.00E-4	> 1.00E-4	
NCI-H460	0.259	2.523	2.661	2.712	0.666	0.337	0.292	106	108	18	3	1	4.42E-7	> 1.00E-4	> 1.00E-4	
NCI-H522	1.295	3.028	2.904	2.612	1.280	1.126	1.171	93	76	-1	-13	-10	2.17E-7	9.66E-7	> 1.00E-4	
**Colon Cancer**																
COLO 205	0.551	2.505	2.490	2.553	0.733	0.426	0.542	99	102	9	-23	-2	3.66E-7	1.95E-6	> 1.00E-4	
HCC-2998	0.668	2.575	2.629	2.324	1.161	0.506	0.414	103	87	26	-24	-38	4.02E-7	3.28E-6	> 1.00E-4	
HCT-116	0.301	2.498	2.503	2.453	0.782	0.816	0.453	100	98	22	23	7	4.27E-7	> 1.00E-4	> 1.00E-4	
HCT-15	0.311	2.500	2.404	1.971	0.391	0.294	0.295	96	76	4	-6	-5	2.28E-7	2.47E-6	> 1.00E-4	
HT29	0.217	1.583	1.616	1.578	0.262	0.235	0.288	102	100	3	1	5	3.27E-7	> 1.00E-4	> 1.00E-4	
KM12	0.494	2.409	2.415	2.238	0.817	0.651	0.540	100	91	17	8	2	3.57E-7	> 1.00E-4	> 1.00E-4	
SW-620	0.268	1.932	2.009	1.922	0.630	0.607	0.507	105	99	22	20	14	4.32E-7	> 1.00E-4	> 1.00E-4	
**CNS Cancer**																
SF-268	1.072	2.816	2.770	2.623	1.703	1.263	1.247	97	89	36	11	10	5.47E-7	> 1.00E-4	> 1.00E-4	
SF-295	1.309	3.287	3.152	3.086	1.531	1.249	1.281	93	90	11	-5	-2	3.21E-7	5.11E-6	> 1.00E-4	
SF-539	0.818	2.411	2.398	2.211	0.621	0.538	0.464	99	87	-24	-34	-43	2.17E-7	6.08E-7	> 1.00E-4	
SNB-19	0.564	1.977	1.879	1.809	0.983	0.867	0.897	93	88	30	21	24	4.49E-7	> 1.00E-4	> 1.00E-4	
SNB-75	1.773	2.753	2.599	2.558	1.847	1.549	1.451	84	80	7	-13	-18	2.59E-7	2.35E-6	> 1.00E-4	
U251	0.353	1.823	1.787	1.750	0.775	0.579	0.521	98	95	29	15	11	4.77E-7	> 1.00E-4	> 1.00E-4	
**Melanoma**																
LOX IMVI	0.406	2.825	2.710	2.446	1.249	0.859	0.655	95	84	35	19	10	4.94E-7	> 1.00E-4	> 1.00E-4	
MALME-3M	0.616	1.715	1.679	1.623	0.875	0.823	0.830	97	92	24	19	19	4.09E-7	> 1.00E-4	> 1.00E-4	
M14	0.601	2.421	2.375	2.248	0.807	0.691	0.745	97	90	11	5	8	3.24E-7	> 1.00E-4	> 1.00E-4	
MDA-MB-435	0.676	2.923	2.859	2.311	0.302	0.403	0.389	97	73	-55	-40	-42	1.51E-7	3.70E-7	.	
SK-MEL-2	0.850	1.956	1.986	1.982	0.955	0.969	1.027	103	102	9	11	16	3.66E-7	> 1.00E-4	> 1.00E-4	
SK-MEL-28	0.751	2.060	1.990	1.784	1.433	1.393	1.272	95	79	52	49	40	4.70E-6	> 1.00E-4	> 1.00E-4	
SK-MEL-5	0.904	3.243	3.033	2.744	0.727	0.587	0.511	91	79	-20	-35	-43	1.96E-7	6.32E-7	> 1.00E-4	
UACC-257	0.919	2.386	2.307	2.127	1.360	1.264	1.243	95	82	30	23	22	4.16E-7	> 1.00E-4	> 1.00E-4	
UACC-62	0.974	2.843	2.645	2.371	1.346	1.484	1.375	89	75	20	27	21	2.83E-7	> 1.00E-4	> 1.00E-4	
**Ovarian Cancer**																
IGROV1	0.436	2.136	2.177	2.122	1.068	0.819	0.721	102	99	37	23	17	6.21E-7	> 1.00E-4	> 1.00E-4	
OVCAR-3	0.626	1.961	2.142	1.898	0.496	0.581	0.586	114	95	-21	-7	-6	2.46E-7	6.62E-7	> 1.00E-4	
OVCAR-4	0.715	1.862	1.834	1.701	1.189	1.131	1.071	98	86	41	36	31	6.37E-7	> 1.00E-4	> 1.00E-4	
OVCAR-5	0.447	1.546	1.544	1.518	0.744	0.624	0.573	100	97	27	16	11	4.72E-7	> 1.00E-4	> 1.00E-4	
OVCAR-8	0.337	1.989	1.834	1.929	0.634	0.405	0.371	91	96	18	4	2	3.90E-7	> 1.00E-4	> 1.00E-4	
NCI/ADR-RES	0.357	1.306	1.309	1.120	0.242	0.209	0.160	100	80	-32	-42	-55	1.86E-7	5.17E-7	4.09E-5	
SK-OV-3	0.838	1.821	1.851	1.860	1.390	1.100	1.268	103	104	56	27	44	1.61E-6	> 1.00E-4	> 1.00E-4	
**Renal Cancer**																
786-0	0.777	2.726	2.666	2.756	2.055	1.791	1.707	97	102	66	52	48	2.94E-5	> 1.00E-4	> 1.00E-4	
A498	1.209	2.109	2.087	2.028	1.986	1.847	1.824	97	91	86	71	68	> 1.00E-4	> 1.00E-4	> 1.00E-4	
ACHN	0.374	1.749	1.691	1.262	0.437	0.415	0.442	96	65	5	3	5	1.75E-7	> 1.00E-4	> 1.00E-4	
CAKI-1	0.495	1.741	1.620	1.327	0.812	0.834	0.839	90	67	25	27	28	2.55E-7	> 1.00E-4	> 1.00E-4	
RXF 393	0.750	1.433	1.417	1.292	0.843	0.816	0.799	98	79	14	10	7	2.80E-7	> 1.00E-4	> 1.00E-4	
SN12C	0.631	2.180	2.011	2.017	1.138	0.785	0.720	89	90	33	10	6	4.97E-7	> 1.00E-4	> 1.00E-4	
TK-10	1.084	2.177	2.081	2.082	1.631	1.575	1.588	91	91	50	45	46	1.01E-6	> 1.00E-4	> 1.00E-4	
UO-31	0.664	2.093	1.852	1.770	1.016	0.987	0.923	83	77	25	23	18	3.30E-7	> 1.00E-4	> 1.00E-4	
**Prostate Cancer**																
PC-3	0.562	2.463	2.463	2.388	1.222	1.037	0.859	100	96	35	25	16	5.64E-7	> 1.00E-4	> 1.00E-4	
DU-145	0.375	1.592	1.663	1.583	0.586	0.440	0.516	106	99	17	5	12	3.99E-7	> 1.00E-4	> 1.00E-4	
**Breast Cancer**																
MCF7	0.450	2.370	2.177	1.283	0.537	0.492	0.447	90	43	5	2	0	7.21E-8	5.84E-5	> 1.00E-4	
MDA-MB-231/ATCC	0.566	1.122	1.124	1.075	0.457	0.336	0.366	100	91	-19	-41	-35	2.37E-7	6.70E-7	> 1.00E-4	
HS 578T	1.374	2.554	2.442	2.282	1.352	1.428	1.364	91	77	-2	5	0	2.20E-7	.	> 1.00E-4	
BT-549	1.398	2.712	2.598	2.550	2.222	1.851	1.717	91	88	63	34	24	2.81E-6	> 1.00E-4	> 1.00E-4	
T-47D	0.747	1.937	2.042	1.735	1.004	1.057	1.009	109	83	22	26	22	3.44E-7	> 1.00E-4	> 1.00E-4	
MDA-MB-468	0.773	1.477	1.475	1.248	0.589	0.671	0.571	100	67	-24	-13	-26	1.55E-7	5.48E-7	> 1.00E-4	

Compound **3e**

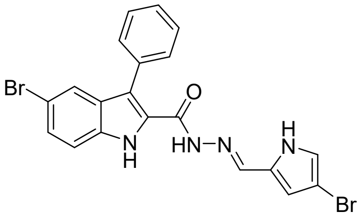

Br
O
HN–N
H
N
Br

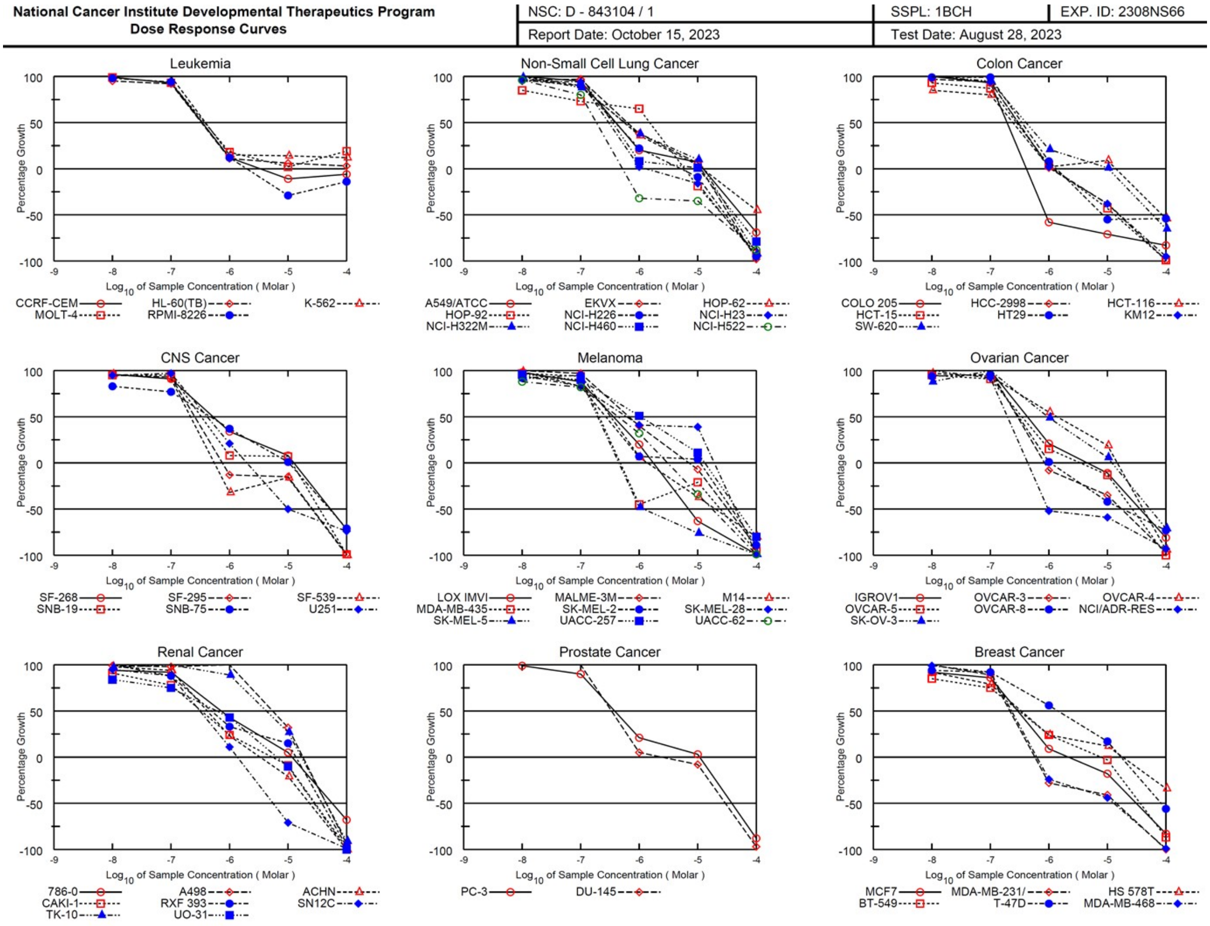

National Cancer Institute Developmental Therapeutics Program
Dose Response Curves
NSC: D - 843104 / 1
Report Date: October 15, 2023
SSPL: 1BCH
EXP. ID: 2308NS66
Test Date: August 28, 2023
Leukemia
Non-Small Cell Lung Cancer
Colon Cancer
CNS Cancer
Melanoma
Ovarian Cancer
Renal Cancer
Prostate Cancer
Breast Cancer
Percentage Growth
Log10 of Sample Concentration (Molar)
CCRF-CEM
HL-60(TB)
K-562
MOLT-4
RPMI-8226
A549/ATCC
EKVX
HOP-62
HOP-92
NCI-H226
NCI-H23
NCI-H322M
NCI-H460
NCI-H522
COLO 205
HCC-2998
HCT-116
HCT-15
HT29
KM12
SW-620
SF-268
SF-295
SF-539
SNB-19
SNB-75
U251
LOX IMVI
MALME-3M
M14
MDA-MB-435
SK-MEL-2
SK-MEL-28
SK-MEL-5
UACC-257
UACC-62
IGROV1
OVCAR-3
OVCAR-4
OVCAR-5
OVCAR-8
NCI/ADR-RES
SK-OV-3
786-0
A498
ACHN
CAKI-1
RXF 393
SN12C
TK-10
UO-31
PC-3
DU-145
MCF7
MDA-MB-231/
HS 578T
BT-549
T-47D
MDA-MB-468

National Cancer Institute Developmental Therapeutics Program
In-Vitro Testing Results

NSC : D - 843104 / 1	Experiment ID : 2308NS66	Test Type : 08	Units : Molar
Report Date : October 15, 2023	Test Date : August 28, 2023	QNS :	MC :
COMI : TO42	Stain Reagent : SRB Dual-Pass Related	SSPL : 1BCH	

Panel/Cell Line	Time Zero	Ctrl	Mean Optical Densities (Log10 Concentration) -8.0	-7.0	-6.0	-5.0	-4.0	Percent Growth (Log10 Concentration) -8.0	-7.0	-6.0	-5.0	-4.0	GI50	TGI	LC50
**Leukemia**															
CCRF-CEM	0.489	2.112	2.093	2.004	0.684	0.434	0.460	99	93	12	-11	-6	3.41E-7	3.28E-6	> 1.00E-4
HL-60(TB)	0.609	2.572	2.467	2.408	0.819	0.722	0.661	95	92	11	6	3	3.27E-7	> 1.00E-4	> 1.00E-4
K-562	0.255	2.236	2.234	2.246	0.562	0.526	0.500	100	101	15	14	12	3.93E-7	> 1.00E-4	> 1.00E-4
MOLT-4	0.491	2.517	2.498	2.379	0.854	0.528	0.876	99	93	18	2	19	3.75E-7	> 1.00E-4	> 1.00E-4
RPMI-8226	0.768	2.366	2.339	2.278	0.959	0.549	0.658	98	94	12	-29	-14	3.46E-7	1.97E-6	> 1.00E-4
**Non-Small Cell Lung Cancer**															
A549/ATCC	0.294	2.072	2.079	1.983	0.642	0.419	0.092	100	95	20	7	-69	3.95E-7	1.24E-5	5.64E-5
EKVX	0.960	2.567	2.499	2.516	1.567	1.052	0.015	96	97	38	6	-98	6.20E-7	1.13E-5	3.43E-5
HOP-62	0.633	2.468	2.505	2.279	1.291	0.698	0.346	102	90	36	4	-45	5.46E-7	1.18E-5	> 1.00E-4
HOP-92	1.139	1.758	1.664	1.589	1.543	0.918	0.089	85	73	65	-19	-92	1.51E-6	5.89E-6	2.63E-5
NCI-H226	1.037	1.772	1.740	1.696	1.195	0.945	0.050	96	90	22	-9	-95	3.82E-7	5.09E-6	2.99E-5
NCI-H23	0.591	1.905	1.839	1.821	0.612	0.496	0.030	95	94	2	-16	-95	2.98E-7	1.22E-6	2.69E-5
NCI-H322M	0.811	2.201	2.185	2.031	1.342	0.950	0.066	99	88	38	10	-92	5.78E-7	1.25E-5	3.88E-5
NCI-H460	0.259	2.695	2.709	2.716	0.457	0.285	0.054	101	101	8	1	-79	3.53E-7	1.03E-5	4.32E-5
NCI-H522	1.295	3.086	3.010	2.727	0.883	0.843	0.150	96	80	-32	-35	-88	1.85E-7	5.19E-7	1.91E-5
**Colon Cancer**															
COLO 205	0.551	2.530	2.615	2.387	0.231	0.159	0.091	104	93	-58	-71	-83	1.92E-7	4.12E-7	8.84E-7
HCC-2998	0.668	2.526	2.465	2.408	0.688	0.413	0.007	97	94	1	-38	-99	2.96E-7	1.07E-6	1.56E-5
HCT-116	0.301	2.952	2.562	2.422	0.355	0.542	0.137	85	80	2	9	-54	2.42E-7	1.39E-5	8.50E-5
HCT-15	0.311	2.556	2.398	2.275	0.396	0.179	0.003	93	87	4	-43	-99	2.80E-7	1.21E-6	1.35E-5
HT29	0.217	1.695	1.681	1.684	0.332	0.097	0.099	99	99	8	-55	-54	3.45E-7	1.33E-6	8.24E-6
KM12	0.494	2.521	2.555	2.390	0.545	0.307	0.026	102	94	2	-38	-95	3.01E-7	1.15E-6	1.63E-5
SW-620	0.268	2.110	2.127	2.025	0.650	0.288	0.094	101	95	21	1	-65	4.05E-7	1.04E-5	5.94E-5
**CNS Cancer**															
SF-268	1.072	2.876	2.805	2.712	1.692	1.215	0.299	96	91	34	8	-72	5.29E-7	1.26E-5	5.29E-5
SF-295	1.309	3.293	3.187	3.145	1.145	1.109	0.011	95	93	-13	-15	-99	2.54E-7	7.59E-7	2.59E-5
SF-539	0.818	2.433	2.373	2.285	0.553	0.690	-0.001	96	91	-32	-16	-100	2.14E-7	5.45E-7	2.55E-5
SNB-19	0.564	1.977	1.902	1.909	0.677	0.664	0.005	95	95	8	7	-99	3.30E-7	1.17E-5	3.45E-5
SNB-75	1.773	2.823	2.646	2.579	2.164	1.781	0.515	83	77	37	1	-71	4.75E-7	1.02E-5	5.10E-5
U251	0.353	1.916	1.838	1.876	0.682	0.177	0.093	95	97	21	-50	-74	4.18E-7	1.98E-6	1.01E-5
**Melanoma**															
LOX IMVI	0.406	2.840	2.791	2.544	0.887	0.151	0.004	98	88	20	-63	-99	3.60E-7	1.73E-6	6.98E-6
MALME-3M	0.616	1.684	1.707	1.654	1.042	0.574	0.055	102	97	40	-7	-91	6.65E-7	7.14E-6	3.25E-5
M14	0.601	2.512	2.499	2.196	0.714	0.380	0.119	99	83	6	-37	-80	2.70E-7	1.38E-6	2.02E-5
MDA-MB-435	0.676	2.967	2.908	2.832	0.372	0.535	0.024	97	94	-45	-21	-96	2.07E-7	4.75E-7	2.43E-5
SK-MEL-2	0.850	2.014	1.923	1.950	0.929	0.894	0.096	92	95	7	4	-89	3.22E-7	1.10E-5	3.81E-5
SK-MEL-28	0.751	2.059	2.000	1.841	1.287	1.259	0.005	95	83	41	39	-99	6.12E-7	1.91E-5	4.39E-5
SK-MEL-5	0.904	3.195	3.012	2.961	0.474	0.220	0.012	92	90	-48	-76	-99	1.95E-7	4.51E-7	1.22E-6
UACC-257	0.919	2.357	2.294	2.217	1.659	1.074	0.187	96	90	51	11	-80	1.09E-6	1.31E-5	4.70E-5
UACC-62	0.974	2.799	2.584	2.475	1.559	0.644	0.012	88	82	32	-34	-99	4.39E-7	3.06E-6	1.77E-5
**Ovarian Cancer**															
IGROV1	0.436	1.986	2.020	2.029	0.758	0.387	0.085	102	103	21	-11	-81	4.40E-7	4.45E-6	3.62E-5
OVCAR-3	0.626	1.971	2.078	2.012	0.579	0.406	0.016	108	103	-8	-35	-97	3.02E-7	8.54E-7	1.73E-5
OVCAR-4	0.715	1.930	1.888	1.861	1.380	0.941	0.041	97	94	55	19	-94	1.35E-6	1.46E-5	4.05E-5
OVCAR-5	0.447	1.578	1.523	1.478	0.614	0.387	0.001	95	91	15	-13	-100	3.46E-7	3.34E-6	2.65E-5
OVCAR-8	0.337	1.885	1.798	1.821	0.350	0.196	0.092	94	96	1	-42	-73	3.04E-7	1.05E-6	1.82E-5
NCI/ADR-RES	0.357	1.310	1.352	1.238	0.172	0.148	0.027	104	92	-52	-59	-93	1.97E-7	4.37E-7	9.71E-7
SK-OV-3	0.838	2.125	1.977	2.193	1.471	0.911	0.247	88	105	49	6	-71	9.66E-7	1.19E-5	5.38E-5
**Renal Cancer**															
786-0	0.777	2.862	2.741	2.705	1.677	0.875	0.245	94	92	43	5	-68	7.27E-7	1.16E-5	5.59E-5
A498	1.209	2.202	2.190	2.182	2.213	1.525	0.069	99	98	101	32	-94	5.47E-6	1.79E-5	4.45E-5
ACHN	0.374	1.749	1.719	1.669	0.692	0.297	-0.005	98	94	23	-21	-100	4.18E-7	3.38E-6	2.35E-5
CAKI-1	0.495	1.813	1.690	1.519	0.814	0.453	0.010	91	78	24	-9	-98	3.29E-7	5.47E-6	2.90E-5
RXF 393	0.750	1.437	1.484	1.357	0.979	0.853	0.021	107	88	33	15	-97	4.97E-7	1.36E-5	3.79E-5
SN12C	0.631	2.173	2.089	1.986	0.797	0.181	0.007	95	88	11	-71	-99	3.10E-7	1.35E-6	5.49E-6
TK-10	1.084	2.166	2.136	2.254	2.044	1.374	0.098	97	108	89	27	-91	4.21E-6	1.69E-5	4.49E-5
UO-31	0.664	2.098	1.871	1.740	1.286	0.601	0.002	84	75	43	-10	-100	6.18E-7	6.60E-6	2.81E-5
**Prostate Cancer**															
PC-3	0.562	2.233	2.212	2.066	0.918	0.608	0.067	99	90	21	3	-88	3.82E-7	1.07E-5	3.81E-5
DU-145	0.375	1.685	1.754	1.733	0.446	0.346	0.012	105	104	5	-8	-97	3.52E-7	2.56E-6	2.97E-5
**Breast Cancer**															
MCF7	0.450	2.377	2.223	2.105	0.628	0.367	0.076	92	86	9	-18	-83	2.94E-7	2.16E-6	3.07E-5
MDA-MB-231/ATCC	0.566	1.142	1.145	1.078	0.408	0.335	0.003	100	89	-28	-41	-100	2.15E-7	5.76E-7	1.43E-5
HS 578T	1.374	2.589	2.491	2.336	1.669	1.517	0.912	92	79	24	12	-34	3.40E-7	1.81E-5	> 1.00E-4
BT-549	1.398	2.877	2.650	2.514	1.758	1.360	0.177	85	75	24	-3	-87	3.14E-7	7.93E-6	3.62E-5
T-47D	0.747	2.065	1.981	1.964	1.479	0.977	0.328	94	92	56	17	-56	1.40E-6	1.73E-5	8.25E-5
MDA-MB-468	0.773	1.481	1.476	1.428	0.587	0.435	0.011	99	92	-24	-44	-99	2.31E-7	6.22E-7	1.30E-5

Compound **3f**

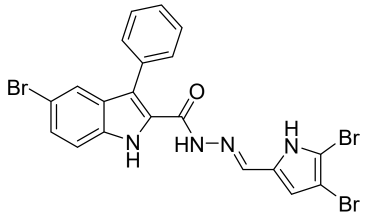

Br
O
N
H
HN–N
H
N
Br
Br

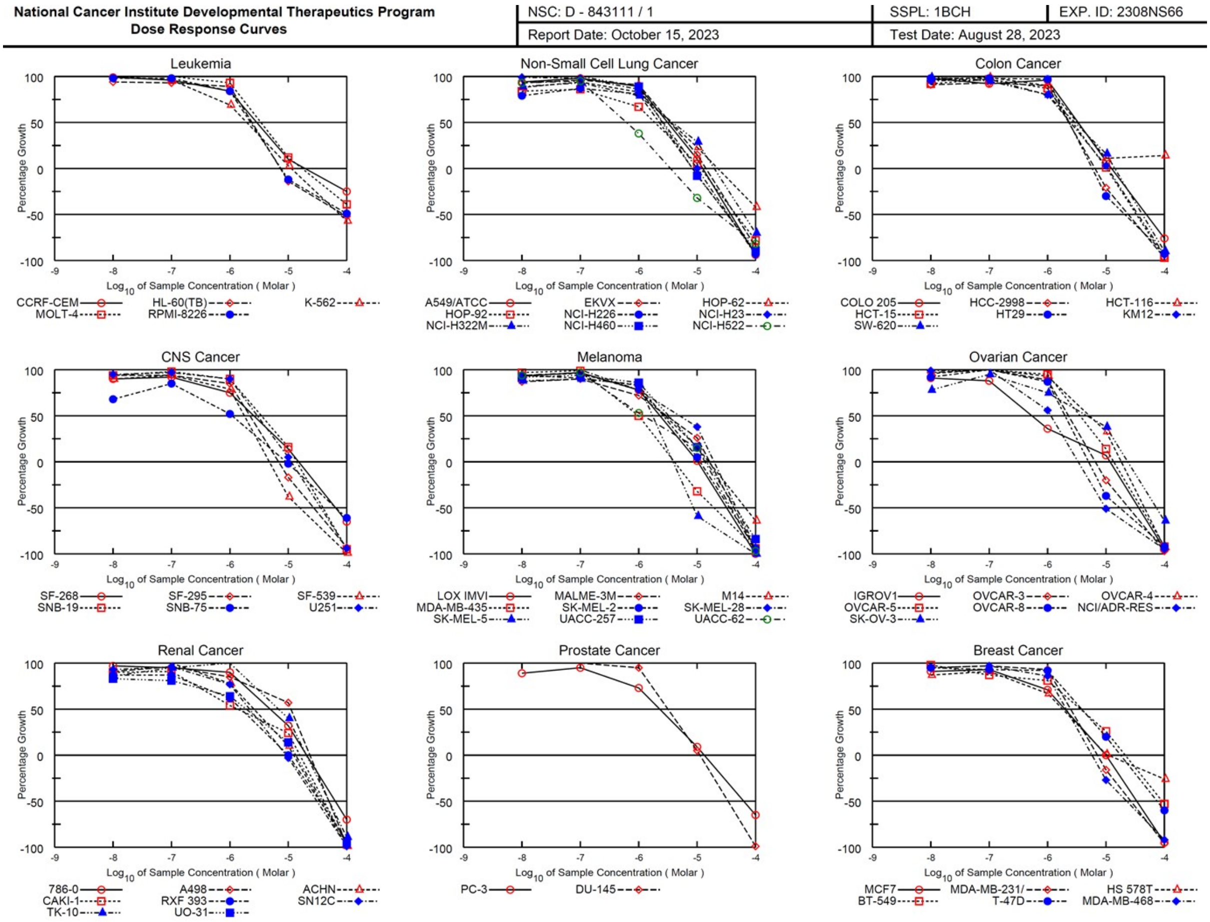

National Cancer Institute Developmental Therapeutics Program
Dose Response Curves
NSC: D - 843111 / 1
Report Date: October 15, 2023
SSPL: 1BCH
EXP. ID: 2308NS66
Test Date: August 28, 2023
Leukemia
Non-Small Cell Lung Cancer
Colon Cancer
CNS Cancer
Melanoma
Ovarian Cancer
Renal Cancer
Prostate Cancer
Breast Cancer
Percentage Growth
Log_{10} of Sample Concentration (Molar)
CCRF-CEM HL-60(TB) K-562 MOLT-4 RPMI-8226
A549/ATCC EKVX HOP-62 HOP-92 NCI-H226 NCI-H23 NCI-H322M NCI-H460 NCI-H522
COLO 205 HCC-2998 HCT-116 HCT-15 HT29 KM12 SW-620
SF-268 SF-295 SF-539 SNB-19 SNB-75 U251
LOX IMVI MALME-3M M14 MDA-MB-435 SK-MEL-2 SK-MEL-28 SK-MEL-5 UACC-257 UACC-62
IGROV1 OVCAR-3 OVCAR-4 OVCAR-5 OVCAR-8 NCI/ADR-RES SK-OV-3
786-0 A498 ACHN CAKI-1 RXF 393 SN12C TK-10 UO-31
PC-3 DU-145
MCF7 MDA-MB-231/ HS 578T BT-549 T-47D MDA-MB-468

National Cancer Institute Developmental Therapeutics Program
In-Vitro Testing Results

NSC : D - 843111 / 1	Experiment ID : 2308NS66	Test Type : 08	Units : Molar
Report Date : October 15, 2023	Test Date : August 28, 2023	QNS :	MC :
COMI : To49	Stain Reagent : SRB Dual-Pass Related	SSPL : 1BCH	

	Log10 Concentration														
	Time		Mean Optical Densities					Percent Growth							
Panel/Cell Line	Zero	Ctrl	-8.0	-7.0	-6.0	-5.0	-4.0	-8.0	-7.0	-6.0	-5.0	-4.0	GI50	TGI	LC50
Leukemia															
CCRF-CEM	0.489	2.559	2.537	2.479	2.231	0.696	0.366	99	96	84	10	-25	2.89E-6	1.93E-5	> 1.00E-4
HL-60(TB)	0.609	2.682	2.567	2.528	2.445	0.525	0.293	94	93	89	-14	-52	2.38E-6	7.33E-6	8.88E-5
K-562	0.255	2.238	2.235	2.165	1.617	0.320	0.110	100	96	69	3	-57	1.93E-6	1.13E-5	7.64E-5
MOLT-4	0.491	2.709	2.759	2.827	2.552	0.754	0.301	102	105	93	12	-39	3.38E-6	1.71E-5	> 1.00E-4
RPMI-8226	0.768	2.490	2.455	2.463	2.215	0.678	0.395	98	98	84	-12	-49	2.27E-6	7.53E-6	> 1.00E-4
Non-Small Cell Lung Cancer															
A549/ATCC	0.294	1.978	1.870	1.939	1.796	0.459	0.018	94	98	89	10	-94	3.11E-6	1.24E-5	3.76E-5
EKVX	0.960	2.566	2.448	2.495	2.419	1.198	0.148	93	96	91	15	-85	3.45E-6	1.41E-5	4.48E-5
HOP-62	0.633	2.173	1.990	2.084	1.914	0.980	0.366	88	94	83	23	-42	3.52E-6	2.23E-5	> 1.00E-4
HOP-92	1.139	1.785	1.681	1.692	1.572	1.171	0.251	84	86	67	5	-78	1.88E-6	1.15E-5	4.60E-5
NCI-H226	1.037	1.796	1.637	1.699	1.655	0.970	0.090	79	87	81	-7	-91	2.28E-6	8.43E-6	3.25E-5
NCI-H23	0.591	1.923	1.911	1.898	1.735	0.591	0.035	99	98	86	0	-94	2.62E-6	1.00E-5	3.40E-5
NCI-H322M	0.811	2.182	2.035	2.090	1.889	1.202	0.245	89	93	79	29	-70	3.73E-6	1.95E-5	6.28E-5
NCI-H460	0.259	2.634	2.712	2.739	2.383	0.237	0.028	103	104	89	-8	-89	2.53E-6	8.19E-6	3.27E-5
NCI-H522	1.295	3.037	2.921	2.948	1.964	0.884	0.237	93	95	38	-32	-82	6.23E-7	3.53E-6	2.32E-5
Colon Cancer															
COLO 205	0.551	2.497	2.452	2.350	2.425	0.714	0.130	98	92	96	8	-76	3.36E-6	1.25E-5	4.88E-5
HCC-2998	0.668	2.623	2.446	2.493	2.434	0.527	0.020	91	93	90	-21	-97	2.30E-6	6.46E-6	2.40E-5
HCT-116	0.301	2.836	2.738	2.798	2.591	0.590	0.661	96	99	90	11	14	3.24E-6	> 1.00E-4	> 1.00E-4
HCT-15	0.311	2.557	2.383	2.488	2.265	0.340	0.009	92	97	87	1	-97	2.70E-6	1.03E-5	3.31E-5
HT29	0.217	1.609	1.587	1.580	1.562	0.151	0.018	98	98	97	-30	-92	2.33E-6	5.76E-6	2.08E-5
KM12	0.494	2.483	2.390	2.426	2.086	0.556	0.030	95	97	80	3	-94	2.46E-6	1.08E-5	3.53E-5
SW-620	0.268	2.045	2.035	1.959	1.691	0.556	0.027	99	95	80	16	-90	2.96E-6	1.42E-5	4.19E-5
CNS Cancer															
SF-268	1.072	2.868	2.682	2.717	2.418	1.327	0.380	90	92	75	14	-65	2.57E-6	1.51E-5	6.53E-5
SF-295	1.309	3.277	3.157	3.166	2.981	1.082	0.088	94	94	85	-17	-93	2.20E-6	6.76E-6	2.69E-5
SF-539	0.818	2.447	2.289	2.352	2.110	0.507	0.010	90	94	79	-38	-99	1.78E-6	4.74E-6	1.57E-5
SNB-19	0.564	1.903	1.822	1.871	1.772	0.776	0.029	94	98	90	16	-95	3.47E-6	1.39E-5	3.93E-5
SNB-75	1.773	2.711	2.414	2.569	2.260	1.745	0.684	68	85	52	-2	-61	1.08E-6	9.33E-6	6.44E-5
U251	0.353	1.764	1.687	1.727	1.617	0.428	0.021	95	97	90	5	-94	2.95E-6	1.13E-5	3.60E-5
Melanoma															
LOX IMVI	0.406	2.859	2.677	2.790	2.323	0.421	.	93	97	78	1	-100	2.31E-6	1.01E-5	3.18E-5
MALME-3M	0.616	1.681	1.548	1.570	1.380	0.891	0.031	87	90	72	26	-95	2.97E-6	1.63E-5	4.24E-5
M14	0.601	2.581	2.469	2.499	2.143	0.912	0.214	94	96	78	16	-64	2.81E-6	1.57E-5	6.61E-5
MDA-MB-435	0.676	2.887	2.825	2.870	1.790	0.461	0.040	97	99	50	-32	-94	1.01E-6	4.10E-6	1.96E-5
SK-MEL-2	0.850	2.043	1.966	1.933	1.845	0.916	0.055	94	91	83	5	-94	2.68E-6	1.14E-5	3.63E-5
SK-MEL-28	0.751	2.144	2.173	2.218	1.843	1.284	0.053	102	105	78	38	-93	5.10E-6	1.96E-5	4.70E-5
SK-MEL-5	0.904	3.201	2.927	2.983	2.827	0.371	0.004	88	90	84	-59	-100	1.72E-6	3.86E-6	8.65E-6
UACC-257	0.919	2.288	2.179	2.213	2.096	1.132	0.150	92	94	86	16	-84	3.24E-6	1.43E-5	4.57E-5
UACC-62	0.974	2.847	2.733	2.792	1.968	1.254	0.030	94	97	53	15	-97	1.20E-6	1.36E-5	3.80E-5
Ovarian Cancer															
IGROV1	0.436	2.147	1.988	1.940	1.045	0.556	0.021	91	88	36	7	-95	5.30E-7	1.17E-5	3.61E-5
OVCAR-3	0.626	1.958	1.918	1.968	1.817	0.503	0.016	97	101	89	-20	-97	2.30E-6	6.59E-6	2.45E-5
OVCAR-4	0.715	1.832	1.792	1.840	1.753	1.083	0.054	96	101	93	33	-93	5.20E-6	1.83E-5	4.58E-5
OVCAR-5	0.447	1.621	1.632	1.662	1.557	0.617	0.035	101	104	95	14	-92	3.60E-6	1.37E-5	4.02E-5
OVCAR-8	0.337	1.868	1.749	1.878	1.663	0.212	0.028	92	101	87	-37	-92	1.98E-6	5.01E-6	1.72E-5
NCI/ADR-RES	0.357	1.333	1.322	1.362	0.904	0.174	0.018	99	103	56	-51	-95	1.14E-6	3.33E-6	9.73E-6
SK-OV-3	0.838	1.889	1.658	1.837	1.627	1.239	0.299	78	95	75	38	-64	4.76E-6	2.36E-5	7.25E-5
Renal Cancer															
786-0	0.777	3.041	2.973	2.932	2.817	1.504	0.236	97	95	90	32	-70	4.91E-6	2.07E-5	6.41E-5
A498	1.209	2.202	2.109	2.164	2.056	1.775	0.034	91	96	85	57	-97	1.11E-5	2.34E-5	4.94E-5
ACHN	0.374	1.700	1.709	1.783	1.408	0.509	0.003	101	106	78	10	-99	2.59E-6	1.24E-5	3.55E-5
CAKI-1	0.495	1.736	1.613	1.620	1.169	0.788	0.017	90	91	54	24	-97	1.38E-6	1.57E-5	4.09E-5
RXF 393	0.750	1.442	1.351	1.349	1.177	0.751	0.049	87	87	62	0	-93	1.55E-6	1.00E-5	3.43E-5
SN12C	0.631	2.311	2.192	2.239	1.918	0.610	0.005	93	96	77	-3	-99	2.15E-6	9.09E-6	3.07E-5
TK-10	1.084	2.183	2.025	2.129	2.194	1.521	0.115	86	95	101	40	-89	6.80E-6	2.03E-5	4.95E-5
UO-31	0.664	2.052	1.816	1.793	1.557	0.865	0.029	83	81	64	14	-96	1.94E-6	1.35E-5	3.85E-5
Prostate Cancer															
PC-3	0.562	2.244	2.060	2.161	1.796	0.706	0.200	89	95	73	9	-65	2.29E-6	1.31E-5	6.33E-5
DU-145	0.375	1.634	1.643	1.668	1.573	0.440	0.005	101	103	95	5	-99	3.18E-6	1.12E-5	3.39E-5
Breast Cancer															
MCF7	0.450	2.385	2.208	2.242	1.833	0.450	0.024	91	93	71	0	-95	1.99E-6	9.96E-6	3.37E-5
MDA-MB-231/ATCC	0.566	1.111	1.085	1.095	1.075	0.474	0.021	95	97	93	-16	-96	2.49E-6	7.10E-6	2.63E-5
HS 578T	1.374	2.564	2.408	2.455	2.173	1.389	1.017	87	91	67	1	-26	1.82E-6	1.11E-5	> 1.00E-4
BT-549	1.398	2.929	2.896	2.733	2.639	1.801	0.653	98	87	81	26	-53	3.69E-6	2.14E-5	9.08E-5
T-47D	0.747	1.941	1.881	1.856	1.849	0.985	0.296	95	93	92	20	-60	3.84E-6	1.77E-5	7.43E-5
MDA-MB-468	0.773	1.451	1.414	1.432	1.356	0.567	0.062	95	97	86	-27	-92	2.09E-6	5.80E-6	2.28E-5

Compound **3g**

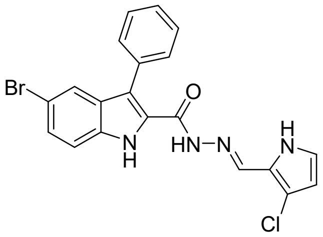
Br
O
HN–N
H
N
H
N
Cl

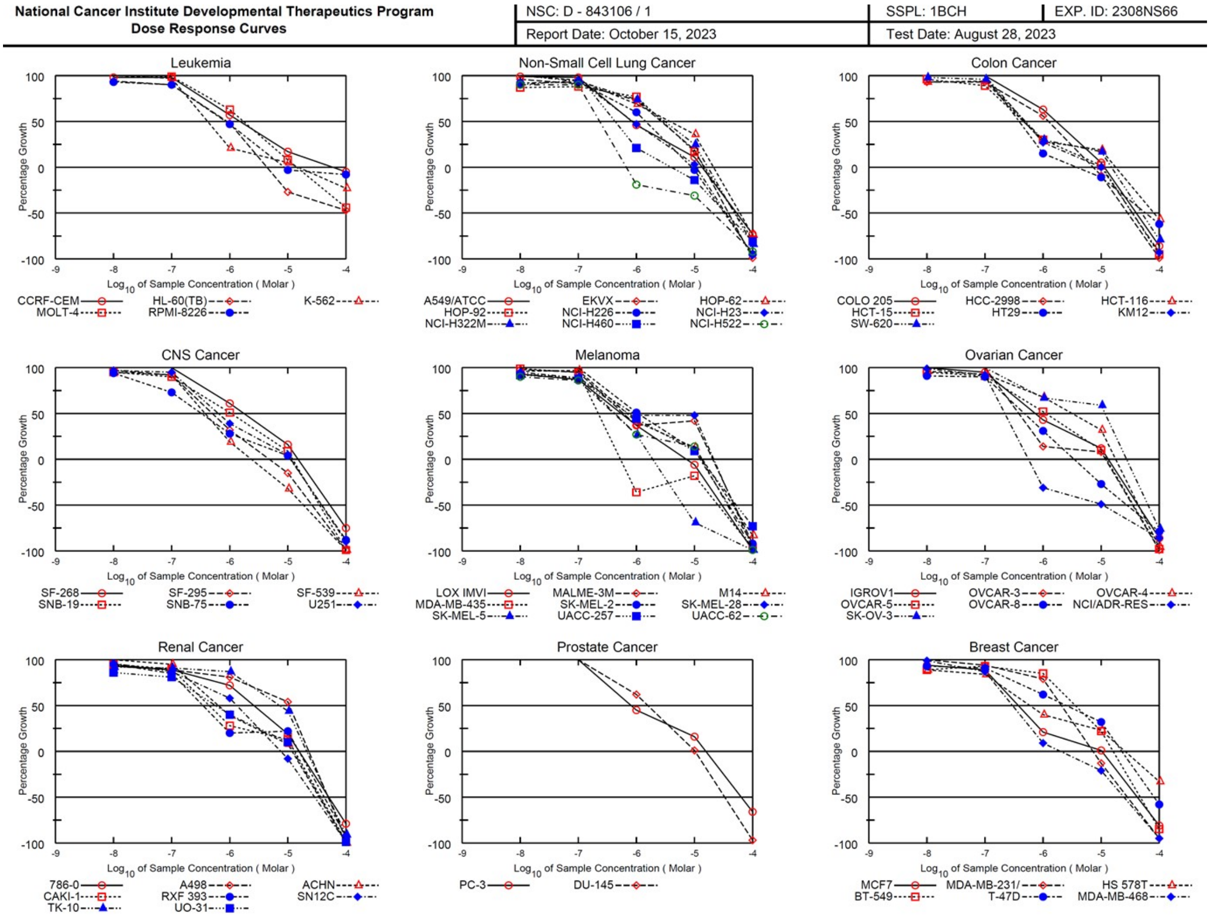
National Cancer Institute Developmental Therapeutics Program
Dose Response Curves
NSC: D - 843106 / 1
Report Date: October 15, 2023
SSPL: 1BCH
EXP. ID: 2308NS66
Test Date: August 28, 2023
Leukemia
Non-Small Cell Lung Cancer
Colon Cancer
CNS Cancer
Melanoma
Ovarian Cancer
Renal Cancer
Prostate Cancer
Breast Cancer
Percentage Growth
Log10 of Sample Concentration (Molar)
CCRF-CEM
HL-60(TB)
K-562
MOLT-4
RPMI-8226
A549/ATCC
EKVX
HOP-62
HOP-92
NCI-H226
NCI-H23
NCI-H322M
NCI-H460
NCI-H522
COLO 205
HCC-2998
HCT-116
HCT-15
HT29
KM12
SW-620
SF-268
SF-295
SF-539
SNB-19
SNB-75
U251
LOX IMVI
MALME-3M
M14
MDA-MB-435
SK-MEL-2
SK-MEL-28
SK-MEL-5
UACC-257
UACC-62
IGROV1
OVCAR-3
OVCAR-4
OVCAR-5
OVCAR-8
NCI/ADR-RES
SK-OV-3
786-0
A498
ACHN
CAKI-1
RXF 393
SN12C
TK-10
UO-31
PC-3
DU-145
MCF7
MDA-MB-231/
HS 578T
BT-549
T-47D
MDA-MB-468

National Cancer Institute Developmental Therapeutics Program
In-Vitro Testing Results

NSC : D - 843106 / 1	Experiment ID : 2308NS66	Test Type : 08	Units : Molar
Report Date : October 15, 2023	Test Date : August 28, 2023	QNS :	MC :
COMI : To44	Stain Reagent : SRB Dual-Pass Related	SSPL : 1BCH	

			Log10 Concentration												
	Time		Mean Optical Densities					Percent Growth							
Panel/Cell Line	Zero	Ctrl	-8.0	-7.0	-6.0	-5.0	-4.0	-8.0	-7.0	-6.0	-5.0	-4.0	GI50	TGI	LC50
Leukemia															
CCRF-CEM	0.489	2.450	2.409	2.406	1.616	0.824	0.465	98	98	57	17	-5	1.53E-6	5.93E-5	> 1.00E-4
HL-60(TB)	0.609	2.329	2.231	2.158	1.433	0.446	0.324	94	90	48	-27	-47	8.91E-7	4.38E-6	> 1.00E-4
K-562	0.255	1.971	2.053	1.919	0.610	0.344	0.196	105	97	21	5	-23	4.13E-7	1.52E-5	> 1.00E-4
MOLT-4	0.491	2.198	2.275	2.178	1.561	0.630	0.274	105	99	63	8	-44	1.71E-6	1.43E-5	> 1.00E-4
RPMI-8226	0.768	2.438	2.323	2.266	1.555	0.746	0.709	93	90	47	-3	-8	8.55E-7	8.74E-6	> 1.00E-4
Non-Small Cell Lung Cancer															
A549/ATCC	0.294	1.897	1.882	1.863	1.032	0.469	0.080	99	98	46	11	-73	8.38E-7	1.35E-5	5.34E-5
EKVX	0.960	2.569	2.505	2.419	2.138	1.258	0.006	96	91	73	19	-99	2.66E-6	1.44E-5	3.81E-5
HOP-62	0.633	2.164	2.230	2.082	1.689	1.189	0.166	104	95	69	36	-74	3.80E-6	2.14E-5	6.08E-5
HOP-92	1.139	1.736	1.655	1.663	1.598	1.238	0.244	87	88	77	17	-79	2.79E-6	1.49E-5	5.01E-5
NCI-H226	1.037	1.813	1.751	1.757	1.503	1.001	0.049	92	93	60	-3	-95	1.44E-6	8.82E-6	3.21E-5
NCI-H23	0.591	1.949	1.819	1.887	1.235	0.637	0.024	90	95	47	3	-96	8.84E-7	1.08E-5	3.44E-5
NCI-H322M	0.811	2.290	2.317	2.197	1.910	1.188	0.128	102	94	74	25	-84	3.14E-6	1.71E-5	4.88E-5
NCI-H460	0.259	2.640	2.756	2.720	0.768	0.222	0.050	105	103	21	-14	-81	4.48E-7	3.95E-6	3.43E-5
NCI-H522	1.295	3.018	2.838	2.850	1.047	0.889	0.110	90	90	-19	-31	-92	2.33E-7	6.68E-7	2.04E-5
Colon Cancer															
COLO 205	0.551	2.402	2.484	2.456	1.711	0.643	0.078	104	103	63	5	-86	1.66E-6	1.13E-5	4.03E-5
HCC-2998	0.668	2.648	2.506	2.502	1.773	0.607	0.008	93	93	56	-9	-99	1.23E-6	7.23E-6	2.86E-5
HCT-116	0.301	2.727	2.553	2.577	0.989	0.770	0.128	93	94	28	19	-57	4.67E-7	1.79E-5	7.99E-5
HCT-15	0.311	2.558	2.463	2.317	0.994	0.366	0.014	96	89	30	2	-95	4.65E-7	1.06E-5	3.43E-5
HT29	0.217	1.552	1.579	1.590	0.421	0.193	0.083	102	103	15	-11	-62	4.01E-7	3.75E-6	5.85E-5
KM12	0.494	2.468	2.479	2.465	1.036	0.501	0.035	101	100	27	0	-93	4.88E-7	1.01E-5	3.46E-5
SW-620	0.268	2.027	1.999	1.958	0.797	0.573	0.057	98	96	30	17	-79	4.99E-7	1.52E-5	5.02E-5
CNS Cancer															
SF-268	1.072	2.841	2.875	2.836	2.151	1.358	0.266	102	100	61	16	-75	1.76E-6	1.50E-5	5.30E-5
SF-295	1.309	3.281	3.212	3.116	1.944	1.112	0.009	97	92	32	-15	-99	5.01E-7	4.80E-6	2.60E-5
SF-539	0.818	2.464	2.379	2.327	1.139	0.556	0.006	95	92	19	-32	-99	3.78E-7	2.39E-6	1.85E-5
SNB-19	0.564	1.998	1.928	1.855	1.295	0.695	0.008	95	90	51	9	-99	1.06E-6	1.22E-5	3.54E-5
SNB-75	1.773	2.794	2.729	2.522	2.056	1.813	0.214	94	73	28	4	-88	3.25E-7	1.10E-5	3.86E-5
U251	0.353	1.742	1.705	1.672	0.891	0.423	0.034	97	95	39	5	-90	6.30E-7	1.13E-5	3.77E-5
Melanoma															
LOX IMVI	0.406	2.881	2.708	2.562	1.328	0.380	0.003	93	87	37	-6	-99	5.55E-7	7.13E-6	2.94E-5
MALME-3M	0.616	1.690	1.664	1.634	1.014	1.063	0.048	98	95	37	42	-92	5.96E-7	2.05E-5	4.84E-5
M14	0.601	2.499	2.419	2.441	1.378	0.857	0.102	96	97	41	13	-83	6.89E-7	1.38E-5	4.54E-5
MDA-MB-435	0.676	2.976	2.964	2.872	0.436	0.555	0.021	99	95	-36	-18	-97	2.22E-7	5.35E-7	2.55E-5
SK-MEL-2	0.850	1.936	1.935	2.005	1.405	0.961	0.066	100	106	51	10	-92	1.07E-6	1.26E-5	3.87E-5
SK-MEL-28	0.751	2.026	1.967	1.889	1.367	1.363	0.025	95	89	48	48	-97	9.09E-7	2.15E-5	4.75E-5
SK-MEL-5	0.904	3.244	3.073	2.991	1.537	0.278	0.012	93	89	27	-69	-99	4.27E-7	1.91E-6	6.30E-6
UACC-257	0.919	2.284	2.171	2.120	1.523	1.040	0.245	92	88	44	9	-73	7.38E-7	1.28E-5	5.20E-5
UACC-62	0.974	2.820	2.641	2.563	1.479	1.234	0.014	90	86	27	14	-99	4.11E-7	1.33E-5	3.70E-5
Ovarian Cancer															
IGROV1	0.436	2.077	2.143	1.998	1.141	0.639	0.061	104	95	43	12	-86	7.34E-7	1.33E-5	4.30E-5
OVCAR-3	0.626	1.986	2.016	2.066	0.823	0.730	0.009	102	106	14	8	-99	4.09E-7	1.18E-5	3.49E-5
OVCAR-4	0.715	1.919	1.875	1.831	1.529	1.096	0.027	96	93	68	32	-96	3.09E-6	1.77E-5	4.35E-5
OVCAR-5	0.447	1.600	1.541	1.501	1.044	0.560	0.007	95	91	52	10	-98	1.10E-6	1.23E-5	3.57E-5
OVCAR-8	0.337	1.912	1.775	1.762	0.828	0.247	0.070	91	90	31	-27	-79	4.81E-7	3.45E-6	2.77E-5
NCI/ADR-RES	0.357	1.353	1.344	1.270	0.248	0.182	0.049	99	92	-31	-49	-86	2.19E-7	5.63E-7	1.05E-5
SK-OV-3	0.838	1.831	1.864	1.863	1.499	1.421	0.202	103	103	67	59	-76	1.16E-5	2.73E-5	6.41E-5
Renal Cancer															
786-0	0.777	3.052	2.886	2.792	2.408	1.211	0.160	93	89	72	19	-79	2.58E-6	1.56E-5	5.02E-5
A498	1.209	2.193	2.130	2.087	2.003	1.739	0.049	94	89	81	54	-96	1.06E-5	2.29E-5	4.93E-5
ACHN	0.374	1.749	1.779	1.680	0.905	0.485	-0.001	102	95	39	8	-100	6.27E-7	1.19E-5	3.45E-5
CAKI-1	0.495	1.770	1.707	1.643	0.856	0.662	0.009	95	90	28	13	-98	4.45E-7	1.31E-5	3.69E-5
RXF 393	0.750	1.441	1.407	1.339	0.887	0.905	0.049	95	85	20	22	-93	3.46E-7	1.56E-5	4.21E-5
SN12C	0.631	2.207	2.151	2.010	1.552	0.584	0.008	96	87	58	-8	-99	1.34E-6	7.69E-6	2.92E-5
TK-10	1.084	2.110	2.025	2.021	1.978	1.532	0.096	92	91	87	44	-91	7.16E-6	2.11E-5	4.95E-5
UO-31	0.664	2.085	1.880	1.816	1.236	0.812	0.007	86	81	40	10	-99	5.77E-7	1.24E-5	3.57E-5
Prostate Cancer															
PC-3	0.562	2.181	2.335	2.294	1.288	0.823	0.190	109	107	45	16	-66	8.26E-7	1.57E-5	6.36E-5
DU-145	0.375	1.649	1.788	1.741	1.167	0.391	0.011	111	107	62	1	-97	1.58E-6	1.03E-5	3.31E-5
Breast Cancer															
MCF7	0.450	2.394	2.273	2.182	0.862	0.478	0.086	94	89	21	1	-81	3.76E-7	1.04E-5	4.22E-5
MDA-MB-231/ATCC	0.566	1.142	1.140	1.106	1.022	0.492	0.029	100	94	79	-13	-95	2.07E-6	7.20E-6	2.82E-5
HS 578T	1.374	2.573	2.439	2.386	1.851	1.646	0.919	89	84	40	23	-33	5.90E-7	2.55E-5	> 1.00E-4
BT-549	1.398	2.810	2.648	2.713	2.592	1.708	0.204	89	93	85	22	-85	3.56E-6	1.60E-5	4.68E-5
T-47D	0.747	2.058	1.963	1.934	1.561	1.163	0.316	93	91	62	32	-58	2.50E-6	2.26E-5	8.19E-5
MDA-MB-468	0.773	1.481	1.475	1.386	0.839	0.613	0.037	99	87	9	-21	-95	2.97E-7	2.04E-6	2.47E-5

Compound **3h**

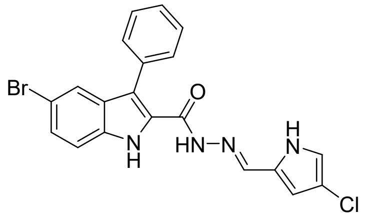

National Cancer Institute Developmental Therapeutics Program Dose Response Curves	NSC: D - 845424 / 1	SSPL: 1CXF	EXP. ID: 2310NS86
	Report Date: November 08, 2023	Test Date: October 02, 2023	

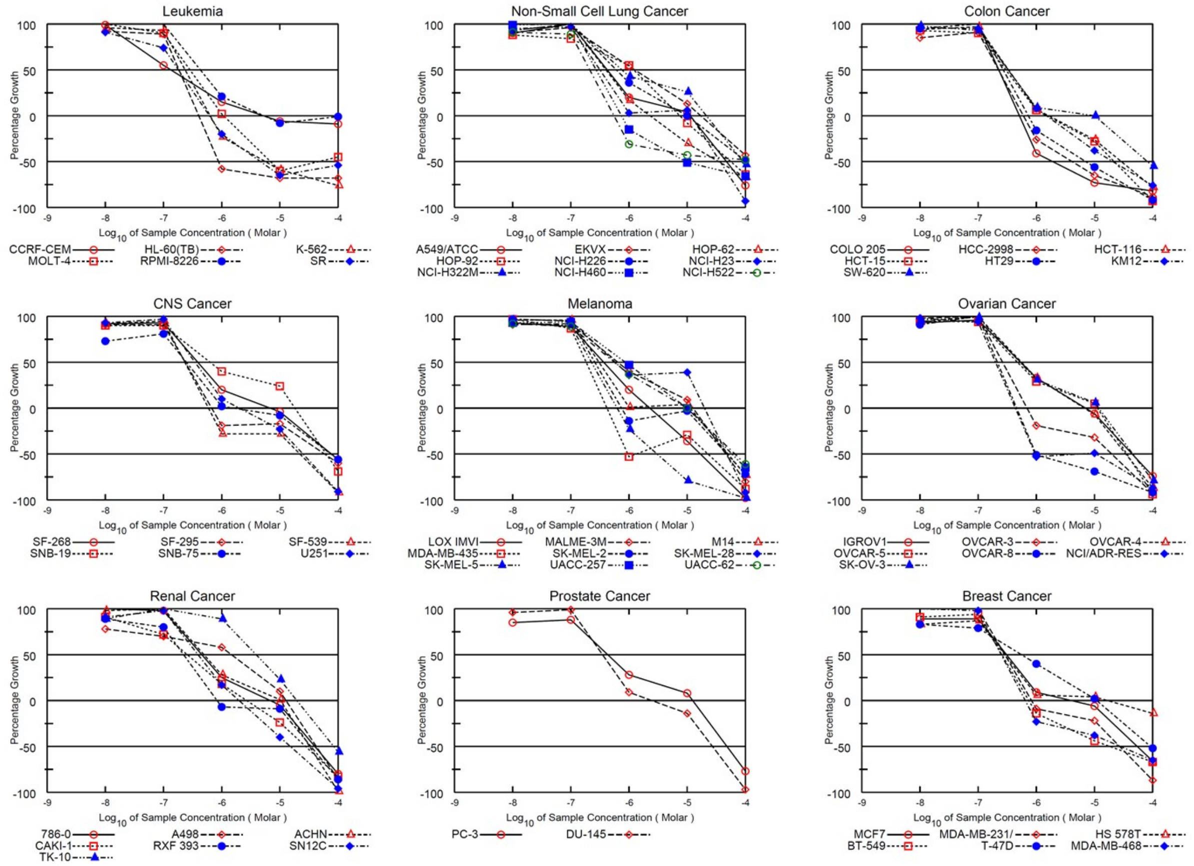

National Cancer Institute Developmental Therapeutics Program
In-Vitro Testing Results

NSC : D - 845424 / 1	Experiment ID : 2310NS86	Test Type : 08	Units : Molar
Report Date : November 08, 2023	Test Date : October 02, 2023	QNS :	MC :
COMI : TO53	Stain Reagent : SRB Dual-Pass Related	SSPL : 1CXF	

			Log10 Concentration												
	Time		Mean Optical Densities					Percent Growth							
Panel/Cell Line	Zero	Ctrl	-8.0	-7.0	-6.0	-5.0	-4.0	-8.0	-7.0	-6.0	-5.0	-4.0	GI50	TGI	LC50
Leukemia															
CCRF-CEM	0.563	2.580	2.567	1.668	0.858	0.532	0.514	99	55	15	-6	-9	1.32E-7	5.32E-6	> 1.00E-4
HL-60(TB)	0.776	2.567	2.430	2.377	0.328	0.248	0.246	92	89	-58	-68	-68	1.85E-7	4.05E-7	8.86E-7
K-562	0.221	1.286	1.243	1.207	0.171	0.091	0.053	96	93	-23	-59	-76	2.34E-7	6.36E-7	5.64E-6
MOLT-4	0.609	2.053	2.189	1.908	0.632	0.245	0.334	109	90	2	-60	-45	2.83E-7	1.06E-6	.
RPMI-8226	0.518	1.951	2.011	1.996	0.824	0.478	0.512	104	103	21	-8	-1	4.46E-7	5.39E-6	> 1.00E-4
SR	0.364	1.347	1.259	1.088	0.291	0.127	0.166	91	74	-20	-65	-54	1.79E-7	6.11E-7	4.60E-6
Non-Small Cell Lung Cancer															
A549/ATCC	0.333	1.903	1.745	1.926	0.651	0.397	0.080	90	101	20	4	-76	4.30E-7	1.12E-5	4.74E-5
EKVX	1.086	2.487	2.360	2.428	1.849	1.272	0.623	91	96	54	13	-43	1.28E-6	1.73E-5	> 1.00E-4
HOP-62	0.559	1.541	1.540	1.556	0.728	0.392	0.184	100	102	17	-30	-67	4.08E-7	2.31E-6	3.47E-5
HOP-92	1.079	1.763	1.678	1.657	1.454	0.991	0.384	88	84	55	-8	-64	1.19E-6	7.42E-6	5.54E-5
NCI-H226	1.191	2.326	2.243	2.321	1.601	1.181	0.610	93	100	36	0	-49	6.04E-7	9.49E-6	> 1.00E-4
NCI-H23	0.621	2.296	2.208	2.254	0.668	0.719	0.041	95	97	3	6	-93	3.17E-7	1.14E-5	3.65E-5
NCI-H322M	0.862	2.331	2.231	2.346	1.500	1.244	0.408	93	101	43	26	-53	7.68E-7	2.14E-5	9.23E-5
NCI-H460	0.359	2.328	2.308	2.383	0.306	0.175	0.123	99	103	-15	-51	-66	2.81E-7	7.49E-7	9.24E-6
NCI-H522	1.404	2.894	2.766	2.727	0.968	0.805	0.730	91	89	-31	-43	-48	2.11E-7	5.51E-7	> 1.00E-4
Colon Cancer															
COLO 205	0.503	1.914	1.988	2.067	0.296	0.134	0.090	105	111	-41	-73	-82	2.51E-7	5.36E-7	1.88E-6
HCC-2998	0.853	3.119	2.776	2.909	0.633	0.302	0.104	85	91	-26	-65	-88	2.24E-7	6.01E-7	4.20E-6
HCT-116	0.349	2.741	2.589	2.660	0.514	0.259	0.077	94	97	7	-26	-78	3.31E-7	1.63E-6	2.91E-5
HCT-15	0.382	2.735	2.573	2.510	0.528	0.276	0.026	93	90	6	-28	-93	3.02E-7	1.52E-6	2.19E-5
HT29	0.257	1.694	1.624	1.778	0.216	0.114	0.020	95	106	-16	-56	-92	2.87E-7	7.37E-7	7.20E-6
KM12	0.611	2.688	2.608	2.584	0.775	0.380	0.146	96	95	8	-38	-76	3.28E-7	1.49E-6	2.07E-5
SW-620	0.402	1.952	1.921	1.850	0.545	0.406	0.181	98	93	9	0	-55	3.28E-7	1.01E-5	8.09E-5
CNS Cancer															
SF-268	0.763	2.115	2.013	2.016	1.035	0.731	0.338	92	93	20	-4	-56	3.88E-7	6.69E-6	7.73E-5
SF-295	1.288	3.130	2.971	2.971	1.044	1.074	0.514	91	91	-19	-17	-60	2.37E-7	6.73E-7	5.85E-5
SF-539	0.714	2.410	2.270	2.318	0.515	0.515	0.057	92	95	-28	-28	-92	2.31E-7	5.91E-7	2.21E-5
SNB-19	0.850	2.368	2.222	2.220	1.461	1.214	0.260	90	90	40	24	-69	6.38E-7	1.81E-5	6.19E-5
SNB-75	0.927	1.458	1.313	1.359	0.935	0.852	0.410	73	81	2	-8	-56	2.47E-7	1.44E-6	7.55E-5
U251	0.268	1.411	1.333	1.381	0.383	0.206	0.024	93	97	10	-23	-91	3.49E-7	2.01E-6	2.49E-5
Melanoma															
LOX IMVI	0.464	2.633	2.481	2.403	0.901	0.295	0.012	93	89	20	-36	-98	3.70E-7	2.27E-6	1.67E-5
MALME-3M	0.739	1.671	1.588	1.595	1.113	0.828	0.147	91	92	40	9	-80	6.45E-7	1.28E-5	4.61E-5
M14	0.494	1.782	1.736	1.730	0.508	0.543	0.134	96	96	1	4	-73	3.05E-7	1.12E-5	5.03E-5
MDA-MB-435	0.751	2.341	2.287	2.140	0.355	0.535	0.087	97	87	-53	-29	-88	1.85E-7	4.20E-7	.
SK-MEL-2	1.244	2.432	2.396	2.375	1.072	1.206	0.348	97	95	-14	-3	-72	2.60E-7	7.47E-7	4.79E-5
SK-MEL-28	0.803	2.295	2.256	2.227	1.341	1.384	0.066	97	95	36	39	-92	5.82E-7	1.99E-5	4.79E-5
SK-MEL-5	1.062	3.211	3.059	3.003	0.822	0.228	0.016	93	90	-23	-79	-98	2.28E-7	6.30E-7	3.08E-6
UACC-257	1.089	2.637	2.525	2.545	1.814	1.093	0.381	93	94	47	0	-65	8.56E-7	1.01E-5	5.88E-5
UACC-62	1.130	3.072	2.922	2.865	1.843	1.136	0.442	92	89	37	0	-61	5.59E-7	1.01E-5	6.63E-5
Ovarian Cancer															
IGROV1	0.598	2.066	1.967	2.003	1.085	0.563	0.154	93	96	33	-6	-74	5.38E-7	7.08E-6	4.41E-5
OVCAR-3	0.648	1.918	1.888	1.950	0.523	0.440	0.046	98	103	-19	-32	-93	2.70E-7	6.94E-7	1.97E-5
OVCAR-4	0.950	2.156	2.085	2.160	1.345	0.880	0.120	94	100	33	-7	-87	5.55E-7	6.53E-6	3.41E-5
OVCAR-5	0.593	1.464	1.419	1.410	0.845	0.635	0.038	95	94	29	5	-94	4.73E-7	1.12E-5	3.60E-5
OVCAR-8	0.526	2.524	2.337	2.517	0.259	0.165	0.044	91	100	-51	-69	-92	2.14E-7	4.60E-7	9.88E-7
NCI/ADR-RES	0.585	2.053	2.026	1.970	0.275	0.297	0.075	98	94	-53	-49	-87	2.00E-7	4.36E-7	.
SK-OV-3	0.720	1.622	1.588	1.610	0.996	0.772	0.151	96	99	31	6	-79	5.19E-7	1.17E-5	4.54E-5
Renal Cancer															
786-0	0.686	2.622	2.628	2.589	1.176	0.652	0.137	100	98	25	-5	-80	4.59E-7	6.82E-6	3.98E-5
A498	1.481	2.269	2.097	2.031	1.936	1.562	0.204	78	70	58	10	-86	1.45E-6	1.28E-5	4.21E-5
ACHN	0.349	1.700	1.675	1.701	0.725	0.360	0.002	98	100	28	1	-99	4.93E-7	1.02E-5	3.21E-5
CAKI-1	0.581	2.043	1.907	1.628	0.845	0.440	0.101	91	72	18	-24	-83	2.53E-7	2.66E-6	2.75E-5
RXF 393	1.053	1.744	1.670	1.604	0.977	0.962	0.149	89	80	-7	-9	-86	2.19E-7	8.25E-7	3.43E-5
SN12C	0.543	2.017	1.887	1.986	0.793	0.328	0.021	91	98	17	-40	-96	3.91E-7	2.00E-6	1.53E-5
TK-10	1.244	2.111	2.014	2.113	2.013	1.447	0.543	89	100	89	23	-56	3.91E-6	1.96E-5	8.32E-5
Prostate Cancer															
PC-3	0.515	2.026	1.798	1.846	0.932	0.632	0.116	85	88	28	8	-77	4.26E-7	1.23E-5	4.76E-5
DU-145	0.361	1.568	1.523	1.558	0.472	0.310	0.013	96	99	9	-14	-97	3.52E-7	2.46E-6	2.72E-5
Breast Cancer															
MCF7	0.544	2.358	2.158	2.162	0.712	0.510	0.184	89	89	9	-6	-66	3.09E-7	3.92E-6	5.35E-5
MDA-MB-231/ATCC	0.605	1.214	1.237	1.228	0.553	0.471	0.079	104	102	-9	-22	-87	2.96E-7	8.35E-7	2.69E-5
HS 578T	1.372	2.393	2.219	2.256	1.431	1.410	1.175	83	87	6	4	-14	2.84E-7	1.61E-5	> 1.00E-4
BT-549	1.308	2.255	2.170	2.199	1.122	0.731	0.435	91	94	-14	-44	-67	2.55E-7	7.39E-7	1.81E-5
T-47D	0.798	1.842	1.664	1.627	1.211	0.824	0.385	83	79	40	2	-52	5.46E-7	1.11E-5	9.26E-5
MDA-MB-468	1.202	2.316	2.335	2.300	0.928	0.741	0.426	102	98	-23	-38	-65	2.51E-7	6.49E-7	2.78E-5

Compound **3i**

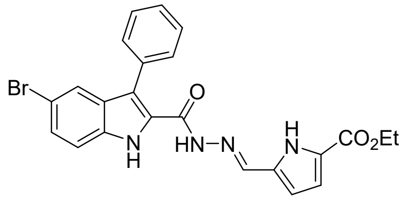

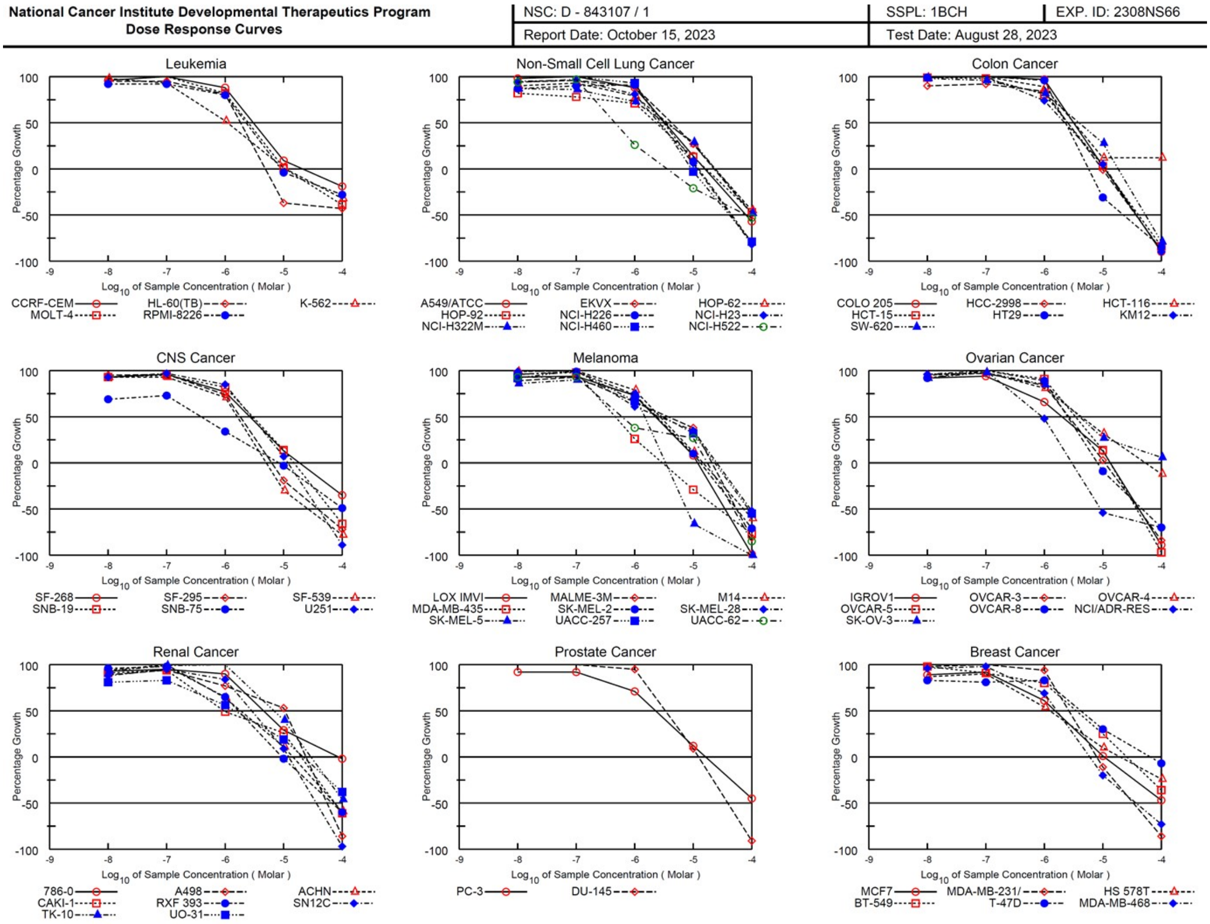
National Cancer Institute Developmental Therapeutics Program
Dose Response Curves
NSC: D - 843107 / 1
Report Date: October 15, 2023
SSPL: 1BCH
EXP. ID: 2308NS66
Test Date: August 28, 2023
Leukemia
Non-Small Cell Lung Cancer
Colon Cancer
CNS Cancer
Melanoma
Ovarian Cancer
Renal Cancer
Prostate Cancer
Breast Cancer
Percentage Growth
Log10 of Sample Concentration (Molar)
CCRF-CEM
HL-60(TB)
K-562
MOLT-4
RPMI-8226
A549/ATCC
EKVX
HOP-62
HOP-92
NCI-H226
NCI-H23
NCI-H322M
NCI-H460
NCI-H522
COLO 205
HCC-2998
HCT-116
HCT-15
HT29
KM12
SW-620
SF-268
SF-295
SF-539
SNB-19
SNB-75
U251
LOX IMVI
MALME-3M
M14
MDA-MB-435
SK-MEL-2
SK-MEL-28
SK-MEL-5
UACC-257
UACC-62
IGROV1
OVCAR-3
OVCAR-4
OVCAR-5
OVCAR-8
NCI/ADR-RES
SK-OV-3
786-0
A498
ACHN
CAKI-1
RXF 393
SN12C
TK-10
UO-31
PC-3
DU-145
MCF7
MDA-MB-231/
HS 578T
BT-549
T-47D
MDA-MB-468

National Cancer Institute Developmental Therapeutics Program
In-Vitro Testing Results

NSC : D - 843107 / 1	Experiment ID : 2308NS66	Test Type : 08	Units : Molar
Report Date : October 15, 2023	Test Date : August 28, 2023	QNS :	MC :
COMI : To45	Stain Reagent : SRB Dual-Pass Related	SSPL : 1BCH	

			Log10 Concentration												
	Time		Mean Optical Densities					Percent Growth							
Panel/Cell Line	Zero	Ctrl	-8.0	-7.0	-6.0	-5.0	-4.0	-8.0	-7.0	-6.0	-5.0	-4.0	GI50	TGI	LC50
Leukemia															
CCRF-CEM	0.489	2.450	2.367	2.446	2.221	0.666	0.397	96	100	88	9	-19	3.04E-6	2.10E-5	> 1.00E-4
HL-60(TB)	0.609	2.329	2.251	2.246	2.009	0.383	0.346	95	95	81	-37	-43	1.84E-6	4.86E-6	> 1.00E-4
K-562	0.255	1.971	1.933	1.850	1.142	0.274	0.173	98	93	52	1	-32	1.08E-6	1.08E-5	> 1.00E-4
MOLT-4	0.491	2.198	2.242	2.195	1.887	0.516	0.300	103	100	82	1	-39	2.49E-6	1.09E-5	> 1.00E-4
RPMI-8226	0.768	2.438	2.306	2.298	2.111	0.735	0.550	92	92	80	-4	-28	2.28E-6	8.88E-6	> 1.00E-4
Non-Small Cell Lung Cancer															
A549/ATCC	0.294	1.897	1.867	1.943	1.700	0.520	0.126	98	103	88	14	-57	3.25E-6	1.58E-5	7.94E-5
EKVX	0.960	2.569	2.470	2.510	2.396	1.397	0.492	94	96	89	27	-49	4.29E-6	2.28E-5	> 1.00E-4
HOP-62	0.633	2.164	2.015	2.063	1.843	1.053	0.351	90	93	79	27	-45	3.65E-6	2.40E-5	> 1.00E-4
HOP-92	1.139	1.736	1.626	1.603	1.565	1.218	0.604	82	78	71	13	-47	2.33E-6	1.66E-5	> 1.00E-4
NCI-H226	1.037	1.813	1.716	1.737	1.743	1.101	0.203	87	90	91	8	-80	3.13E-6	1.24E-5	4.54E-5
NCI-H23	0.591	1.949	1.887	1.892	1.692	0.672	0.106	95	96	81	6	-82	2.59E-6	1.17E-5	4.32E-5
NCI-H322M	0.811	2.290	2.103	2.076	1.887	1.239	0.419	87	86	73	29	-48	3.30E-6	2.37E-5	> 1.00E-4
NCI-H460	0.259	2.640	2.633	2.692	2.463	0.252	0.054	100	102	93	-3	-79	2.80E-6	9.37E-6	4.14E-5
NCI-H522	1.295	3.018	2.914	2.972	1.742	1.023	0.613	94	97	26	-21	-53	4.60E-7	3.57E-6	8.22E-5
Colon Cancer															
COLO 205	0.551	2.402	2.654	2.510	2.348	0.600	0.054	114	106	97	3	-90	3.15E-6	1.07E-5	3.69E-5
HCC-2998	0.668	2.648	2.452	2.492	2.336	0.659	0.081	90	92	84	-1	-88	2.51E-6	9.63E-6	3.64E-5
HCT-116	0.301	2.727	2.693	2.811	2.467	0.602	0.581	99	103	89	12	12	3.24E-6	> 1.00E-4	> 1.00E-4
HCT-15	0.311	2.558	2.537	2.519	2.121	0.379	0.049	99	98	81	3	-84	2.48E-6	1.08E-5	4.05E-5
HT29	0.217	1.552	1.541	1.625	1.505	0.151	0.034	99	105	96	-31	-85	2.32E-6	5.74E-6	2.29E-5
KM12	0.494	2.468	2.442	2.521	1.948	0.596	0.050	99	103	74	5	-90	2.22E-6	1.13E-5	3.80E-5
SW-620	0.268	2.027	1.996	1.954	1.717	0.760	0.057	98	96	82	28	-79	3.93E-6	1.83E-5	5.36E-5
CNS Cancer															
SF-268	1.072	2.841	2.720	2.765	2.443	1.308	0.702	93	96	77	13	-35	2.68E-6	1.90E-5	> 1.00E-4
SF-295	1.309	3.281	3.181	3.199	2.762	1.062	0.361	95	96	74	-19	-72	1.80E-6	6.25E-6	3.81E-5
SF-539	0.818	2.464	2.356	2.350	1.979	0.572	0.183	93	93	71	-30	-78	1.60E-6	5.03E-6	2.62E-5
SNB-19	0.564	1.998	1.893	1.919	1.745	0.766	0.191	93	95	82	14	-66	2.98E-6	1.50E-5	6.28E-5
SNB-75	1.773	2.794	2.475	2.523	2.125	1.726	0.911	69	73	34	-3	-49	3.99E-7	8.48E-6	> 1.00E-4
U251	0.353	1.742	1.644	1.701	1.532	0.455	0.039	93	97	85	7	-89	2.82E-6	1.19E-5	3.94E-5
Melanoma															
LOX IMVI	0.406	2.881	2.712	2.731	2.207	0.615	0.006	93	94	73	8	-99	2.26E-6	1.20E-5	3.52E-5
MALME-3M	0.616	1.690	1.572	1.611	1.330	1.027	0.116	89	93	66	38	-81	3.83E-6	2.09E-5	5.48E-5
M14	0.601	2.499	2.479	2.516	2.104	0.834	0.238	99	101	79	12	-60	2.73E-6	1.48E-5	7.19E-5
MDA-MB-435	0.676	2.976	2.895	2.927	1.275	0.481	0.157	96	98	26	-29	-77	4.64E-7	2.98E-6	2.76E-5
SK-MEL-2	0.850	1.936	1.890	1.920	1.654	0.963	0.245	96	99	74	10	-71	2.38E-6	1.34E-5	5.50E-5
SK-MEL-28	0.751	2.026	2.008	2.076	1.530	1.196	0.360	99	104	61	35	-52	2.65E-6	2.52E-5	9.45E-5
SK-MEL-5	0.904	3.244	2.919	3.021	2.632	0.307	0.001	86	90	74	-66	-100	1.48E-6	3.37E-6	7.68E-6
UACC-257	0.919	2.284	2.170	2.285	1.828	1.350	0.413	92	100	67	32	-55	2.97E-6	2.31E-5	8.74E-5
UACC-62	0.974	2.820	2.683	2.696	1.683	1.478	0.143	93	93	38	27	-85	6.14E-7	1.75E-5	4.85E-5
Ovarian Cancer															
IGROV1	0.436	2.077	1.940	1.976	1.524	0.650	0.049	92	94	66	13	-89	2.02E-6	1.34E-5	4.15E-5
OVCAR-3	0.626	1.986	1.931	1.942	1.769	0.668	0.101	96	97	84	3	-84	2.63E-6	1.09E-5	4.07E-5
OVCAR-4	0.715	1.919	1.857	1.890	1.688	1.099	0.631	95	98	81	32	-12	4.26E-6	5.38E-5	> 1.00E-4
OVCAR-5	0.447	1.600	1.638	1.619	1.497	0.614	0.015	103	102	91	14	-97	3.43E-6	1.35E-5	3.80E-5
OVCAR-8	0.337	1.912	1.782	1.963	1.735	0.307	0.103	92	103	89	-9	-70	2.49E-6	8.11E-6	4.76E-5
NCI/ADR-RES	0.357	1.353	1.312	1.357	0.830	0.166	0.107	96	100	48	-54	-70	8.97E-7	2.95E-6	9.20E-6
SK-OV-3	0.838	1.831	1.750	1.814	1.684	1.105	0.896	92	98	85	27	6	4.01E-6	> 1.00E-4	> 1.00E-4
Renal Cancer															
786-0	0.777	3.052	2.891	2.949	2.814	1.432	0.762	93	95	90	29	-2	4.48E-6	8.61E-5	> 1.00E-4
A498	1.209	2.193	2.083	2.138	1.965	1.733	0.171	89	94	77	53	-86	1.05E-5	2.41E-5	5.52E-5
ACHN	0.374	1.749	1.674	1.797	1.258	0.532	0.152	94	103	64	11	-59	1.86E-6	1.45E-5	7.38E-5
CAKI-1	0.495	1.770	1.656	1.692	1.123	0.833	0.191	91	94	49	26	-61	9.61E-7	2.00E-5	7.42E-5
RXF 393	0.750	1.441	1.410	1.429	1.201	0.739	0.297	96	98	65	-2	-60	1.69E-6	9.48E-6	6.64E-5
SN12C	0.631	2.207	2.014	2.129	1.958	0.776	0.021	88	95	84	9	-97	2.86E-6	1.22E-5	3.62E-5
TK-10	1.084	2.110	2.034	2.100	2.161	1.490	0.584	93	99	105	40	-46	6.92E-6	2.89E-5	> 1.00E-4
UO-31	0.664	2.085	1.820	1.851	1.465	0.931	0.410	81	83	56	19	-38	1.48E-6	2.13E-5	> 1.00E-4
Prostate Cancer															
PC-3	0.562	2.181	2.049	2.057	1.718	0.749	0.310	92	92	71	12	-45	2.28E-6	1.60E-5	> 1.00E-4
DU-145	0.375	1.649	1.644	1.650	1.583	0.487	0.033	100	100	95	9	-91	3.32E-6	1.22E-5	3.87E-5
Breast Cancer															
MCF7	0.450	2.394	2.188	2.245	1.642	0.473	0.240	89	92	61	1	-47	1.54E-6	1.06E-5	> 1.00E-4
MDA-MB-231/ATCC	0.566	1.142	1.130	1.146	1.106	0.507	0.078	98	101	94	-11	-86	2.63E-6	7.93E-6	3.32E-5
HS 578T	1.374	2.573	2.412	2.457	2.017	1.496	1.048	87	90	54	10	-24	1.21E-6	2.00E-5	> 1.00E-4
BT-549	1.398	2.810	2.784	2.689	2.522	1.748	0.888	98	91	80	25	-36	3.47E-6	2.54E-5	> 1.00E-4
T-47D	0.747	2.058	1.841	1.805	1.831	1.144	0.697	83	81	83	30	-7	4.20E-6	6.57E-5	> 1.00E-4
MDA-MB-468	0.773	1.481	1.456	1.465	1.262	0.615	0.210	96	98	69	-20	-73	1.63E-6	5.91E-6	3.66E-5

Compound **3j**

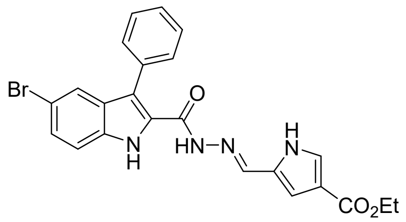

Br
O
HN–N
H
N
H
N
CO_2Et

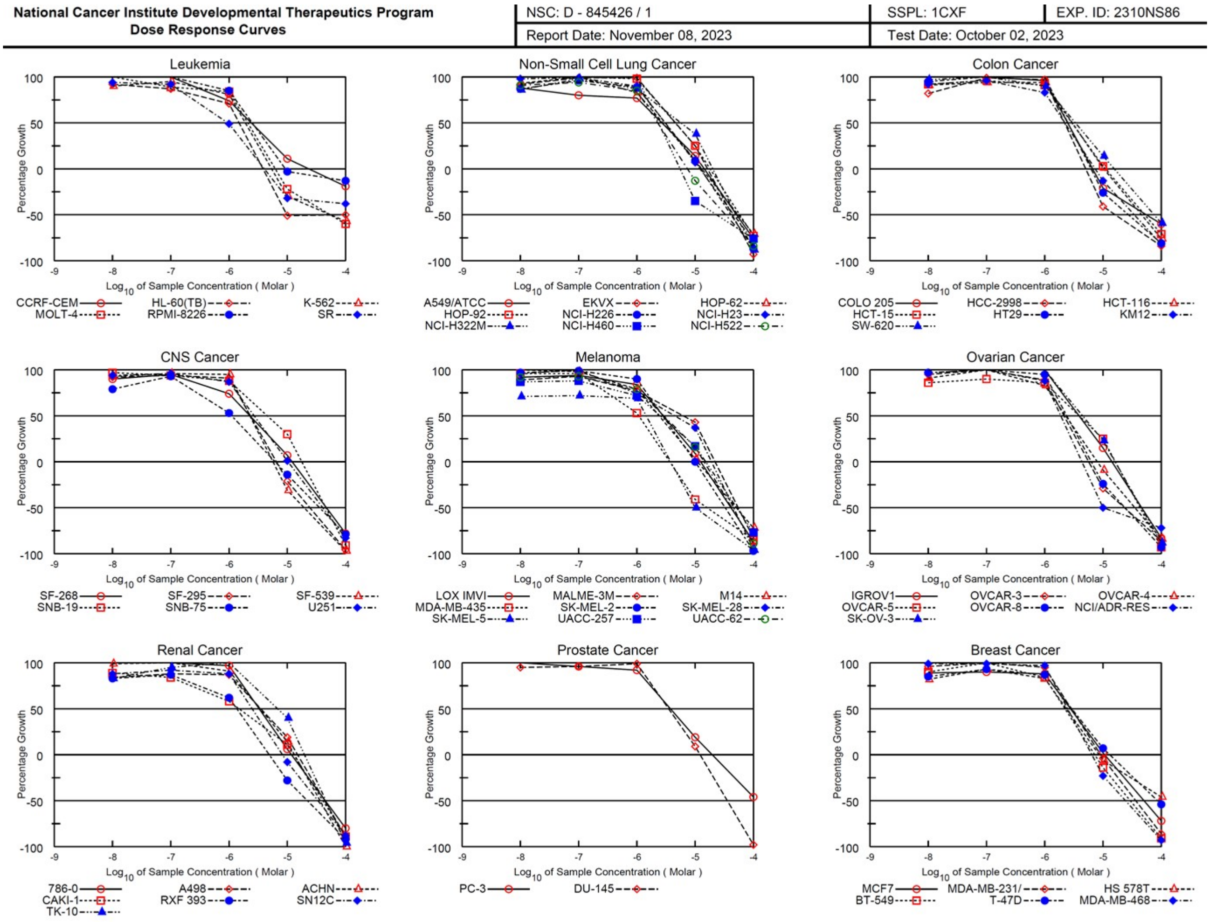

National Cancer Institute Developmental Therapeutics Program
Dose Response Curves
NSC: D - 845426 / 1
Report Date: November 08, 2023
SSPL: 1CXF
EXP. ID: 2310NS86
Test Date: October 02, 2023
Leukemia
Non-Small Cell Lung Cancer
Colon Cancer
CNS Cancer
Melanoma
Ovarian Cancer
Renal Cancer
Prostate Cancer
Breast Cancer
Percentage Growth
Log10 of Sample Concentration (Molar)
CCRF-CEM
HL-60(TB)
K-562
MOLT-4
RPMI-8226
SR
A549/ATCC
EKVX
HOP-62
HOP-92
NCI-H226
NCI-H23
NCI-H322M
NCI-H460
NCI-H522
COLO 205
HCC-2998
HCT-116
HCT-15
HT29
KM12
SW-620
SF-268
SF-295
SF-539
SNB-19
SNB-75
U251
LOX IMVI
MALME-3M
M14
MDA-MB-435
SK-MEL-2
SK-MEL-28
SK-MEL-5
UACC-257
UACC-62
IGROV1
OVCAR-3
OVCAR-4
OVCAR-5
OVCAR-8
NCI/ADR-RES
SK-OV-3
786-0
A498
ACHN
CAKI-1
RXF 393
SN12C
TK-10
PC-3
DU-145
MCF7
MDA-MB-231/
HS 578T
BT-549
T-47D
MDA-MB-468

National Cancer Institute Developmental Therapeutics Program
In-Vitro Testing Results

NSC : D - 845426 / 1	Experiment ID : 2310NS86	Test Type : 08	Units : Molar
Report Date : November 08, 2023	Test Date : October 02, 2023	QNS :	MC :
COMI : TO56	Stain Reagent : SRB Dual-Pass Related	SSPL : 1CXF	

			Log10 Concentration												
	Time		Mean Optical Densities					Percent Growth							
Panel/Cell Line	Zero	Ctrl	-8.0	-7.0	-6.0	-5.0	-4.0	-8.0	-7.0	-6.0	-5.0	-4.0	GI50	TGI	LC50
Leukemia															
CCRF-CEM	0.563	2.634	2.801	2.680	2.095	0.797	0.457	108	102	74	11	-19	2.41E-6	2.37E-5	> 1.00E-4
HL-60(TB)	0.776	2.697	2.553	2.438	2.147	0.383	0.387	92	87	71	-51	-50	1.50E-6	3.85E-6	9.88E-6
K-562	0.221	1.759	1.600	1.680	1.465	0.156	0.096	90	95	81	-30	-57	1.90E-6	5.39E-6	5.71E-5
MOLT-4	0.609	2.259	2.261	2.078	1.999	0.474	0.241	100	89	84	-22	-60	2.10E-6	6.19E-6	5.34E-5
RPMI-8226	0.518	2.021	2.067	2.029	1.801	0.503	0.450	103	101	85	-3	-13	2.52E-6	9.27E-6	> 1.00E-4
SR	0.364	1.688	1.609	1.580	1.013	0.248	0.225	94	92	49	-32	-38	9.48E-7	4.04E-6	> 1.00E-4
Non-Small Cell Lung Cancer															
A549/ATCC	0.333	1.992	1.796	1.668	1.616	0.564	0.082	88	80	77	14	-76	2.70E-6	1.43E-5	5.18E-5
EKVX	1.086	2.483	2.365	2.475	2.472	1.431	0.076	92	99	99	25	-93	4.57E-6	1.62E-5	4.31E-5
HOP-62	0.559	1.632	1.555	1.654	1.445	0.658	0.161	93	102	83	9	-71	2.78E-6	1.30E-5	5.44E-5
HOP-92	1.079	1.656	1.659	1.738	1.643	1.225	0.294	101	114	98	25	-73	4.56E-6	1.81E-5	5.86E-5
NCI-H226	1.191	2.315	2.171	2.273	2.195	1.288	0.161	87	96	89	9	-87	3.07E-6	1.23E-5	4.13E-5
NCI-H23	0.621	2.299	2.261	2.278	2.139	0.741	0.133	98	99	90	7	-79	3.06E-6	1.21E-5	4.64E-5
NCI-H322M	0.862	2.098	1.928	2.069	1.941	1.327	0.106	86	98	87	38	-88	5.63E-6	2.00E-5	5.00E-5
NCI-H460	0.359	2.288	2.293	2.415	2.343	0.232	0.088	100	107	103	-35	-76	2.41E-6	5.55E-6	2.31E-5
NCI-H522	1.404	3.169	3.014	3.070	2.907	1.217	0.225	91	94	85	-13	-84	2.27E-6	7.32E-6	3.30E-5
Colon Cancer															
COLO 205	0.503	1.916	1.990	2.062	1.867	0.398	0.201	105	110	96	-21	-60	2.49E-6	6.64E-6	5.52E-5
HCC-2998	0.853	3.101	2.692	3.081	3.029	0.500	0.137	82	99	97	-41	-84	2.18E-6	5.02E-6	1.59E-5
HCT-116	0.349	2.690	2.490	2.546	2.545	0.364	0.083	91	94	94	1	-76	2.95E-6	1.02E-5	4.56E-5
HCT-15	0.382	2.806	2.609	2.714	2.587	0.460	0.110	92	96	91	3	-71	2.93E-6	1.10E-5	5.19E-5
HT29	0.257	1.628	1.566	1.643	1.674	0.191	0.050	95	101	103	-26	-81	2.59E-6	6.31E-6	2.75E-5
KM12	0.611	2.536	2.386	2.454	2.217	0.529	0.107	92	96	83	-13	-82	2.21E-6	7.27E-6	3.39E-5
SW-620	0.402	1.887	1.843	2.055	1.734	0.614	0.167	97	111	90	14	-59	3.36E-6	1.57E-5	7.62E-5
CNS Cancer															
SF-268	0.763	2.102	1.973	2.033	1.759	0.851	0.165	90	95	74	7	-78	2.29E-6	1.19E-5	4.63E-5
SF-295	1.288	3.174	3.021	3.059	3.012	1.018	0.054	92	94	91	-21	-96	2.34E-6	6.51E-6	2.44E-5
SF-539	0.714	2.364	2.245	2.303	2.274	0.492	0.024	93	96	95	-31	-97	2.26E-6	5.66E-6	1.94E-5
SNB-19	0.850	2.289	2.247	2.210	2.116	1.281	0.080	97	94	88	30	-91	4.51E-6	1.77E-5	4.61E-5
SNB-75	0.927	1.323	1.241	1.294	1.135	0.796	0.196	79	93	53	-14	-79	1.09E-6	6.13E-6	3.58E-5
U251	0.268	1.411	1.348	1.360	1.257	0.276	0.047	94	96	87	1	-83	2.66E-6	1.02E-5	4.06E-5
Melanoma															
LOX IMVI	0.464	2.625	2.449	2.506	2.278	0.650	0.060	92	94	84	9	-87	2.82E-6	1.23E-5	4.09E-5
MALME-3M	0.739	1.616	1.519	1.557	1.411	1.113	0.129	89	93	77	43	-83	6.06E-6	2.19E-5	5.49E-5
M14	0.494	1.745	1.749	1.718	1.480	0.527	0.137	100	98	79	3	-72	2.39E-6	1.08E-5	5.03E-5
MDA-MB-435	0.751	2.235	2.170	2.180	1.544	0.444	0.114	96	96	53	-41	-85	1.09E-6	3.68E-6	1.61E-5
SK-MEL-2	1.244	2.506	2.474	2.489	2.382	1.247	0.036	97	99	90	0	-97	2.80E-6	1.01E-5	3.28E-5
SK-MEL-28	0.803	2.344	2.290	2.359	1.949	1.380	0.042	96	101	74	37	-95	4.56E-6	1.92E-5	4.58E-5
SK-MEL-5	1.062	3.058	2.481	2.509	2.434	0.534	0.045	71	72	69	-50	-96	1.44E-6	3.80E-6	1.01E-5
UACC-257	1.089	2.575	2.389	2.395	2.162	1.342	0.253	87	88	72	17	-77	2.53E-6	1.52E-5	5.18E-5
UACC-62	1.130	3.011	2.856	2.888	2.624	1.434	0.125	92	93	79	16	-89	2.92E-6	1.42E-5	4.26E-5
Ovarian Cancer															
IGROV1	0.598	1.926	2.126	1.940	1.939	0.795	0.103	115	101	101	15	-83	3.91E-6	1.42E-5	4.61E-5
OVCAR-3	0.648	1.874	1.815	1.957	1.736	0.460	0.088	95	107	89	-29	-86	2.13E-6	5.66E-6	2.31E-5
OVCAR-4	0.950	2.034	1.935	2.036	1.852	0.861	0.149	91	100	83	-9	-84	2.28E-6	7.91E-6	3.48E-5
OVCAR-5	0.593	1.544	1.408	1.453	1.410	0.829	0.039	86	90	86	25	-93	3.87E-6	1.62E-5	4.29E-5
OVCAR-8	0.526	2.370	2.315	2.379	2.271	0.398	0.039	97	101	95	-24	-93	2.37E-6	6.23E-6	2.37E-5
NCI/ADR-RES	0.585	2.074	2.026	2.086	1.896	0.295	0.164	97	101	88	-50	-72	1.89E-6	4.36E-6	1.04E-5
SK-OV-3	0.720	1.567	1.596	1.607	1.577	0.916	0.085	103	105	101	23	-88	4.53E-6	1.61E-5	4.53E-5
Renal Cancer															
786-0	0.686	2.574	2.569	2.582	2.511	0.807	0.135	100	100	97	6	-80	3.29E-6	1.18E-5	4.47E-5
A498	1.481	2.247	2.121	2.154	2.147	1.630	0.156	84	88	87	19	-89	3.53E-6	1.51E-5	4.34E-5
ACHN	0.349	1.729	1.719	1.812	1.602	0.540	-0.001	99	106	91	14	-100	3.39E-6	1.32E-5	3.64E-5
CAKI-1	0.581	1.985	1.827	1.763	1.392	0.733	0.062	89	84	58	11	-89	1.46E-6	1.28E-5	4.05E-5
RXF 393	1.053	1.825	1.696	1.725	1.533	0.754	0.090	83	87	62	-28	-92	1.36E-6	4.86E-6	2.20E-5
SN12C	0.543	2.056	1.875	1.935	1.878	0.499	0.064	88	92	88	-8	-88	2.49E-6	8.24E-6	3.33E-5
TK-10	1.244	2.057	1.921	2.017	2.161	1.572	0.056	83	95	113	40	-96	7.35E-6	1.98E-5	4.62E-5
Prostate Cancer															
PC-3	0.515	2.002	2.008	1.936	1.881	0.799	0.281	100	96	92	19	-46	3.76E-6	1.97E-5	> 1.00E-4
DU-145	0.361	1.577	1.521	1.526	1.559	0.469	0.008	95	96	99	9	-98	3.48E-6	1.21E-5	3.56E-5
Breast Cancer															
MCF7	0.544	2.461	2.250	2.267	2.230	0.577	0.152	89	90	88	2	-72	2.76E-6	1.06E-5	5.02E-5
MDA-MB-231/ATCC	0.605	1.235	1.212	1.246	1.202	0.583	0.087	96	102	95	-4	-86	2.85E-6	9.18E-6	3.68E-5
HS 578T	1.372	2.322	2.152	2.256	2.163	1.293	0.737	82	93	83	-6	-46	2.36E-6	8.61E-6	> 1.00E-4
BT-549	1.308	2.211	2.122	2.342	2.067	1.127	0.120	90	114	84	-14	-91	2.23E-6	7.22E-6	2.95E-5
T-47D	0.798	1.803	1.655	1.736	1.677	0.866	0.370	85	93	87	7	-54	2.91E-6	1.29E-5	8.69E-5
MDA-MB-468	1.202	2.268	2.256	2.261	2.239	0.923	0.084	99	99	97	-23	-93	2.47E-6	6.42E-6	2.42E-5

Compound **3n**

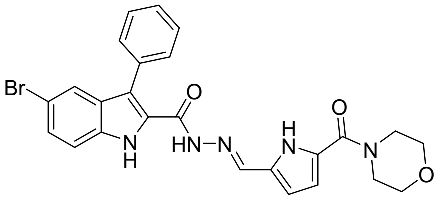

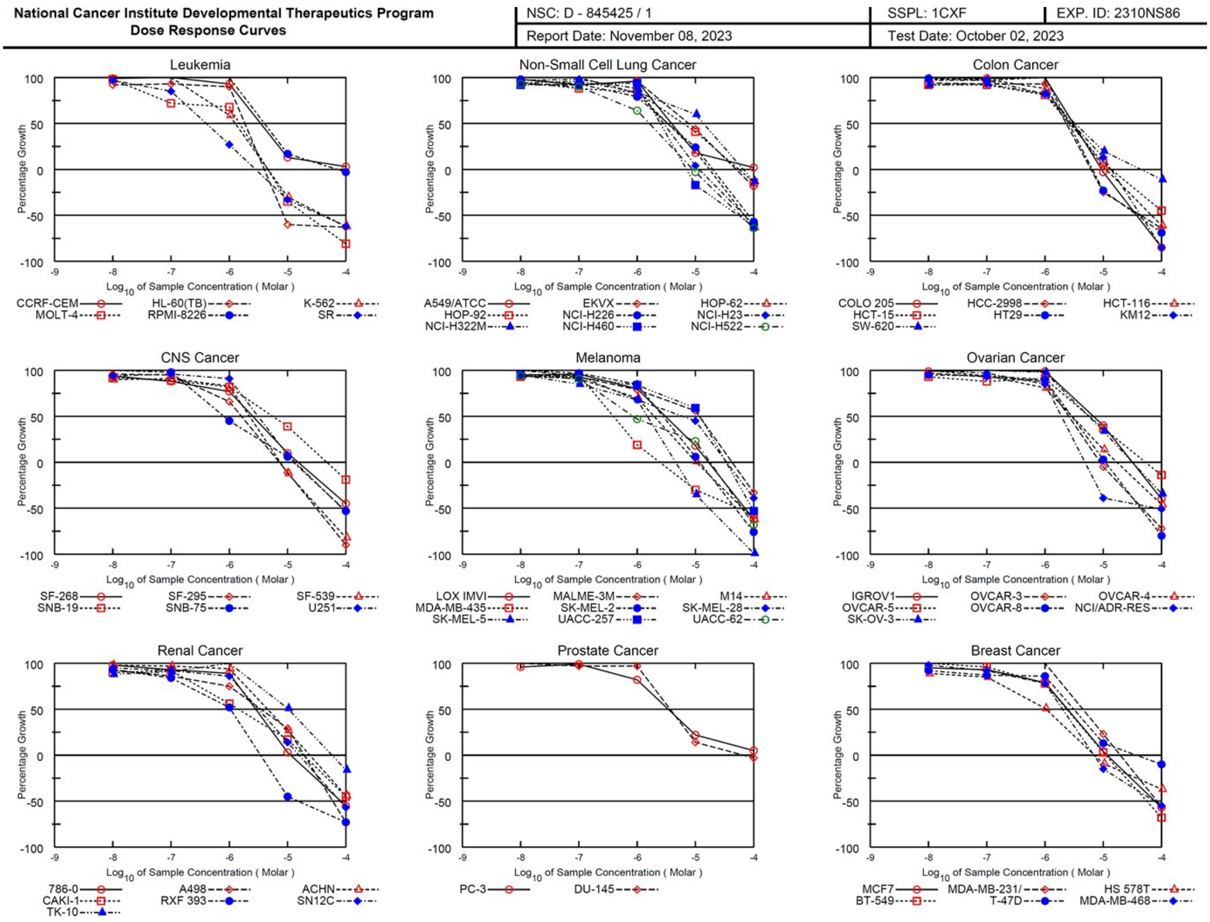
National Cancer Institute Developmental Therapeutics Program
Dose Response Curves
NSC: D - 845425 / 1
Report Date: November 08, 2023
SSPL: 1CXF
Test Date: October 02, 2023
EXP. ID: 2310NS86
Leukemia
Non-Small Cell Lung Cancer
Colon Cancer
CNS Cancer
Melanoma
Ovarian Cancer
Renal Cancer
Prostate Cancer
Breast Cancer
Percentage Growth
Log10 of Sample Concentration (Molar)
CCRF-CEM
HL-60(TB)
K-562
MOLT-4
RPMI-8226
SR
A549/ATCC
EKVX
HOP-62
HOP-92
NCI-H226
NCI-H23
NCI-H322M
NCI-H460
NCI-H522
COLO 205
HCC-2998
HCT-116
HCT-15
HT29
KM12
SW-620
SF-268
SF-295
SF-539
SNB-19
SNB-75
U251
LOX IMVI
MALME-3M
M14
MDA-MB-435
SK-MEL-2
SK-MEL-28
SK-MEL-5
UACC-257
UACC-62
IGROV1
OVCAR-3
OVCAR-4
OVCAR-5
OVCAR-8
NCI/ADR-RES
SK-OV-3
786-0
A498
ACHN
CAKI-1
RXF 393
SN12C
TK-10
PC-3
DU-145
MCF7
MDA-MB-231/
HS 578T
BT-549
T-47D
MDA-MB-468

National Cancer Institute Developmental Therapeutics Program
In-Vitro Testing Results

NSC : D - 845425 / 1	Experiment ID : 2310NS86	Test Type : 08	Units : Molar
Report Date : November 08, 2023	Test Date : October 02, 2023	QNS :	MC :
COMI : TO54	Stain Reagent : SRB Dual-Pass Related	SSPL : 1CXF	

			Log10 Concentration												
	Time		Mean Optical Densities					Percent Growth							
Panel/Cell Line	Zero	Ctrl	-8.0	-7.0	-6.0	-5.0	-4.0	-8.0	-7.0	-6.0	-5.0	-4.0	GI50	TGI	LC50
Leukemia															
CCRF-CEM	0.563	2.580	2.569	2.779	2.436	0.828	0.629	99	110	93	13	3	3.45E-6	> 1.00E-4	> 1.00E-4
HL-60(TB)	0.776	2.567	2.427	2.438	2.380	0.310	0.286	92	93	90	-60	-63	1.84E-6	3.97E-6	8.56E-6
K-562	0.221	1.286	1.399	1.390	0.854	0.154	0.085	111	110	59	-30	-62	1.27E-6	4.60E-6	4.27E-5
MOLT-4	0.609	2.053	2.031	1.653	1.594	0.393	0.114	98	72	68	-35	-81	1.50E-6	4.55E-6	2.08E-5
RPMI-8226	0.518	1.951	2.110	1.957	2.114	0.769	0.505	111	100	111	17	-3	4.50E-6	7.49E-5	> 1.00E-4
SR	0.364	1.347	1.314	1.199	0.633	0.244	0.139	97	85	27	-33	-62	4.05E-7	2.84E-6	3.89E-5
Non-Small Cell Lung Cancer															
A549/ATCC	0.333	1.903	1.865	1.779	1.841	0.614	0.358	98	92	96	18	2	3.88E-6	> 1.00E-4	> 1.00E-4
EKVX	1.086	2.487	2.392	2.371	2.425	1.696	0.882	93	92	96	44	-19	7.51E-6	4.99E-5	> 1.00E-4
HOP-62	0.559	1.541	1.572	1.563	1.377	0.757	0.205	103	102	83	20	-63	3.36E-6	1.74E-5	6.92E-5
HOP-92	1.079	1.763	1.728	1.680	1.651	1.360	0.929	95	88	84	41	-14	6.14E-6	5.57E-5	> 1.00E-4
NCI-H226	1.191	2.326	2.301	2.251	2.091	1.464	0.511	98	93	79	24	-57	3.39E-6	1.98E-5	8.17E-5
NCI-H23	0.621	2.296	2.250	2.257	2.097	0.686	0.249	97	98	88	4	-60	2.83E-6	1.15E-5	6.98E-5
NCI-H322M	0.862	2.331	2.236	2.274	2.099	1.749	0.754	94	96	84	60	-13	1.39E-5	6.73E-5	> 1.00E-4
NCI-H460	0.359	2.328	2.167	2.164	2.217	0.297	0.137	92	92	94	-17	-62	2.49E-6	6.99E-6	5.41E-5
NCI-H522	1.404	2.894	2.796	2.751	2.354	1.355	0.507	93	90	64	-3	-64	1.60E-6	8.87E-6	5.88E-5
Colon Cancer															
COLO 205	0.503	1.914	1.943	1.900	1.940	0.487	0.077	102	99	102	-3	-85	3.12E-6	9.33E-6	3.75E-5
HCC-2998	0.853	3.119	2.988	2.953	2.956	0.639	0.304	94	93	93	-25	-64	2.31E-6	6.12E-6	4.29E-5
HCT-116	0.349	2.741	2.695	2.692	2.446	0.421	0.135	98	98	88	3	-61	2.79E-6	1.11E-5	6.67E-5
HCT-15	0.382	2.735	2.557	2.537	2.287	0.543	0.210	92	92	81	7	-45	2.62E-6	1.35E-5	> 1.00E-4
HT29	0.257	1.694	1.676	1.658	1.702	0.199	0.079	99	97	101	-23	-69	2.57E-6	6.54E-6	3.83E-5
KM12	0.611	2.688	2.618	2.622	2.315	0.880	0.089	97	97	82	13	-85	2.91E-6	1.35E-5	4.36E-5
SW-620	0.402	1.952	1.835	1.844	1.670	0.710	0.360	92	93	82	20	-11	3.26E-6	4.50E-5	> 1.00E-4
CNS Cancer															
SF-268	0.763	2.115	2.029	1.952	1.803	0.894	0.421	94	88	77	10	-45	2.51E-6	1.50E-5	> 1.00E-4
SF-295	1.288	3.130	3.053	3.040	2.511	1.143	0.129	96	95	66	-11	-90	1.63E-6	7.16E-6	3.10E-5
SF-539	0.714	2.410	2.247	2.250	2.123	0.630	0.128	90	91	83	-12	-82	2.23E-6	7.50E-6	3.49E-5
SNB-19	0.850	2.368	2.243	2.209	2.097	1.449	0.687	92	89	82	39	-19	5.66E-6	4.71E-5	> 1.00E-4
SNB-75	0.927	1.458	1.456	1.448	1.167	0.961	0.436	100	98	45	6	-53	8.09E-7	1.28E-5	8.89E-5
U251	0.268	1.411	1.347	1.366	1.308	0.355	0.122	94	96	91	8	-54	3.10E-6	1.33E-5	8.47E-5
Melanoma															
LOX IMVI	0.464	2.633	2.535	2.550	2.204	0.854	0.178	95	96	80	18	-62	3.06E-6	1.68E-5	7.12E-5
MALME-3M	0.739	1.671	1.638	1.608	1.473	1.262	0.498	96	93	79	56	-33	1.17E-5	4.29E-5	> 1.00E-4
M14	0.494	1.782	1.710	1.660	1.391	0.502	0.188	94	91	70	1	-62	1.92E-6	1.02E-5	6.42E-5
MDA-MB-435	0.751	2.341	2.236	2.258	1.059	0.527	0.324	93	95	19	-30	-57	3.92E-7	2.47E-6	5.54E-5
SK-MEL-2	1.244	2.432	2.438	2.392	2.257	1.319	0.299	101	97	85	6	-76	2.80E-6	1.19E-5	4.83E-5
SK-MEL-28	0.803	2.295	2.320	2.212	1.825	1.474	0.494	102	94	68	45	-39	6.10E-6	3.45E-5	> 1.00E-4
SK-MEL-5	1.062	3.211	3.097	2.887	2.516	0.687	0.010	95	85	68	-35	-99	1.48E-6	4.54E-6	1.70E-5
UACC-257	1.089	2.637	2.563	2.519	2.384	1.996	0.514	95	92	84	59	-53	1.19E-5	3.36E-5	9.44E-5
UACC-62	1.130	3.072	2.950	2.890	2.048	1.582	0.363	94	91	47	23	-68	8.64E-7	1.80E-5	6.37E-5
Ovarian Cancer															
IGROV1	0.598	2.066	2.049	2.191	2.059	1.179	0.359	99	109	99	40	-40	6.69E-6	3.14E-5	> 1.00E-4
OVCAR-3	0.648	1.918	1.882	1.832	1.767	0.618	0.183	97	93	88	-5	-72	2.57E-6	8.90E-6	4.74E-5
OVCAR-4	0.950	2.156	2.152	2.086	1.931	1.122	0.516	100	94	81	14	-46	2.93E-6	1.73E-5	> 1.00E-4
OVCAR-5	0.593	1.464	1.400	1.363	1.382	0.918	0.511	93	88	91	37	-14	5.78E-6	5.37E-5	> 1.00E-4
OVCAR-8	0.526	2.524	2.428	2.410	2.319	0.577	0.104	95	94	90	3	-80	2.86E-6	1.07E-5	4.31E-5
NCI/ADR-RES	0.585	2.053	2.047	2.008	1.838	0.354	0.288	100	97	85	-39	-51	1.92E-6	4.83E-6	8.41E-5
SK-OV-3	0.720	1.622	1.692	1.701	1.602	1.031	0.473	108	109	98	34	-34	5.68E-6	3.17E-5	> 1.00E-4
Renal Cancer															
786-0	0.686	2.622	2.576	2.490	2.414	0.747	0.306	98	93	89	3	-55	2.86E-6	1.13E-5	8.07E-5
A498	1.481	2.269	2.216	2.158	2.071	1.713	0.403	93	86	75	29	-73	3.53E-6	1.94E-5	5.99E-5
ACHN	0.349	1.700	1.679	1.654	1.622	0.715	0.194	98	97	94	27	-44	4.56E-6	2.39E-5	> 1.00E-4
CAKI-1	0.581	2.043	1.903	1.933	1.406	0.831	0.318	90	92	56	17	-45	1.46E-6	1.88E-5	> 1.00E-4
RXF 393	1.053	1.744	1.693	1.633	1.415	0.575	0.285	93	84	52	-45	-73	1.06E-6	3.43E-6	1.47E-5
SN12C	0.543	2.017	1.948	1.902	1.804	0.749	0.234	95	92	86	14	-57	3.14E-6	1.57E-5	7.97E-5
TK-10	1.244	2.111	2.008	2.035	2.161	1.688	1.044	88	91	106	51	-16	1.04E-5	5.76E-5	> 1.00E-4
Prostate Cancer															
PC-3	0.515	2.026	1.965	2.011	1.760	0.849	0.584	96	99	82	22	5	3.44E-6	> 1.00E-4	> 1.00E-4
DU-145	0.361	1.568	1.594	1.530	1.538	0.533	0.351	102	97	97	14	-3	3.72E-6	6.87E-5	> 1.00E-4
Breast Cancer															
MCF7	0.544	2.358	2.268	2.229	1.983	0.620	0.236	95	93	79	4	-57	2.46E-6	1.17E-5	7.78E-5
MDA-MB-231/ATCC	0.605	1.214	1.242	1.221	1.255	0.748	0.264	105	101	107	23	-56	4.81E-6	1.97E-5	8.32E-5
HS 578T	1.372	2.393	2.286	2.239	1.892	1.254	0.867	89	85	51	-9	-37	1.04E-6	7.16E-6	> 1.00E-4
BT-549	1.308	2.255	2.364	2.219	2.047	1.334	0.414	112	96	78	3	-68	2.36E-6	1.09E-5	5.52E-5
T-47D	0.798	1.842	1.758	1.707	1.692	0.935	0.718	92	87	86	13	-10	3.10E-6	3.69E-5	> 1.00E-4
MDA-MB-468	1.202	2.316	2.296	2.229	2.066	1.022	0.543	98	92	78	-15	-55	1.98E-6	6.89E-6	7.55E-5

Compound **3p**

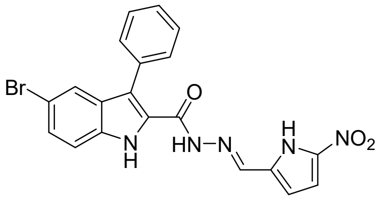
Br
O
HN–N
H
N
NO2
N
H

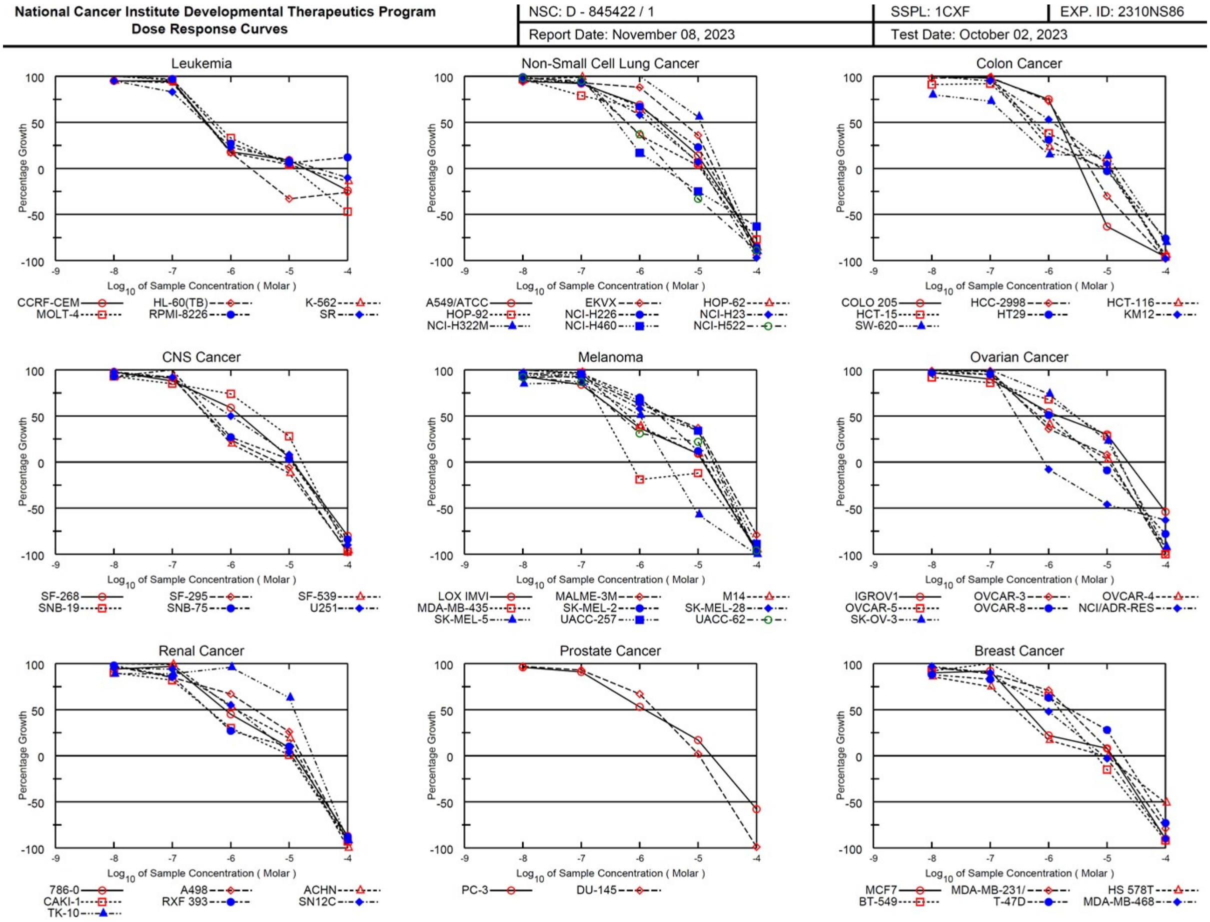
National Cancer Institute Developmental Therapeutics Program
Dose Response Curves
NSC: D - 845422 / 1
Report Date: November 08, 2023
SSPL: 1CXF
EXP. ID: 2310NS86
Test Date: October 02, 2023
Leukemia
CCRF-CEM HL-60(TB) K-562 MOLT-4 RPMI-8226 SR
Non-Small Cell Lung Cancer
A549/ATCC EKVX HOP-62 HOP-92 NCI-H226 NCI-H23 NCI-H322M NCI-H460 NCI-H522
Colon Cancer
COLO 205 HCC-2998 HCT-116 HCT-15 HT29 KM12 SW-620
CNS Cancer
SF-268 SF-295 SF-539 SNB-19 SNB-75 U251
Melanoma
LOX IMVI MALME-3M M14 MDA-MB-435 SK-MEL-2 SK-MEL-28 SK-MEL-5 UACC-257 UACC-62
Ovarian Cancer
IGROV1 OVCAR-3 OVCAR-4 OVCAR-5 OVCAR-8 NCI/ADR-RES SK-OV-3
Renal Cancer
786-0 A498 ACHN CAKI-1 RXF 393 SN12C TK-10
Prostate Cancer
PC-3 DU-145
Breast Cancer
MCF7 MDA-MB-231/ HS 578T BT-549 T-47D MDA-MB-468
Percentage Growth
Log10 of Sample Concentration (Molar)

National Cancer Institute Developmental Therapeutics Program
In-Vitro Testing Results

NSC : D - 845422 / 1	Experiment ID : 2310NS86	Test Type : 08	Units : Molar
Report Date : November 08, 2023	Test Date : October 02, 2023	QNS :	MC :
COMI : TO51	Stain Reagent : SRB Dual-Pass Related	SSPL : 1CXF	

			Log10 Concentration												
	Time		Mean Optical Densities					Percent Growth							
Panel/Cell Line	Zero	Ctrl	-8.0	-7.0	-6.0	-5.0	-4.0	-8.0	-7.0	-6.0	-5.0	-4.0	GI50	TGI	LC50
**Leukemia**															
CCRF-CEM	0.563	2.758	2.656	2.649	0.950	0.755	0.429	95	95	18	9	-24	3.82E-7	1.86E-5	> 1.00E-4
HL-60(TB)	0.776	3.017	2.906	2.881	1.164	0.523	0.572	95	94	17	-33	-26	3.74E-7	2.22E-6	> 1.00E-4
K-562	0.221	1.956	1.874	1.830	0.511	0.291	0.191	95	93	17	4	-14	3.65E-7	1.69E-5	> 1.00E-4
MOLT-4	0.609	2.570	2.626	2.488	1.258	0.680	0.322	103	96	33	4	-47	5.37E-7	1.18E-5	> 1.00E-4
RPMI-8226	0.518	2.017	2.052	1.979	0.918	0.610	0.704	102	97	27	6	12	4.68E-7	> 1.00E-4	> 1.00E-4
SR	0.364	1.925	1.849	1.656	0.724	0.502	0.328	95	83	23	9	-10	3.54E-7	2.94E-5	> 1.00E-4
**Non-Small Cell Lung Cancer**															
A549/ATCC	0.333	1.973	1.891	1.847	1.470	0.561	0.053	95	92	69	14	-84	2.23E-6	1.39E-5	4.49E-5
EKVX	1.086	2.585	2.500	2.477	2.401	1.627	0.062	94	93	88	36	-94	5.37E-6	1.89E-5	4.57E-5
HOP-62	0.559	1.486	1.458	1.473	0.897	0.590	0.078	97	99	36	3	-86	6.06E-7	1.09E-5	3.95E-5
HOP-92	1.079	1.806	1.784	1.650	1.550	1.126	0.247	97	79	65	6	-77	1.79E-6	1.19E-5	4.73E-5
NCI-H226	1.191	2.375	2.361	2.288	1.984	1.462	0.171	99	93	67	23	-86	2.43E-6	1.62E-5	4.69E-5
NCI-H23	0.621	2.395	2.322	2.266	1.654	0.748	0.019	96	93	58	7	-97	1.45E-6	1.17E-5	3.54E-5
NCI-H322M	0.862	2.192	2.223	2.487	2.320	1.607	0.088	102	122	110	56	-90	1.10E-5	2.42E-5	5.33E-5
NCI-H460	0.359	2.328	2.414	2.425	0.692	0.268	0.135	104	105	17	-25	-63	4.21E-7	2.51E-6	4.59E-5
NCI-H522	1.404	3.221	3.183	3.124	2.070	0.935	0.153	98	95	37	-33	-89	5.88E-7	3.33E-6	1.98E-5
**Colon Cancer**															
COLO 205	0.503	1.750	1.798	1.724	1.438	0.188	0.021	104	98	75	-63	-96	1.52E-6	3.50E-6	8.08E-6
HCC-2998	0.853	3.103	3.055	3.076	2.496	0.595	0.018	98	99	73	-30	-98	1.67E-6	5.09E-6	1.96E-5
HCT-116	0.349	2.429	2.457	2.391	0.831	0.346	0.022	101	98	23	0	-94	4.39E-7	9.21E-6	3.38E-5
HCT-15	0.382	2.828	2.600	2.626	1.323	0.544	0.016	91	92	38	7	-96	6.07E-7	1.16E-5	3.57E-5
HT29	0.257	1.855	1.858	1.901	0.760	0.250	0.061	100	103	31	-3	-76	5.50E-7	8.32E-6	4.39E-5
KM12	0.611	2.750	2.850	2.650	1.749	0.728	0.011	105	95	53	5	-98	1.17E-6	1.13E-5	3.42E-5
SW-620	0.402	2.243	1.879	1.743	0.682	0.665	0.082	80	73	15	14	-80	2.49E-7	1.42E-5	4.84E-5
**CNS Cancer**															
SF-268	0.763	2.193	2.158	2.026	1.608	0.831	0.152	98	88	59	5	-80	1.47E-6	1.14E-5	4.42E-5
SF-295	1.288	3.156	3.093	2.993	1.741	1.205	0.027	97	91	24	-6	-98	4.13E-7	6.15E-6	2.99E-5
SF-539	0.714	2.368	2.248	2.249	1.049	0.625	0.028	93	93	20	-12	-96	3.89E-7	4.16E-6	2.81E-5
SNB-19	0.850	2.354	2.248	2.125	1.961	1.275	0.030	93	85	74	28	-97	3.34E-6	1.68E-5	4.24E-5
SNB-75	0.927	1.395	1.363	1.393	1.053	0.942	0.145	93	100	27	3	-84	4.82E-7	1.09E-5	4.05E-5
U251	0.268	1.457	1.434	1.367	0.860	0.369	0.027	98	92	50	8	-90	9.87E-7	1.22E-5	3.92E-5
**Melanoma**															
LOX IMVI	0.464	2.822	2.647	2.441	1.325	0.670	0.013	93	84	37	9	-97	5.19E-7	1.21E-5	3.58E-5
MALME-3M	0.739	1.684	1.611	1.621	1.336	1.092	0.154	92	93	63	37	-79	3.23E-6	2.09E-5	5.62E-5
M14	0.494	1.647	1.601	1.613	0.947	0.594	0.027	96	97	39	9	-95	6.52E-7	1.21E-5	3.70E-5
MDA-MB-435	0.751	2.257	2.271	2.208	0.606	0.663	0.057	101	97	-19	-12	-92	2.53E-7	6.81E-7	2.98E-5
SK-MEL-2	1.244	2.524	2.563	2.475	2.137	1.393	0.026	103	96	70	12	-98	2.18E-6	1.28E-5	3.65E-5
SK-MEL-28	0.803	2.340	2.289	2.199	1.701	1.331	0.019	97	91	58	34	-98	2.24E-6	1.82E-5	4.35E-5
SK-MEL-5	1.062	3.258	2.937	2.946	2.176	0.462	0.004	85	86	51	-57	-100	1.02E-6	2.97E-6	8.69E-6
UACC-257	1.089	2.733	2.638	2.654	2.171	1.645	0.122	94	95	66	34	-89	3.12E-6	1.89E-5	4.82E-5
UACC-62	1.130	3.075	2.922	2.820	1.724	1.562	0.048	92	87	31	22	-96	4.51E-7	1.54E-5	4.09E-5
**Ovarian Cancer**															
IGROV1	0.598	2.205	2.158	2.049	1.468	1.081	0.273	97	90	54	30	-54	1.48E-6	2.27E-5	8.88E-5
OVCAR-3	0.648	1.970	1.952	1.963	1.124	0.756	0.006	99	99	36	8	-99	6.02E-7	1.19E-5	3.48E-5
OVCAR-4	0.950	2.012	2.027	1.958	1.385	0.982	0.071	101	95	41	3	-93	6.79E-7	1.07E-5	3.59E-5
OVCAR-5	0.593	1.433	1.366	1.313	1.168	0.830	0.003	92	86	68	28	-100	2.87E-6	1.66E-5	4.09E-5
OVCAR-8	0.526	2.558	2.488	2.448	1.555	0.477	0.116	97	95	51	-9	-78	1.02E-6	6.97E-6	3.90E-5
NCI/ADR-RES	0.585	2.176	2.172	2.144	0.541	0.313	0.215	100	98	-8	-46	-63	2.85E-7	8.49E-7	1.62E-5
SK-OV-3	0.720	1.462	1.556	1.488	1.270	0.889	0.058	113	103	74	23	-92	2.95E-6	1.58E-5	4.30E-5
**Renal Cancer**															
786-0	0.686	2.451	2.353	2.393	1.482	0.850	0.090	94	97	45	9	-87	8.03E-7	1.25E-5	4.14E-5
A498	1.481	2.327	2.302	2.200	2.048	1.701	0.108	97	85	67	26	-93	2.60E-6	1.66E-5	4.37E-5
ACHN	0.349	1.710	1.779	1.694	1.077	0.607	-0.001	105	99	53	19	-100	1.26E-6	1.44E-5	3.80E-5
CAKI-1	0.581	2.010	1.869	1.750	1.010	0.597	0.040	90	82	30	1	-93	4.11E-7	1.03E-5	3.49E-5
RXF 393	1.053	1.801	1.789	1.697	1.258	1.130	0.129	98	86	27	10	-88	4.11E-7	1.27E-5	4.12E-5
SN12C	0.543	2.105	2.032	2.018	1.399	0.601	0.047	95	94	55	4	-91	1.24E-6	1.09E-5	3.67E-5
TK-10	1.244	2.251	2.137	2.139	2.209	1.882	0.102	89	89	96	63	-92	1.22E-5	2.56E-5	5.38E-5
**Prostate Cancer**															
PC-3	0.515	2.183	2.122	2.033	1.398	0.795	0.217	96	91	53	17	-58	1.20E-6	1.68E-5	7.85E-5
DU-145	0.361	1.801	1.758	1.697	1.333	0.383	0.003	97	93	67	2	-99	1.84E-6	1.04E-5	3.25E-5
**Breast Cancer**															
MCF7	0.544	2.513	2.311	2.349	0.972	0.710	0.052	90	92	22	8	-90	3.94E-7	1.22E-5	3.90E-5
MDA-MB-231/ATCC	0.605	1.261	1.237	1.190	1.070	0.656	0.129	96	89	71	8	-79	2.14E-6	1.23E-5	4.66E-5
HS 578T	1.372	2.418	2.276	2.161	1.546	1.355	0.669	86	75	17	-1	-51	2.70E-7	8.49E-6	9.43E-5
BT-549	1.308	2.241	2.167	2.237	1.910	1.106	0.109	92	100	65	-15	-92	1.52E-6	6.41E-6	2.84E-5
T-47D	0.798	1.703	1.590	1.550	1.371	1.054	0.218	88	83	63	28	-73	2.40E-6	1.90E-5	5.96E-5
MDA-MB-468	1.202	2.446	2.403	2.318	1.793	1.163	0.124	97	90	48	-3	-90	8.73E-7	8.63E-6	3.47E-5

Compound **3q**

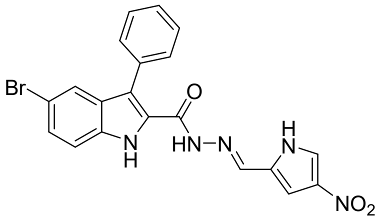

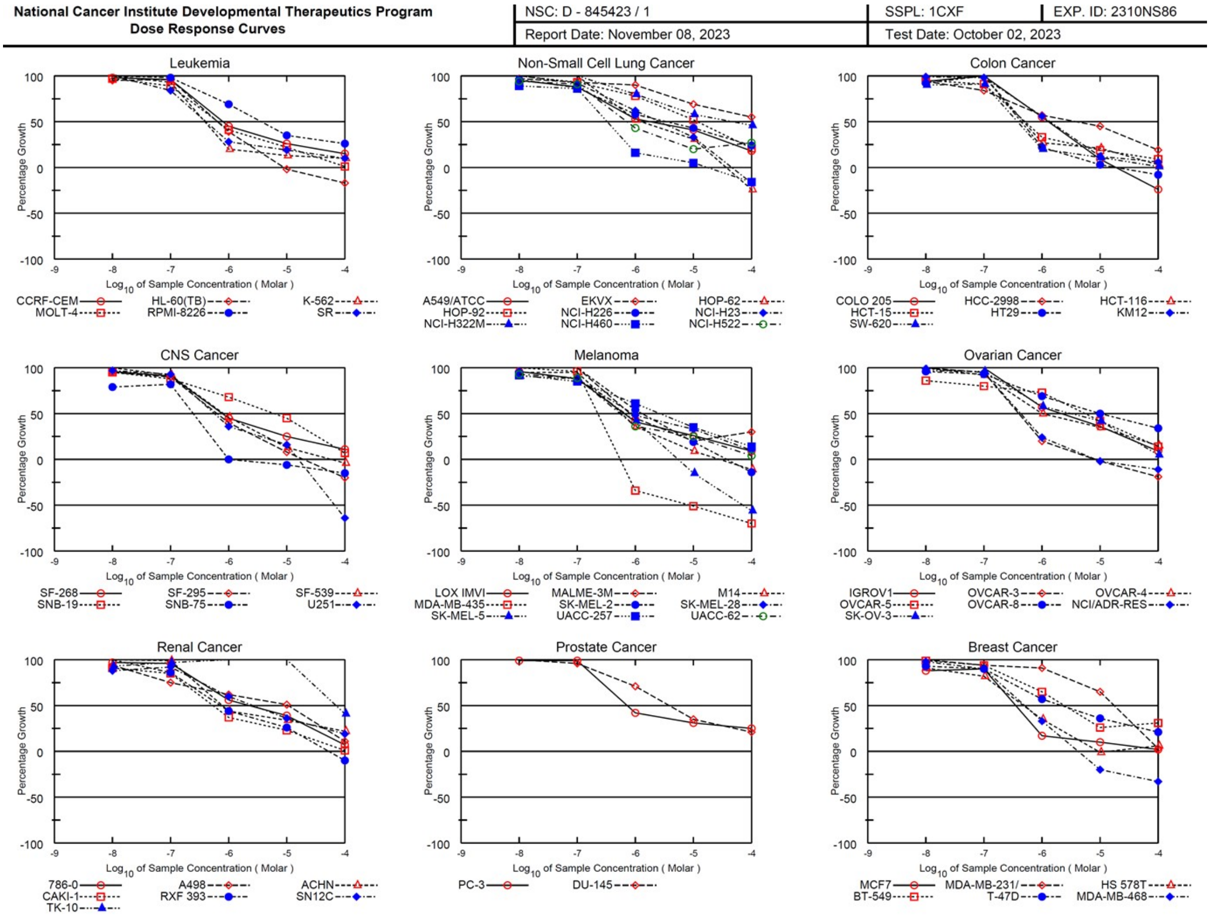
National Cancer Institute Developmental Therapeutics Program
Dose Response Curves
NSC: D - 845423 / 1
Report Date: November 08, 2023
SSPL: 1CXF
EXP. ID: 2310NS86
Test Date: October 02, 2023
Leukemia
CCRF-CEM
HL-60(TB)
K-562
MOLT-4
RPMI-8226
SR
Non-Small Cell Lung Cancer
A549/ATCC
EKVX
HOP-62
HOP-92
NCI-H226
NCI-H23
NCI-H322M
NCI-H460
NCI-H522
Colon Cancer
COLO 205
HCC-2998
HCT-116
HCT-15
HT29
KM12
SW-620
CNS Cancer
SF-268
SF-295
SF-539
SNB-19
SNB-75
U251
Melanoma
LOX IMVI
MALME-3M
M14
MDA-MB-435
SK-MEL-2
SK-MEL-28
SK-MEL-5
UACC-257
UACC-62
Ovarian Cancer
IGROV1
OVCAR-3
OVCAR-4
OVCAR-5
OVCAR-8
NCI/ADR-RES
SK-OV-3
Renal Cancer
786-0
A498
ACHN
CAKI-1
RXF 393
SN12C
TK-10
Prostate Cancer
PC-3
DU-145
Breast Cancer
MCF7
MDA-MB-231/
HS 578T
BT-549
T-47D
MDA-MB-468
Percentage Growth
Log10 of Sample Concentration (Molar)

National Cancer Institute Developmental Therapeutics Program
In-Vitro Testing Results

NSC : D - 845423 / 1	Experiment ID : 2310NS86	Test Type : 08	Units : Molar
Report Date : November 08, 2023	Test Date : October 02, 2023	QNS :	MC :
COMI : TO52	Stain Reagent : SRB Dual-Pass Related	SSPL : 1CXF	

			Log10 Concentration												
	Time		Mean Optical Densities					Percent Growth							
Panel/Cell Line	Zero	Ctrl	-8.0	-7.0	-6.0	-5.0	-4.0	-8.0	-7.0	-6.0	-5.0	-4.0	GI50	TGI	LC50
Leukemia															
CCRF-CEM	0.563	2.708	2.666	2.625	1.529	1.125	0.884	98	96	45	26	15	7.99E-7	> 1.00E-4	> 1.00E-4
HL-60(TB)	0.776	2.875	2.780	2.783	1.589	0.762	0.641	95	96	39	-2	-17	6.33E-7	8.99E-6	> 1.00E-4
K-562	0.221	1.937	1.939	1.820	0.560	0.436	0.399	100	93	20	13	10	3.87E-7	> 1.00E-4	> 1.00E-4
MOLT-4	0.609	2.640	2.583	2.408	1.449	1.055	0.629	97	89	41	22	1	6.57E-7	> 1.00E-4	> 1.00E-4
RPMI-8226	0.518	2.210	2.295	2.169	1.691	1.117	0.959	105	98	69	35	26	3.71E-6	> 1.00E-4	> 1.00E-4
SR	0.364	1.896	1.922	1.646	0.797	0.662	0.512	102	84	28	19	10	4.05E-7	> 1.00E-4	> 1.00E-4
Non-Small Cell Lung Cancer															
A549/ATCC	0.333	2.100	2.009	1.885	1.271	1.056	0.653	95	88	53	41	18	1.79E-6	> 1.00E-4	> 1.00E-4
EKVX	1.086	2.450	2.409	2.350	2.319	2.023	1.837	97	93	90	69	55	> 1.00E-4	> 1.00E-4	> 1.00E-4
HOP-62	0.559	1.609	1.636	1.648	1.106	0.890	0.423	103	104	52	31	-24	1.26E-6	3.66E-5	> 1.00E-4
HOP-92	1.079	1.815	1.816	1.764	1.654	1.462	1.236	100	93	78	52	21	1.16E-5	> 1.00E-4	> 1.00E-4
NCI-H226	1.191	2.391	2.341	2.230	1.885	1.708	1.475	96	87	58	43	24	3.39E-6	> 1.00E-4	> 1.00E-4
NCI-H23	0.621	2.299	2.294	2.157	1.654	1.181	0.514	100	92	62	33	-17	2.57E-6	4.57E-5	> 1.00E-4
NCI-H322M	0.862	2.366	2.406	2.465	2.068	1.729	1.556	103	107	80	58	46	4.64E-5	> 1.00E-4	> 1.00E-4
NCI-H460	0.359	2.348	2.136	2.069	0.684	0.455	0.303	89	86	16	5	-16	3.28E-7	1.72E-5	> 1.00E-4
NCI-H522	1.404	2.984	2.885	2.847	2.086	1.718	1.829	94	91	43	20	27	7.22E-7	> 1.00E-4	> 1.00E-4
Colon Cancer															
COLO 205	0.503	1.910	1.830	1.929	1.275	0.629	0.384	94	101	55	9	-24	1.28E-6	1.88E-5	> 1.00E-4
HCC-2998	0.853	3.130	2.982	2.768	2.152	1.867	1.289	94	84	57	45	19	3.66E-6	> 1.00E-4	> 1.00E-4
HCT-116	0.349	2.790	2.796	2.800	1.019	0.864	0.432	100	100	27	21	3	4.91E-7	> 1.00E-4	> 1.00E-4
HCT-15	0.382	2.718	2.592	2.497	1.149	0.829	0.590	95	91	33	19	9	5.04E-7	> 1.00E-4	> 1.00E-4
HT29	0.257	1.732	1.619	1.830	0.585	0.299	0.236	92	107	22	3	-8	4.69E-7	1.79E-5	> 1.00E-4
KM12	0.611	2.721	2.696	2.679	1.799	0.870	0.734	99	98	56	12	6	1.39E-6	> 1.00E-4	> 1.00E-4
SW-620	0.402	1.969	1.806	1.825	0.723	0.569	0.413	90	91	20	11	1	3.80E-7	> 1.00E-4	> 1.00E-4
CNS Cancer															
SF-268	0.763	2.146	2.084	2.026	1.383	1.111	0.920	96	91	45	25	11	7.74E-7	> 1.00E-4	> 1.00E-4
SF-295	1.288	3.165	3.078	2.980	2.032	1.448	1.034	95	90	40	8	-20	6.23E-7	2.00E-5	> 1.00E-4
SF-539	0.714	2.257	2.250	2.136	1.425	0.921	0.683	100	92	46	13	-4	8.22E-7	5.70E-5	> 1.00E-4
SNB-19	0.850	2.342	2.265	2.156	1.864	1.527	0.961	95	88	68	45	7	6.25E-6	> 1.00E-4	> 1.00E-4
SNB-75	0.927	1.613	1.470	1.486	0.922	0.873	0.788	79	82	0	-6	-15	2.42E-7	9.85E-7	> 1.00E-4
U251	0.268	1.484	1.446	1.399	0.710	0.462	0.097	97	93	36	16	-64	5.74E-7	1.58E-5	6.68E-5
Melanoma															
LOX IMVI	0.464	2.632	2.545	2.372	1.357	1.021	0.657	96	88	41	26	9	6.47E-7	> 1.00E-4	> 1.00E-4
MALME-3M	0.739	1.684	1.745	1.702	1.169	0.930	1.019	106	102	45	20	30	8.30E-7	> 1.00E-4	> 1.00E-4
M14	0.494	1.802	1.739	1.736	0.961	0.606	0.439	95	95	36	9	-11	5.74E-7	2.70E-5	> 1.00E-4
MDA-MB-435	0.751	2.268	2.264	2.210	0.492	0.369	0.228	100	96	-34	-51	-70	2.26E-7	5.45E-7	8.78E-6
SK-MEL-2	1.244	2.432	2.469	2.440	1.885	1.469	1.075	103	101	54	19	-14	1.30E-6	3.82E-5	> 1.00E-4
SK-MEL-28	0.803	2.435	2.365	2.235	1.586	1.336	0.967	96	88	48	33	10	8.90E-7	> 1.00E-4	> 1.00E-4
SK-MEL-5	1.062	3.271	3.081	3.010	1.990	0.900	0.463	91	88	42	-15	-56	6.71E-7	5.41E-6	6.99E-5
UACC-257	1.089	2.793	2.661	2.540	2.124	1.681	1.326	92	85	61	35	14	2.58E-6	> 1.00E-4	> 1.00E-4
UACC-62	1.130	3.089	2.959	2.872	1.829	1.619	1.212	93	89	36	25	4	5.38E-7	> 1.00E-4	> 1.00E-4
Ovarian Cancer															
IGROV1	0.598	2.230	2.372	2.509	1.521	1.179	0.745	109	117	57	36	9	2.06E-6	> 1.00E-4	> 1.00E-4
OVCAR-3	0.648	1.924	2.001	1.859	0.903	0.634	0.528	106	95	20	-2	-19	3.97E-7	7.93E-6	> 1.00E-4
OVCAR-4	0.950	2.239	2.234	2.140	1.600	1.405	1.144	100	92	50	35	15	1.06E-6	> 1.00E-4	> 1.00E-4
OVCAR-5	0.593	1.490	1.365	1.314	1.246	0.966	0.717	86	80	73	42	14	5.37E-6	> 1.00E-4	> 1.00E-4
OVCAR-8	0.526	2.519	2.436	2.374	1.908	1.525	1.197	96	93	69	50	34	1.02E-5	> 1.00E-4	> 1.00E-4
NCI/ADR-RES	0.585	2.090	2.080	2.021	0.949	0.571	0.519	99	95	24	-2	-11	4.34E-7	8.13E-6	> 1.00E-4
SK-OV-3	0.720	1.685	1.653	1.651	1.276	1.129	0.764	97	96	58	42	5	3.14E-6	> 1.00E-4	> 1.00E-4
Renal Cancer															
786-0	0.686	2.744	2.691	2.660	1.830	1.498	0.832	97	96	56	39	7	2.21E-6	> 1.00E-4	> 1.00E-4
A498	1.481	2.293	2.242	2.090	1.986	1.896	1.571	94	75	62	51	11	1.07E-5	> 1.00E-4	> 1.00E-4
ACHN	0.349	1.641	1.661	1.633	0.923	0.784	0.634	101	99	44	34	22	7.91E-7	> 1.00E-4	> 1.00E-4
CAKI-1	0.581	2.059	1.921	1.844	1.121	0.918	0.595	91	85	37	23	1	5.30E-7	> 1.00E-4	> 1.00E-4
RXF 393	1.053	1.801	1.807	1.696	1.385	1.250	0.952	101	86	44	26	-10	7.34E-7	5.39E-5	> 1.00E-4
SN12C	0.543	2.174	1.983	2.043	1.523	1.124	0.850	88	92	60	36	19	2.58E-6	> 1.00E-4	> 1.00E-4
TK-10	1.244	2.175	2.110	2.145	2.261	2.326	1.626	93	97	109	116	41	7.60E-5	> 1.00E-4	> 1.00E-4
Prostate Cancer															
PC-3	0.515	2.085	2.074	2.076	1.174	0.997	0.910	99	99	42	31	25	7.24E-7	> 1.00E-4	> 1.00E-4
DU-145	0.361	1.618	1.637	1.570	1.258	0.801	0.621	102	96	71	35	21	3.87E-6	> 1.00E-4	> 1.00E-4
Breast Cancer															
MCF7	0.544	2.394	2.178	2.207	0.864	0.730	0.582	88	90	17	10	2	3.54E-7	> 1.00E-4	> 1.00E-4
MDA-MB-231/ATCC	0.605	1.228	1.260	1.189	1.174	1.009	0.627	105	94	91	65	3	1.74E-5	> 1.00E-4	> 1.00E-4
HS 578T	1.372	2.406	2.312	2.218	1.739	1.352	1.437	91	82	35	-1	6	4.86E-7	.	> 1.00E-4
BT-549	1.308	2.362	2.348	2.300	1.995	1.587	1.635	99	94	65	26	31	2.47E-6	> 1.00E-4	> 1.00E-4
T-47D	0.798	1.846	1.777	1.739	1.397	1.180	1.022	93	90	57	36	21	2.21E-6	> 1.00E-4	> 1.00E-4
MDA-MB-468	1.202	2.408	2.380	2.294	1.598	0.962	0.807	98	90	33	-20	-33	5.03E-7	4.18E-6	> 1.00E-4

Compound **3r**

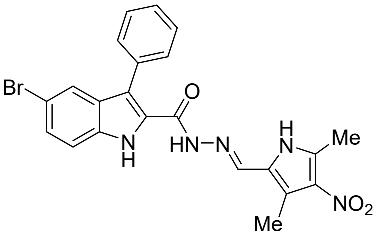
Br
O
HN–N
H
N
Me
NO2
Me
N
H

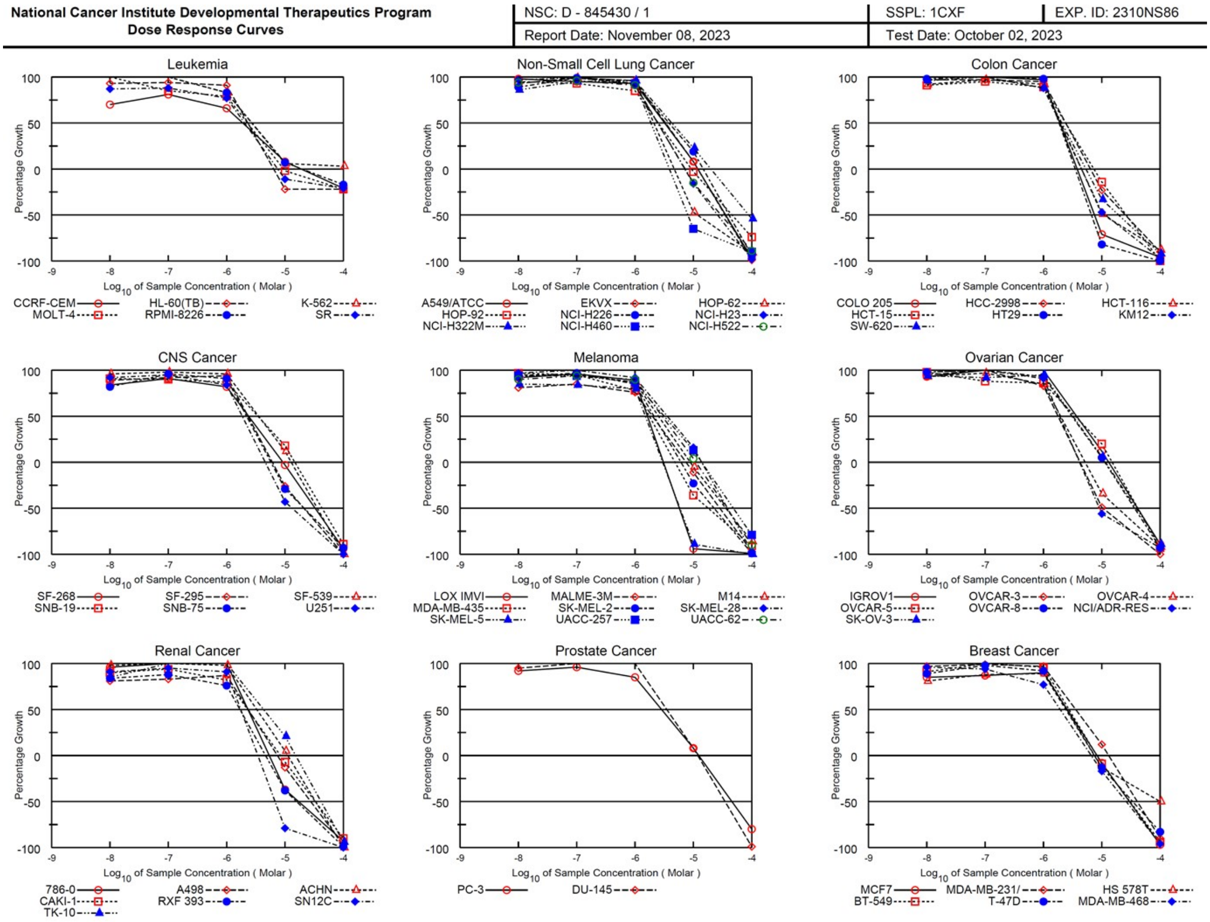
National Cancer Institute Developmental Therapeutics Program
Dose Response Curves
NSC: D - 845430 / 1
Report Date: November 08, 2023
SSPL: 1CXF
EXP. ID: 2310NS86
Test Date: October 02, 2023
Leukemia
Non-Small Cell Lung Cancer
Colon Cancer
CNS Cancer
Melanoma
Ovarian Cancer
Renal Cancer
Prostate Cancer
Breast Cancer
Percentage Growth
Log10 of Sample Concentration (Molar)
CCRF-CEM
HL-60(TB)
K-562
MOLT-4
RPMI-8226
SR
A549/ATCC
EKVX
HOP-62
HOP-92
NCI-H226
NCI-H23
NCI-H322M
NCI-H460
NCI-H522
COLO 205
HCC-2998
HCT-116
HCT-15
HT29
KM12
SW-620
SF-268
SF-295
SF-539
SNB-19
SNB-75
U251
LOX IMVI
MALME-3M
M14
MDA-MB-435
SK-MEL-2
SK-MEL-28
SK-MEL-5
UACC-257
UACC-62
IGROV1
OVCAR-3
OVCAR-4
OVCAR-5
OVCAR-8
NCI/ADR-RES
SK-OV-3
786-0
A498
ACHN
CAKI-1
RXF 393
SN12C
TK-10
PC-3
DU-145
MCF7
MDA-MB-231/
HS 578T
BT-549
T-47D
MDA-MB-468

National Cancer Institute Developmental Therapeutics Program
In-Vitro Testing Results

NSC : D - 845430 / 1	Experiment ID : 2310NS86	Test Type : 08	Units : Molar
Report Date : November 08, 2023	Test Date : October 02, 2023	QNS :	MC :
COMI : TO59	Stain Reagent : SRB Dual-Pass Related	SSPL : 1CXF	

			Log10 Concentration													
	Time		Mean Optical Densities					Percent Growth							
Panel/Cell Line	Zero	Ctrl	-8.0	-7.0	-6.0	-5.0	-4.0	-8.0	-7.0	-6.0	-5.0	-4.0	GI50	TGI	LC50
**Leukemia**															
CCRF-CEM	0.563	2.779	2.115	2.363	2.024	0.743	0.448	70	81	66	8	-21	1.89E-6	1.92E-5	> 1.00E-4
HL-60(TB)	0.776	3.029	2.865	2.889	2.824	0.608	0.605	93	94	91	-22	-22	2.31E-6	6.42E-6	> 1.00E-4
K-562	0.221	1.956	1.955	1.967	1.665	0.323	0.275	100	101	83	6	3	2.69E-6	> 1.00E-4	> 1.00E-4
MOLT-4	0.609	2.594	2.628	2.302	2.184	0.595	0.473	102	85	79	-2	-22	2.29E-6	9.37E-6	> 1.00E-4
RPMI-8226	0.518	2.030	2.082	2.134	1.768	0.623	0.433	103	107	83	7	-17	2.70E-6	1.97E-5	> 1.00E-4
SR	0.364	1.947	1.747	1.755	1.586	0.325	0.289	87	88	77	-11	-21	2.04E-6	7.55E-6	> 1.00E-4
**Non-Small Cell Lung Cancer**															
A549/ATCC	0.333	1.973	1.946	1.897	1.860	0.458	0.011	98	95	93	8	-97	3.19E-6	1.18E-5	3.56E-5
EKVX	1.086	2.587	2.479	2.577	2.528	1.208	0.013	93	99	96	8	-99	3.34E-6	1.19E-5	3.49E-5
HOP-62	0.559	1.521	1.569	1.508	1.480	0.296	0.051	105	99	96	-47	-91	2.09E-6	4.68E-6	1.17E-5
HOP-92	1.079	1.834	1.796	1.779	1.724	1.043	0.282	95	93	85	-3	-74	2.51E-6	9.16E-6	4.58E-5
NCI-H226	1.191	2.429	2.293	2.422	2.339	1.423	0.070	89	99	93	19	-94	3.78E-6	1.46E-5	4.06E-5
NCI-H23	0.621	2.422	2.372	2.399	2.283	0.524	0.016	97	99	92	-16	-98	2.46E-6	7.15E-6	2.63E-5
NCI-H322M	0.862	2.225	2.032	2.151	2.167	1.170	0.400	86	95	96	23	-54	4.22E-6	1.98E-5	8.95E-5
NCI-H460	0.359	2.009	2.180	2.192	1.891	0.126	0.037	110	111	93	-65	-90	1.87E-6	3.88E-6	8.05E-6
NCI-H522	1.404	3.221	3.106	3.142	3.053	1.193	0.153	94	96	91	-15	-89	2.43E-6	7.21E-6	2.96E-5
**Colon Cancer**															
COLO 205	0.503	1.733	1.806	1.925	1.696	0.147	0.021	106	116	97	-71	-96	1.90E-6	3.78E-6	7.51E-6
HCC-2998	0.853	3.141	3.078	3.069	3.028	0.660	0.083	97	97	95	-23	-90	2.41E-6	6.42E-6	2.54E-5
HCT-116	0.349	2.419	2.257	2.354	2.254	0.177	0.041	92	97	92	-49	-88	1.98E-6	4.48E-6	1.04E-5
HCT-15	0.382	2.771	2.567	2.660	2.518	0.330	-0.005	91	95	89	-14	-100	2.41E-6	7.38E-6	2.64E-5
HT29	0.257	1.740	1.714	1.830	1.703	0.048	-0.008	98	106	98	-82	-100	1.84E-6	3.50E-6	6.67E-6
KM12	0.611	2.642	2.660	2.707	2.407	0.326	0.021	101	103	88	-47	-97	1.92E-6	4.51E-6	1.16E-5
SW-620	0.402	1.794	1.742	1.788	1.630	0.268	0.031	96	100	88	-33	-92	2.06E-6	5.31E-6	1.91E-5
**CNS Cancer**															
SF-268	0.763	2.242	2.010	2.102	1.971	0.743	0.048	84	91	82	-3	-94	2.37E-6	9.29E-6	3.31E-5
SF-295	1.288	3.190	2.989	3.039	3.070	0.956	0.003	89	92	94	-26	-100	2.32E-6	6.08E-6	2.12E-5
SF-539	0.714	2.357	2.290	2.325	2.287	0.910	-0.002	96	98	96	12	-100	3.51E-6	1.28E-5	3.57E-5
SNB-19	0.850	2.362	2.218	2.211	2.163	1.128	0.090	91	90	87	18	-89	3.45E-6	1.48E-5	4.31E-5
SNB-75	0.927	1.404	1.320	1.387	1.359	0.661	0.066	82	96	91	-29	-93	2.19E-6	5.75E-6	2.15E-5
U251	0.268	1.494	1.401	1.433	1.297	0.154	-0.003	92	95	84	-43	-100	1.85E-6	4.60E-6	1.34E-5
**Melanoma**															
LOX IMVI	0.464	2.847	2.653	2.743	2.589	0.026	0.004	92	96	89	-94	-99	1.63E-6	3.06E-6	5.73E-6
MALME-3M	0.739	1.699	1.518	1.558	1.468	0.657	0.026	81	85	76	-11	-97	1.99E-6	7.44E-6	2.85E-5
M14	0.494	1.642	1.584	1.590	1.498	0.470	0.072	95	95	87	-5	-86	2.54E-6	8.86E-6	3.63E-5
MDA-MB-435	0.751	2.340	2.296	2.255	1.997	0.481	0.067	97	95	78	-36	-91	1.77E-6	4.84E-6	1.79E-5
SK-MEL-2	1.244	2.512	2.426	2.475	2.326	0.954	0.017	93	97	85	-23	-99	2.11E-6	6.10E-6	2.26E-5
SK-MEL-28	0.803	2.314	2.264	2.329	2.187	1.049	0.028	97	101	92	16	-97	3.56E-6	1.39E-5	3.87E-5
SK-MEL-5	1.062	3.341	3.000	2.982	2.876	0.117	-0.007	85	84	80	-89	-100	1.50E-6	2.97E-6	5.87E-6
UACC-257	1.089	2.845	2.737	2.735	2.613	1.311	0.227	94	94	87	13	-79	3.13E-6	1.37E-5	4.81E-5
UACC-62	1.130	3.069	2.880	2.947	2.866	1.206	0.114	90	94	90	4	-90	2.90E-6	1.10E-5	3.75E-5
**Ovarian Cancer**															
IGROV1	0.598	2.046	1.946	2.088	2.098	0.768	0.068	93	103	104	12	-89	3.83E-6	1.31E-5	4.12E-5
OVCAR-3	0.648	1.980	1.917	1.991	1.751	0.329	-0.007	95	101	83	-49	-100	1.77E-6	4.24E-6	1.04E-5
OVCAR-4	0.950	2.081	2.001	2.051	1.910	0.627	0.074	93	97	85	-34	-92	1.97E-6	5.18E-6	1.88E-5
OVCAR-5	0.593	1.503	1.488	1.393	1.378	0.775	0.040	98	88	86	20	-93	3.53E-6	1.50E-5	4.15E-5
OVCAR-8	0.526	2.450	2.418	2.516	2.309	0.628	0.037	98	103	93	5	-93	3.08E-6	1.13E-5	3.65E-5
NCI/ADR-RES	0.585	2.189	2.161	2.216	2.038	0.260	0.043	98	102	91	-56	-93	1.89E-6	4.16E-6	9.15E-6
SK-OV-3	0.720	1.585	1.534	1.512	1.545	0.775	0.083	94	92	95	6	-89	3.23E-6	1.17E-5	3.92E-5
**Renal Cancer**															
786-0	0.686	2.384	2.318	2.439	2.411	0.431	0.051	96	103	102	-37	-93	2.35E-6	5.40E-6	1.70E-5
A498	1.481	2.250	2.106	2.117	2.152	1.287	0.049	81	83	87	-13	-97	2.35E-6	7.40E-6	2.76E-5
ACHN	0.349	1.742	1.713	1.766	1.719	0.418	-0.009	98	102	98	5	-100	3.30E-6	1.11E-5	3.34E-5
CAKI-1	0.581	2.022	1.880	1.939	1.760	0.543	0.057	90	94	82	-7	-90	2.29E-6	8.43E-6	3.31E-5
RXF 393	1.053	1.870	1.738	1.773	1.677	0.657	0.013	84	88	76	-38	-99	1.70E-6	4.67E-6	1.59E-5
SN12C	0.543	2.111	1.968	2.033	1.968	0.113	-0.007	91	95	91	-79	-100	1.74E-6	3.42E-6	6.73E-6
TK-10	1.244	2.171	2.034	2.212	2.364	1.436	0.071	85	104	121	21	-94	5.10E-6	1.51E-5	4.12E-5
**Prostate Cancer**															
PC-3	0.515	2.187	2.045	2.128	1.940	0.647	0.103	92	96	85	8	-80	2.85E-6	1.23E-5	4.55E-5
DU-145	0.361	1.749	1.681	1.746	1.745	0.472	0.005	95	100	100	8	-99	3.48E-6	1.19E-5	3.49E-5
**Breast Cancer**															
MCF7	0.544	2.485	2.185	2.232	2.294	0.502	0.019	85	87	90	-8	-97	2.57E-6	8.34E-6	2.99E-5
MDA-MB-231/ATCC	0.605	1.276	1.254	1.269	1.255	0.685	0.061	97	99	97	12	-90	3.56E-6	1.31E-5	4.05E-5
HS 578T	1.372	2.278	2.104	2.172	2.182	1.201	0.680	81	88	89	-13	-50	2.43E-6	7.54E-6	9.72E-5
BT-549	1.308	2.221	2.135	2.225	2.184	1.190	0.085	91	100	96	-9	-94	2.74E-6	8.20E-6	3.06E-5
T-47D	0.798	1.683	1.587	1.668	1.615	0.692	0.134	89	98	92	-13	-83	2.51E-6	7.48E-6	3.35E-5
MDA-MB-468	1.202	2.484	2.430	2.412	2.192	0.994	0.051	96	94	77	-17	-96	1.94E-6	6.55E-6	2.61E-5

Compound **3s**

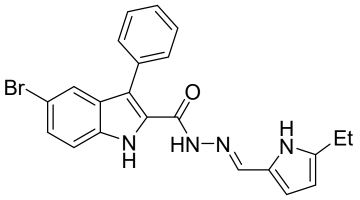
Br
O
HN–N
H
N
Et
N
H

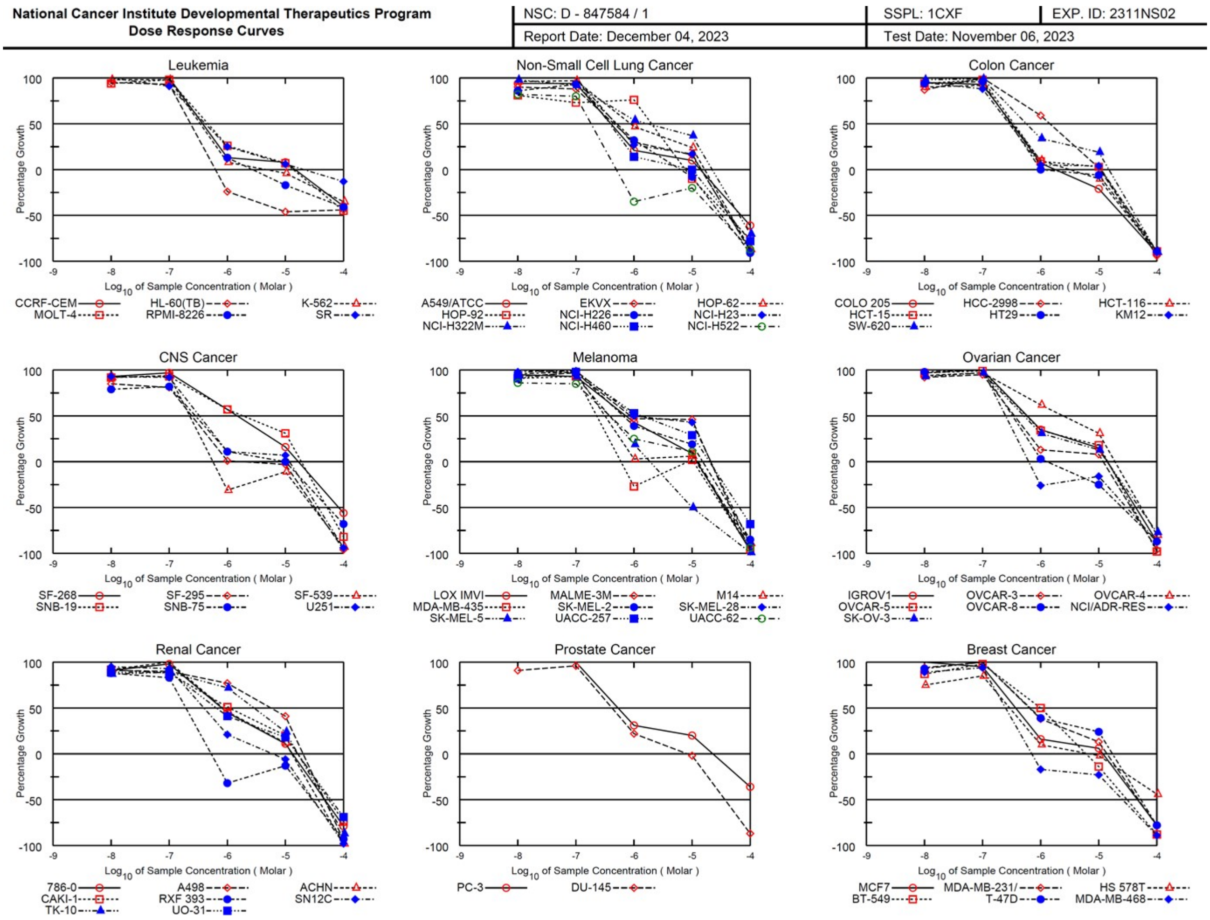
National Cancer Institute Developmental Therapeutics Program
Dose Response Curves
NSC: D - 847584 / 1
Report Date: December 04, 2023
SSPL: 1CXF
EXP. ID: 2311NS02
Test Date: November 06, 2023
Leukemia
CCRF-CEM HL-60(TB) K-562 MOLT-4 RPMI-8226 SR
Non-Small Cell Lung Cancer
A549/ATCC EKVX HOP-62 HOP-92 NCI-H226 NCI-H23 NCI-H322M NCI-H460 NCI-H522
Colon Cancer
COLO 205 HCC-2998 HCT-116 HCT-15 HT29 KM12 SW-620
CNS Cancer
SF-268 SF-295 SF-539 SNB-19 SNB-75 U251
Melanoma
LOX IMVI MALME-3M M14 MDA-MB-435 SK-MEL-2 SK-MEL-28 SK-MEL-5 UACC-257 UACC-62
Ovarian Cancer
IGROV1 OVCAR-3 OVCAR-4 OVCAR-5 OVCAR-8 NCI/ADR-RES SK-OV-3
Renal Cancer
786-0 A498 ACHN CAKI-1 RXF 393 SN12C TK-10 UO-31
Prostate Cancer
PC-3 DU-145
Breast Cancer
MCF7 MDA-MB-231/ HS 578T BT-549 T-47D MDA-MB-468
Percentage Growth
Log10 of Sample Concentration (Molar)

National Cancer Institute Developmental Therapeutics Program
In-Vitro Testing Results

NSC : D - 847584 / 1	Experiment ID : 2311NS02	Test Type : 08	Units : Molar
Report Date : December 04, 2023	Test Date : November 06, 2023	QNS :	MC :
COMI : TO61	Stain Reagent : SRB Dual-Pass Related	SSPL : 1CXF	

			Log10 Concentration												
	Time		Mean Optical Densities					Percent Growth							
Panel/Cell Line	Zero	Ctrl	-8.0	-7.0	-6.0	-5.0	-4.0	-8.0	-7.0	-6.0	-5.0	-4.0	GI50	TGI	LC50
**Leukemia**															
CCRF-CEM	0.546	2.925	2.945	2.920	0.856	0.731	0.327	101	100	13	8	-40	3.75E-7	1.45E-5	> 1.00E-4
HL-60(TB)	0.835	3.007	2.898	2.866	0.637	0.448	0.469	95	93	-24	-46	-44	2.35E-7	6.28E-7	> 1.00E-4
K-562	0.279	2.009	1.981	1.970	0.410	0.268	0.182	98	98	8	-4	-35	3.38E-7	4.54E-6	> 1.00E-4
MOLT-4	0.563	2.435	2.315	2.400	1.049	0.702	0.311	94	98	26	7	-45	4.64E-7	1.39E-5	> 1.00E-4
RPMI-8226	0.827	2.220	2.226	2.221	1.006	0.686	0.490	100	100	13	-17	-41	3.75E-7	2.69E-6	> 1.00E-4
SR	0.219	0.563	0.571	0.532	0.306	0.240	0.191	102	91	25	6	-13	4.19E-7	2.09E-5	> 1.00E-4
**Non-Small Cell Lung Cancer**															
A549/ATCC	0.217	1.198	1.138	1.140	0.420	0.316	0.085	94	94	21	10	-61	3.99E-7	1.39E-5	7.03E-5
EKVX	0.536	1.764	1.643	1.612	0.908	0.736	0.063	90	88	30	16	-88	4.53E-7	1.43E-5	4.30E-5
HOP-62	0.641	1.775	1.725	1.743	1.171	0.913	0.082	96	97	47	24	-87	8.61E-7	1.64E-5	4.62E-5
HOP-92	1.403	1.718	1.657	1.634	1.643	1.261	0.333	81	73	76	-10	-76	2.01E-6	7.63E-6	4.01E-5
NCI-H226	0.894	1.655	1.551	1.604	1.137	0.824	0.083	86	93	32	-8	-91	5.06E-7	6.35E-6	3.23E-5
NCI-H23	0.505	1.722	1.717	1.723	0.833	0.708	0.054	100	100	27	17	-89	4.84E-7	1.44E-5	4.26E-5
NCI-H322M	0.560	2.071	2.046	1.952	1.377	1.121	0.166	98	92	54	37	-70	1.73E-6	2.21E-5	6.46E-5
NCI-H460	0.286	2.453	2.468	2.519	0.596	0.287	0.062	101	103	14	0	-78	3.96E-7	1.00E-5	4.35E-5
NCI-H522	1.112	2.814	2.515	2.476	0.721	0.887	0.149	82	80	-35	-20	-87	1.83E-7	4.95E-7	2.80E-5
**Colon Cancer**															
COLO 205	0.476	2.080	2.001	1.970	0.585	0.377	0.045	95	93	7	-21	-91	3.16E-7	1.76E-6	2.61E-5
HCC-2998	0.821	2.699	2.450	2.724	1.933	0.862	0.053	87	101	59	2	-94	1.45E-6	1.05E-5	3.51E-5
HCT-116	0.267	2.090	1.911	1.939	0.454	0.241	0.028	90	92	10	-10	-90	3.25E-7	3.22E-6	3.19E-5
HCT-15	0.311	2.050	1.940	2.019	0.474	0.359	0.033	94	98	9	3	-89	3.49E-7	1.07E-5	3.74E-5
HT29	0.201	1.335	1.268	1.286	0.200	0.189	0.022	94	96	0	-6	-89	2.98E-7	9.82E-7	3.38E-5
KM12	0.615	2.374	2.290	2.163	0.712	0.686	0.061	95	88	5	4	-90	2.89E-7	1.10E-5	3.75E-5
SW-620	0.279	1.981	1.946	1.957	0.850	0.603	0.029	98	99	34	19	-90	5.58E-7	1.50E-5	4.32E-5
**CNS Cancer**															
SF-268	0.838	2.283	2.187	2.237	1.661	1.065	0.371	93	97	57	16	-56	1.47E-6	1.66E-5	8.30E-5
SF-295	0.431	1.904	1.682	1.629	0.447	0.416	0.019	85	81	1	-3	-96	2.46E-7	1.73E-6	3.19E-5
SF-539	0.637	2.043	1.937	1.961	0.439	0.566	0.045	92	94	-31	-11	-93	2.25E-7	5.65E-7	2.98E-5
SNB-19	0.692	1.919	1.825	1.836	1.389	1.069	0.123	92	93	57	31	-82	1.82E-6	1.87E-5	5.18E-5
SNB-75	1.075	2.104	1.889	1.924	1.188	1.071	0.343	79	82	11	0	-68	2.85E-7	9.27E-6	5.41E-5
U251	0.199	1.132	1.067	1.058	0.300	0.265	0.012	93	92	11	7	-94	3.29E-7	1.17E-5	3.67E-5
**Melanoma**															
LOX IMVI	0.472	3.086	2.952	2.915	1.592	0.718	0.014	95	93	43	9	-97	7.22E-7	1.23E-5	3.61E-5
MALME-3M	0.602	1.682	1.611	1.651	1.112	1.100	0.013	93	97	47	46	-98	8.80E-7	2.09E-5	4.65E-5
M14	0.428	1.515	1.533	1.486	0.462	0.495	0.046	102	97	3	6	-89	3.18E-7	1.16E-5	3.87E-5
MDA-MB-435	0.562	2.514	2.346	2.368	0.411	0.610	0.022	91	93	-27	2	-96	2.27E-7	.	3.40E-5
SK-MEL-2	1.611	3.189	3.131	3.154	2.225	1.905	0.241	96	98	39	19	-85	6.47E-7	1.51E-5	4.59E-5
SK-MEL-28	0.646	1.975	1.950	1.965	1.305	1.211	0.063	98	99	50	43	-90	9.79E-7	2.09E-5	4.98E-5
SK-MEL-5	1.258	3.300	3.201	3.164	1.641	0.631	0.008	95	93	19	-50	-99	3.81E-7	1.88E-6	1.01E-5
UACC-257	0.788	2.063	1.953	2.038	1.464	1.153	0.250	91	98	53	29	-68	1.32E-6	1.97E-5	6.47E-5
UACC-62	0.740	2.662	2.397	2.379	1.211	0.952	0.045	86	85	25	11	-94	3.81E-7	1.27E-5	3.81E-5
**Ovarian Cancer**															
IGROV1	0.491	1.987	1.981	2.061	1.017	0.709	0.065	100	105	35	15	-87	6.12E-7	1.39E-5	4.33E-5
OVCAR-3	0.610	2.126	2.006	2.057	0.806	0.738	0.022	92	95	13	8	-96	3.56E-7	1.20E-5	3.61E-5
OVCAR-4	0.855	2.145	2.062	2.110	1.650	1.256	0.171	94	97	62	31	-80	2.41E-6	1.91E-5	5.37E-5
OVCAR-5	0.523	1.462	1.438	1.454	0.845	0.691	0.013	97	99	34	18	-98	5.73E-7	1.43E-5	3.87E-5
OVCAR-8	0.394	1.708	1.686	1.739	0.437	0.295	0.051	98	102	3	-25	-87	3.37E-7	1.30E-6	2.52E-5
NCI/ADR-RES	0.514	1.958	1.921	1.974	0.382	0.434	0.065	97	101	-26	-16	-87	2.53E-7	6.27E-7	3.02E-5
SK-OV-3	0.822	1.821	1.750	1.789	1.132	0.956	0.188	93	97	31	13	-77	5.14E-7	1.41E-5	5.01E-5
**Renal Cancer**															
786-0	0.606	2.332	2.176	2.302	1.380	0.804	0.125	91	98	45	11	-79	8.01E-7	1.34E-5	4.74E-5
A498	1.529	2.366	2.288	2.281	2.172	1.872	0.093	91	90	77	41	-94	5.59E-6	2.01E-5	4.72E-5
ACHN	0.357	1.506	1.419	1.512	0.891	0.500	0.006	92	101	46	12	-98	8.61E-7	1.29E-5	3.66E-5
CAKI-1	0.633	2.110	1.987	1.931	1.380	0.935	0.168	92	88	51	20	-74	1.04E-6	1.65E-5	5.62E-5
RXF 393	1.036	1.278	1.248	1.238	0.700	0.902	0.069	88	83	-32	-13	-93	1.94E-7	5.24E-7	2.89E-5
SN12C	0.558	2.056	1.987	1.947	0.868	0.527	0.012	95	93	21	-6	-98	3.92E-7	6.11E-6	3.03E-5
TK-10	1.130	2.103	1.980	1.999	1.832	1.368	0.142	87	89	72	24	-87	2.91E-6	1.65E-5	4.63E-5
UO-31	0.596	2.297	2.115	2.115	1.289	0.898	0.183	89	89	41	18	-69	6.44E-7	1.60E-5	5.99E-5
**Prostate Cancer**															
PC-3	0.500	1.836	1.844	1.832	0.918	0.764	0.320	101	100	31	20	-36	5.32E-7	2.26E-5	> 1.00E-4
DU-145	0.494	1.676	1.575	1.632	0.751	0.484	0.065	91	96	22	-2	-87	4.18E-7	8.22E-6	3.67E-5
**Breast Cancer**															
MCF7	0.445	1.907	1.902	1.834	0.672	0.529	0.096	100	95	16	6	-78	3.69E-7	1.17E-5	4.59E-5
MDA-MB-231/ATCC	0.643	1.341	1.303	1.338	0.905	0.737	0.134	94	100	38	13	-79	6.30E-7	1.40E-5	4.84E-5
HS 578T	1.428	2.417	2.169	2.273	1.525	1.404	0.796	75	85	10	-2	-44	2.94E-7	7.09E-6	> 1.00E-4
BT-549	1.245	2.025	1.926	2.010	1.632	1.066	0.145	87	98	50	-14	-88	9.79E-7	5.95E-6	3.03E-5
T-47D	0.725	1.670	1.604	1.703	1.096	0.951	0.156	93	103	39	24	-78	6.81E-7	1.71E-5	5.27E-5
MDA-MB-468	0.842	1.366	1.306	1.332	0.702	0.652	0.092	89	94	-17	-23	-89	2.48E-7	7.06E-7	2.59E-5

Compound **3t**

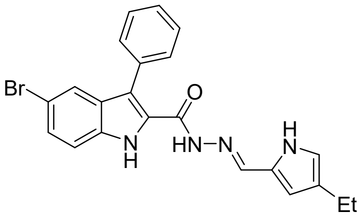

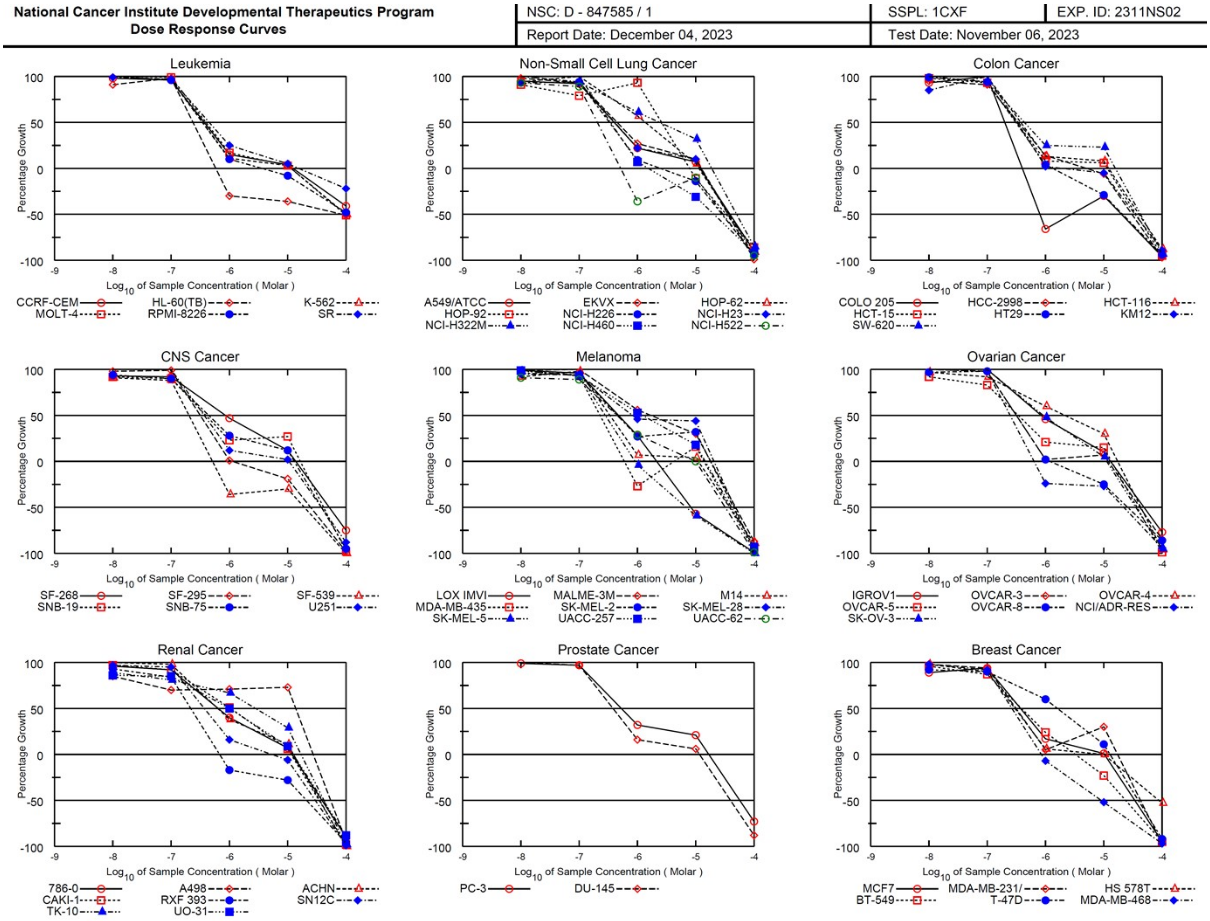
National Cancer Institute Developmental Therapeutics Program
Dose Response Curves
NSC: D - 847585 / 1
Report Date: December 04, 2023
SSPL: 1CXF
EXP. ID: 2311NS02
Test Date: November 06, 2023
Leukemia
Non-Small Cell Lung Cancer
Colon Cancer
CNS Cancer
Melanoma
Ovarian Cancer
Renal Cancer
Prostate Cancer
Breast Cancer
Percentage Growth
Log10 of Sample Concentration (Molar)
CCRF-CEM HL-60(TB) K-562 MOLT-4 RPMI-8226 SR
A549/ATCC EKVX HOP-62 HOP-92 NCI-H226 NCI-H23 NCI-H322M NCI-H460 NCI-H522
COLO 205 HCC-2998 HCT-116 HCT-15 HT29 KM12 SW-620
SF-268 SF-295 SF-539 SNB-19 SNB-75 U251
LOX IMVI MALME-3M M14 MDA-MB-435 SK-MEL-2 SK-MEL-28 SK-MEL-5 UACC-257 UACC-62
IGROV1 OVCAR-3 OVCAR-4 OVCAR-5 OVCAR-8 NCI/ADR-RES SK-OV-3
786-0 A498 ACHN CAKI-1 RXF 393 SN12C TK-10 UO-31
PC-3 DU-145
MCF7 MDA-MB-231/ HS 578T BT-549 T-47D MDA-MB-468

National Cancer Institute Developmental Therapeutics Program
In-Vitro Testing Results

NSC : D - 847585 / 1	Experiment ID : 2311NS02	Test Type : 08	Units : Molar
Report Date : December 04, 2023	Test Date : November 06, 2023	QNS :	MC :
COMI : TO62	Stain Reagent : SRB Dual-Pass Related	SSPL : 1CXF	

			Log10 Concentration												
	Time		Mean Optical Densities					Percent Growth							
Panel/Cell Line	Zero	Ctrl	-8.0	-7.0	-6.0	-5.0	-4.0	-8.0	-7.0	-6.0	-5.0	-4.0	GI50	TGI	LC50
**Leukemia**															
CCRF-CEM	0.546	3.038	2.981	2.926	0.922	0.638	0.322	98	96	15	4	-41	3.68E-7	1.21E-5	> 1.00E-4
HL-60(TB)	0.835	3.077	2.873	3.028	0.585	0.536	0.413	91	98	-30	-36	-51	2.37E-7	5.83E-7	9.19E-5
K-562	0.279	2.073	2.066	1.997	0.482	0.335	0.140	100	96	11	3	-50	3.48E-7	1.14E-5	> 1.00E-4
MOLT-4	0.563	2.491	2.497	2.481	0.894	0.629	0.274	100	99	17	3	-51	3.99E-7	1.15E-5	9.42E-5
RPMI-8226	0.827	2.245	2.288	2.188	0.971	0.760	0.434	103	96	10	-8	-48	3.43E-7	3.58E-6	> 1.00E-4
SR	0.219	0.594	0.590	0.582	0.312	0.237	0.170	99	97	25	5	-22	4.46E-7	1.49E-5	> 1.00E-4
**Non-Small Cell Lung Cancer**															
A549/ATCC	0.217	1.313	1.258	1.222	0.460	0.294	0.022	95	92	22	7	-90	3.97E-7	1.18E-5	3.88E-5
EKVX	0.536	1.764	1.736	1.682	0.865	0.663	0.006	98	93	27	10	-99	4.48E-7	1.24E-5	3.56E-5
HOP-62	0.641	1.783	1.712	1.819	1.289	0.698	0.059	94	103	57	5	-91	1.35E-6	1.13E-5	3.75E-5
HOP-92	1.403	1.855	1.812	1.758	1.821	1.242	0.199	91	79	93	-11	-86	2.57E-6	7.76E-6	3.30E-5
NCI-H226	0.894	1.679	1.626	1.633	0.966	0.767	0.052	93	94	9	-14	-94	3.30E-7	2.47E-6	2.80E-5
NCI-H23	0.505	1.709	1.706	1.636	0.771	0.629	0.026	100	94	22	10	-95	4.08E-7	1.25E-5	3.74E-5
NCI-H322M	0.560	2.127	2.183	2.030	1.508	1.056	0.082	104	94	61	32	-85	2.31E-6	1.86E-5	4.98E-5
NCI-H460	0.286	2.283	2.374	2.293	0.424	0.196	0.022	105	100	7	-31	-92	3.46E-7	1.51E-6	2.02E-5
NCI-H522	1.112	2.848	2.733	2.658	0.716	1.001	0.053	93	89	-36	-10	-95	2.06E-7	5.18E-7	2.95E-5
**Colon Cancer**															
COLO 205	0.476	2.094	1.980	2.146	0.163	0.333	0.020	93	103	-66	-30	-96	2.07E-7	4.08E-7	.
HCC-2998	0.821	2.728	2.639	2.561	1.089	0.772	0.024	95	91	14	-6	-97	3.42E-7	5.01E-6	3.04E-5
HCT-116	0.267	2.062	2.183	2.107	0.498	0.407	0.033	107	102	13	8	-88	3.85E-7	1.21E-5	4.03E-5
HCT-15	0.311	2.034	2.007	1.915	0.441	0.413	0.014	98	93	8	6	-95	3.19E-7	1.14E-5	3.56E-5
HT29	0.201	1.353	1.347	1.279	0.250	0.143	0.012	99	94	4	-29	-94	3.08E-7	1.34E-6	2.10E-5
KM12	0.615	2.343	2.091	2.340	0.658	0.584	0.069	85	100	2	-5	-89	3.25E-7	2.13E-6	3.44E-5
SW-620	0.279	1.786	1.783	1.691	0.653	0.626	0.019	100	94	25	23	-93	4.31E-7	1.58E-5	4.25E-5
**CNS Cancer**															
SF-268	0.838	2.192	2.096	2.087	1.476	1.003	0.212	93	92	47	12	-75	8.61E-7	1.38E-5	5.19E-5
SF-295	0.431	1.922	1.897	1.912	0.450	0.351	0.007	98	99	1	-19	-98	3.19E-7	1.16E-6	2.47E-5
SF-539	0.637	2.041	1.912	1.875	0.409	0.449	0.002	91	88	-36	-30	-100	2.03E-7	5.14E-7	1.96E-5
SNB-19	0.692	1.913	1.811	1.796	0.971	1.022	0.014	92	90	23	27	-98	3.96E-7	1.65E-5	4.13E-5
SNB-75	1.075	1.955	1.905	1.868	1.320	1.179	0.052	94	90	28	12	-95	4.40E-7	1.29E-5	3.78E-5
U251	0.199	1.125	1.123	1.127	0.314	0.221	0.025	100	100	12	2	-88	3.73E-7	1.06E-5	3.82E-5
**Melanoma**															
LOX IMVI	0.472	3.079	3.043	2.983	1.191	0.203	0.004	99	96	28	-57	-99	4.72E-7	2.12E-6	8.27E-6
MALME-3M	0.602	1.712	1.699	1.732	1.227	0.931	0.077	99	102	56	30	-87	1.72E-6	1.79E-5	4.80E-5
M14	0.428	1.574	1.486	1.556	0.504	0.478	0.049	92	98	7	4	-89	3.37E-7	1.11E-5	3.84E-5
MDA-MB-435	0.562	2.409	2.398	2.287	0.412	0.866	0.020	99	93	-27	16	-97	2.30E-7	.	3.87E-5
SK-MEL-2	1.611	3.184	3.119	3.094	2.034	2.107	0.073	96	94	27	32	-95	4.54E-7	1.77E-5	4.38E-5
SK-MEL-28	0.646	1.981	1.915	1.900	1.265	1.238	0.021	95	94	46	44	-97	8.39E-7	2.06E-5	4.66E-5
SK-MEL-5	1.258	3.297	3.250	3.168	1.203	0.522	0.005	98	94	-4	-59	-100	2.79E-7	9.02E-7	6.96E-6
UACC-257	0.788	2.110	2.099	2.021	1.490	1.027	0.059	99	93	53	18	-93	1.23E-6	1.46E-5	4.13E-5
UACC-62	0.740	2.597	2.436	2.394	1.270	0.746	0.007	91	89	29	0	-99	4.42E-7	1.01E-5	3.21E-5
**Ovarian Cancer**															
IGROV1	0.491	1.970	2.136	2.336	1.170	0.639	0.111	111	125	46	10	-77	8.88E-7	1.30E-5	4.86E-5
OVCAR-3	0.610	1.900	1.925	1.878	0.630	0.706	0.090	102	98	2	7	-85	3.16E-7	1.20E-5	4.17E-5
OVCAR-4	0.855	2.123	2.082	2.017	1.620	1.231	0.034	97	92	60	30	-96	2.17E-6	1.72E-5	4.30E-5
OVCAR-5	0.523	1.449	1.373	1.291	0.713	0.665	0.005	92	83	21	15	-99	3.37E-7	1.36E-5	3.72E-5
OVCAR-8	0.394	1.813	1.777	1.784	0.421	0.297	0.057	97	98	2	-25	-86	3.16E-7	1.18E-6	2.60E-5
NCI/ADR-RES	0.514	1.974	1.983	1.940	0.393	0.375	0.030	101	98	-24	-27	-94	2.47E-7	6.38E-7	2.20E-5
SK-OV-3	0.822	1.959	2.007	1.963	1.370	0.884	0.037	104	100	48	5	-95	9.22E-7	1.13E-5	3.54E-5
**Renal Cancer**															
786-0	0.606	2.390	2.316	2.252	1.321	0.726	0.060	96	92	40	7	-90	6.46E-7	1.17E-5	3.85E-5
A498	1.529	2.333	2.215	2.096	2.098	2.115	0.093	85	70	71	73	-94	1.37E-5	2.74E-5	5.45E-5
ACHN	0.357	1.507	1.551	1.483	0.793	0.484	-0.001	104	98	38	11	-100	6.28E-7	1.26E-5	3.55E-5
CAKI-1	0.633	2.124	2.072	2.012	1.392	0.721	0.011	97	92	51	6	-98	1.05E-6	1.14E-5	3.44E-5
RXF 393	1.036	1.348	1.327	1.298	0.860	0.742	0.046	93	84	-17	-28	-96	2.17E-7	6.79E-7	2.10E-5
SN12C	0.558	2.041	1.993	1.963	0.800	0.527	0.007	97	95	16	-6	-99	3.72E-7	5.57E-6	3.00E-5
TK-10	1.130	2.170	2.055	1.971	1.830	1.431	0.028	89	81	67	29	-98	2.83E-6	1.69E-5	4.21E-5
UO-31	0.596	2.364	2.119	2.105	1.481	0.749	0.075	86	85	50	9	-88	1.00E-6	1.23E-5	4.07E-5
**Prostate Cancer**															
PC-3	0.500	1.998	1.987	1.948	0.982	0.815	0.136	99	97	32	21	-73	5.29E-7	1.67E-5	5.70E-5
DU-145	0.494	1.660	1.676	1.624	0.681	0.559	0.062	101	97	16	6	-88	3.80E-7	1.15E-5	3.95E-5
**Breast Cancer**															
MCF7	0.445	1.981	1.806	1.893	0.709	0.458	0.021	89	94	17	1	-95	3.75E-7	1.02E-5	3.38E-5
MDA-MB-231/ATCC	0.643	1.333	1.319	1.288	0.680	0.847	0.018	98	94	5	30	-97	3.11E-7	1.71E-5	4.24E-5
HS 578T	1.428	2.372	2.355	2.277	1.486	1.417	0.670	98	90	6	0	-53	3.00E-7	7.66E-6	8.73E-5
BT-549	1.245	2.110	2.079	1.998	1.457	0.961	0.068	96	87	24	-23	-95	3.90E-7	3.29E-6	2.39E-5
T-47D	0.725	1.697	1.616	1.599	1.307	0.833	0.060	92	90	60	11	-92	1.59E-6	1.28E-5	3.93E-5
MDA-MB-468	0.842	1.396	1.384	1.350	0.779	0.408	0.025	98	92	-7	-52	-97	2.63E-7	8.40E-7	9.22E-6

Compound **3u**

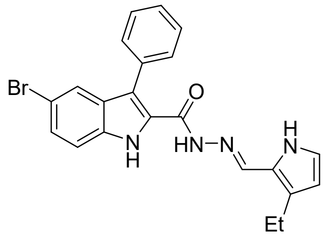
Br
O
HN
N
H
N
H
Et

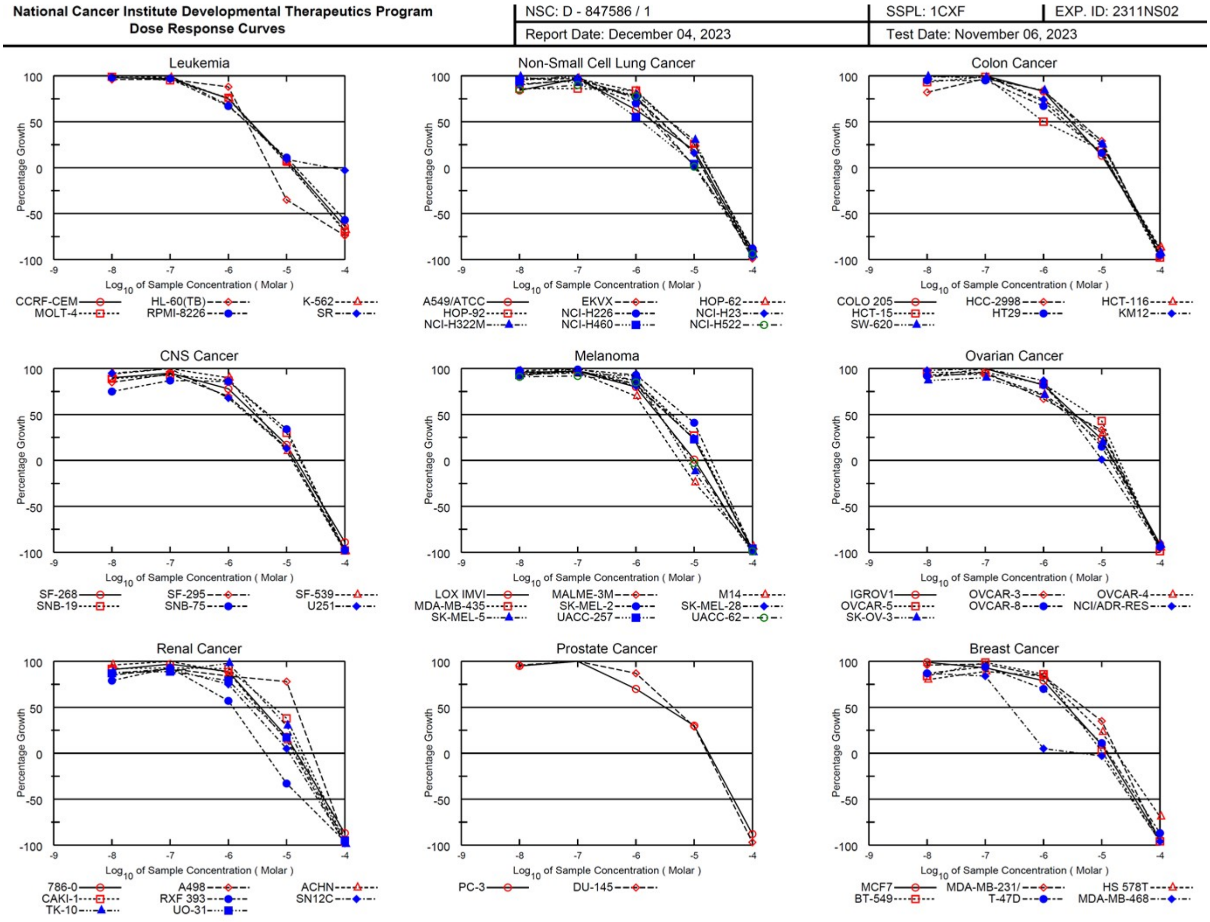
National Cancer Institute Developmental Therapeutics Program
Dose Response Curves
NSC: D - 847586 / 1
Report Date: December 04, 2023
SSPL: 1CXF
EXP. ID: 2311NS02
Test Date: November 06, 2023
Leukemia
Non-Small Cell Lung Cancer
Colon Cancer
CNS Cancer
Melanoma
Ovarian Cancer
Renal Cancer
Prostate Cancer
Breast Cancer
Percentage Growth
Log10 of Sample Concentration (Molar)
CCRF-CEM
HL-60(TB)
K-562
MOLT-4
RPMI-8226
SR
A549/ATCC
EKVX
HOP-62
HOP-92
NCI-H226
NCI-H23
NCI-H322M
NCI-H460
NCI-H522
COLO 205
HCC-2998
HCT-116
HCT-15
HT29
KM12
SW-620
SF-268
SF-295
SF-539
SNB-19
SNB-75
U251
LOX IMVI
MALME-3M
M14
MDA-MB-435
SK-MEL-2
SK-MEL-28
SK-MEL-5
UACC-257
UACC-62
IGROV1
OVCAR-3
OVCAR-4
OVCAR-5
OVCAR-8
NCI/ADR-RES
SK-OV-3
786-0
A498
ACHN
CAKI-1
RXF 393
SN12C
TK-10
UO-31
PC-3
DU-145
MCF7
MDA-MB-231/
HS 578T
BT-549
T-47D
MDA-MB-468

National Cancer Institute Developmental Therapeutics Program
In-Vitro Testing Results

NSC : D - 847586 / 1	Experiment ID : 2311NS02	Test Type : 08	Units : Molar
Report Date : December 04, 2023	Test Date : November 06, 2023	QNS :	MC :
COMI : TO63	Stain Reagent : SRB Dual-Pass Related	SSPL : 1CXF	

			Log10 Concentration												
	Time		Mean Optical Densities					Percent Growth							
Panel/Cell Line	Zero	Ctrl	-8.0	-7.0	-6.0	-5.0	-4.0	-8.0	-7.0	-6.0	-5.0	-4.0	GI50	TGI	LC50
Leukemia															
CCRF-CEM	0.546	3.038	2.980	2.968	2.411	0.746	0.196	98	97	75	8	-64	2.35E-6	1.29E-5	6.38E-5
HL-60(TB)	0.835	3.077	2.981	2.984	2.803	0.544	0.217	96	96	88	-35	-74	2.03E-6	5.20E-6	2.43E-5
K-562	0.279	2.073	2.083	2.036	1.623	0.365	0.089	101	98	75	5	-68	2.27E-6	1.16E-5	5.62E-5
MOLT-4	0.563	2.491	2.479	2.392	2.021	0.704	0.169	99	95	76	7	-70	2.37E-6	1.24E-5	5.51E-5
RPMI-8226	0.827	2.245	2.218	2.200	1.781	0.989	0.354	98	97	67	11	-57	2.04E-6	1.47E-5	7.86E-5
SR	0.219	0.594	0.616	0.619	0.474	0.254	0.213	106	107	68	9	-3	2.02E-6	5.90E-5	> 1.00E-4
Non-Small Cell Lung Cancer															
A549/ATCC	0.217	1.313	1.135	1.286	0.913	0.425	0.016	84	97	63	19	-93	2.00E-6	1.48E-5	4.15E-5
EKVX	0.536	1.764	1.645	1.715	1.483	0.735	0.007	90	96	77	16	-99	2.79E-6	1.38E-5	3.77E-5
HOP-62	0.641	1.783	1.733	1.744	1.593	0.933	0.072	96	97	83	26	-89	3.77E-6	1.67E-5	4.58E-5
HOP-92	1.403	1.855	1.794	1.792	1.781	1.519	0.121	86	86	84	26	-91	3.80E-6	1.66E-5	4.43E-5
NCI-H226	0.894	1.679	1.612	1.642	1.445	0.908	0.106	91	95	70	2	-88	1.97E-6	1.05E-5	3.77E-5
NCI-H23	0.505	1.709	1.674	1.687	1.410	0.699	0.015	97	98	75	16	-97	2.66E-6	1.39E-5	3.83E-5
NCI-H322M	0.560	2.127	2.113	1.998	1.802	1.031	0.026	99	92	79	30	-95	3.93E-6	1.74E-5	4.34E-5
NCI-H460	0.286	2.283	2.193	2.312	1.376	0.364	0.024	95	101	55	4	-92	1.23E-6	1.10E-5	3.67E-5
NCI-H522	1.112	2.848	2.595	2.671	2.466	1.121	0.064	85	90	78	1	-94	2.30E-6	1.01E-5	3.41E-5
Colon Cancer															
COLO 205	0.476	2.094	2.130	2.201	1.823	0.683	0.052	102	107	83	13	-89	2.96E-6	1.33E-5	4.13E-5
HCC-2998	0.821	2.728	2.385	2.676	2.414	1.372	0.026	82	97	84	29	-97	4.11E-6	1.70E-5	4.24E-5
HCT-116	0.267	2.062	2.044	2.157	1.565	0.561	0.036	99	105	72	16	-87	2.50E-6	1.44E-5	4.40E-5
HCT-15	0.311	2.034	1.916	2.022	1.180	0.631	0.007	93	99	50	19	-98	1.03E-6	1.44E-5	3.88E-5
HT29	0.201	1.353	1.293	1.300	0.974	0.389	0.010	95	95	67	16	-95	2.17E-6	1.40E-5	3.93E-5
KM12	0.615	2.343	2.543	2.440	1.902	1.059	0.030	112	106	74	26	-95	3.17E-6	1.63E-5	4.23E-5
SW-620	0.279	1.786	1.776	1.750	1.551	0.660	0.020	99	98	84	25	-93	3.82E-6	1.64E-5	4.33E-5
CNS Cancer															
SF-268	0.838	2.192	2.062	2.126	1.894	1.065	0.092	90	95	78	17	-89	2.86E-6	1.44E-5	4.27E-5
SF-295	0.431	1.922	1.691	1.829	1.479	0.638	0.007	85	94	70	14	-98	2.29E-6	1.33E-5	3.71E-5
SF-539	0.637	2.041	1.955	2.075	1.903	0.779	0.007	94	102	90	10	-99	3.17E-6	1.24E-5	3.56E-5
SNB-19	0.692	1.913	1.783	1.832	1.757	1.062	0.014	89	93	87	30	-98	4.50E-6	1.72E-5	4.22E-5
SNB-75	1.075	1.955	1.736	1.838	1.831	1.376	0.022	75	87	86	34	-98	4.94E-6	1.81E-5	4.34E-5
U251	0.199	1.125	1.080	1.153	0.830	0.324	0.007	95	103	68	13	-96	2.15E-6	1.33E-5	3.78E-5
Melanoma															
LOX IMVI	0.472	3.079	2.979	3.001	2.571	0.496	0.005	96	97	80	1	-99	2.42E-6	1.02E-5	3.23E-5
MALME-3M	0.602	1.712	1.619	1.691	1.520	0.853	0.013	92	98	83	23	-98	3.50E-6	1.54E-5	4.00E-5
M14	0.428	1.574	1.543	1.533	1.225	0.326	0.028	97	96	70	-24	-94	1.62E-6	5.56E-6	2.37E-5
MDA-MB-435	0.562	2.409	2.299	2.345	2.171	1.057	0.015	94	96	87	27	-97	4.12E-6	1.64E-5	4.16E-5
SK-MEL-2	1.611	3.184	3.152	3.166	3.080	2.258	0.054	98	99	93	41	-97	6.77E-6	1.99E-5	4.58E-5
SK-MEL-28	0.646	1.981	1.904	1.949	1.726	0.961	0.011	94	98	81	24	-98	3.46E-6	1.56E-5	4.01E-5
SK-MEL-5	1.258	3.297	3.172	3.199	3.132	1.111	0.004	94	95	92	-12	-100	2.54E-6	7.71E-6	2.72E-5
UACC-257	0.788	2.110	2.016	2.123	1.939	1.091	0.028	93	101	87	23	-96	3.78E-6	1.56E-5	4.08E-5
UACC-62	0.740	2.597	2.434	2.454	2.313	0.715	0.010	91	92	85	-3	-99	2.48E-6	9.14E-6	3.08E-5
Ovarian Cancer															
IGROV1	0.491	1.970	2.045	2.023	1.710	0.832	0.047	105	104	82	23	-91	3.52E-6	1.60E-5	4.40E-5
OVCAR-3	0.610	1.900	1.784	1.854	1.477	1.037	0.025	91	96	67	33	-96	3.20E-6	1.81E-5	4.40E-5
OVCAR-4	0.855	2.123	2.040	2.049	1.763	1.239	0.043	93	94	72	30	-95	3.33E-6	1.74E-5	4.37E-5
OVCAR-5	0.523	1.449	1.415	1.416	1.292	0.924	0.007	96	96	83	43	-99	6.77E-6	2.02E-5	4.54E-5
OVCAR-8	0.394	1.813	1.699	1.839	1.559	0.611	0.026	92	102	82	15	-94	3.02E-6	1.38E-5	3.98E-5
NCI/ADR-RES	0.514	1.974	1.943	2.019	1.778	0.532	0.032	98	103	87	1	-94	2.68E-6	1.03E-5	3.46E-5
SK-OV-3	0.822	1.959	1.810	1.851	1.630	1.050	0.069	87	90	71	20	-92	2.59E-6	1.51E-5	4.24E-5
Renal Cancer															
786-0	0.606	2.390	2.231	2.334	2.194	0.919	0.079	91	97	89	18	-87	3.51E-6	1.47E-5	4.43E-5
A498	1.529	2.333	2.215	2.271	2.201	2.154	0.090	85	92	84	78	-94	1.45E-5	2.83E-5	5.54E-5
ACHN	0.357	1.507	1.467	1.550	1.368	0.521	0.002	96	104	88	14	-99	3.27E-6	1.33E-5	3.67E-5
CAKI-1	0.633	2.124	2.002	2.014	2.010	1.201	0.025	92	93	92	38	-96	6.04E-6	1.92E-5	4.53E-5
RXF 393	1.036	1.348	1.281	1.326	1.213	0.697	0.037	79	93	57	-33	-96	1.19E-6	4.31E-6	1.87E-5
SN12C	0.558	2.041	1.847	1.916	1.667	0.632	0.009	87	92	75	5	-98	2.26E-6	1.12E-5	3.40E-5
TK-10	1.130	2.170	2.050	2.053	2.151	1.438	0.011	88	89	98	30	-99	5.05E-6	1.70E-5	4.16E-5
UO-31	0.596	2.364	2.140	2.175	1.988	0.905	0.029	87	89	79	17	-95	2.94E-6	1.43E-5	3.97E-5
Prostate Cancer															
PC-3	0.500	1.998	1.929	2.005	1.542	0.944	0.061	95	100	70	30	-88	3.09E-6	1.79E-5	4.76E-5
DU-145	0.494	1.660	1.610	1.684	1.506	0.829	0.015	96	102	87	29	-97	4.30E-6	1.69E-5	4.23E-5
Breast Cancer															
MCF7	0.445	1.981	1.965	1.867	1.665	0.590	0.026	99	93	79	9	-94	2.63E-6	1.23E-5	3.75E-5
MDA-MB-231/ATCC	0.643	1.333	1.302	1.313	1.225	0.887	0.026	96	97	84	35	-96	5.02E-6	1.86E-5	4.47E-5
HS 578T	1.428	2.372	2.180	2.263	2.224	1.642	0.440	80	89	84	23	-69	3.60E-6	1.76E-5	6.18E-5
BT-549	1.245	2.110	1.970	2.103	1.990	1.265	0.055	84	99	86	2	-96	2.70E-6	1.06E-5	3.42E-5
T-47D	0.725	1.697	1.573	1.638	1.404	0.831	0.095	87	94	70	11	-87	2.17E-6	1.29E-5	4.19E-5
MDA-MB-468	0.842	1.396	1.326	1.309	0.872	0.815	0.038	87	84	5	-3	-96	2.72E-7	4.20E-6	3.21E-5

Compound **3v**

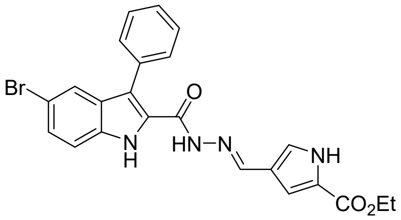
Br
O
HN–N
N
H
NH
CO_2Et

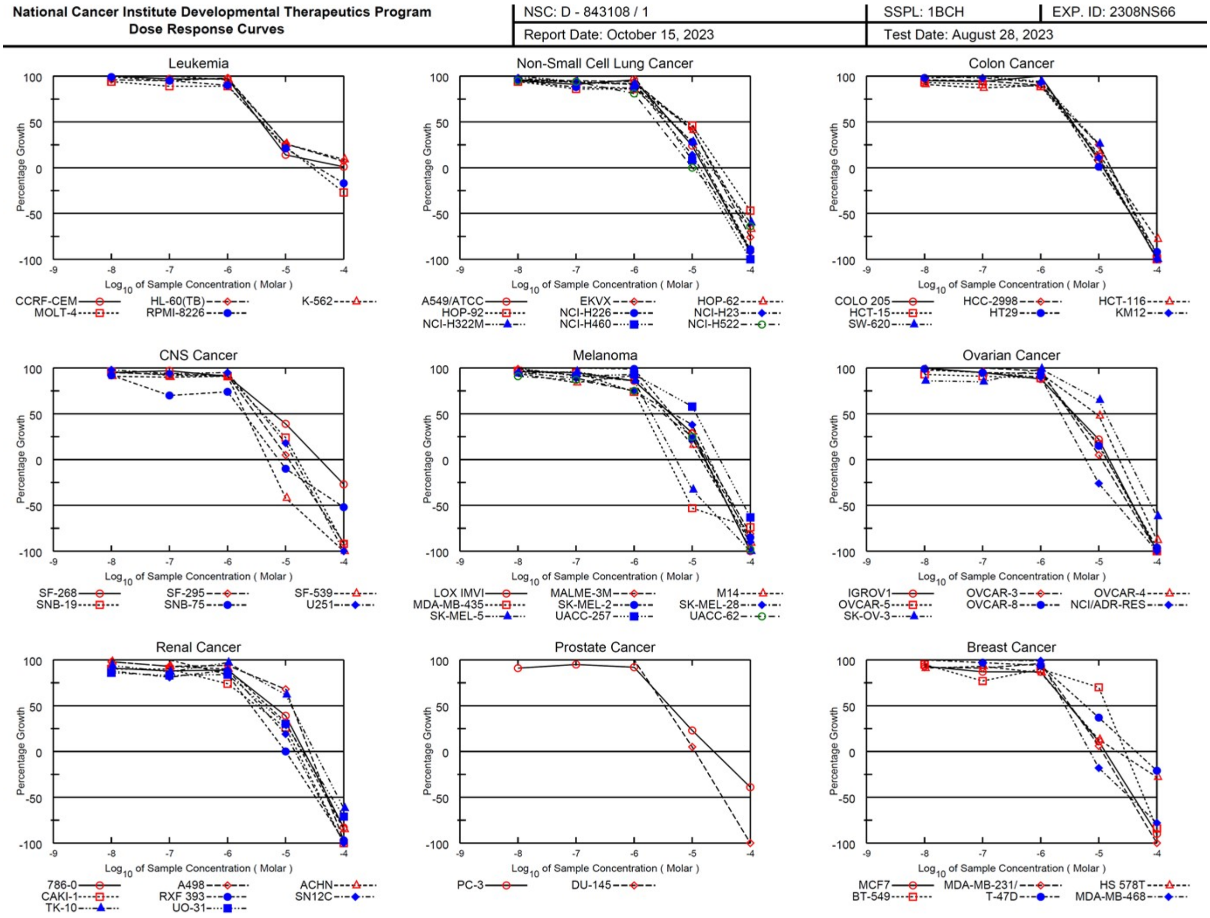
National Cancer Institute Developmental Therapeutics Program
Dose Response Curves
NSC: D - 843108 / 1
SSPL: 1BCH
EXP. ID: 2308NS66
Report Date: October 15, 2023
Test Date: August 28, 2023
Leukemia
Non-Small Cell Lung Cancer
Colon Cancer
CNS Cancer
Melanoma
Ovarian Cancer
Renal Cancer
Prostate Cancer
Breast Cancer
Percentage Growth
Log_{10} of Sample Concentration (Molar)
CCRF-CEM
HL-60(TB)
K-562
MOLT-4
RPMI-8226
A549/ATCC
EKVX
HOP-62
HOP-92
NCI-H226
NCI-H23
NCI-H322M
NCI-H460
NCI-H522
COLO 205
HCC-2998
HCT-116
HCT-15
HT29
KM12
SW-620
SF-268
SF-295
SF-539
SNB-19
SNB-75
U251
LOX IMVI
MALME-3M
M14
MDA-MB-435
SK-MEL-2
SK-MEL-28
SK-MEL-5
UACC-257
UACC-62
IGROV1
OVCAR-3
OVCAR-4
OVCAR-5
OVCAR-8
NCI/ADR-RES
SK-OV-3
786-0
A498
ACHN
CAKI-1
RXF 393
SN12C
TK-10
UO-31
PC-3
DU-145
MCF7
MDA-MB-231/
HS 578T
BT-549
T-47D
MDA-MB-468

National Cancer Institute Developmental Therapeutics Program
In-Vitro Testing Results

NSC : D - 843108 / 1	Experiment ID : 2308NS66	Test Type : 08	Units : Molar
Report Date : October 15, 2023	Test Date : August 28, 2023	QNS :	MC :
COMI : To46	Stain Reagent : SRB Dual-Pass Related	SSPL : 1BCH	

			Log10 Concentration													
	Time		Mean Optical Densities					Percent Growth								
Panel/Cell Line	Zero	Ctrl	-8.0	-7.0	-6.0	-5.0	-4.0	-8.0	-7.0	-6.0	-5.0	-4.0	GI50	TGI	LC50
Leukemia															
CCRF-CEM	0.489	2.559	2.615	2.494	2.492	0.789	0.510	103	97	97	14	1	3.70E-6	> 1.00E-4	> 1.00E-4
HL-60(TB)	0.609	2.682	2.606	2.586	2.639	1.157	0.749	96	95	98	26	7	4.68E-6	> 1.00E-4	> 1.00E-4
K-562	0.255	2.238	2.292	2.237	2.159	0.780	0.432	103	100	96	26	9	4.59E-6	> 1.00E-4	> 1.00E-4
MOLT-4	0.491	2.709	2.586	2.474	2.468	1.003	0.360	94	89	89	23	-27	3.91E-6	2.91E-5	> 1.00E-4
RPMI-8226	0.768	2.490	2.471	2.401	2.321	1.138	0.640	99	95	90	21	-17	3.84E-6	3.65E-5	> 1.00E-4
Non-Small Cell Lung Cancer															
A549/ATCC	0.294	1.978	1.893	1.825	1.906	0.704	0.027	95	91	96	24	-91	4.37E-6	1.63E-5	4.42E-5
EKVX	0.960	2.566	2.503	2.484	2.474	1.641	0.234	96	95	94	42	-76	7.14E-6	2.29E-5	6.07E-5
HOP-62	0.633	2.173	2.106	2.059	2.055	1.263	0.207	96	93	92	41	-67	6.66E-6	2.39E-5	6.92E-5
HOP-92	1.139	1.785	1.748	1.696	1.692	1.437	0.601	94	86	86	46	-47	7.99E-6	3.12E-5	> 1.00E-4
NCI-H226	1.037	1.796	1.758	1.703	1.697	1.248	0.112	95	88	87	28	-89	4.21E-6	1.73E-5	4.62E-5
NCI-H23	0.591	1.923	1.893	1.829	1.804	0.780	0.049	98	93	91	14	-92	3.42E-6	1.36E-5	4.04E-5
NCI-H322M	0.811	2.182	2.224	2.105	2.053	1.191	0.321	103	94	91	28	-60	4.42E-6	2.06E-5	7.61E-5
NCI-H460	0.259	2.634	2.697	2.670	2.644	0.438	-0.002	103	102	100	8	-100	3.49E-6	1.17E-5	3.43E-5
NCI-H522	1.295	3.037	2.946	2.934	2.700	1.304	0.470	95	94	81	0	-64	2.41E-6	1.02E-5	6.12E-5
Colon Cancer															
COLO 205	0.551	2.497	2.402	2.383	2.505	0.707	0.006	95	94	100	8	-99	3.51E-6	1.19E-5	3.48E-5
HCC-2998	0.668	2.623	2.551	2.516	2.427	1.166	0.031	96	95	90	25	-95	4.17E-6	1.62E-5	4.21E-5
HCT-116	0.301	2.836	2.614	2.512	2.575	0.693	0.068	91	87	90	15	-78	3.42E-6	1.47E-5	5.05E-5
HCT-15	0.311	2.557	2.401	2.364	2.317	0.573	-0.024	93	91	89	12	-100	3.21E-6	1.27E-5	3.57E-5
HT29	0.217	1.609	1.584	1.621	1.653	0.236	0.017	98	101	103	1	-92	3.33E-6	1.03E-5	3.53E-5
KM12	0.494	2.483	2.525	2.443	2.347	0.719	.	102	98	93	11	-100	3.37E-6	1.26E-5	3.55E-5
SW-620	0.268	2.045	2.101	2.077	1.935	0.727	-0.002	103	102	94	26	-100	4.41E-6	1.60E-5	4.01E-5
CNS Cancer															
SF-268	1.072	2.868	2.776	2.806	2.701	1.766	0.784	95	97	91	39	-27	6.04E-6	3.88E-5	> 1.00E-4
SF-295	1.309	3.277	3.180	3.147	3.126	1.401	0.115	95	93	92	5	-91	3.04E-6	1.12E-5	3.72E-5
SF-539	0.818	2.447	2.293	2.279	2.296	0.471	-0.040	91	90	91	-42	-100	2.02E-6	4.80E-6	1.35E-5
SNB-19	0.564	1.903	1.856	1.790	1.781	0.886	0.045	96	92	91	24	-92	4.08E-6	1.61E-5	4.34E-5
SNB-75	1.773	2.711	2.635	2.434	2.470	1.605	0.855	92	70	74	-10	-52	1.95E-6	7.70E-6	9.06E-5
U251	0.353	1.764	1.739	1.680	1.689	0.602	-0.044	98	94	95	18	-100	3.80E-6	1.41E-5	3.76E-5
Melanoma															
LOX IMVI	0.406	2.859	2.768	2.733	2.508	1.129	-0.076	96	95	86	29	-100	4.31E-6	1.69E-5	4.11E-5
MALME-3M	0.616	1.681	1.673	1.601	1.531	0.929	0.120	99	92	86	29	-81	4.32E-6	1.85E-5	5.28E-5
M14	0.601	2.581	2.468	2.273	2.417	0.913	0.054	94	84	92	16	-91	3.54E-6	1.40E-5	4.13E-5
MDA-MB-435	0.676	2.887	2.816	2.770	2.306	0.320	0.179	97	95	74	-53	-74	1.54E-6	3.83E-6	9.53E-6
SK-MEL-2	0.850	2.043	2.058	2.097	2.029	1.120	0.128	101	104	99	23	-85	4.37E-6	1.62E-5	4.73E-5
SK-MEL-28	0.751	2.144	2.069	1.996	1.789	1.283	0.073	95	89	75	38	-90	4.73E-6	1.98E-5	4.85E-5
SK-MEL-5	0.904	3.201	3.059	3.120	2.882	0.605	-0.078	94	96	86	-33	-100	2.01E-6	5.27E-6	1.79E-5
UACC-257	0.919	2.288	2.292	2.172	2.187	1.716	0.338	100	91	93	58	-63	1.17E-5	3.01E-5	7.78E-5
UACC-62	0.974	2.847	2.685	2.607	2.386	1.440	0.020	91	87	75	25	-98	3.18E-6	1.59E-5	4.07E-5
Ovarian Cancer															
IGROV1	0.436	2.147	2.165	2.065	1.938	0.813	-0.028	101	95	88	22	-100	3.75E-6	1.51E-5	3.89E-5
OVCAR-3	0.626	1.958	2.088	2.016	1.916	0.692	-0.030	110	104	97	5	-100	3.23E-6	1.11E-5	3.34E-5
OVCAR-4	0.715	1.832	1.811	1.772	1.767	1.250	0.089	98	95	94	48	-88	9.00E-6	2.26E-5	5.28E-5
OVCAR-5	0.447	1.621	1.535	1.516	1.493	0.647	-0.069	93	91	89	17	-100	3.49E-6	1.40E-5	3.74E-5
OVCAR-8	0.337	1.868	1.849	1.789	1.781	0.573	0.013	99	95	94	15	-96	3.65E-6	1.37E-5	3.85E-5
NCI/ADR-RES	0.357	1.333	1.380	1.283	1.240	0.265	-0.013	105	95	90	-26	-100	2.23E-6	6.00E-6	2.12E-5
SK-OV-3	0.838	1.889	1.744	1.732	1.876	1.523	0.318	86	85	99	65	-62	1.31E-5	3.25E-5	8.03E-5
Renal Cancer															
786-0	0.777	3.041	2.832	2.774	2.784	1.661	0.129	91	88	89	39	-83	6.01E-6	2.08E-5	5.33E-5
A498	1.209	2.202	2.186	2.131	2.145	1.885	0.042	98	93	94	68	-97	1.29E-5	2.59E-5	5.21E-5
ACHN	0.374	1.700	1.673	1.611	1.569	0.795	0.055	98	93	90	32	-85	4.87E-6	1.87E-5	4.99E-5
CAKI-1	0.495	1.736	1.613	1.612	1.413	0.823	-0.008	90	90	74	26	-100	3.20E-6	1.62E-5	4.02E-5
RXF 393	0.750	1.442	1.452	1.444	1.334	0.752	0.025	101	100	84	0	-97	2.56E-6	1.01E-5	3.30E-5
SN12C	0.631	2.311	2.106	1.988	2.129	0.955	-0.062	88	81	89	19	-100	3.63E-6	1.45E-5	3.81E-5
TK-10	1.084	2.183	2.122	2.037	2.146	1.766	0.417	94	87	97	62	-62	1.25E-5	3.18E-5	8.07E-5
UO-31	0.664	2.052	1.855	1.809	1.825	1.077	0.193	86	83	84	30	-71	4.21E-6	1.97E-5	6.19E-5
Prostate Cancer															
PC-3	0.562	2.244	2.097	2.165	2.112	0.949	0.345	91	95	92	23	-39	4.07E-6	2.36E-5	> 1.00E-4
DU-145	0.375	1.634	1.687	1.666	1.634	0.438	-0.036	104	103	100	5	-100	3.36E-6	1.12E-5	3.34E-5
Breast Cancer															
MCF7	0.450	2.385	2.245	2.130	2.124	0.655	0.047	93	87	87	11	-90	3.03E-6	1.28E-5	4.03E-5
MDA-MB-231/ATCC	0.566	1.111	1.068	1.063	1.091	0.600	-0.003	92	91	96	6	-100	3.27E-6	1.14E-5	3.38E-5
HS 578T	1.374	2.564	2.457	2.481	2.399	1.526	0.985	91	93	86	13	-28	3.11E-6	2.04E-5	> 1.00E-4
BT-549	1.398	2.929	2.856	2.582	2.783	2.466	0.226	95	77	90	70	-84	1.34E-5	2.84E-5	6.02E-5
T-47D	0.747	1.941	1.972	1.903	1.873	1.186	0.593	103	97	94	37	-21	5.88E-6	4.37E-5	> 1.00E-4
MDA-MB-468	0.773	1.451	1.472	1.468	1.441	0.631	0.171	103	102	99	-18	-78	2.60E-6	6.96E-6	3.40E-5

Figure S4. Screening derivatives **3a–x** against T‑47D with two different concentration (10 µM and 50 µM)

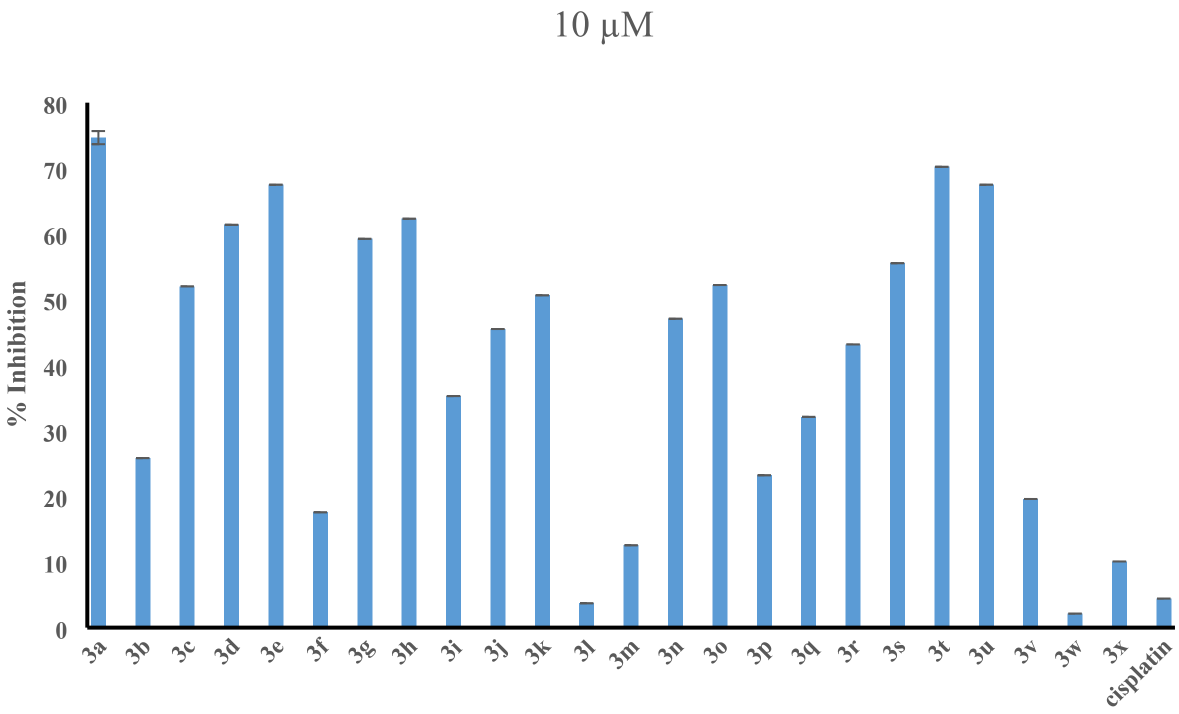

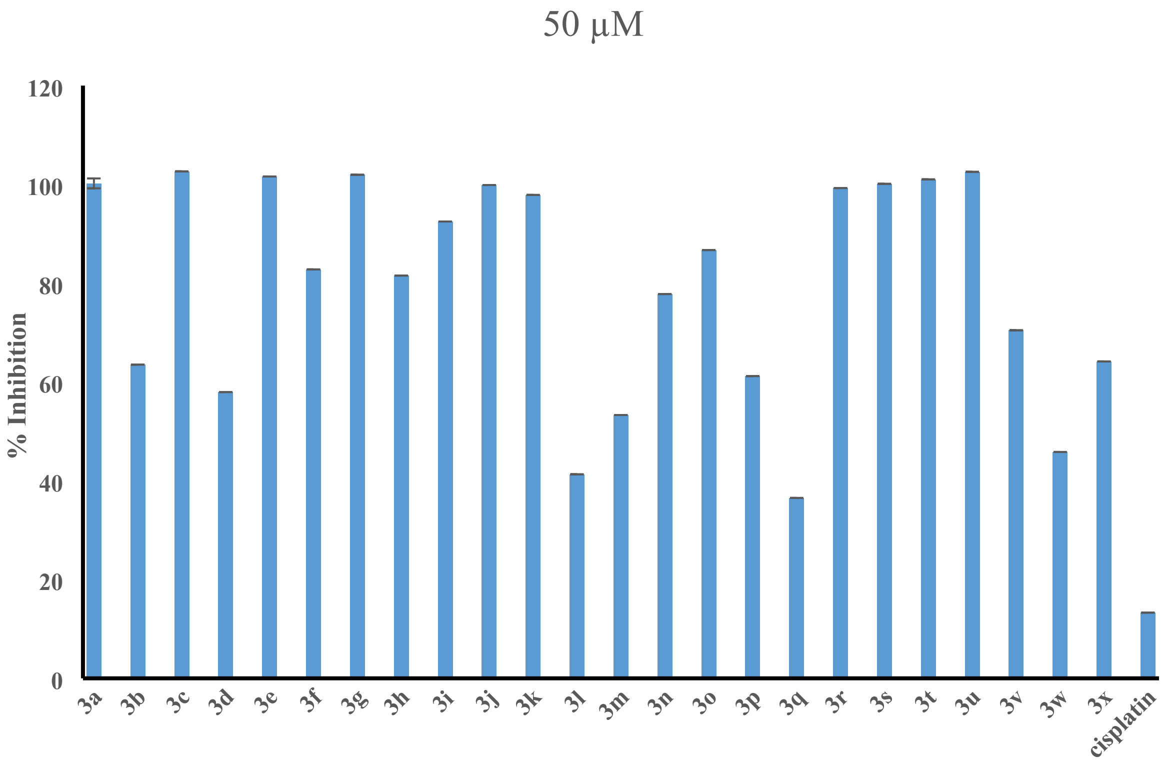

Figure S5. Correlations between IC_{50} values and GI_{50} values of the tested compounds against the T47D breast cancer cell line (R^2=0.7884).

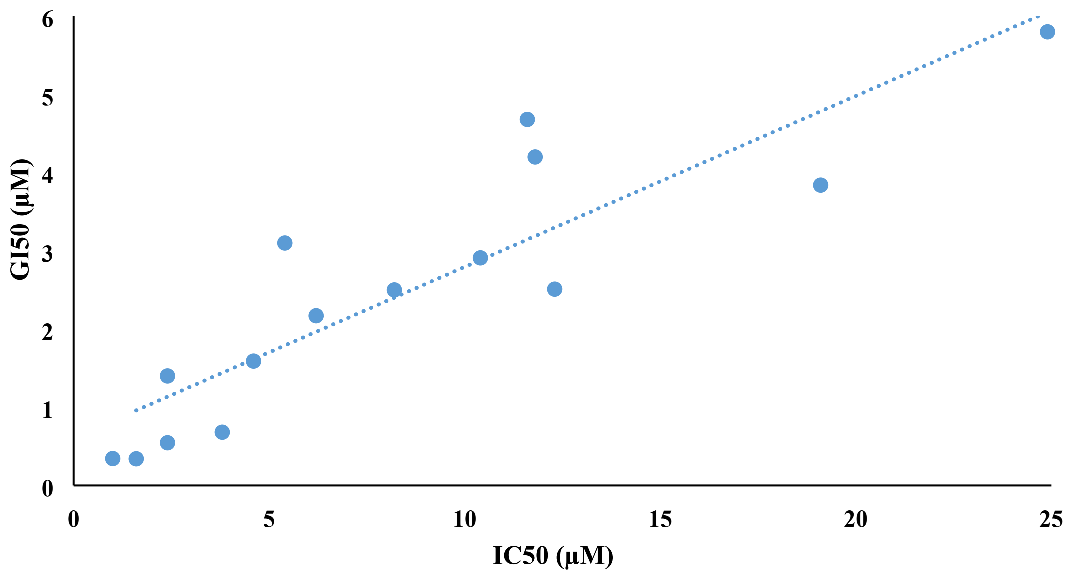

Fig. S6. Overlay between the co-crystallized colchicine (green) and the re-docked colchicine conformer using the GoldScore scoring function. The RMSD between the heavy atoms was calculated to be 1.33 Å.

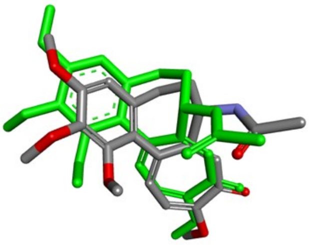

Fig. S7. Overlay between the co-crystallized exemestane (green) and the re-docked exemestane conformer using the GoldScore scoring function. The RMSD between the heavy atoms was calculated to be 0.46 Å.

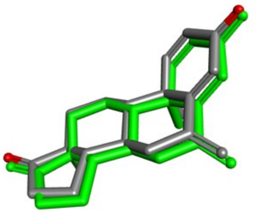

Table S8. The physicochemical properties of the newly synthesised furanyl- and thiophenyl-3-phenyl-1*H*-indole-2-carbohydrazide derivatives.

**Derivatives**	**Molecular weight (g/mol)**	***M*logP**	**Hydrogen bond donor**	**Hydrogen bond acceptor**	**Topological polar surface area (Å^2)**	**Rotatable bond count**
**3a**	407.29	3.112	3	3	73.04	4
**3b**	407.29	3.112	3	3	73.04	4
**3c**	435.35	3.542	3	3	73.04	4
**3d**	421.32	3.329	2	3	62.18	4
**3e**	486.18	3.704	3	3	73.04	4
**3f**	565.07	4.019	3	3	73.04	4
**3g**	441.73	3.597	3	3	73.04	4
**3h**	441.73	3.597	3	3	73.04	4
**3i**	479.36	3.229	3	5	99.34	7
**3j**	479.36	3.229	3	5	99.34	7
**3k**	479.36	3.229	3	5	99.34	7
**3l**	451.3	2.806	4	5	110.34	5
**3m**	451.3	2.806	4	5	110.34	5
**3n**	520.42	2.483	3	6	102.58	5
**3o**	520.42	2.483	3	6	102.58	5
**3p**	452.29	3.605	3	5	118.86	5
**3q**	452.29	3.199	3	5	118.86	5
**3r**	480.35	3.628	3	5	118.86	5
**3s**	435.35	3.542	3	3	73.04	5
**3t**	435.35	3.542	3	3	73.04	5
**3u**	435.35	3.542	3	3	73.04	5
**3v**	479.36	3.229	3	5	99.34	7
**3w**	451.3	2.806	4	5	110.34	5
**3x**	520.42	2.483	3	6	102.58	5

Table S9. Toxicity evaluation of the most active compounds.

	**3a**	**3e**	**3h**	**3p**	**3r**	**3t**
hERG blocker	0.149	0.277	0.268	0.341	0.139	0.293
AMES mutagenicity	0.562	0.471	0.473	0.867	0.697	0.546
Human hepatotoxicity	0.898	0.841	0.885	0.868	0.939	0.857
RPMI-8226 immunotoxicity	0.157	0.121	0.125	0.082	0.060	0.090

[a]All values represent toxicity probabilities within the range of 0 to 1.
